# Arab female and male perceptions of factors facilitating and inhibiting their physical activity: Findings from a qualitative study in the Middle East

**DOI:** 10.1371/journal.pone.0199336

**Published:** 2018-07-16

**Authors:** Tam Truong Donnelly, Al-Anoud bint Mohammed Al-Thani, Kathleen Benjamin, Al-Hareth Al-Khater, Tak Shing Fung, Mohamed Ahmedna, Ailsa Welch

**Affiliations:** 1 Faculty of Nursing and Medicine, University of Calgary, Calgary, Alberta, Canada; 2 Qatar Ministry of Public Health, Public Health Department, Doha, Qatar; 3 University of Calgary-Qatar, Doha, Qatar; 4 National Center for Cancer Care & Research, Hamad Medical Corporation, Doha, Qatar; 5 Qatar University, Doha, Qatar; 6 University of East Anglia, Norwich, Norfolk, United Kingdom; Anglia Ruskin University, UNITED KINGDOM

## Abstract

**Objectives:**

Physical inactivity is a leading global risk to health by contributing to obesity and other chronic diseases. Many chronic non-communicable diseases, such as cancer, diabetes, and cardiovascular diseases (CVDs), can be prevented and controlled by modifying lifestyle behaviors such as physical activity [PA]. However, prevalence of insufficient physical activity and obesity is high in the Middle East Region. In Qatar, the incidence rates of CVDs, diabetes, colon, and breast cancer have been rising rapidly. The purpose of this study was to explore facilitators and barriers influencing PA of adult Arab men and women living in Qatar and to understand what they think would be helpful to increase PA. The goal of the research is to identify culturally appropriate and effective interventions that improve the health of Arab population.

**Design:**

Using the socioecological model as the theoretical framework, we conducted an exploratory qualitative study with 128 Arab adult men and women living in Qatar. We utilized focus group interviews to collect the data and performed thematic analysis to generate themes.

**Results:**

At the individual level, perceived benefits of PA, presence of diseases, person’s will, motivation and goals, and time to exercise influenced the individual’s PA. At the sociocultural level, religious teachings of Islam, cultural, attitude, beliefs, and practices, and informal support influenced the participants’ PA. At the organizational and political level, physical environment to exercise, accessibility of facilities, organizational support, and health information about PA influenced their PA.

**Conclusion:**

Arab men and women are aware of the importance and benefits of PA. They have the motivation to be physically active, but in the absence of supportive environment, their knowledge might not translate into action. Creating supportive environments at multiple levels that are conducive to PA is warranted.

## Background

Physical inactivity is known as one of the leading global risk factors for non-communicable diseases (NCDs) worldwide [1]. It is estimated to cause about 27% of diabetes, 30% of ischemic heart diseases, and 21–25% of breast and colon cancer globally. The incidence of NCDs such as diabetes mellitus type 2 (diabetes), cardiovascular diseases (CVDs), and breast and colon cancer is high in the Eastern Mediterranean region (EMR) [1–4]; NCDs represents more than half of total causes of death. According to the 2006 Qatar World Health Survey (WHS) [5], 24% of the people surveyed in Qatar were normal weight, 39% were overweight, and nearly 29% were obese. In 2014, the World Health Organization (WHO) [6] reported that 42% of the population in Qatar was considered obese. The Supreme Council of Health (SCH) [7] in Qatar reported that Qatari population is at high risk of ischemic strokes due to hypertension, diabetes, and hypercholesterolemia. Indeed, CVDs are the leading causes of mortality and morbidity in Qatar [7]. Among the Gulf Cooperation Council (GCC) countries, Qatar has the highest prevalence of diabetes [8]. Moreover, 28% of the Qatari population was found to have metabolic syndrome such as raised blood pressure, fasting blood glucose, triglycerides and high-density lipoprotein cholesterol [9]. In the EMR, the incidence of breast cancer has increased substantially in the last two decades. In 2006, breast cancer was the leading cancer diagnosis for Qatari women with the incidence increasing significantly with age [10]. Apart from death and comorbidity, consequences of obesity due to physical inactivity include adverse effects on well-being, physical and psychological suffering, reduced role performance, emotional hardship to families and caregivers, and an increase in medical and health care costs [7, 11–13].

Studies have shown that many NCDs can be prevented and controlled by modifying lifestyle behaviors such as physical activity and healthy diet [14–18]. Results of a recent Cochrane review suggest that among people with depression, exercise may reduce the symptoms of depression [19]. Physical activity is fundamental in achieving energy balance and weight control [1]. The WHO [20] recommends that healthy adults 18–64 years of age should accumulate at least 150 minutes of moderate intensity aerobic activity per week, or 75 minutes of vigorous intensity, or an equivalent combination of moderate and vigorous-intensity aerobic activity in bouts of 10 minutes or more. According to the most recent statistics from WHO [21], in 2008, 31% of people 15 years of age and older worldwide had insufficient levels of physical activity. In the GCC countries, only 40% of men and 27% of women reported that they were physically active for at least 150 min per week. In the State of Qatar, nearly 50% of young adults 18–19 years of age had insufficient levels of physical activity and this rate increased substantially with age. Among people 60–69 years of age, 75% had insufficient levels of physical activity. According to the WHO survey, the prevalence of insufficient physical activity in Qatar among adult males aged 18 and above were 33.4% and females were 49.7% [22].

A review of the literature revealed barriers and facilitators influencing PA of Arabic-speaking adults. At an individual level, lack of time to exercise, lack of willpower, the presence of health conditions, fatigue/tiredness, ruining grooming efforts such as clothing and make-up for women, and lack of experience with exercise were reported as hindering physical activity [23–26]. At a sociocultural level, cultural and social norms such as women need to be accompanied by a male family member when going outdoors, public modesty, and the need to wear traditional dress, women’s roles and domestic responsibilities, social milieu that deemphasizes the importance of PA, lack of health discourse in society, and social belief and attitude towards women who try to be physically active, were reported as barriers [23, 25, 27–30]. At an organizational and political level, allocation of funding for sports, especially for women, i.e., limited access and funding for women to join exercise facilities such as sports clubs, jogging trails, swilling pools, lack of information on exercise, costs of gym memberships, and living in suburban or villages where there are less funding for sports programs were reported as barriers [25, 28, 31, 32].

At an environmental level, hot desert climate and lack of indoor walking trails and affordable exercise venues were identified as barriers [23, 25, 28]. In one qualitative study by Ali et al. [31], an example of intersecting barrier was illustrated, i.e., family discouraged a woman from buying a treadmill for home use (barrier at the social level) because there was not enough space for it in the home (barrier at the environmental level). On the other hand, facilitators for PA included the presence of health condition, feeling younger and healthier as a result of exercise, valuing individual responsibility, being religious, having supportive social systems, and having accessible and affordable recreational facilities nearby [25, 26, 31, 33].

Our review of the literature informed us that a person’s health-related decisions and behaviors are influenced by various factors at multiple levels; therefore, it would be important to develop interventions that address multiple levels and factors, targeting both the individual and the environment. There is a dearth of knowledge in PA of adult population in Qatar. Our understanding of the factors that influence the PA of Qatari population is limited. This study aimed to explore how Qatari adult men and women’s awareness, knowledge, and beliefs of PA influence their choices and behaviors in actively participating in PA. Such understanding is critical in tailoring culturally and socially appropriate interventions that effectively improve the health of Arab Qatari population. The goals of this research were to (1) understand the concept of physical activity as experienced by Arabs living in the State of Qatar; (2) identify the barriers and facilitators influencing the physical activity in Arabic adults; (3) recommend evidence-informed health promotion strategies that could contribute to healthy lifestyle thus lower the risk of chronic diseases and obesity. In this report, we discuss the findings in relation to the facilitator and barriers influencing physical activity in Arab adults in Qatar. We will recommend its implications for practice, health care, and policy. The ethical approvals were obtained from several ethics review boards which include: Hamad Medical Corporation/Weill Cornell Institutional Review Board, the University of Calgary’s Conjoint Health Research Ethics Board, Qatar Primary Health Care Research Committee, Qatar University, College of North Atlantic in Qatar, and Qatar Supreme Council of Health. No incentive was given to participants.

## Methods

### Theoretical framework

The Socioecological Model [34] was the overarching theoretical framework that guided this research, from literature review, questionnaire development for data collection, and data analysis to interpretations of the findings. The model has been widely used as a theoretical foundation to inform the Canadian health promotion framework such as the Ottawa Charter for Health promotion (Epp, 1986) [35], Epp’s Framework (1986) [35], and the Lalonde’s report (1974) [36]. Its philosophical underpinning emphasis the interaction between individuals and their physical and sociocultural environment; it helps understand how an individual’s health-related behaviors are influenced by various social determinants of health [37–40]. We chose this framework because it guides researchers to look beyond the individual level for influencing factors at different levels of an ecological system (i.e., interpersonal, intrapersonal, organizational, policy, and environmental levels). The model recognizes the interaction between influencing factors across the different levels of an eco-system; therefore, multi-level interventions are likely to be the most effective types of interventions to change behavior [37–39, 41]. To address physical inactivity among Arabic-speaking people in Qatar and to promote healthy lifestyle, we investigated with an open mind and curiosity to listen to participants regarding various factors and social determinants of health that affect their health and health care choices.

### Research design

To answer the research question—What are the facilitators and barriers influencing physical activity in Arab adults in Qatar?, we conducted an exploratory qualitative research using focus group interviews as the method of data collection. We asked focus group interview guiding questions such as: What comes to your mind when I say the word “Physical Activity”? Can you please tell me about things that make it easy for you to engage in physical activity and why? Can you please tell me about things that make it difficult for you to engage in physical activity and why? Can you please offer your ideas and suggestions about how to promote physical activity for Arabic adults living in Qatar? This study took place in Doha (Capital of Qatar), Al Wakra (South of Qatar), and Al Khor (North of Qatar).

#### Sampling and participants characteristics

The inclusion criteria for this study were: men and women who are (a) 18 years of age and older, (b) born and raised in Qatar, or had lived in Qatar for more than 5 years, (c) self-identified as Arabic-speaking man or woman, (d) willing to commit to an interview that lasts at least for 60 minutes, and (e) provided a written consent. We excluded Arab people who have lived in Qatar for less than five years because we assume it takes at least five years to adjust after relocating to Qatar’s very diverse and complex society, to gain awareness of the available supports and in-depth understanding of the contextual factors that influence physical activity and healthy diet practice in Qatar. To capture a wide range of perspectives from participants relating to the topic of physical activity, we used maximum variation purposive sampling technique, which is “the process of deliberately selecting a heterogeneous sample and observing commonalities in their experiences” [42]. The study participants were recruited from three universities and colleges and five primary health centers in Doha, Al Wakra, and Al Khor. These sites were chosen to ensure a representative sample of informants from an urban setting (Doha) and semi-urban/rural settings in both the north and south of Qatar. To ensure that the project is open to as many people as possible, all of the research information was available in both Arabic and English. Prior to conducting an interview, each participant was given an explanation of the study and opportunities to ask questions. They were assured that their participation was voluntary with rights to withdraw at any time without giving any explanation and that all information would remain confidential. Then the written consent was obtained before beginning the focus group interview.

#### Data collection

Focus groups are characterized by the explicit use of group interaction to develop new understanding and to explain previously unstudied phenomena [43, 44]. A strength of the focus group research is that it can produce concentrated data and collective voice in non-threatening environment that enhance participants to speak freely and build on each other’s ideas [43, 44]. The dynamics of the focus group help participants express their views in ways that are less likely to occur in an one-to-one interview [45]. The key investigators of this study trained interviewers who conducted the focus group interviews with participants. We created several different focus groups stratified by sex and age–male vs. female and age of 18–30 years vs. 31–50 years vs. 50+ years. We believe that participants would be more comfortable sharing their thoughts among people who share same gender, similar age group, life experiences, and interests [43, 44, 45]. Having separate men and women groups was important culturally given the social, cultural and religious context of Qatar. Each focus group lasted for about 60 to 75 minutes. All interviews were conducted in Arabic and audio taped with consent from all participants. An interview guide with open-ended questions was designed specifically for this study, i.e, “Can you please tell me about things that make it easy/difficult for you to engage in PA?” and “Can you please offer your ideas and suggestions about how to promote PA for Arab adults living in Qatar?” We conducted pilot testing of the questionnaires and focus group protocols with six focus groups. Only minor revisions were needed to the questions—some questions were revised to make it more clear to participants, and focus group protocols (e.g. location of the interview and number of research assistants to facilitate the interview session); hence, the data collected during pilot testing were analyzed along with the data from the rest of the interviews. When the data reached saturation, a point at which no new information was reported by the participants [42], data collection stopped. To ensure the rigor and credibility of the study, as well as to confirm the emerging themes, ideas, and concepts, researchers discussed the preliminary results with several participants in the second interviews as member check, using two focus groups of ten participants (a female group comprised of six participants and a male group of four participants). In total, 32 focus groups (6 pilot, 24 main, and 2 member checks) were conducted with 128 participants.

#### Data analysis

Data analysis occurred concurrently with data collection as an ongoing process [43, 44, 45]. The interview data were transcribed into English by our trained research assistants who are fluent in both Arabic and English. To ensure accuracy of the transcription, a trained research manager who did not transcribe the original transcripts checked the accuracy of the Arabic audio recordings and the English transcripts. The transcripts were then coded using a qualitative data software, NVivo version 9. Thematic analysis of the data was undertaken. The process involved a reading and rereading the transcripts, coding the concepts, and categorizing emerging themes. A coding tree with themes and subthemes was generated with each focus group interview and underwent refinement as more data were added to the coding tree. The analysis was conducted by the research project manager and research assistants under supervision of the lead principal investigator. The whole research team met for two hours each month, in person or by telephone conference, to discuss analysis outcomes and its process. Audit trail was established to ensure reliability and rigor of data analysis.

## Findings

An analysis of the focus group interview data resulted in identification of facilitators and barriers that can influence adult Qatari population’s choices and behaviors on their physical activity (PA). Using the socioecological model as a theoretical framework, the themes and subthemes are organized into three different levels—individual level, sociocultural level, and organizational and political level. At the individual level, perceived benefits of PA, presence of diseases, person’s will, motivation and goals, and time to exercise influenced the individual’s physical activity. At the social cultural level, religious teachings of Islam, cultural attitude and beliefs, and informal support influenced the participant’s physical activity. At the organizational and political level, physical environment to exercise, accessibility of facilities, organizational support, health information about PA influenced the participant’s physical activity. A quote coming from a female participant is indicated with (F) and male participant with (M). The age group is also indicated for each participant’s quote.

### Individual level

#### Perceived benefits of physical activity

All participants were well aware of the health benefits of PA. Reducing weight and preventing obesity and other chronic diseases were the most common reported facilitators for most participants to engage in PA. Some participants suggested that prolonged engagement in PA would not only prevent diseases, but also improve longevity and quality of life. For example, participants mentioned:

“To live a good, comfortable, and long life is the biggest motivation.” (M, 18–30)

“To maintain health. As I said before PA is considered as the first line of defense against diseases. You can also continue living in a good health condition.” (M, 31–50)

While health benefits of PA acted as a facilitator for some, the fear of getting diseases and injuries, such as heart diseases, diabetes and falls, as a consequence of not living active lifestyle was a facilitator for others to engage in PA. For example, participants stated:

“The fear of getting diabetes, because it is a widespread disease. One must try to avoid these diseases by doing sport. This is what encourages me to do sport.” (M, 18–30)

“I don’t want to be fat. I feel fear from people around me who have diseases.” (F, 31–50)

“I try to walk. If the person falls, especially the elderly, it is over. If the person stayed inactive, he will be sluggish and the whole world’s diseases will affect him. He has to walk, if he cannot do exercise, he should walk at least, walking is enough.” (F, 50+).

Improving or maintaining physical health, improving functioning and preventing diseases were communicated as important for all age groups. Maintaining an attractive or ideal body shape was important to both women and men participants, especially among the younger (age 18–30 or university students) participants.

Attaining an “ideal, appropriate, and nice” body shape was considered desirable perceived benefit of PA that acted as a facilitator for many participants in younger age groups. Mostly, female participants spoke about their desire to have the slimmer body by losing weight. Women participants said comments like “these days we focus on the body shape to be appropriate” (18–30) and “I want my body to be nice like them” (18–30). They would start exercising when they feel that they have gained weight to achieve an ideal body shape. Having an attractive body shape was important for the male participants as well. Several men in the younger age group spoke about perceived social pressure to have a fit, healthy looking body. According to some, looking good could be connected to social reputation among men university students. For example, men student participants mentioned:

“The social pressure upon man about his body to be in a certain shape pressures me to do sport. At least to have a fit and sporty body shape if not having the maximum fitness or full muscle shape.” (M, 18–30)

“To build up a better body and maintain the body’s health, better for your reputation … you will have a special [social] position.” (M, 18–30)

Aside from the physical benefits, improved mood and cognition was another benefit that facilitated participants to engage in PA. Several women participants in all age groups found that regular PA relaxed their mind and body, improved focus, reduced stress and fatigue, and elevated mood. For example, participants mentioned:

“When I exercise, I psychologically relax, and my mind becomes active, it helps me specially in studying. It benefits the mind. At once you would feel the difference if you did sport!” (F, 18–30)

“When I am stressed I do sports to vent my stress. By playing sports, I would relax.” (F, 31–50)

As well, some suggested that they feel energized after exercising, thus they would exercise when they feel “tired and apathy … to eliminate the tiredness from the body [and] to feel more active” (F, 31–50). From the focus group interview, we could discern that direct and indirect physical health benefits such as losing weight, preventing diseases, improving physical and mental health, and building the fit and attractive body were facilitators for PA.

#### Presence of disease(s)

The presence of diseases such as obesity, diabetes, hypertension, and hyperlipidemia was discussed as both a facilitator and a barrier to PA. Some participants were motivated to engage in PA because they wanted to lose weight and manage their chronic diseases with lifestyle modification. For example, some participants mentioned that they walk because they have high cholesterol and high blood glucose level. And another participant mentioned that their family’s lifestyle has changed with her husband’s illness.

“[My husband] was obese in the beginning. He did an operation to lose weight. Since that time everything at home started changing to be healthy, including our food. [My husband encourages me] that I should lose weight. When you are at the borderline to be diabetic, hypertension, you will push yourself to do.” (F, 31–50)

However, the presence of disease was a barrier to PA for some participants because the disease’s symptoms limited their ability to exercise even if they wanted to. Several participants with obesity and osteoarthritis mentioned that they could not be physically active because of severe knee pain. For example, a participant said:

“If the person is old and has pain, it is difficult for him to practice PA. For example, I am diabetic and if I have the ability to walk for 1–2 kilometers daily. I know that my blood sugar level will drop, no doubt. But in the evening, I might have knee pain because I walked in the morning.” (M, 31–50)

#### Person’s will, motivation, and goals

The majority of the participants believed that living a healthy lifestyle often comes from personal motivation. It is also based on how the person prioritizes health. Many participants valued individual responsibility for health and suggested having a strong will is a facilitator.

For example, a participant elaborated:

“The person’s will is what makes him to practice sport or not. It comes from himself…. I was 50 when I started doing sports and thank god there isn’t an obstacle. One should have the power and energy and the intention to start doing sports, go out, not necessary to do body building or weight lifting, go out walk, ride a bike, go to the corniche and walk for 2 hours, himself, there is no obstacle” (M, 18–30).

Similarly, some participants suggested having particular motivations and goals, such as preventing diseases and having an ideal body shape, facilitate them to commit to PA in the long term. For example, a participant said:

“If I don’t have the goal that I want to achieve, I will not go to any [health] club. For me, it is essential to have goals, to have the fitness, not only shape.” (M, 18–30)

Conversely, lack of will or motivation was discussed as one of the major personal barriers. Participants mentioned that everything is available for them to exercise, but they lack the will, motivation, and desire to engage in PA. Taking much time and effort to see its outcomes (i.e., weight loss) was discouraging for some. Many said that they felt lazy and tired to exercise after work or school; watching T.V. was an easier entertainment than going out and playing sports. Some regarded PA as a waste of time and something unimportant. For example, participants mentioned:

“I think the first obstacle is the will from the person himself. If the person is lazy and likes to eat and sleep or watch T.V. and doesn’t like to move, then it is an obstacle.” (M, 18–30)

“There are no barriers regarding the environment. But when you don’t have the desire, the initiative and joy, this will make you inactive.” (M, 18–30)

“Some people might think [PA] is a waste of time…. Men in our culture who are of high social status might think, ‘why should I waste time in doing exercise, I am an important person. I have the money and I don’t need to exercise.” (M, 18–30)

To overcome this personal barrier such as lack of will and feelings of lazy, participants recommended that it is important to find the motivation or goal, believe in the outcomes of PA, and allocate specific time to exercise. Participants recommended the following:

“If the motivational factors are not there, person should encourage himself and find the motivation. Find a certain time within the 24 hours to exercise. For me, the perfect time to exercise is after the dawn prayer.” (M, 50+)

“If we’re under pressure to study, we will face difficulties in doing sport. One must set a time, exercise according to the schedule, not according to the mood.” (M, 18–30)

#### Time to exercise

The lack of time to exercise was often discussed as a barrier to PA. Most participants perceived that they were too busy with work, study, and family obligations and responsibilities to “exercise and play sports” (Many participants perceived PA as exercising or playing sports). University student participants felt that their life was all about schooling—attending lectures all day and studying/working after school. Some university student participants mentioned that society’s pressure on men to be successful is very high; thus, they are usually occupied with studying and excelling in courses. Older age group participants, both women and men, mentioned that their work schedules, getting up early to go to work and returning home late at night, shift work, and heavy workload exhausted them to be physically active after work. Both women and men participants said that they have parental and familial responsibilities to attend after work, such as taking care of the family members and spending time with them at home. Participants explained the time barrier:

“The lifestyle as a student, we go to the university then we come home. We would either want to sleep or study. There is no time to play sport or to go out and do something. For me, there is only university and nothing else in my life at the moment. … In our society, the pressure on Arab men is huge in general. In addition to studying, the things he has to do in the home and in the society. Men have many pressures. The time is limited even for resting. So there is no time left to do any sport activities.” (M, 18–30)

"Some people work and don’t find time to practice sports, they work early in the morning and they finish late so they don’t find time … the physical laborers, their work makes them unable to find time to practice sports. Work is all our exercise.” (M, 31–50)

PA was particularly challenging for married women being the main caregiver of the children and family. They had domestic responsibilities such as food preparation, house chores, helping children with homework and studying, and taking care of their husbands and other extended family members. Furthermore, more women are now working outside the home than before in Qatar; working women found it was difficult to balance work, domestic responsibilities, and their health. For example, a woman participant mentioned:

“I have responsibilities, house and children. But I can devote some time to exercise. At night, I can exercise after I put [my daughter] to bed.” (F, 50+)

Nevertheless, many agreed that the lack of time should not be an excuse not to involve in PA because they believed that they could make time if they had a strong will and intention to exercise. Participant emphasized the importance of prioritizing health, managing time effectively, and devoting some time for PA to overcome this barrier of time. The following is the participants’ recommendations for themselves and others.

“You can find time like before going to bed or when you wake up. Maybe like my colleague said, use the break time. It is the matter of time management. If you managed your time effectively, you will find time for everything.” (M, 18–30)

“We don’t do our best, honestly. Everyone is busy with work, with children, with school, and with home responsibilities. The most important thing is to find time to exercise whether it is in the morning or in the night.” (M, 50+)

“Allocate time to exercise at least 30 min and walk 30 min around the house daily until it becomes a lifestyle or a habit to walk every morning.” (F, 18–30)

### Sociocultural level

#### Religious teachings of Islam

The majority of the participants discussed that being a Muslim and following Islamic religious teachings were facilitators to engaging in PA. Participants suggested that having faith in God helped them to prioritize their health and take personal responsibility of health. For them, adopting a healthy lifestyle meant not only advocating for their own health, but also living up to the values and teachings of Islam. Some participants argued that being religious equates being physically active because the physical movement that results from doing five prayers a day, including walking to the mosque for each prayer, help Muslims remain physically active. Therefore, some participants believed that praying was a way of being physically active. They said:

“My health is the most important priority. I walk as much as I can and taking care of my diet. I see my future in my health. Thanks to God who made me Muslim and gifted me with 5 prayers daily. Through the prayer I feel very active. I pray *Al-Fajor* (dawn prayer) on time. I walk to the mosque. *Al-Roqua’* and *AL-Sojoud* (prayer movement) all these movements and fulfilling the rituals of Pilgrimage, *Sa’ay and Tawaf* (walking fast back and forth). … Islam with all its rituals and worships calls for exercise. The prophet came for health. If we practiced our religion through timely prayers and walking to the mosque to pray, we will not need anything else.” (M, 31–50)

“Praying on time is the most important [PA] for me. The dawn Prayer, afternoon prayer and evening prayer… It is full exercise. If he went for prayer *Thuhr* and *Asr*, this is the most important thing to keep health.” (M, 50+)

“Medical research found that people who pray the dawn prayer has less stroke than people who do not. Why? Imagine your dawn prayer when you do it. They found the continuous sleeping for 8 hours causes stasis in the blood circulation.” (M, 31–50)

Furthermore, some participants discussed the parents’ roles in encouraging their children’s participation in PA by referring to scriptures from the Quran. For example, a woman participant referred to PA as an important part of child rearing:

“It’s the role of each mother to encourage their children not to sit for long hours watching TV, computers, or other electronic devices because they are harmful! Prophet said that it’s good to be active. There is a *hadith* [the traditions of the Prophet Mohammed] of Prophet Mohammed, ‘teach your sons swimming, archery and horse riding’ which encourage them for doing sport.” (F, 31–50)

Thus, Islamic teachings were a strong influencing factor for participants, especially among the older participants. Religion can be utilized in health promotion education.

#### Cultural attitude, beliefs, and practices

Sociocultural attitudes and beliefs towards ageing can be a barrier to PA. Several participants implied in their narratives that exercising or playing sports is for young people. Vigorous exercise was not suitable for them; walking was the most suitable exercise they perceived they could do. Exercising at an old age was not supported by the society and was viewed as a shameful act, especially for women. For example, a woman from an older age group mentioned that,

“The society's perception, ‘you are old now, why do you want to exercise?’ The Society doesn’t have an open view. When people see a 40 year old woman jogging or running on the street, people will say ‘see how silly she is running on the street. She became hysteric. She wants to be like youth.’ People would not say sports is for both young and old.” (F, 50+)

As reflected in above quote, cultural attitudes and beliefs toward the ageing population and gender can affect women’s health, especially elderly women’s health, differently than others. As participants mentioned, “Exercise in our culture is considered a shame for our women” (M, 18–30) and “Most of the sports activities and health centers are for young boys. There is only one place for girls, and it is in Doha” (F, 31–50). “When [a woman] wants to go outside, her husband will not allow her to walk alone. I can’t go alone for a walk” (F, 31–50). Women participants mentioned that some families and husbands are supportive of their women family members’ participation in PA. However, some families do not allow women to practice sports, go to the gym, or go for walk, unless they are accompanied by a male family member. A woman participant said:

“We are married, my husband doesn’t like me going to the sports club. Even for my daughters, same thing, their husbands don’t allow them. Not all men have the same mentality. Some would allow her to go, but some wouldn’t. My husband wouldn’t accept.” (F, 31–50)

However, older women participants in this study resisted this view and argued that it is their right to take care of their own health and it must be supported by the society.

“There are some people who would say ‘we passed this stage and exercise is for the young.’ Exercise is not for young people. This is for every person who has a live body. [Age doesn’t matter.] If I cannot exercise, then I should walk.” (F, 50+)

The participants voiced that they would like to see gym facilities for the elderly because they have different health needs and physical ability than other age groups. Furthermore, older people can encourage each other to exercise. The following quotes are their recommendations.

“The country can provide a club for the elderly so that they can get encouraged. For example, for the age 45 and above, with trainers and with small registration fees because not everyone can afford it. I and the sister (another woman participant) can go together and it will encourage us to exercise. You feel company when you are with people and learn from other people’s experiences.” (F, 50+)

“Provide us with places for exercise with a [knowledgeable] trainer, someone who can encourage, support, and teach us about exercise routines appropriate for age, health status, and diseases.” (F, 50+)

The lack of time to exercise described earlier can be interpreted as participants affording a lower priority related to physical activity engagement of health in relation to work duties, studying, family roles and taking care of others. For example, some younger participants suggested that their parents did not support their PA because studying, being successful, and attaining a higher social status through education and career were more important than being physically active or maintaining good health. They said:

“The most frustrating factor is the lack of support from family. [My parents] would say, ‘don’t go, it’s not necessary, don’t waste your time.’” (M, 18–30)

“The European’s community has the culture of taking care of self. But in Arab, we don’t have it. Once we return home from school, we find a dish of rice, eat, and sleep.” (M, 18–30)

“Some parents think exercise is a shame” (M, 18–30)

Some participants suggested that Arab people typically socialize by sharing food, inviting people over and sharing sweets or going to a cafe or restaurants. Rarely, people will socialize by playing sports or doing outdoor activities together. Perhaps this cultural practice might be affected by the desert climate.

“When someone invites me out, no one will tell me ‘let’s go out for a walk or let’s go to the sea or go to the Cornish. There are no places outside to hang out. So usually people will say ‘let’s go, I will invite you to a restaurant.’ Even for a change, we said we will go to the Aspire together. But suddenly there was the dining table full of goodies. There is always food before we leave and do something. I don’t know why.” (F, 18–30)

Participants hoped that the culture of socialization can shift from sharing food to doing activities together. As reflected in above quotes, some participants tried to improve their PA, but cultural beliefs, values, and practices can deter them from making the change.

#### Informal support

Informal support from family and friends, such as encouragements and accompaniment to the gym and exercising together, was a facilitator to PA for many participants. They suggested that they would be more active if their family and friends exercised together with them and if they had a role model, family or friends who are physically active. Participants from all age groups discussed the importance of having informal support; however, this factor seemed particularly crucial to the participants in the younger age group. For younger participants, the influence of peers and their presence was perceived as a critical factor. For example, participants said:

“If my family and friends practiced sport, I will have the motivation to practice more. I will try to give more or to be at same level they are or even more. … For young people, friends are the factor.” (M, 18–30)

“Encouragement is important. Last time, I made a competition with my friends to motivate each other. The person who lost most weight received a gift at the end.” (F, 18–30)

“The presence of a partner or someone who can encourage you will facilitate to do sport. Like ‘let’s go and play sport.’” (F, 18–30)

On the other hand, lack of encouragement and support from family and friends was perceived as a barrier by participants. Exercising alone and boredom deterred them from initiating or maintaining PA. For example, participants said:

“I stopped exercising after trying three or four times. I felt bored [to exercise alone]. I need encouragement. If someone does it with me, I will continue.” (F, 18–30)

“I need somebody to encourage me. If I do not have any friend with me in the club, it will discourage me to exercise. In the past, I bought a machine [like a treadmill] to exercise at home instead of going to the gym alone. But then I ended up not using it.” (F, 18–30)

Having good company who can motivate each other and having fun while exercising played a role in promoting PA for the participants.

In addition, some participants from the middle age group recommended parental support and guidance to encourage PA of their children. They perceived that they can play an important role in educating their children about the importance of PA and in supporting children’s engagement in sports and exercise groups. For example, a participant said:

“Family education and upbringing is an essential factor that contributes to PA. For example, when someone came from a culture where families are supportive of healthy lifestyle, the child will do exercise and eat healthy from childhood. It was difficult for us to practice sports because we did not have such supportive environment. But the children who had the support from his parents from childhood will be different. They will continue exercise for the rest of his life.” (M, 31–50)

Younger participants agreed that they would need their parents’ support to be physically active. For example, younger participants recommended to their parents:

“Parents should implant love of exercise in children. If a child says, ‘I want to play father’, then he should say yes and make the child feel that he is doing something right. So then the child will grow up with the love of sports.” (M, 31–50)

“The supports should come from mother and father. They should teach their kids about PA. No one is born having this knowledge.” (F, 31–50)

Throughout the interviews, it was evident that informal support from families and friends, having a good role model, and having parental support to be active physically were important to engage in PA and maintain healthy habit.

### Organizational and political level

#### Physical environments to exercise

Qatar’s natural environment, weather and the dust from the dessert, is not conducive to exercise outdoors. The temperature in summer months, from June to September, can reach 40°C or higher. Winter, spring, and autumn are relatively cooler with temperatures between 25°C and 30°C during the day and 15°C to 22°C at night. Because of the hot climate, the main mode of transportation is by car, as one participant narrated, “from home to car, from car to work, from work to car, and from car to back home” (F, 18–30), and there is no “lifestyle of walking or bicycling” (F, 18–30). Although some participants mentioned that the weather should not stop them from being physically active because they are used to live in the climate, most of the participants discussed weather as a barrier. For example, a participant said, “When the weather is hot, it is difficult to do exercise outside. With high temperature and humidity, how can I play sports outside? I cannot even walk under the sun” (M, 18–30). Availability of air-conditioned fitness centers with gym equipment, public places like parks and gardens, walkable streets with designated walking trails, and playgrounds were identified as a facilitator. Many participants suggested indoor, “suitable environments to exercise and play sports” (M, 18–30) was crucial in the summer.

“In Qatar, the heat makes [PA] impossible. I tried walking along the corniche (walk way along the waterfront) in the morning. It is better in the evening when the sun is down. I live in a place where there is no sports facility. These are things that would prevent me from doing sports.” (F, 18–30)

“In summer, it is hard to walk outside when you are totally wet [from sweating and humidity]. So if there are more sports facilities distributed [throughout the city], it will help me [to be physically active].” (M, 50+)

#### Accessibility of facilities

Many participants expressed their gratitude towards the government’s support in promoting a healthy lifestyle to its citizens. Participants suggested that places are available, but they are not always accessible because of distance, fees, gym policies on scheduling, and insufficient women-friendly facilities. “External factors, such as lack of spaces to exercise, could be a negative barrier for the person who wants to practice sports.” (M, 18–30)

According to the participants, there were lots of places to be physically active; however, the places they identified were often limited to a few popular public places in the capital Doha such as: the Aspire Academy, Katara Cultural Village, and the corniche. Some participants suggested that these places are great but far away from the residential areas, therefore, it is not convenient to access. Having to drive long distance and being stuck in heavy traffic were cited as barriers to some participants. They suggested that gyms that are near where they live would facilitate PA. For example, participants mentioned:

“If the sports facilities are close to your house, it would make it easy to reach them.” (M, 31–50)

“Increasing the number of sports centers in many places will facilitate PA. For example, we are living in Al Wakra and there is no single sports club. Each neighborhood should have a sports club. Otherwise all clubs you find them are far away in Doha. If there is a sport club, it will motivate people in the area to practice sports.” (F, 31–50)

Participants recommended that it is important to increase the number of gym facilities throughout the city and each village to improve geographic accessibility. The recommendations included building new gyms and community halls for exercise and activities, utilizing schools during summer when there are no classes, and utilizing shopping malls for walking.

“There are not many health clubs in the area. Each village must have a club to practices for free, so the people of this village would go and exercise. … The most important thing is to provide necessities such as playgrounds, sports clubs and fields.” (M, 50+)

“There are schools. All of these schools are air conditioned, and there are playgrounds. If these schools can be used as sports halls during summer, it will provide a place for more people.” (M, 50+)

“I was on vacation in another Gulf country, I saw women wearing training clothes in the mall. They walked around the mall. It is cheap and cost nothing and you can leave your children in a place to play or to leave them with the housemaid and you can walk around the mall. This will avoid the weather problem.” (F, 31–50)

High membership fee to join the gym or playground was a barrier to PA for some participants, especially university students. Although the Aspire–a public government funded sport facility was accessible at about 300–600 Qatari Riyal (QR) (approximately $82 –$164 USD) monthly, participants found it was too far from their homes. Private health clubs and the youth centers near their homes were usually very expensive (costing up to $3000 QR or $823 USD per month, according to the participants). Many university student participants reported that “not all students can afford to join the gym membership or buy gym equipment to use at home” (M). Some playgrounds reservations cost around 500–1000 QR hourly, students perceived it was not a reasonable price to spend on a leisure/physical activity. They said:

“It is better than before because the government increased the number of the playgrounds to have the world cup in Doha. However, the playgrounds reservation is very expensive, it is not a price that someone can afford… It kills the motivation. Even when there is a playground, money makes it difficult to register.” (M, 18–30)

“The problem is that other sports clubs are expensive. I cannot afford to exercise there. Aspire is affordable, costing about 300–600 QR monthly, but it is far.” (F, 18–30)

To reduce the financial accessibility barrier, they recommended that gym membership fees to be reasonable and to be free for people who cannot afford to pay. Some recommended there should be gyms that everyone can join for free. For example, participants said:

“My suggestion is to increase the number of sports clubs and to reduce the fees. Reasonable fees can be encouraging. Women wouldn’t go to the gym because the fees are expensive.” (F, 31–50)

“If all the cultural centers had a small gym room for free or with small fees, this will encourage us to be active. … I feel that the prices should promote health and should be reasonable for people.” (F, 31–50)

“There should be gyms that everyone can join for free and should not be expensive.” (F, 18–30)

Participants suggested that current public gym’s policies on scheduling were a barrier to accessing the gym. Gyms operated in a way that they give their members specific days and times to come to the gym, instead of members showing up whenever they want to. Participants mentioned that many activities such as swimming, football, and other trainings are available at the gym; however, it is not being utilized by many people because such scheduling is inconvenient. Moreover, some participants reported that the Aspire (the government funded sport facility located in the capital Doha) was difficult to access because it is usually fully booked. Some of them said they could not make a reservation because it was booked up to a few months ahead. Furthermore, playgrounds in the public area were operated with registration and could get over crowded; therefore, it was not always accessible. Participants emphasized the need to have gyms and playgrounds that are freely accessible without scheduling or waiting for months to access.

“Here the clubs specify the day that you go to do the training not the day you want to go to train. … Activities are available but unfortunately it is not used because they set schedules for people. How do you make appointments obligatory and then you tell me come and practice sports? I like to do sports, I like to do it every day, I have half an hour free, I will come. But if I am forced to come at a certain time, I can’t always make it. I might not be free on the day they want me to come.” (M, 31–50)

“The clubs at Aspire, it’s difficult to put your name on it. The reservation is very booked. My husband is little bit over weight. I [finally] persuaded him to join the gym after a long discussion. So he went to register at Aspire, but he was told to come back after three months to register. This means that the person needs to wait for months to start practicing sports? So he said I am not going to exercise.” (F, 18–30)

Most women participants suggested that there are not enough supportive environments for them to be physically active. Accessing the gym facilities was particularly challenging for female participants because of sociocultural beliefs and practices, such as public modesty and wearing *hijab* (a veil that covers the hair and chest) and *abaya* (a long-sleeved over-garment that covers the whole body except head, hands, and feet) in the presence of adult males outside the immediate family members. Being veiled limited movement and choice of PA. The issue of modesty and gender segregation were most important to women participants. All women participants preferred going to the women-only gym so that they could wear comfortable sportswear when exercising. For example, a participant explained:

“For example, if it is a mixed club for both men and women, it will make me feel lazy to go to the gym. I will say ‘oh, I should wear *abaya* and *hijab*.’ But if you are at the sport clubs for women, you will feel comfortable to exercise, and this will be a motive. They should offer some privacy for women.” (F, 18–30)

“For me if the gym is mixed, I will not go there to exercise. When I joined the university and found a gym for women, it encouraged me. We need to support and advertise women only sports clubs.” (F, 18–30)

Moreover, women with children could not leave their children alone in the home or could not bring them to the gym with them, so being a major caregiver for children and the lack of child care facilities deterred them from going to the gym.

“My children sometimes don’t allow me to go out. Children have to be with me. Where should I put them? I can’t put them in the walking area. There are many single men there. So I have to be with them in the house.” (F, 31–50)

“I have children, it’s my responsibility. How can I leave them alone and go for walk? I can take the older children, but what about the little one?” (F, 31–50)

Participants recommended that they would like to see more gym facilities for women, for example separate sports halls, parks, and swimming pools for women only, and gyms that have a children’s play area where they can leave their children while they work out. As well, women participants hoped that there were more events at the sociocultural level that promote activities and sports competitions for women to increase awareness of PA for women. Currently, participants perceived that there was insufficient awareness and space for PA for women.

“It is important to provide the appropriate environment like [culturally] appropriate gyms. We have Aspire for females, but we don’t have many places for females in Doha.” (F, 18–30)

“Provide ladies-only gardens and walking tracks. We can put our children to play and we can walk at the same time with the family.” (F, 31–50)

“Here they care more for men [than women]. In the youth centers, the man branch and woman branch are completely different. The woman branch only has meeting rooms. There is no gym. On the other hand, in the man branch, there is football field, tennis court, swimming pool, and more. If there is a place like that for ladies, it will motivate us to go and participate every day. If the weather is hot and we can’t go to gardens for walk, there should be youth centers available for us.” (F, 31–50)

Overall, we learned that physical environments that are inductive to PA are not always available or accessible to the population. It would be important to improve geographic, financial, and cultural accessibility of the available resources and to make environments user friendly by creating policies that are welcoming and accommodating.

### Organizational support

Having most of their day time occupied in school and work, many participants advocated for organizational support for PA in their schools and workplaces. “Environments [in which the person lives in], home, university, [and work] can motivate the person to exercise” (F, 18–30).

#### Workplaces that support PA

Participants, in the age group of 31–50, recommended workplaces that can encourage PA by providing a room with gym equipment that employees can use at any time and by providing breaks allotted for exercise during working hours. Some participants mentioned that part of their breaks can be spent on working out. They suggested that prolonged sitting and working on the computer have changed people’s lifestyle, making people physically inactive. They said the benefits of supporting employee’s PA would improve health and work performance by “lowering stress” and increasing “alertness, new thoughts, and refreshing mind”. Moreover, having a gym at work can also help people save time spent on driving to the gym. People can work out conveniently either before or after work. The following are participants’ recommendations.

“Here we have lots of work tasks and when back home we feel exhausted. I have seen on a TV program, in a Western country, they provide the gym at workplace to assist the staff to be active. I work at a company that has a branch in Dubai, the staff have free time to exercise in Dubai. They do that during their work hours.” (M, 31–50)

“If they make a small gym for the staff, this may help people and it will not cost a lot. A simple thing like treadmills, steps. After or before work, she can do it with her colleagues for half an hour. Starting work with some exercise might help people to activate their brain. These are simple things and I think it could be done.” (F, 31–50)

“The most important thing is providing the time, whether by a weekly day off or taking a period of time from the official working hours, for example half an hour out of the official 7 hours. They can offer a day off for you to do sports. It will not only provide PA for me but for my family as well. My wife wouldn’t go out except when I am there. So the day off will benefit me and those who are around me. For a long shift duty, I’d prefer that my work provides me a place to exercise during the break. (M, 31–50)

#### Schools that are supportive of PA

Several participants suggested that the education system that is not supportive of PA hinders the students’ participation in PA. Participants in both younger and older age groups suggested that physical education (PE) classes had been neglected and considered optional and that these classes could be replaced by science or mathematics classes which had more importance in the society. From the participants’ perspective, specific PE curriculum in the school is not adequate. For example, participants said:

“How will student develop love for sports if the sport class was not important? The issue is the lack of attention to the sport. … The sport class is taken by the other teachers. So this is the problem. … Even in the sport class, a teacher comes to the class and throws a ball to students and tells them ‘go play.’ After 20 or 30 minutes, he comes back and asks students to go back inside.” (M, 18–30)

“Our generation was not raised to love exercise or adopt healthy behaviours. Our health is in extreme threat. We should learn from our mistake and lead and motivate our children to love exercise so that the mistake will not be repeated in the next generations. Health depends on the environment in which the person was raised, like family. Usually physical education is considered secondary to other courses in Arab countries. Physical education class is only once a week.” (M, 31–50)

Participants believed that it is important to support school children’s PA through regular PE classes so that children, both girls and boys, can understand its importance early on and can make it part of their personal value and lifestyle. Participants recommended that strong emphasis be placed on PE classes by having it more often and making it a mandatory attendance. The following is their recommendations.

“Increase PA at schools, more PE classes, increase the importance of doing exercise at school. Offer PE classes 2 to 4 hours per week. Create after school exercise programs and tournaments. That would be the beginning. Children will grow with the awareness of PA. In the end, many more people will live active lifestyle.” (M, 18–30)

“What activities they provide to the boys, they should do the same for the girls, so they will be involved in that…[Educate] them and this will make a big difference … We must give the girls the sense of sport and make it a habit since childhood. When she grows up, she will not be shy. She will be happy to exercise and be familiar with it.” (F, 31–50)

According to participants, changes are already happening in some schools. The PE classes are given more serious attention, and schools stopped selling unhealthy snacks. These were seen as a facilitator by participants.

“In the school the sport class became very important. The boys are very keen about it and the teacher as well.” (F, 31–50)

“In the school they don’t sell candies, chips or Pepsi for children. They sell things with good nutrition values like cheese and fruits. My daughter told me that they sell fruits and vegetables in school.” (F, 31–50)

#### Information and awareness about PA

Participants suggested that the lack of health information and public health discourse were a barrier to engaging in PA. Many participants in this study, especially younger participants, perceived that sociocultural awareness of PA is insufficient in Qatar. For example, participants said,

“We didn’t know about the diseases related to lack of exercise until we are adults. For example, if the diabetic person had the knowledge of practicing sport earlier, it could have prevented diabetes. He wouldn’t have gotten diabetes.” (M, 50+)

“The problem is that many people don’t care and are not aware of the value of PA, especially some elderly people. They just sit in the house watching television and do not practice PA.” (M, 31–50)

“There is no encouragement for the young people like putting advertisement for them about importance of being physically active. There is lack of motivation and sport awareness. Make the sport likable.” (M, 18–30)

Participants recommended that it is important to raise awareness through various means, such as mass media, social media, public health education and campaigns, forming a volunteer group that can raise awareness of PA in the community, increasing the number of contests and competitions, and creating family-oriented activities so that all family members can participate. As well, participants recommended providing health information and knowledge to the public, i.e., the importance of PA and various ways to get involved in PA. Participants shared many great ideas; many of these ideas came from the university participants.

“For example, the advertisement ‘how did you look and how do you look now.’ This can be use in the gym. From day 1 in the gym, take a photo of yourself. After 2 months take another photo and compare the difference. And repeat after 3 months, 1 year, and 2 years. When people see changes over time, this will encourage them to continue what they have been doing. Similarly, we can advertise awareness campaigns on the T.V that show the difference between guys who play sports regularly and guys who live sedentary lifestyle.” (M, 18–30)

“We can use the social media to advertise the importance of PA. When we see pictures of other people with fit body, we will start to talk how do we reach the same level, achieve the body shape? People around me started exercising like that. Advertisement through social media can open the door.” (M, 18–30)

“We can encourage people through games. There was a competition that was done in *Katara* and there were lots of people there.” (M, 18–30)

“There are universities in Qatar that set up a day for a championship for all the sports. They give awards and academic recognitions that you receive it with the graduation certificate.” (M, 18–30)

“They could have activities that include all members of the family. If the mother likes to take her children, her husband would be there with them… they will be encouraged to join if there are such activities.” (F, 18–30)

“Make groups of people that would encourage each other such as volunteer groups. The girls can gather and form sport clubs for them. They can approach and encourage other girls.” (F, 18–30)

Some participants suggested that the support to increase PA knowledge and awareness of the public should be at the national level by placing greater emphasis on PA all year around, instead of one National Sports Day. They concurred that increasing awareness will assist more people live active lifestyle and reduce the treatment cost spent on NCDs. For example, participants said:

“On society level, [increase] awareness for those who don’t know. Provide more [ways] to motivate society such as the sport day and to make it regularly as well … One sports day per year is not enough; it has to be always there to encourage active lifestyle in general.” (M, 31–50)

“Make a long-term investment on PA, it will impact the treatment cost of diabetes and other diseases, and so it is cost efficient by all measures. If not, you will pay money on those who have diabetes, stroke, or other chronic diseases.” (M, 31–50)

#### Roles of the professionals

Many participants suggested that lack of information, knowledge, and direction was a barrier to PA. They appreciated the role of professional in sharing information and knowledge about PA and recommended that professional trainers be available at the gyms. Participants suggested that trainers can teach people how to use exercise equipments correctly, give beginning direction for starters, and provide encouragements. From participants’ perspectives:

“In western countries, they have a coach who motivates peoples, while here you are left alone with tools, without knowing how to use them. … Trainers can make workout easier and enjoyable for us. They can bring trainers who can show us exercises that are beneficial for us because there are lots of people who don’t know that.” (F, 18–30)

“There should be a trainer who gives you correct information in each club. When you are exercising alone, you might overdo or underdo it. If there is a trainer who can show you what is excess in your body, he can teach you how often and how much you need to exercise.” (M, 18–30)

“If there are trainers who would have education programs, not only for young but all age groups, [it would improve my participation in PA].” (M, 50+)

As well, several participants mentioned the important roles of health care professionals in providing information and knowledge about PA, recommending healthy lifestyle changes and disease management. Participants acknowledged:

“[Doctors] advised me to walk a lot to reduce the cholesterol, to avoid obesity and diseases. Thank God I started walking.” (F, 18–30)

“When the doctor informed me that I became obese, gained weight and I might have so and so [diseases], I started running.” (M, 31–50)

“The things that make it easy for me is the presence of someone who support me, presence of a person who is specialized to direct me exercise moves. Frankly speaking, the physiotherapy that I had at Qatar hospital made me feel better, move faster and more.” (F, 50+)

“In hospitals, doctors treat diseases with medications, meanwhile the most diseases like hypertension and diabetes can be treated by exercise. Hypertension can be treated by decreasing weight. The hospitals should add the exercise program in their treatment plan, not only medications, because most medications have side effects. So we should add an exercise program for patients with hypertension and diabetes.” (M, 18–30)

As described in above quotes, professionals like physical trainers, physiotherapists, doctors, and other health care practitioners can play a role in increasing knowledge and awareness of the Qatari adult population about PA. Overall, we could learn that the environment in which the person dwells—the culture, work, school, family, and formal support systems—all affect greatly the population’s physical activity and their health.

## Discussion and recommendations

In this article, we provide an understanding of various personal and environmental factors that might influence how adult Arab Qatari men and women participate in physical activity (PA). Unique to this study was the opportunity to explore the factors through focus group interviews, where participants could share their perspectives and build upon them to elicit collective narratives of a target group. Similar to the findings of previous studies conducted in the EMR [23–32], barriers to participants’ PA are: the presence of disease(s), feeling tired and pain, lack of willpower and motivation, lack of time and energy to exercise, low personal priority of health and competing demands, cultural norms and practices of public modesty (i.e., the need for women to wear traditional dress and to be accompanied by a male family member when going out), lack of social support, friends and companionship, low value of exercise such as insufficient health discourse in daily life, lack of affordable and accessible environments to exercise, lack of information about PA, and lack of supportive social milieu for PA especially for women. Congruent with the previous studies [25, 26, 31, 33], participants of this study suggested that the motivation to “fight” disease(s)/illnesses, perceived benefits of PA, having willpower and motivations to be fit, have fun and good health, being religious, valuing individual responsibility for health, having supportive social systems and affordable and accessible exercise facilities were facilitators to be physically active. The findings of this study add to the existing evidence that PA is a contextualized experience within the specific society, culture, and history.

Unique to this study was the findings specific to Qatar’s sociocultural context, in which many participants perceived that there is low value of PA in their work and school environments. Although some studies conducted with university students reported barriers existing at school [33, 46, 47], no studies have reported barriers existing at the work environment. We suggest that exploration of the research phenomena with various age groups at both university and primary health center yielded a broad range of perspective on PA. Participants’ recommendations about how workplaces and schools can be supportive of their PA are congruent with the WHO’s Healthy Workplaces [48] and Global School Health [49] initiatives that are guided by the Ottawa Charter for Health Promotion. Future interventions and policies can support health of the population by reducing physical and psychosocial risks, by promoting and supporting healthy behaviors, by implementing evidence-informed strategies that will enhance the health and well-being of individuals, families, community members, and by creating the healthy environment for living, learning, and working. Workplaces and school, along with hospitals and marketplaces, are the priority settings for health promotion [50]. In schools, health promotion strategies can include modifying curricula as well as school policies and environments. For example, administrators and educators can emphasize the importance of PA start at the elementary schools level, increase the amount of physical education (PE) classes by increasing the number and hours of PE classes and creating after-school programs, improve access to gender appropriate gyms, offer students a time management and PA counseling, improve teachers’ enthusiasm, and give recognition/awards to individuals who live a healthy lifestyle. Qatar Active Schools [51] is an exemplary community-based program that aims to enhance the health of children from early years. Finally, workplaces can modify organizational policies and environments to allow staff to work flexible hours that can accommodate PA, such as longer lunch breaks and other designated breaks for PA, encouraging the use of stairs, providing a place with equipment that staff can utilize during breaks, before or after work, and arranging a corporate challenge with prizes/incentives that involves PA.

From the interviews, we could see that cultural values, beliefs, and practices, such as the way people perceive older people’s PA, the way people practice their Islamic teachings, the way people socialize, as well as sociocultural value of exercise, influenced PA. Similar to Berger et al’s report [52], low value of exercise was not only prevalent among PE teachers at school, but also found within a social environment of family and friends. In this study, parental influence was an important factor influencing PA for some younger age participants (i.e., university students). This is perhaps partly due to Islamic teaching that emphasizes respect and adherence to parents’ wishes [53]. Some participants felt that their parents valued academic achievement and career success over PA or health. Some participants also reported that some parents might regard PA as a waste of time. Some male students felt pressured to be successful in society as men has been traditionally expected to be the breadwinner of the family; effort and time should be devoted to work on achieving that goal. Thus, there is no time for exercising. They emphasized the importance of parental support, guidance, education, and role-modeling in supporting their PA since childhood. Informal support from family and friends was particularly important to many participants. Lack of encouragement, peer support, and role-models were major barriers that repeatedly discussed by participants. Al-Kaabi [27] supported that possible way to improve compliance and continuity of PA would be involving family and friends. Qatar is a collectivist community [54]; therefore, people are influenced by other people who live in the community. This strength of the collectivist community can be capitalized in creating the sociocultural environment, where people encourage, support, and motivate each other’s PA and healthy lifestyle. Social support interventions, such as a peer mentoring and a buddy system, in community settings might be effective in increasing levels of PA in Qatar.

Sociocultural environment exerts great influence on the health behavior of the women participants of all age groups. Some cultural traditions and customs were obstacles to PA because women often felt that their PA was not supported by family members, especially husband. The need for female to be accompanied by a male family member when going outside, limits women’s PA options. Participants upheld the value of public modesty; keeping distant from men in the public setting, and the need to wear *abaya* and *hijab* hindered women participation in public gyms, sport clubs, or recreational centers of mixed gender as there are still limited facilities available exclusively to women. Female participants in this study suggested that PA is generally supported and encouraged for men counterparts, but there is no social structure that encourages women to be physically active. Berger et al. [52] explained that girls reach the age of puberty, when their breasts start to show, they are discouraged from exercise and practice public modesty and concealment of sexuality. The findings draw attention to the issue of health disparities that might exist among women. It would be difficult to empower women to overcome barriers to be physically active without challenging cultural beliefs about women and challenging how these beliefs are perpetuated in society. Changes to improve women’s PA need to be started at the political level. Health promotion strategies for women can include allocating appropriate funds to advocate women’s PA, building more culturally appropriate facilities like separate gyms and indoor swimming pools with women trainers, and incorporating a community-based approach such as involving community leaders to facilitate healthy trends such as a walking group. Shuval [29] reported that small groups of women doing power-walking helped gain public acceptance of women’s PA and in the Arab Israeli society. Increasing the population’s awareness of women’s PA, along with supportive social trends such as walking groups can lead to a change in sociocultural norms and might encourage behavioral change of women and men. Similarly, family-oriented events can be promoted and incorporated in the community to have all family members involved in PA.

Health promotion is defined as “the process of enabling people to increase control over and to improve their health” [35]. Application of the concept of health promotion requires actions on both risk behaviors of the person and the risk factors inherent in the environment of people. WHO suggested that the more health literate people are the more they are able to protect their health [55]. Therefore, strategies should include developing health promotion awareness and educational interventions. As participants recommended, information on PA should be actively distributed via different community-wide campaigns such as support groups, PA counseling with an exercise specialist, community events, mass media campaigns, and the creation of walking trails and parks in the community. Of the numerous interventions, the role played by mass media in influencing public behavior is widely recognized. National public health campaigns and social marketing campaigns can be created to raise the public awareness and knowledge about PA and to bring about behavioral change, particularly among youth and at-risk populations, i.e., who suffer from chronic diseases [50]. Health care and government can cooperate with broadcasting stations, social media experts, and health journalists to enhance health literacy.

Contrast to the findings of our review of the literature that family physicians were reported to be the most important source of formal support for adopting a healthy lifestyle [23, 27], the participants in this study seldom mentioned physician’s roles in assisting their PA. Rather, lack of trainers at the facilities was often discussed as a barrier. Thus having professional trainer assistance was recommended as an important formal support. We assume this difference in the findings might be because our participants were well aware of the importance of PA and were ready to make changes, but faced the barrier of lack of knowledge/past experience of how to exercise. It might be that physicians are engaging in promotion of healthy lifestyle, but behavioral changes do not occur due to other intersecting barriers such as lack of trainers, lack of affordable health clubs, and lack of informal support. We agree with participants that a trainer can be hired in each facility to help, support, and educate people about how to exercise appropriately, safely, and effectively.

Nonetheless, we argue that professional counseling and guidance, especially physicians’, regarding medical health, weight loss, and exercise can be a pivotal formal support to modify lifestyle behavior [25, 56]. As one participant recommended, physicians can play an important role in prescribing physical activity to individual clients because many NCDs can be improved and prevented with lifestyle changes. The prescription of PA should begin by determining the person’s PA preferences, activity patterns, social support, educational level, time constraints, and other challenges [27]. It is important to work with the individual and tailor PA counseling and intervention to the individual’s unique needs by mitigating barriers and leveraging strengths and resources of the person [27]. As well, physicians can tailor the PA prescription and set goals that are realistic, attainable and measurable with the client, this will facilitate follow up with the client in evaluating the process and effectiveness of the intervention. It would be important for physicians to collaborate with diabetic educators, physiotherapist, nurses, and other healthcare providers and utilize each other’s expertise and roles so that their effort to reinforce PA to their clients can be maximized.

An ecological perspective highlights a shared responsibility for health between the person and the environment. It is important that people live in environments conducive to health and healthy lifestyles. In Qatar, endorsing a healthy lifestyle through physical activity and healthy diet has received national recognition. In 2012, H.H Sheikh Tamim bin Hamad Al Thani, the then Deputy Emir and Heir Apparent, issued an Emiri Decree declaring the “National Sport’s day,” a national holiday on the second Tuesday in February. As well, various kinds of sports events are promoted through the Aspire Zone and the Katara Cultural Center. However, due to the rapid growth in the population along with the growth in wealth, with influx of immigrants and overseas workers, increasing the number of culturally appropriate and affordable recreational facilities throughout all regions of Qatar became essential in promoting PA. As many participants suggested, some part of Qatar did not have any sports facilities. Even in Doha (the capital city), sports facilities were not always easily accessible due to various accessibility barriers described in the findings. To the participants, adequate support meant accessibility such as building available, affordable, and appropriate sports and recreational facilities, changing operation policies of the gyms to make them user friendly, providing safe public spaces for exercise, creating sociocultural and organizational environments that people support each other’s health and healthy lifestyles. The social determinants of health framework can be emphasized to ensure health equity and create opportunities for health for all. Increasing access to culturally, financially and geographically accessible facilities should become a priority. Policy changes in allocating appropriate funds to promote PA and distributing resources equally throughout the State must be considered.

WHO stated in their ‘review of best practice in interventions to promote physical activity in developing countries’ [57] that PA interventions should comprise a combination of several intervention strategies, such as raising awareness on the importance and benefits of PA, creating local exercise programs, provisioning facilities for PA, and including various settings such as workplace, schools, health centers, and community. This aligns with the participants’ recommendations. Such large-scale interventions must be initiated at the national level with multiple points of entry to planning and implementation at the state and local levels [57]. In order to influence multiple determinants of health at different levels, a multidisciplinary and multisectoral approach, such as consulting the public, private sectors, and organizations and forming alliances across different sectors, such as health care, education, justice, government, social services, religious institutions, and community organizations will be critical. Moreover, dissemination of the interventions through appropriate technology and evaluation and monitoring of the interventions will be imperative to identify which strategies have been implemented effectively or ineffectively [57]. Finally, the State of Qatar National Physical Activity Guidelines [58] were developed in 2014 as a guiding reference to leading more physically active lives of the people living in Qatar. The guidelines offer information on the frequency, duration, intensity, and types of PA necessary for health not only for the healthy individuals of different age groups, but also for people who have chronic diseases such as CVDs, diabetes, and osteoarthritis. As well, the Qatar’s PA guidelines considered environmental factors such as hot weather and fasting during *Ramadan* (a month of religious fasting). This guideline should be actively promoted and utilize in PA education and practice.

The factors presented in this article as influencing female’s PA are not unique to Qatar or other Middle Eastern regions. In fact, many of these factors, such as lack of will, tiredness, lack of accessibility to available resources, lack of informal and formal support, have been shown to influence PA of people with other ethnocultural backgrounds worldwide. However, Arab adult populations’ experiences of PA is unique because they live in desert climate, a society with a strong religious orientation, gender and class hierarchical differences. In the milieu of rapid urbanization and Westernization, Qatar, similar to many other Middle Eastern countries, is in transition with changing societal structure, cultural, and social norms. Therefore, interventions addressing challenges of promoting PA in Qatar should reflect these transforming social changes and incorporate up-to-date evidence on what people view as important to them. Consulting the public for their recommendations on how to promote PA, through participatory approach as in this research, is essential in designing culturally acceptable interventions and to give them a sense of empowerment. Modernization and urbanization of Qatari society can pose a challenge because of differences in how body image and PA are defined and structured in Western societies versus Islamic societies. Interventions must consider social structure, culture, tradition, and history to be useful and effective.

### Limitations and recommendations for future research

The findings of this study cannot be generalized to other Arab populations because of the qualitative nature of the study design, such as sampling method and geographic/cultural differences. However, the findings are transferrable to the population of similar ethnic and cultural backgrounds worldwide as it offers some insights to the values, beliefs, and practices of healthy diet and physical activity of Arabic-speaking, Muslim population. A particular disadvantage of a focus group is the possibility that member might withhold personal opinions and be hesitant to share their honest thoughts for various reasons. Data collected from self-reported focus group interviews might be subject to recall or social-desirability response bias. According to Ganster and colleagues (1983), in an effort to conform to social norms, individual may give favorable responses which they perceive as acceptable by researchers and others regardless of their true feeling or actual behavior [59]. However, we tried to reduce this limitation as much as possible by training the facilitator to create unbiased, open atmosphere of the interview and by dividing groups into similar age groups and same gender. We recommend replication of the study to identify and fill in further gaps over time, to better understand the population’s beliefs, values, and their preferences and to identify opportunities for best health status possible. Our study participants’ age included 18+. Perhaps research study with school students younger than 18 might give another view of PA. Finally, one-on-one individual interview might help identify different values, beliefs, and knowledge that could not be openly expressed in a group setting. More research will be needed to understand fully the research phenomenon.

## Conclusion

Physical inactivity is a leading global risk to health by contributing to obesity. Many people are obese in Qatar. This is influenced by lifestyle changes that came along with the Westernization of the society. With the immense wealth from natural energy, the urbanization has influenced Qatari Arab population’s life such as the way people work, socialize, and entertain and their values, priorities, and beliefs. In Qatar, the prevalence and incidence of NCDs are high; people suffer from cancer and CVDs. The purpose of this study was to explore qualitatively the factors that influence how Qatari men and women engage in PA and ways to assist them to undertake the required amount of PA recommended by WHO. The findings of this study inform researchers, HCPs, and policy makers that Qatari population is well aware of the importance of PA, but their knowledge might not necessarily translate into action because of barriers at multiple levels. The individual’s willpower and motivation alone would be insufficient to adhere to recommended amount of PA in the face of numerous intersecting environmental barriers beyond the individual’s control. Understanding the facilitators and barriers to PA is important to tailor culturally appropriate interventions. The participants’ opinions and recommendations must be taken into consideration when planning culturally appropriate interventions and when planning care and treatment with the population. Multiple intervention programs will require multiple entry points across different levels and sectors; therefore, collaboration between healthcare, government, education, and community leaders would be critical.

## Ethics approval

The ethical approvals were obtained from several ethics review boards which include: Hamad Medical Corporation/Weill Cornell Institutional Review Board (IRB NO: 13–00051), the University of Calgary’s Conjoint Health Research Ethics Board (REB13-0347), Qatar Primary Health Care Research Committee (Reference NO: PHCC/RC/14/02/004), Qatar University (QU-IRB 244-E/13), College of North Atlantic in Qatar (CNAQ Approval NO: 2015–3), and Qatar Supreme Council of Health (SCH-A-UCQ-050).

## Supporting information

S1 File Arab adults’ perceptions of PA narrative data. (DOCX)
