# Supplementary material for: Arab female and male perceptions of factors facilitating and inhibiting their physical activity: Findings from a qualitative study in the Middle East
Source: PLoS One. 2018 Jul 16;13(7):e0199336. doi: 10.1371/journal.pone.0199336 (PMC6047779; doi:10.1371/journal.pone.0199336)
Supplement: S1 File — (DOCX) [file pone.0199336.s001.docx]

**Colour coding for Age Groups**

| **Blue** | **18-30** |
| --- | --- |
| **Green** | **31-50** |
| **Purple** | **50+** |

**Physical Activity**

- **Definition of Physical Activity**
- **Factors that make it difficult for you to engage in Physical Activity**
- **Factors that make it easy for you to engage in Physical Activity**
- **Ideas and Suggestions to Promote Physical Activity**
- **Definition of Physical Activity**

| **No.** | **Definition of Physical Activity (PA)** | | | **References** |
| --- | --- | --- | --- | --- |
| 1. **1.** | **Health** | | | **86** |
| 1. **2.** | **Types of sports** | | | **55** |
| 1. **5.** | **Movement** | | | **44** |
| 1. **3.** | **Personal belief** | 1. Age related (7) 2. Enjoyment (7) | | **35** |
| 1. **6.** | **Difference between PA and exercise** | | 1. Difference between PA and exercise- Different (15) 2. Difference between PA and exercise- Same (14) | **31** |
| 1. **4.** | **Relation of PA with food** | | | **10** |

1. **Health**

F1: it is that okay activate your mind and your body

F: anything that is sports and activates the body.

Male6: any sport activity which can help to circulate the circulatory system or re activate the …

Male 7: any physical activity that lead to build up the body and help it to be strong.

M4: It is burning energy, burning calories in the body through exercise or different things.

M5: Activity means movement, and as long as it is physical activity, physical means body and this can be anything like exercise or anything else not only exercises that nourishes the body, but also food, drink and most things.

F: healthy diet, it is everything related to sports, healthy diet and weight, that’s it

F: and to be aware not to increase amount of food to not gain weight.

F: that either burn calories or moves human blood circulation, physical activity not necessary helps to lose weight as long the person is active and his blood circulation moving not in the stage of Indolence or laziness that is the physical activity.

Female4: physical activity helps in thinking if it’s healthy food, its helps in thinking and cognition and capacity while studying, its help..., there will be activity and interaction that’s all.

F1: sports, and good activity

F1: everything is good.

F1: it’s for body, activity, is that right?

F2: energy, activity.

F2: doing effort which gets rid of toxic materials from body through sweating, person eliminates extra calories in order to prevent diseases such as diabetic or lethargy; it will renew vitality of brain and the body at some time.

F3: it is health and fitness for body, person get use to sport so body will became active and vital

F3: prevention for certain diseases

F1: it’s for health

M1: the physical activity means the health, exercise. It is about the exercise and about the health. It is person care about his health, sport, makes his blood circulation active, and makes the brain and body active.

M3: Physical activity is, is to take his medication, make it organized and looks after his health. Follow his medication, his life style, his exercise… if he does heavy duty job so he is practicing…, no laziness and no sleep, no…

F: you have the pushups for the stomach, and you have some exercises that can help make the joints stronger.

F: prefer it be one time at least per week although I don’t practice exercise unless I go to the physiotherapy. At the physiotherapy I practice exercise there. That is it.

F3: Walla, I do exercise in the hospital, I take the stairs and go down and I do exercise at home but the body is tired a bit. I also eat light food not much.

M: All replied at the same time: it’s so clear “it’s health and wellbeing”. [All laugh]

M2: well as am a diabetic person so sport makes me healthy. Sport is good for the body, stomach and for everything actually sport is… [M3: Interruption: maintain good blood circulation and movement is important]

M2: no it keeps look young and fit

M3: It will make you young and strong.

M3: fitness is the most important thing inside the human body. If the body is lacked of fitness them movement become hard during praying and muscles movement or any kind of body’s work. Body becomes stiffed but when the body moves the fitness will be. Fitness is flexibility of the body and when the body is flexible this lead to nerves and veins flexibility. Flexibility is everything in the body. Well flexibility of the body is very important for the athletic person or to the normal person. For the athletic the movement should be more but for the normal person as much as he moves it can be consider as activity for him.

M2: but the most important thing is to leave life’s problems behind.

M3: it doesn’t end. It is always after you, there is no use in that.

F2: physical activity might have many benefits from all aspects might be beneficial for health and might be enjoyable in the same time and is a good way of spending time and I recommend it.

F3: it’s beneficial for the body and the mind **a sound mind in a sound body** and the person would be relaxed and happy he would be comfortable.

F4: he would be more active and healthy also.

F5: what I know that it would make the person healthy and would feel his life being renewed every day that’s it.

M3: physical activity, the purpose of it is that one would control the excess storage of calories. In a very simple way; there shouldn’t be excess storage of calories in the body so that it wouldn’t be stored as fat after that there would be consequences on health for the fat storage, so physical activity any physical activity that is good for the person;

F1: health

F2: for sure health.

F3: health and energy,

F4: preserving body fitness and like that. That’s it.

M3: practicing sports generally I s the best thing for the human being, as physical activity

M4: Physical activity is important for the person’s health and you benefit from it every one would benefit from it, sports; volleyball, basketball, football, there is running, there is swimming

M1: the right body; the healthy body. It’s a method for production, renaissance and development. So when you ask about these things you ask about a healthy person that able to give and give a good tender

M2: it means health “A sound mind in a sound body”. If you eat properly that is all. Then you can function with other things.

M3: healthiness and activities

M4: the physical activity it’s when you can do things. If you were not active or sporty person you can’t do anything.

F4: exercise is something important for the health and the shape of the body all of and for many thing. exercise now has a high concept especially here in Qatar.

Female 4: What comes to mind thestrength , activity, and energy. And the health for sure. the fats move and the cholesterol move it will decrease from it little bit.

F1: sport, health

M1: Look, we don’t practice sport but we know that sport is health, exercise has benefits, but I’m not longer practicing

M2: What comes to my mind, exercise is good there is no doubt, for the health of body, it’s good for everything in the body, exercise is its essential ,it is important for the person to exercise so his health is good.

M3: exercise is a training for the body, makes body soft and flexible and its prevents the causes of strain, it’s like swimming, walking, running volleyball, there is a lot type of sports,

M: any one practices exercise doses not need a doctor, medication or anything else,

M:make specific time to practice exercise for a month, if he continue for month his spirits will be good, disease will go and will be fully healthy.

Female 4: It means I have to workout to maintain my health and not to gain weightand to maintain my energy and to protect myself from the disease.

Female 1: exercise or physical activity it means health. It means that I am enjoying with body and good health. I don’t have cholesterol or hypertension. Well I had cholesterol couples of years ago despite that my weight was less. I was 70 kg or 65 kg I did not reach the 70 kgalthough of that I had a high percentage of cholesterol. It’s not necessary to be fat to have cholesterol. Also the sugar appears high in some periods of time. I am not that fat to have high sugar. I found that I have to stop here. I am doing something wrong; I had to make a stop. I feel that am still in the beginning of life and am not too old. These things occur to the people when they are aging.

Female 5: The human has toexercise this is very important. Healthy eating is very important, very important. It is right the sound mind in sound body. The health on the top of the head of the healthier no one see her.God decide when the human got sick he does not feel the value what you have or health until he found himself tired and found another human enjoying the vitality and energy and going back and force Mash-Allah. I remembered when I was pregnant and I can’t move the doctor told me not to move because I had bleeding, and in the same time high cholesterol and sugar. The bleeding does not need to move and the sugar and the cholesterol need movement to lower its level in blood... I used to see when I was following Mash-Allah no power than the God Mash-Allahno power than the God people you can tell that they are older in age and running. There is one lady she was pregnant may in her 8 months and running Mash-Allah. I felt that am younger than her in age and I can’t. I suppose to move so that I can reduce the sugar and reduce the cholesterol. In the same time I don’t eat much and the physiological state increased this.

Female 1: Activity

Female 2: Health.

Female 4: Health and movement

Female 5: Better life and health.

Female 6: Agility.

Male 1: the person must be healthy and exercise and keeps his own body healthy

Male 2: excuse me what is the question?

Male 2: practicing activity always makes you healthy and teaches you how to eat in healthy way. And when exercise in a proper way it makes you follows a good diet in a proper way too. It protects your body circulation in your body also makes you. Always it makes you active and that is it.

Male 1: for me the two of them are the same. If am I going to play sport intuitively I’ll make a physical activity both of them are the ingredients of life.

Male 1: there are no diseases at all

M: Health

Male 2: exercise is power

Male 2: or food consumption so there will be no harm to the body.

M: it’s eating healthy food

M: sleeping healthy and

M: It is also good for all body parts including the muscles, hearts, blood sugar or anything.

Male 2: For sure the most helpful activities for human body and life are practicing physical activities and movement.

M: It helps a lot in reducing the weight, for burn fats,

M:Physical activity is excellent. It is also good for burn body fats.

Male 4: They are linked to each other because spots improve the blood circulation that helps in digesting the foods, burn fats and sugar, make the body energetic, and improve the body function. It is also helpful to increase fluids intake especially during summer.

1. **Type of Sports**

M1: this is movement practicing some games, walking, running, these stuff, these are practicing sports.

M2: it is walking

M2: yes walking yes

F3: sports machines or walking, running

F5: it doesn’t have to only be sports specifically it can be football or basketball or any physical activity mean any movement!

F5: it doesn’t have to be a specific exercise.

F1: gym, walking, any type of physical activity

F2: almost same, sport, jogging, various sports not necessary specific thing

F: simplest like walking; it can be other type of cardio, aerobic, yoga, all sports.

Male 1: sport, practicing sport for example

Male2: gym, practicing sport, playing football, walking, everything let the body moves

male3: swimming, volleyball, practicing sport in general.

Male4: any practical activity that the body does in each different sport

Male 5: doing any physical activity produces energy and utilizes this energy in part of any activities and so

Male6: any sport activity which can help to circulate the circulatory system or re activate the …

Male 7: any physical activity that lead to build up the body and help it to be strong.

M1: Physical activity, in general it will be exercise, exercise with all types.

M2: The same time, the exercise…

M2: Something with activity to the body like running like that and swimming like that.

M: there are a lot of sports that moves the body, that is physical activity.

M2:Ahhhh, the ability to play anything, to move….. muscle and use it in the correct place

M4: as other youth says, the body moves to do a particular effort, to move muscle, fat, the body supposed to move it created for that. That is physical activity such as sport, walk, jogging, effort

Female1: physical activity means practicing sports,

Female2: physical activity is practicing sports, different type of sport not only one type, to maintain your fitness and to be aware not to increase amount of food to not gain weight

F1: sports, and good activity

F1: everything is good,

F1: it’s for body, activity, is that right?

M:On the other side we have, I am talking about Sudanese community; we consider the sport on football. This is what we have in the neighborhood even the Sudanese woman doesn’t know the concept of the exercise and physical activity and its importance to the body. This is regarding the girls

Female1: the sport that the person do daily either walk,

F: playing sport, going to the gym or swim and for how long for example. Does he walk for 10 minutes or half an hour. Some people do that more and some do it less. It is the activities that the person does it in daily basis.

Female2: the movement and the walk, more walking in the kitchen or in the street or in any place.

Female3: it it the practicing sport, or the activity that we do every day either house works or sport outside. That is it.

Female 1: Practicing sport could be something you do it every day, for example you go to the gym every day, using the trait mills every day.

F: running, walking for a long distance,

Male 1: what I think about it?

Male 1: running, walking. In Qatar it is walking

Male 2: walking and running, the same, I swear

M1: it is the simple sports like walking, running, activity at the gym, this is it

M2: same thing; it is walking, sports, football, sometimes ------

M2: the hobbies I have; football, walking, anything that has movement ---- and that’s it

M: walking

M: machine or going to the gym, swimming and the activity differs according to how many calories you spend in it. Each activity is different than the other, for example; swimming is the best kind of activity because in one minute you burn more calories than the calories you burn in one minute of walking, and there is something called wobbling activity; the person can… but in this the person should be an athletic person so that he wouldn’t collapse; that he would walk the highest level for one minute and then walk the lowest for one minute. And after than the highest for one minute and then the lowest for one minute, all for 15 minutes would make is as if he walked for an hour, because he is casing a wobble in the heart beat so blood would move in the arteries faster and this wouldn’t let there be any sediments of cholesterol in the arteries and any sport activity the most important thing Is that the person does any sport activity that is available

F1: in my opinion the physical activity is mostly is walking. You have to walk. Don’t sit for a long time.

F1: swimming for example. It’s one of the most beautiful things or walking.

F2: running.

F1: no, running is not suitable for me. May be the walking and the swimming is the best thing.

F3: walking is the best.

M2: my understanding of practicing sport of course is that it’s something like walking for half an hour

M4: Physical activity is important for the person’s health and you benefit from it every one would benefit from it, sports; volleyball, basketball, football, there is running, there is swimming

M4: horse riding

M5: it’s every effort one would make in his walking in his work, sometime he would make more effort in his work than a physical fitness effort

M1: Sport, football, walking,

M1: volleyball,

M2: football, table tennis, walking,

M2: jogging, that’s all

M3: all exercise in general,

M3: practicing sport regular at morning or evening, practicing jogging, football, any sports during day and evening that makes body active

M4: yes, physical activity is the exercise and the physical effort of work, for me personally I start with physical effort of work, physical activity is weak according to me [laughing].

F2: sport

F3: practicing sport

F2: practicing sport

F4: practicing sport

M3: exercise is a training for the body, makes body soft and flexible and its prevents the causes of strain, it’s like swimming, walking, running volleyball, there is a lot type of sports,

Female 1: walking and doing exercise.

Female 1: walking and doing physical activity.

Female 3: sometimes I feel I want to walk but walking for an hour and not to eat anything for 1:30 hours after the exercise and then maybe I will eat light meal.

M: play football, we play rugby;

Male 4: for me it’s almost the same thing but the physical activity comes from the sport; if you didn’t play sport you basically won’t have physical activity. Not the physical activity and then sport. When you play sport, first time you will have fatigue in the body you’re not going to say I won’t do it and so on I have no energy. When you play sport you will get the activity by itself. Every time you woke up and you know that you’re going to the GYM and you’re going to play sport so for sure you will more active.

Male 1: basketball.

Male 2: Jogging, the jogging.

Male 3: walking, all kind of sports games.

Male 4: anything that has movement and practicing sports.

Male 5: what comes to my mind is the jogging, the jogging that I do daily. Come to my mind also the football that I do often weekly. That is practicing sports.

Male 5: for me I think the sport, the sport is to be practicing. For example I jog every two or three days in a regular way for me I consider this as sport. The activity is like simple movement getting up or going here and there this is by nature you do it this is an activity, but practicing is to be continuity.

Male 3: Jogging

Male 1: Physical activity is good in all types even if it is swimming or walking.

Male 4: for youth; there are many sports for youths such as swimming, walking and football, but for us as elderly people above 60 the only sport we can practice is walking.

1. **Movement**

M1: it is movement, activity

M1: this is movement practicing some games, walking, running, these stuff, these are practicing sports.

F1: for me it mean sports, movement, walking anything that makes the body move that it doesn’t stay still or lazy.

F: it could be stretches early morning or anything else.

F3: anything with body movement that benefit the body as all kind of exercise, gym that’s it.

M: everything let the body moves

Male4: any practical activity that the body does in each different sport

Male 5: doing any physical activity produces energy and utilizes this energy in part of any activities and so

M3: anything that has movement, movement of the person and requires effort is physical activity.

M5: Activity means movement, and as long as it is physical activity, physical means body and this can be anything like exercise or anything else not only exercises that nourishes the body, but also food, drink and most things.

M1: to move the body or the body is in a state movement such as Sports, there are a lot of sports that moves the body, that is physical activity.

M2:Ahhhh, the ability to play anything, to move….. muscle and use it in the correct place

M4: as other youth says, the body moves to do a particular effort, to move muscle, fat, the body supposed to move it created for that. That is physical activity such as sport, walk, jogging, effort

Female1: physical activity means practicing sports,

Female3: any physical movement

M: if he does heavy duty job so he is practicing…, no laziness and no sleep, no…

Female1: the sport that the person do daily either walk, work in the house, playing sport, going to the gym or swim and for how long for example. Does he walk for 10 minutes or half an hour. Some people do that more and some do it less. It is the activities that the person does it in daily basis.

Female2: the movement and the walk, more walking in the kitchen or in the street or in any place.

Female3: it it the practicing sport, or the activity that we do every day either house works or sport outside. That is it.

Female 1: Practicing sport could be something you do it every day, for example you go to the gym every day, using the trait mills every day. The physical activity for example it is something you don’t do it every day, like house work, using vacuum in the house, climbing stairs, things not in daily basis.

Female2: I fell it is the same thing, they are the same.

Female3: doing sport required daily rhythm, every day, but the physical activity is something mandatory to do it every day. Even for going to the work or house work etc. I think this is the difference.

F1: Playing exercise is practicing exercise, running, walking for a long distance, you have the pushups for the stomach, and you have some exercises that can help make the joints stronger.

F2: Physical activity is exercise, is to practice exercise in our life and to be, I prefer it be one time at least per week although I don’t practice exercise unless I go to the physiotherapy. At the physiotherapy I practice exercise there. That is it.

F4: For me, I can practice exercise all the day, if I have some duties for home, if I have an appointment at the hospital, I would take advantage of that use the stairs instead of the elevator. So I try to take advantage of moving, and moving all day is physical activity and I prefer walking daily if there is the chance.

M: become hard during praying and muscles movement or any kind of body’s work.

M: Flexibility is everything in the body. Well flexibility of the body is very important for the athletic person or to the normal person. For the athletic the movement should be more but for the normal person as much as he moves it can be consider as activity for him.

M: anything that has movement ---- and that’s it

F1: it is exercise or…

F1: the person moves.

F2: its body movement and walking.

F3: walking

F2: Walking which is much better than sitting down. It’s good for blood circulation also the muscles move, walking is better.

F3: Even working at home is movement.

F: But the preferred be active and active. Something that moves the body well and keeps your heart well.

Female 1: Exercise when I was young in my 30th and 35 I used to do rope skipping every day and playing sport and I never gained. Now I can’t walk from here to here. Right now I am treated in the physiotherapy I stand and move my hand this is exercise.

Female 1: this movement of my hand is exercise this is I can do it. I go out in the morning or after five to water my plant I consider it exercise. The movement of my finger I consider it exercises.

Female 1: I love movement and exercise. I am farmer. My family implants plants 5, 10 kilo the long of the plants. They harvest the plants and they collect the wheat which is longest than the roof. My family is farmers they have the cows, horses, camels, and the land which can walk on it the horses. My family is farmers what should I say more about exercises.

Female 2: exercise I don’t go to do it.

Female 2: You walk and go it exercises.

Female 1: Walking is exercise.

Female 3: Just walking.

Female 1: walking and doing exercise.

Female 1: walking and doing physical activity.

Female 1: The exercise then exercise then exercise.

Female 1: Activity

Female 3: Movement.

Female 4: Health and movement

Female 5: Better life and health.

Female 6: Agility.

F: movement, exercise,

M: exercise

Male 4: anything that has movement and practicing sports.

Male 6: the movement once woke up. Even at sleep time itself. The moment he drives the car or come down from the stairs or go to office or to work all these kind of movement is considered as sport in general.

Male 7: well for me it’s any movement in general but outside the office. As I am a student I spend most of the time in the office so anything else for me is considered sport.

Male 1: exercise

Male 2: Movement

Male 2: For sure the most helpful activities for human body and life are practicing physical activities and movement.

1. **Personal Beliefs**

M: but sports [laughing with M2] 50 years it is hard now,

F4: physical activity for me is the daily work I do and I can add to it daily exercise like walking for 30 minutes. That’s it.

F4: it’s a lifestyle, how person start his day physically, it’s something that the person is used to do, not for the first time.

M3: physical activity its vary according to age, there is difference according to age , if the person at early 20ths, physical activity will be more active while at end of 20ths its will be tiring on the person himself.

M2: Physical activity it is mostly the exercise dosage that the person take on the ordinary days or weekly and that is according to his functional (work) performance. Some people have the chance daily, some people are busy with the work and they don’t have chances except intermittent periods of time. In general this is our understanding to the physical activity which is the exercising time that you allot for yourself.

M: You come with no time available for exercise, our exercise is work. That is it

M1: this is exercise also (laugh)

M2: if you are anxious and worried you can go for a walk and you will find that the worries decreased.

Side talking by M3: that is correct, the air opens up the chest. (Makes it happy).

Male 3: tiredness

Male 4: I agree, the same, tiredness

Male 4: I don’t know, when someone hears the word (sport) he feels lazy, feels lazy to go out to practice sports or anything. He stays at home and that is it, or to go with his car

F 1: physical activity for me is for me something I can’t do or commit to it.

F: might be enjoyable in the same time

M1: it is the same thing but each person according to his own needs, excuse me there is one person that is obese; he would do sports more

It is different according to the age

M1: sport is very good and the physical activity and I am talking about myself it is very good thank god for me and there is sports I do practice sport

M2: When I do it I feel that I got tired.

F1: fatigue [laughing] tiredness

F3: what is the definition of sport! [Laugh] Well I feel like its fun; what can I say; nothing it’s just needs willing

F3: It needs continuity.

F1: We continue for some time and then not, although the machines are available at home.

F4: no one encourages you!

F3: for me I think it willing.

F3: yes willing,

M: its depends what suits you

M4: Exercise is happiness; it is life, it is everything.

M1: Diseases have nothing to do about that by the way. Diseases never go, if you became sick, you will stay on medication, how many years I have been taking medication. It never goes.

M1: hypertension, diabetes doesn’t work when you do exercise; you have to take your dose, are you going to cure it when we do exercise? It will not work, whether you do exercise or not.

M: you have walking, you have exercise healthy diet but the treatment should not be stopped.

M2: So he said walking, treatment and regulating your food. So this includes walking which is the exercise that we have to do.

M4: the smile, when you smile, it is itself an exercise,

Female 2: nice feeling.

Female 3: sometimes I feel I want to walk but walking for an hour and not to eat anything for 1:30 hours after the exercise and then maybe I will eat light meal.

Male 3: it means to me; the person change his life from negativity to positivity through practicing sport daily and when the person walks in a good way, his life is going to be well too.

Male 4: honestly when I hear the word exercise s I get excited because I am an athletic person and I practice sport so I always love the word exercise

M: organizing

M: Fun

Male 4: for youth; there are many sports for youths such as swimming, walking and football, but for us as elderly people above 60 the only sport we can practice is walking.

1. **Enjoyment**

F: might be enjoyable in the same time

F3: what is the definition of sport! [Laugh] Well I feel like its fun;

M4: Exercise is happiness; it is life, it is everything.

M4: the smile, when you smile, it is itself an exercise,

Female 2: nice feeling.

M: organizing

M:Fun

1. **Age Related**

M: but sports [laughing with M2] 50 years it is hard now,

M3: physical activity its vary according to age, there is difference according to age , if the person at early 20ths, physical activity will be more active while at end of 20ths its will be tiring on the person himself.

M1: it is the same thing but each person according to his own needs, excuse me there is one person that is obese; he would do sports more

M: It is different according to the age

M: its depends what suits you

Male 4: for youth; there are many sports for youths such as swimming, walking and football, but for us as elderly people above 60 the only sport we can practice is walking.

1. **Difference between PA and Exercise**

Female 1: Practicing sport could be something you do it every day, for example you go to the gym every day, using the trait mills every day. The physical activity for example it is something you don’t do it every day, like house work, using vacuum in the house, climbing stairs, things not in daily basis.

Female2: I fell it is the same thing, they are the same.

Female3: doing sport required daily rhythm, every day, but the physical activity is something mandatory to do it every day. Even for going to the work or house work etc. I think this is the difference.

F1: The definition is that some take the physical activity as the activity of the body from inside; they don’t take it that it is the definition of exercise. We, the generation of elder women, are weak a bit in thinking; I will not say that we are expert as today’s generation. When you ask physical activity, she will tell you what is physical activity, maybe it is something inside myself and I don’t know about it? This is the thought that I have. I have something that I don’t know about.

F2: Physical activity can be homework, I work home, clean the rooms, go up and down the stairs, and move, this is physical activity. For exercise, I become committed to rules, strengthen my muscles, arms and legs, carry weights, walk on a treadmill that will strengthen my body, this is active exercise.

F3: I am the same, I work at home and do exercise and I feel that I became better in movement and like that, which also digest better the food, at night I go and do things so the food will be digested. I do things bit by bit.

F4: Physical activity is your normal routine, your normal routine from when you wake up till you go back to sleep at night. How did you spend it, did you move or stayed sitting or all sleeping. At *Thuhur (12:00pm)* you have a break but some would make it long around 5 hours, so how you will spend it, but the active exercise is not, you spend certain timing for growing a certain muscle or make a certain area in our body slimmer or we can say to increase your activeness and daily movement. This is exercise.

M1: it is the same thing but each person according to his own needs, excuse me there is one person that is obese; he would do sports more

M2: it is sports ------ but physical activity it must be practicing sports physical activity and practicing sports or work, so these would be different for me if you said physical activity is sports I would say okay, moving physical activity might be at work; this is physical activity

M3: sports; if he is specialized in one sport in particular, if he is basketball player, if he is football player so he is committed certain kind of training, but physical activity in his work, he could move today in his work more than yesterday and tomorrow he might move more than… and one day he might not move at all. One could create his own physical activity, for example I can’t stay setting all day at work, setting on the chair, I can’t stay setting, I should every half an hour, hour to go out move about an hour and come back. This is called -------. One should create activity in his work, park your car far from the parking, instead of the elevator use the stairs this is called physical activity. But doing sports, it’s about liking a certain sport for example; specialized in sports or plays a certain sport so this is considered his daily work, which is sports, so it is a job so this is his daily work and he has to do it, but the physical activity its when one creates physical activity for himself.

Female 1: the same thing

Female 2: different

Female 3: the same thing

Female 4: physical activity and exercise

Interviewer: Yes are they the same?

Female 5: Yes the same thing.

Interviewer: you said they are different?

Female 6: I feel some times I do exercise but I don’t consider myself much active so I feel they are different.

Female 2: the same.

Interviewer 2: ok one last thing I would like to ask. The work sport and the physical activity is the same thing for you?

Male 1: it might mean a move in the body it may be another thing..

Male 2: there is a difference, the physical activity is on the north side and the sport is in the west side. The physical activity is like your body being active, how is your lifestyle, anything that has activity but sport is you practice sports a physical activity.

M: it’s not a different word it’s just almost this related to each other. When you play sport you will be active, energetic and so on.

M: for me the two of them are the same. If am I going to play sport intuitively I’ll make a physical activity both of them are the ingredients of life.

Male 1: yes it’s almost the same because if you’re doing sport you’re doing physical activity. They are associated with each other.

Male 2: yes they are close to each other. In my opinion they are the same thing approximately.

Male 3: the sport and the physical activity are might be different; the physical activity maybe is a movement; I do exercise this is physical activity. But the sport is playing basketball, football, jogging or walking. This is sport.

Male 4: I think they are associated with each other because any sport has a physical activity.

Male 5: for me I think the sport, the sport is to be practicing. For example I jog every two or three days in a regular way for me I consider this as sport. The activity is like simple movement getting up or going here and there this is by nature you do it this is an activity, but practicing is to be continuity.

Male 6: they are exactly having the same meaning, but when they are a specialty in certain kind of game or sport where there is more commitment in certain periods more than 3 days per week or more than 3 days weekly it turns to be specialty and then the type of sport is determined. According to it the body gets to use on it and becomes accustomed to, but all other kind of sport any movement you do is consider to be sport.

Male 7: they said it all. I won’t add more...Well same idea.

Interviewer: Are you saying they are one thing?

Male 7: yes, but it differ in the outcome of each, but at the end they are the same.

Male 1: Physical activity is good in all types even if it is swimming or walking. It is also good for all body parts including the muscles, hearts, blood sugar or anything. Physical activity is very good there is no doubt, we know that.

Male 2: For sure the most helpful activities for human body and life are practicing physical activities and movement. It helps a lot in reducing the weight, for burn fats, and protects human body from different kinds of diseases. It consider as the first line of defense against diseases at this time. Peoples are moving away from physical activities, but it is really very important.

Male 3: Also physical activity is helpful for the body, for blood sugar, blood pressure, and liver. Physical activity is excellent. It is also good for burn body fats.

Male 4: for youth; there are many sports for youths such as swimming, walking and football, but for us as elderly people above 60 the only sport we can practice is walking. It is better to have a program to walk for an hour in the morning and evening according to your capabilities. For me, I am over 60 and I walk daily at Aspire, but my sons and daughters who are married their weight is increasing because they do not like and practice physical activity. It is better to practice sports and eat healthy food, this is very important. At night, he shouldn’t eat heavy food and then sleep, the triglycerides in the body.

1. **Different**

Female 1: Practicing sport could be something you do it every day, for example you go to the gym every day, using the trait mills every day. The physical activity for example it is something you don’t do it every day, like house work, using vacuum in the house, climbing stairs, things not in daily basis.

Female3: doing sport required daily rhythm, every day, but the physical activity is something mandatory to do it every day. Even for going to the work or house work etc. I think this is the difference.

F2: Physical activity can be homework, I work home, clean the rooms, go up and down the stairs, and move, this is physical activity. For exercise, I become committed to rules, strengthen my muscles, arms and legs, carry weights, walk on a treadmill that will strengthen my body, this is active exercise.

Interviewer1: Ok and you,

F3: I am the same, I work at home and do exercise and I feel that I became better in movement and like that, which also digest better the food, at night I go and do things so the food will be digested. I do things bit by bit.

F4: Physical activity is your normal routine, your normal routine from when you wake up till you go back to sleep at night. How did you spend it, did you move or stayed sitting or all sleeping. At *Thuhur (12:00pm)* you have a break but some would make it long around 5 hours, so how you will spend it, but the active exercise is not, you spend certain timing for growing a certain muscle or make a certain area in our body slimmer or we can say to increase your activeness and daily movement. This is exercise.

M2: it is sports ------ but physical activity it must be practicing sports physical activity and practicing sports or work, so these would be different for me if you said physical activity is sports I would say okay, moving physical activity might be at work; this is physical activity

M3: sports; if he is specialized in one sport in particular, if he is basketball player, if he is football player so he is committed certain kind of training, but physical activity in his work, he could move today in his work more than yesterday and tomorrow he might move more than… and one day he might not move at all. One could create his own physical activity, for example I can’t stay setting all day at work, setting on the chair, I can’t stay setting, I should every half an hour, hour to go out move about an hour and come back. This is called -------. One should create activity in his work, park your car far from the parking, instead of the elevator use the stairs this is called physical activity. But doing sports, it’s about liking a certain sport for example; specialized in sports or plays a certain sport so this is considered his daily work, which is sports, so it is a job so this is his daily work and he has to do it, but the physical activity its when one creates physical activity for himself.

Female 2: different

Female 6: I feel some times I do exercise but I don’t consider myself much active so I feel they are different.

Male 2: there is a difference, the physical activity is on the north side and the sport is in the west side. The physical activity is like your body being active, how is your lifestyle, anything that has activity but sport is you practice sports a physical activity.

M: it’s not a different word it’s just almost this related to each other. When you play sport you will be active, energetic and so on.

M:for me the two of them are the same. If am I going to play sport intuitively I’ll make a physical activity both of them are the ingredients of life.

Male 3: the sport and the physical activity are might be different; the physical activity maybe is a movement; I do exercise this is physical activity. But the sport is playing basketball, football, jogging or walking. This is sport.

Male 5: for me I think the sport, the sport is to be practicing. For example I jog every two or three days in a regular way for me I consider this as sport. The activity is like simple movement getting up or going here and there this is by nature you do it this is an activity, but practicing is to be continuity.

M: when they are a specialty in certain kind of game or sport where there is more commitment in certain periods more than 3 days per week or more than 3 days weekly it turns to be specialty and then the type of sport is determined. According to it the body gets to use on it and becomes accustomed to, but all other kind of sport any movement you do is consider to be sport.

1. **Same**

Female2: I fell it is the same thing, they are the same.

F1: the difference between them you mean?

F1: The definition is that some take the physical activity as the activity of the body from inside; they don’t take it that it is the definition of exercise. We, the generation of elder women, are weak a bit in thinking; I will not say that we are expert as today’s generation. When you ask physical activity, she will tell you what is physical activity, maybe it is something inside myself and I don’t know about it? This is the thought that I have. I have something that I don’t know about.

M1: it is the same thing but each person according to his own needs, excuse me there is one person that is obese; he would do sports more

Female 1: the same thing

Female 3: the same thing

Female 4: physical activity and exercise

Female 5: Yes the same thing.

Female 2: the same.

M: ok one last thing I would like to ask. The work sport and the physical activity is the same thing for you?

M: it’s not a different word it’s just almost this related to each other. When you play sport you will be active, energetic and so on.

M: for me the two of them are the same. If am I going to play sport intuitively I’ll make a physical activity both of them are the ingredients of life.

Male 1: yes it’s almost the same because if you’re doing sport you’re doing physical activity. They are associated with each other.

Male 2: yes they are close to each other. In my opinion they are the same thing approximately.

Male 4: I think they are associated with each other because any sport has a physical activity.

Male 6: they are exactly having the same meaning, but when they are a specialty in certain kind of game or sport where there is more commitment in certain periods more than 3 days per week or more than 3 days weekly it turns to be specialty and then the type of sport is determined. According to it the body gets to use on it and becomes accustomed to, but all other kind of sport any movement you do is consider to be sport.

Male 7: they said it all. I won’t add more...Well same idea.

Interviewer: Are you saying they are one thing?

Male 7: yes, but it differ in the outcome of each, but at the end they are the same.

1. **Relation of PA with Food**

M5: Activity means movement, and as long as it is physical activity, physical means body and this can be anything like exercise or anything else not only exercises that nourishes the body, but also food, drink and most things.

F: healthy diet, it is everything related to sports, healthy diet and weight, that’s it

F: and to be aware not to increase amount of food to not gain weight

F: that either burn calories or moves human blood circulation,

M3: physical activity, the purpose of it is that one would control the excess storage of calories. In a very simple way; there shouldn’t be excess storage of calories in the body so that it wouldn’t be stored as fat after that there would be consequences on health for the fat storage, so physical activity any physical activity that is good for the person;

Female 1: To forget about fast food, no fast food..

Female 2: Vegetables.

Female 3: Water

Female 4: Refrigerator full of vegetables and fruits without Pepsi and no junk food

F: And when exercise in a proper way it makes you follows a good diet in a proper way too

Male 2: exercise is power

Male 2: or food consumption so there will be no harm to the body.

M: it’s eating healthy food

Male 4: They are linked to each other because spots improve the blood circulation that helps in digesting the foods, burn fats and sugar, make the body energetic, and improve the body function. It is also helpful to increase fluids intake especially during summer.

- **Factors that make it difficult for you to engage in Physical Activity**

| **No.** | **Factors that makes it difficult for you to engage in physical activity** | | **References** |
| --- | --- | --- | --- |
|  | **Personal beliefs** | 1. Laziness (47) 2. Lack of will (44) 3. Personal belief- elderly perception (10) 4. Concern about body shape (7) 5. Cultural diversity (6) | **152** |
|  | **Time factor** | 1. Family obligation (32) 2. Work nature (23) 3. Study commitments (9) 4. Time management (8) | **103** |
|  | **Environment** | 1. Physical environment (65)  - Lack of Physical Environment – Work place  1. Natural environment (47) | **97** |
|  | **Lack of support** | 1. Lack of family support (45)  - Having a housemaid (2)  1. Lack of knowledge and awareness (28)  - Lack of awareness-schools (8)  1. Lack of cultural support (24)  - lack of support to woman (12)  1. Lack of group (21) 2. Financial factor (16) 3. Lack of governmental (8) 4. Lack of Media Support (7) | **87** |
|  | **Health Factor** | 1. Mental health (16)  - Presence of depression (3)  1. Presences of diseases (9) 2. Obesity factor (6) 3. Lack of diseases (2) 4. Pregnancy (2) | **56** |

1. **Personal Beliefs**

M2: it is walking but sports [laughing with M2] 50 years it is hard now,

Interviewer 1: makes it difficult now

M1: there is nothing that make it difficult unless it is yourself, if you insist on doing sports you I will do, if you failed in it (meaning you didn’t take action) you will not do it, it means the will

F: oh I don’t want to do sports.

F3: I don’t feel any pleasure when I eat healthy food

F?: right

F3: I don’t feel pleasure or zest, if I force myself I can barely eat it.

F?: right

E?: right

F3: that’s it. That’s why I look over to other kind of food because I don’t feel I would be full from it. That’s it. I don’t like healthy food.

F1: I think it is from yourself, the person should force himself but once you start you will continue,

F2: its need time to see exercise result which consider frustrating factor , so I will see another thing because exercise takes long time to see the result , so I will look to alternative thing takes short time than the exercise.

F: also the effort that i need it to do it to practice exercise, or go to gym, we lack the simple things here

F: for example, ln western they have coach who motivates peoples while here they will provide tools and you go and do exercise by yourself without knowing the way of using it ,

F; secondly , for me and for other people, we feel disappointed when we see the fast result of plastic surgery, we don’t get the result we want fast and so we feel disappointed, this maybe one of the factors.

I1: what do you mean plastic surgery?

F5: for example, plastic surgery cover places that we cannot lose it buy sports

I: body shaping.

M: laziness

Male4: I think the first obstacle is the will, will, form the person himself. He doesn’t like to move for example if he is lazy, like to eat, like to sleep or like to watch T.V. he doesn’t like the movement. The is the obstacle.

M: The will is weak in our student society

M: Thirdly the weak will of the student

M: So, there is deficit and the reason for this deficit is that there is no encouragement for the young people like putting advertisement for them in this aspect.

Male2: most of the young people don’t accept that the sport is … it is rarely when you find it very few. If you see the volunteering work in Qatar who did it start? It was rarely when you see the Qatari young people go for volunteering like volunteering in Qatar volunteering company or Qatar University they all go why?

Male1: when the food is nice and you can’t resist

Samira: nice you mean tasty?

Male1: tasty. If I don’t have to take 100 grams it will increase

Male2: from a personal experience, I did it for 2 weeks, I ate healthy diet but when I saw all the house members eat fatty food I couldn’t resist.

Male5: the advertisement, once you go out from your home, you find the streets full of advertisement. When a restaurant shows a new meal you find that all people saw this meal. ~~(20.17)~~ (laugh). This this will motivate you to try this meal, new meal and looks nice in the photo, so you try it if it is tasty or not, so that will increase the turnout to fast food

M: gym alone is not alone enough, those who go gym focus on body building and we want fitness to go out any play something. That is it for exercise.

M: For myself, If is essential to have the goal to have the body and fit, not only shape, there should be fitness in the end. This is for myself.

M1: There are no barriers regarding the place, regarding the place there are no barriers, but for the initiative and the desire, joy, when you don’t have the desire, this will make you leave exercise. It’s one of the main factors, desire.

M1: do they think it is a waste of time for example?

M3: they might think it is a waste of time…might …

M4: not important.

M3: yes. That is it.

M1: to prevent it no, there are clubs and other places. I think in the State of Qatar the main barrier is the desire, the desire. For myself, there are many students and for different ages they don’t have the desire to start doing exercise. The main problem is the desire, the desire; apart from that everything is available *Al-Hamdulleah* (thank God).

M:other western countries for example, for them exercise is a big part of their lives for different age groups but for us if we looked now you will find that most of the people who practice are for our age group not the other ages, there is no..

M2: There is another factor. When you don’t give a care to play, build muscles or jog or anything , and people around you they don’t give any concern, for examples I will tell someone I want to play and do so many things he will tell me no need, so why I have to do and for what? They don’t care

I: so there is no psychological motive?

M2: yes. Exactly

I: And what frustrates you to not practice, existing of that factor makes you to not practice sports?

M3: with availability of everything, you lack the motive, or lack of knowledge when you get older

M4: things make you lazy to practice sports are the people, family, lack of encouragement, or lack of arranged facilities , also you are not doing the outcome for sports its self , its vary according to your interest, according to outcome you want it. I’m not practicing because I don’t want to live….

M: Fast food is tasty and fast in preparation with low cost that what makes people to alienate from healthy food

I: what are the factors that motives you to eat healthy food, for example if you give choice between foods you know healthy food , so what motives you?

Female1: as usual the people around me they eat fast foods, then why i will not eat like them, even though its is personal things, furthermore the figure is too delicious and tempt you, impossible i will eat healthy food and others eating more beautiful than what I’m eating

I1: What are the factors makes difficult to practices sport, things prevent you or stop you to practices sport? You said [f1: pain]

F1: yes, sever knee pain

I1: ok, is there anything else besides knee pain prevent you to practices sport or any movement?

F1: too much effort prevent me, I’m also hypertensive

I1: what?

F1: hypertensive

I1: hypertensive, ok

I1: and you?

I1: is there something prevents you?

F3: no, but sometimes laziness and the exhaustion only

I1: ok,

F1: yes, not cheese, not mayonnaise, that make you sick, also a lot of meat makes you sick.

I1: ok, good, and you was saying family, rice..?

F2: yes rice, the family and bread, fast food, family want that

I1: family want that?

F2: yes family, fast food cause diseases for people

F1: no body eats homemade food

F3: yes, fast food

F2: These toxins

F:most important thing fast food, fast food are the most dangers thing in human body.

F1: yes that is right, they would leave homemade food.

M1: nothing prevents me but it sometimes being lazy

Interviewer1: being lazy,

M1: being lazy in the home

Interviewer1: and you; the obstacles?

M2: for me the personal itself!

Interviewer1: what are your suggestions and opinions that you would like to say for males in your age on the state level or personal regarding the physical activity how do you encourage them ?

M1: nothing, not that there is responsibilities or that, it is that we don’t do our best, honestly

M2: am agreeing with most of what he said, some duties prevent you from

M1: mostly the sweets. I eat little of it or the fat food like sacrificed meats with full of fat especially if it is in the outside kitchens. My brother knows [laugh] Person should be away from it, honestly or he should burn it with sport, burn it.

F:it is also the culture of the western people, they go out and any form of exercise with their kids, so they would grow having this point inside them, they would do exercise and they won’t feel embarrassed.

F: Because my children don’t like the healthy food. They like fired food, they like *Makboos* with fired chicken (rice with fired chicken). They don’t like grilled foods.

Interviewer1: And you what makes it difficult on you?

F3: at night tiredness makes me not able to sleep.

Interviewer1: what prevents you?

F3: tiredness, tiredness, at night it doesn’t make me sleep, and I stand to pray *Fajr* (dawn) then I lay down and sleep. Hamduleah, I have nothing, my husband encourages me to go and do this, do exercise but I, I don’t find myself.

Interviewer2: you don’t have the likeness to exercise?

F3: I don’t have the likeness to exercise

F3: we cannot, god protect you, we get scared, scared,

F2: for health

F3: you will get scared; you know the mouse while he is eating if he sees people he will get scared. [Laugh] Where are we now, Not like old days;…..now where?

Interviewer2: May God gives you health.

F3: *Walla* it is gone, this is life.

Interviewer1: What are the things that prevent you, what are the barriers?

F3: I will tell you, I like the food that has taste.

Interviewer1: so you also the taste.

F3: Yes. I once had food from the neighbor I said yes that is nice, so I left my food and at that one.

Interviewer1: so you mean worry

M1: yes worry [interruption by M3: confusion] if the person has all of this his sons, his wife, his driver and all of it start to make problems so you would be affected too with it

Interviewer1: so according to all of this it may affect being…

M1: yes very much. If you have a troubled son; what to do? Or a girl that is disobeying

M2: interruption: this is the life’s problems.

M1: yes this is life.

Interviewer1: so all of this may prevents you…

M1: it doesn’t prevent, it restricts you...

Interviewer1: you mean make it harder

M1: exactly makes it harder.

Interviewier1: and what are the things that make it easy for you to do exercise?

M1: you know some people are careless Subhan Allah what happened to them doesn’t matter to them and this is difficult, and some of them the simplest thing may have an effect on them emotionally; physically and mentally. While someone just lives only for him-self but who lives for himself, for his sons, his wives will affect the others and get affected too. Salute you; I think I’m innocent so can I go now [said with smile]

M2: Subhan Allah next day I get dressed and go for walk but something get me distressed I get inside my car and I return back home

M3: interruption: take this idea out of your head. Envy is there but don’t think about it

M2: change your place. Not necessary to go over and over to the same place.

M3: let me tell you something if you believe that you’re envied too much that would have an effect on your life. Don’t believe too much about curse. Keep believing in God only. You know why?! Because as much as they envy you they can’t affect you but the God’s will. If I were you I would even bother myself of what they do.

Interviewer1: you said that…

M1: I used to play in Al-Ahli sport club. In the past it was famous and was called Al-Najah club. But as I told you before I love sport but what I can say after that day (the envy) I became not interested… well I walk after the dawn prayer for 30 min.

M: They are not thankless but they have their own life, husbands, kids, and my son with his wife and this…The whole idea is about…

Interviewer1: you mean these are the things that eases doing sport for you

M1: no, no he’s saying these are the obstacles. The psychological stress.

Interviewer1: what makes it difficult for you follow a health diet?

M1: nothing… just there is, no understanding…

Interviewer1: you mean there is no awareness

M1: yes no awareness. We know the meat and rice but we don’t know what eat with because they are different types of food. We don’t put some other additional dishes next to the main course we just mix these two foods together and that is it [laugh]

M2: despite to what my doctor tells me one day I saw the rice I feel like forget about the doctor [laugh]

Interviewer2: lack of self will.

M2: yes it’s a must.

Male 4: I don’t know, when someone hears the word (sport) he feels lazy, feels lazy to go out to practice sports or anything. He stays at home and that is it, or to go with his car

Male4: I don’t like healthy diet; honestly, I feel this food for sick patients

Male4: I don’t like the healthy food

I: but what are the obstacles that you might face and would prevent you from practicing exercise?

F2: maybe not having enough time, and sometimes the fatigue

F1: the lifestyle itself doesn’t help, sometimes one wants to do exercise but he doesn’t because he knows he will do it only once a month [Laughs] so what is the benefit of it.

M: but it is a psychological status.

Interviewer 1: what is the psychological status?

M2: the psychological status is that the person has his work he is busy so it is laziness, job, work, laziness I can’t!

M3: the stresses around us

M2: the stresses around us

M: there are a lot of factors, the psychological factors, psychological stress at work,

Interviewer 1: psychological factor. Can you explain more?

F2: for example psychologically when you feel you are psychologically stressed you don’t feel you have the desire to do sports,

F2: it’s not... I mean depression, --- you are in depression specifically, you don’t feel you have the desire to do sports or other than sports or anything. Some people get depressed they would eat, other people when they get depressed they don’t have the desire to eat,

[Interruption]

F2: you see there is opposite things, some people get depressed they would eat, some people when they get depressed they lose their appetite.

Interviewer 1: so for you physiological stress prevents you from doing sports

F2: sometimes. Sometime. And sometimes I try to overcome them.

Interviewer 2: how the psychological status ------

F2: enough you feel enough, that your bound, depressed that’s it you are bound you don’t have desire, your desire don’t ask even for sports, as I told you now you have the tendency enough you have depression you don’t have the desire to eat, some people (khalas) their appetite for everything is gone.

Interviewer 1: okay. What are factors makes it difficult for you to do sports?

F1: makes it difficult?

Interviewer 1: yes

F1: sometimes social condition

Interviewer 1: you mean by it?

F1: I mean stresses inside the house,

F2: that you commit from inside,

F1: no... No you will consider it like going to school; it a goal and you want to reach it. (Back ground talk)

F2: Wallah for me…

F1: I used to be like a school girl; I go 2 hours and come back home even that I have children (side talks) once you have a goal you will reach it.

Interviewer 1: and you? What prevents you from doing sports? You have said some things but I want to... Because there points in the middle I want you to say them again

F2: I told you the most that would prevent me if I was psychologically stressed and this is the most that would make me not do sports… as long as I am relaxed psychologically

M: unless he was psychologically ill

M: was tired he can’t walk or something,

M: There is not an obstacle for someone to not be able to practice in my point of view.

M4: I don’t have anything, I have the same thing it’s obvious. I don’t have anything to prevent me. I am a military person I am always at work, practicing work exercise from morning, noon and after noon, maybe the thing that could prevent me it’s only sickness

M4: The same thing. the age, like my age, I have the right to practice sports but without the stress of work and the children and things at home. In my spare time, especially at my age, the age affects the person and every person will die.

M1: talking about myself I don’t like it!

M: because you have to be committed to it; and the human by nature doesn’t want to be committed to things he has to be committed to; also it lacks the sugar etc. so it is not encouraging. But the fatty food is delicious and you want to eat it but the unhealthy food people don’t wanted.

M: I will say again the psychological factor

F:only problem in exercise that it needs commitment and time although it’s nice…

Interviewer3: why there is no healthy food at home?

F1: you mean they be committed to eat health food; not really. This is our nature we love to eat fatty food, there is no…

F1: she’s right. There is no strong will if you have the will you’ll do. Believe me you will do it every day.

Interviewer1 & Interviewer3 [so it’s all about willing]

Interviewer1: what are the factors that prevent you to play sport? the obstacles for you?

F4: maybe sometimes laziness, laziness. Maybe for one week you are lazy; maybe you be forced to do if you joined club that you go for the classes

F: Sometimes If I am very tried I can’t do it but some time when I lay on my back I do it and I can’t go up.

Female 3: whenever I walk I feel my heart beat increase so the tiredness.

F2: (8.3)for me frankly speaking I don’t like it

I1: you don’t like the healthy food

F2: no, I don’t like it.

I1: what does make you dislike it?

Because I think it is all to eat about boiled food, grilled food

F4: I have tendency to eat any food with extra sugar, but I burn it fast, as I work a lot inside the house and I don’t like any one to help

M1: Diseases have nothing to do about that by the way. Diseases never go, if you became sick, you will stay on medication, how many years I have been taking medication. It never goes.

M1: hypertension, diabetes doesn’t work when you do exercise; you have to take your dose, are you going to cure it when we do exercise? It will not work, whether you do exercise or not.

M1: but when your pocket is empty, your wife sick, you will not be active, there won’t be activeness.

I2: there is any difficulties makes you to not do physical activity?

M1: nothing prevent me, no obstacles,

M4: or lazy, maybe A little of laziness

M2: No al *hamduleah* everything is ok.We are young.

Female 1: Temptations.

Female 2: the available food.

Female 1: Temptations.

Female 3: Temptations.

F: Especially the old women who sit at homes, I have my mother in lowwith me I always ask her to go out and we registered her at the diet center and then she used to go and then got laziness. You know the elderly women they got laziness when we go back and force.

F: unlike women because we are encouraged to lose weight to make sure our clothes will fit. Men only wear Thoub(the traditional Qatari male dress) or trousers

F:This destroyed me psychologically and gets me frustratedand thing makes me go there and pay money and go before the time and alone. No it’s better to be at home and gets exercise machine to practice on it. I got the machine and I don’t use it.

F: In the same time I don’t eat much and the physiological state increased this.

Female 6: the physiological state is the difficult thing.

Female 5: I can’t move because the movement is bad to baby. I really understood the value of exercise. How much is the value of health. And we are really in place thanks God.

Even if he has time he ordinarysay I want to sit. May he doesn’t have time he is working all day and he doesn’t have the willing. He can wear sport cloth on Thursdaysand go for a run at Aspire.

Female 3: We have to be realistic they aregetting tried on their work. This thing depends on their job situation also the weather here. Some of them get tried from their work all day and I am standing all day on my feet. I want to rest

Female2: Thursday at the evening can wear sport cloth at least two hours in the week so that the body can move

Female 3: Here he needs the engagement factor. He has the willing but does not the encouragement to release the energy. He needs someone to encourage him in the first place. Someone has to be with him to compete with him. The competition let the human to… For example if there is smart one at the school but he is too shy to tell the teacher the information so whyI do not involve him with it with the group but if he founds the group in the class there is competition and everyone wants to show he’s better than the other. Each one will compete to tell the right information. Each one will say because he wants to prove to his group he is the better.

Female 1: The family’s way of thinking, for instance my mother at home she thinks that the more you are fat the more you are healthy. This opinion is always at my home.

Female 5: ya it’s boring

Female6: Boring

Female 1: Boring

Female 3: laziness and tiredness.

Female 3: laziness no one encourage you at home. If you want to go out no one tell you lets go out so you get laziness and stay at home.

Female 5: I feel that I do exercise but I don’t eat healthy diet so I don’t benefit from it so why I do exercise.

Female 1: Because my shape as woman I should always be beautiful and nice in front of people. Why I looked fat and flabbiness why.

Interviewer: you mean you are shy to do exercise in front of them?

Female 1: yes in front of people.

Interviewer: why?

Female 1: Because my shape is horrible.

Interviewer: ok; anything else! What are the difficulties to you said it.? For example you said when you do exercise your family bring something you like for example why this happen?

Female 3: Luck.

Female 4: luck. Suddenly I do exercise and reduces sugar for suddenly people start to invite me to weddings, parties. Suddenly everything happens when I started.

F:I feel that suddenly if someone wants to make project its food project. If someone wants to make something its suddenly cooking cupcakes I don’t know. Even when we go out its moves and at the end we have to eat dinner at restaurant. I feel that is because there is nothing to do.

F:Let’s sit. I don’t know I feel there is nothing we can do about it and then when we go out we use to this even when someone tells you lets go out with me and I will invite you. No one will tell you let go to walk or let go to the sea or let go to the Cornish together. There are no places outside, so let’s go and I will invite you at a restaurant.

Interviewer: why they think this way for example why they say to you let set in coffee shop instead of the Cornish?

Female 3: Because these people thing wrong.

F: Even when it’s for change, like let’s go to Aspire to gather and suddenly the dining table full of goodies. Always before we leave there is food I don’t know why.

M: the laziness, or for school life or for the carrier life. And the obstacle is you get lazy and tired. In the first day in GYM you’re excited and then with time you get bored of this.

F: The one feels lazy and can’t play.

M:fatigue

Male 3: yes fatigue

M: the factor that prevents me from playing sport is the injuries factor. There are some injuries that are very bad in a way you even can’t continue

M: And regardless the laziness

M: your psychological condition will gets tired because of…

M: So if you’re on the same rhythm, same shape, the same way without changing the direction of sport. For example; if you today start running; today you’ll change the working on the muscles and so on

M: yes exactly diversify the sport ways it shouldn’t be the same way so you don’t feel bored

M: Psychologically it’s a factor in human that helps the person.

M: and also the circumstances, the circumstances for example if you have exam and so on; now I’m in the finale term exam so nobody in this time will go and play football or do exercise. Well that it is.

Male 2: the studying

Interviewer: the studying! .That what prevent you from playing sport.

Male 2: yes

Male 3: the life style

M: The life style as we’re student we go to the university and then we get back; we want to sleep or we go and study there is no time to play sport or to go out and so on

M: And the exercise is something know; the effect of the right sport that the one must put a schedule to follow not according to the mood. So if we’re under pressure because of study the one is facing certain difficulties in doing sport.

M: The second problem where all the Arab suffers from is the laziness it ruined us.

Male 6: I think it’s according to the personal goals for each individual.

M: Nobody comes and nobody of you lives near by the university comes; all of you live far away; am I right or not?! You have other places if you have an open place you’ll play in the *Hosh* (it’s like an open empty space) right or wrong?! So it’s either according to the personal goal or…..

Male 2: it’s according to the personal goal

M: If the goal if the personal has no goal to achieve he’ll not going to do exercise.

M: and maybe the tiredness.

M: Or even ... I am not familiar with…. [Male 6: interruption: if the person is sick…]. If I played once I get tired; so it’s the tiredness; and I get lazy because of the weather that’s why I don’t do exercise in the morning that’s impossible because more recently I started for a while and then I stopped.

M:The European’s community has the culture of taking care of self but in Arab no and vice versa once they go back home from school they find a dish of rice he start to eat and sleeps no this is true no this is real. So may find most of the students are *Mash-Allah* and the weight is… so I believe if we took care of these things I think we we’ll have a healthy environment and better.

Male 5: If the coach is absent the sport class is taken by the other teachers so this is the problem

Male 4: another problem is the attention to football only. I love the basketball but the coach is telling me “my son the basketball has no future either you play football or nothing”. So this makes you frustrated. All the guys play football while am the only one who plays basketball; this is a problem for me. Another thing when a coach or the talent hunters came to choose students, he chooses 1, 2, and 3 and that it is. He came only once; ok; what about us?! Check our skills. Maybe if his performance improved he would be good. But to come and choose only as you like this is not going to work. You made the student to hate the sport.

Male 1: but you can’t blame them because of that. Now when you go for a school and they want to make a school team. They are going to look for the best players in the school

Male 5: you didn’t get his point. He’s talking about the method of selections. For example; I am a teacher. They take only 1, 2, and 3 without paying attention to the rest. Maybe you have a talent that I don’t know it, because I don’t know you personally as a coach and you as a student.

Male 1: just a second! What I am trying to say is when the coach is choosing players for the school team in general. I like to tell you…he goes for the club players because he knows them. Now a school’s coach is about to participate in a champion to present the school. Honestly who is goin26g to choose; you have the club’s players, normal players and players who just love the game that they play it but not that much. Are going to blame the coach why you didn’t choose me. No you can’t blame him [Group talking] let me just finish and then take your time …

Interruption by Male 5: now you play in the university’s basketball team right?

Male 1: yes I do.

Male 5: let us assume that I’m the coach of the basketball’s team and I have a champion I want to participate in it. I came I took A, B, C and D of students because I know them personally that they play sport. I don’t know that you have the skills. I didn’t come to you. I didn’t choose you whether I succeed or I failed. What will happen to you if you knew and I didn’t choose you? You know that you’re better than who did I choose (have personal relationship with them). Would you get sad or not?!

Male 1: yes I’ll get sad if he knew that am ok and didn’t choose me

Male 5: you see! That is what he is trying to tell you. [Group talking]

Male 4: listen the idea is the coach come and pick up only the distinct players only he asks the teacher; where are the distinct players you have? Bring them… [Group talking] The thing is he doesn’t come and talk to you and ask you what do kind of sport you like other than football? No he chooses only football players what about the basketball players. And this teacher who told me that the basketball is not a wide spread sport, because of him I stopped playing it.

M: There is lack of motivation to practice.

M: as well lack of motive

M: Here, there is some kind of laziness due to eating meats, yogurt, rice, bread, and vegetables.

Male 1: said if the person is old and has pain it become difficult for him to practice physical activity, but if the person is ready to practice he will do it even he is young or old. If the person is old he cannot. Most of us would like to walk , For example I am diabetic and if I have the ability to walk for 1-2 kilometers daily I know that my blood sugar level will drop, no doubt, but at evening I may have knee pain because I walked in the morning.

M: In addition, the foods have reasons; I prefer the person to not fill his stomach, instead of eating 50 % eat 30% and do not eat one meal only, it is better to distribute them between breakfast, lunch, and dinner. If he can walk about 10 meters, 20 meters, 100 meters or 1 kilometer after the dinner it would be fine. On the other hands there are some people who eat and immediately sleep and this has an effect on the body and all these things depend on person’s strength and weaknesses

M: People do not have vision and the person is a doctor for himself; a doctor for himself. Also if the person has one meal only and realize what benefits he got from this meal, did he got from it benefits, heaviness, or harm. We have here in the gulf, thanks god; many goods and meals are available. We have a sheep for dinner, a sheep for lunch [other participants laughing and comments conformity with him]. You can find also the dining tables is full of vegetables and sweets so the person feels fullness during the 24 hours and couldn’t find a chance for two- three hours only to let the body to move or feel to feel empty as an opportunity to alternate; thanks god.

Male 1: There are two types of barriers to practice physical activities; the first one is from the person himself, weakness, aging, or the effect of his body.

Male 1: Anyway the years go over and humans also go over. Living person still alive and dead one already died. Whatever you are writing here will not return back the one who died and will not create new people. This is the same track that everyone goes through.

M: Lebanon has the best weather, In Doha and other desert places are the same because everyone has a specific time to be strong and after that he lose his strength and return back to his weakness (he means when the person was child) even if he was landing in the earth or flying into the sky, like the scale index rise until it stop in a specific point whatever are the status and the condition. If diet, weather, health, and doctors can do anything, kings and Sheikh would not die. They can pay lots of money to cure and improve their health condition, but even the money cannot cure them and as I said the person is like the scale index has limit and then it has end.

M: Again regarding the length or shortness of the person age is from the god. Thanks and I know that you people came here to teach us and educate us regarding our health. This life will end and no one will stay till the end. Before 30-40 years nothing was in the land, we have only birds and rabbits, but now cities and buildings are constructing and the people who constructed them gone already, also the people who are building now will also go no one will stay.

M: For example my father lives long time in a good health, but now people reach the age of forty and have many diseases

1. Laziness

F1: for me the laziness is motives me to do physical activity, because laziness is a bad feeling, but it can vary from one person to another

F2: its need time to see exercise result which consider frustrating factor , so I will see another thing because exercise takes long time to see the result , so I will look to alternative thing takes short time than the exercise

F: secondly , for me and for other people, we feel disappointed when we see the fast result of plastic surgery, we don’t get the result we want fast and so we feel disappointed, this maybe one of the factors.

F5: for example, plastic surgery cover places that we cannot lose it buy sports

F: laziness

F: if he is lazy, like to eat, like to sleep or like to watch T.V. he doesn’t like the movement. The is the obstacle.

F: So, there is deficit and the reason for this deficit is that there is no encouragement for the young people like putting advertisement for them in this aspect.

I: that stops you from practise sport?

Female2: yes

Female3: indeed the factor is the time, 24hrs you will be busy even to have one hour or 30min to practice exercise, also if you have to go out till you will reach your destination there is traffic so you will be lazy due to that traffic, if know that you’re going to mix place so its need extra effort, also commitment yes... that only

I1: What are the factors makes difficult to practices sport, things prevent you or stop you to practices sport? You said [f1: pain]

F1: yes, sever knee pain

I1: ok, is there anything else besides knee pain prevent you to practices sport or any movement?

F1: too much effort prevent me, I’m also hypertensive

I1: what?

F1: hypertensive

I1: hypertensive, ok

I1: and you?

I1: is there something prevents you?

F3: no, but sometimes laziness and the exhaustion only

I1: ok,

I1: all of you said, that customs could prevents, pain,

F3: lethargy

I1: pain, laziness, lethargy, what else could prevent from practicing?

F1: no

M1: nothing prevents me but it sometimes being lazy

Interviewer1: being lazy,

M1: being lazy in the home

F: The weather shouldn’t be considered as a factor, it should not stop us. It is sometimes the laziness.

Female3: they are not willing to do effort.

Interviewer1: And you what makes it difficult on you?

F3: at night tiredness makes me not able to sleep.

Interviewer1: what prevents you?

F3: tiredness, tiredness, at night it doesn’t make me sleep, and I stand to pray *Fajr* (dawn) then I lay down and sleep. Hamduleah, I have nothing, my husband encourages me to go and do this, do exercise but I, I don’t find myself.

Interviewer2: you don’t have the likeness to exercise?

F3: I don’t have the likeness to exercise

I: but what are the obstacles that you might face and would prevent you from practicing exercise?

F2: maybe not having enough time, and sometimes the fatigue

M2: the psychological status is that the person has his work he is busy so it is laziness, job, work, laziness I can’t!

M: laziness, also psychologically laziness,

M3: for me it is laziness only, yes I have time but sometimes one feels lazy but he must do sports

M: was tired he can’t walk or something,

M: and traffic load,

M1: Traffic Transport, road, traffic especially at weekend,

Interviewer1: what are the factors that prevent you to play sport? the obstacles for you?

F4: maybe sometimes laziness, laziness. Maybe for one week you are lazy; maybe you be forced to do if you joined club that you go for the classes

F:Sometimes If I am very tried I can’t do it but some time when I lay on my back I do it and I can’t go up.

Female 3: whenever I walk I feel my heart beat increase so the tiredness.

M1: but when your pocket is empty, your wife sick, you will not be active, there won’t be activeness.

M4: or lazy, maybe A little of laziness

F: Especially the old women who sit at homes, I have my mother in lowwith me I always ask her to go out and we registered her at the diet center and then she used to go and thengot laziness. You know the elderly women they got laziness when we go back and force.

Female 3: laziness and tiredness.

Female 3: laziness no one encourage you at home. If you want to go out no one tell you lets go out so you get laziness and stay at home.

M: the laziness, or for school life or for the carrier life. And the obstacle is you get lazy and tired. In the first day in GYM you’re excited and then with time you get bored of this.

M: The one feels lazy and can’t play.

M: fatigue

Male 3: yes fatigue

M: And regardless the laziness

M: So if you’re on the same rhythm, same shape, the same way without changing the direction of sport. For example; if you today start running; today you’ll change the working on the muscles and so on

M: yes exactly diversify the sport ways it shouldn’t be the same way so you don’t feel bored

M: and also the circumstances, the circumstances for example if you have exam and so on; now I’m in the finale term exam so nobody in this time will go and play football or do exercise. Well that it is.

Male 2: the studying

Interviewer: the studying! .That what prevent you from playing sport.

Male 2: yes

Male 3: the life style

M: The life style as we’re student we go to the university and then we get back; we want to sleep or we go and study there is no time to play sport or to go out and so on

The second problem where all the Arab suffers from is the laziness it ruined us.

M; and maybe the tiredness.

M: Or even ... I am not familiar with…. [Male 6: interruption: if the person is sick…]. If I played once I get tired; so it’s the tiredness; and I get lazy because of the weather that’s why I don’t do exercise in the morning that’s impossible because more recently I started for a while and then I stopped.

M: Here, there is some kind of laziness due to eating meats, yogurt, rice, bread, and vegetables.

Male 1: There are two types of barriers to practice physical activities; the first one is from the person himself, weakness, aging, or the effect of his body.

M: For example my father lives long time in a good health, but now people reach the age of forty and have many diseases

1. Lack of will

M1: there is nothing that make it difficult unless it is yourself, if you insist on doing sports you I will do, if you failed in it (meaning you didn’t take action) you will not do it, it means the will

F1: I think it is from yourself, the person should force himself but once you start you will continue,

F: also the effort that i need it to do it to practice exercise, or go to gym, we lack the simple things here

Male4: I think the first obstacle is the will, will, form the person himself. He doesn’t like to move for example if he is lazy, like to eat, like to sleep or like to watch T.V. he doesn’t like the movement. The is the obstacle.

M: The will is weak in our student society

M: Thirdly the weak will of the student

M1: There are no barriers regarding the place, regarding the place there are no barriers, but for the initiative and the desire, joy, when you don’t have the desire, this will make you leave exercise. It’s one of the main factors, desire.

M1: to prevent it no, there are clubs and other places. I think in the State of Qatar the main barrier is the desire, the desire. For myself, there are many students and for different ages they don’t have the desire to start doing exercise. The main problem is the desire, the desire; apart from that everything is available *Al-Hamdulleah* (thank God).

M2: There is another factor. When you don’t give a care to play, build muscles or jog or anything , and people around you they don’t give any concern, for examples I will tell someone I want to play and do so many things he will tell me no need, so why I have to do and for what? They don’t care

I: And what frustrates you to not practice, existing of that factor makes you to not practice sports?

M4: things make you lazy to practice sports are the people, family, lack of encouragement, or lack of arranged facilities , also you are not doing the outcome for sports its self , its vary according to your interest, according to outcome you want it. I’m not practicing because I don’t want to live….

I1: is there something prevents you?

F3: no, but sometimes laziness and the exhaustion only

I1: ok,

M1: nothing prevents me but it sometimes being lazy

Interviewer1: being lazy,

M1: being lazy in the home

Interviewer1: and you; the obstacles?

M2: for me the personal itself!

Interviewer1: what are your suggestions and opinions that you would like to say for males in your age on the state level or personal regarding the physical activity how do you encourage them ?

M1: nothing, not that there is responsibilities or that, it is that we don’t do our best, honestly

M2: am agreeing with most of what he said, some duties prevent you from

F3: at night tiredness makes me not able to sleep.

Interviewer1: what prevents you?

F3: tiredness, tiredness, at night it doesn’t make me sleep, and I stand to pray *Fajr* (dawn) then I lay down and sleep. Hamduleah, I have nothing, my husband encourages me to go and do this, do exercise but I, I don’t find myself.

Interviewer2: you don’t have the likeness to exercise?

F3: I don’t have the likeness to exercise

Interviewier1: and what are the things that make it easy for you to do exercise?

M1: you know some people are careless Subhan Allah what happened to them doesn’t matter to them and this is difficult, and some of them the simplest thing may have an effect on them emotionally; physically and mentally. While someone just lives only for him-self but who lives for himself, for his sons, his wives will affect the others and get affected too. Salute you; I think I’m innocent so can I go now [said with smile]

M2: Subhan Allah next day I get dressed and go for walk but something get me distressed I get inside my car and I return back home

Male 4: I don’t know, when someone hears the word (sport) he feels lazy, feels lazy to go out to practice sports or anything. He stays at home and that is it, or to go with his car

F1: the lifestyle itself doesn’t help, sometimes one wants to do exercise but he doesn’t because he knows he will do it only once a month [Laughs] so what is the benefit of it.

F2: that you commit from inside,

F1: no... No you will consider it like going to school; it a goal and you want to reach it. (Back ground talk)

F2: Wallah for me…

F1: I used to be like a school girl; I go 2 hours and come back home even that I have children (side talks) once you have a goal you will reach it.

F1: she’s right. There is no strong will if you have the will you’ll do. Believe me you will do it every day.

Interviewer1 & Interviewer3 [so it’s all about willing]

M1: but when your pocket is empty, your wife sick, you will not be active, there won’t be activeness.

F: This destroyed me psychologically and gets me frustratedand thing makes me go there and pay money and go before the time and alone. No it’s better to be at home and gets exercise machine to practice on it. I got the machine and I don’t use it.

F: Even if he has time he ordinarysay I want to sit. May he doesn’t have time he is working all day and he doesn’t have the willing. He can wear sport cloth on Thursdaysand go for a run at Aspire.

Female 3: We have to be realistic they aregetting tried on their work. This thing depends on their job situation also the weather here. Some of them get tried from their work all day and I am standing all day on my feet. I want to rest

Female2: Thursday at the evening can wear sport cloth at least two hours in the week so that the body can move

Female 3: Here he needs the engagement factor. He has the willing but does not the encouragement to release the energy. He needs someone to encourage him in the first place. Someone has to be with him to compete with him. The competition let the human to… For example if there is smart one at the school but he is too shy to tell the teacher the information so whyI do not involve him with it with the group but if he founds the group in the class there is competition and everyone wants to show he’s better than the other. Each one will compete to tell the right information. Each one will say because he wants to prove to his group he is the better.

F: I feel that suddenly if someone wants to make project its food project. If someone wants to make something its suddenly cooking cupcakes I don’t know. Even when we go out its moves and at the end we have to eat dinner at restaurant. I feel that is because there is nothing to do.

F; Let’s sit. I don’t know I feel there is nothing we can do about it and then when we go out we use to this even when someone tells you lets go out with me and I will invite you. No one will tell you let go to walk or let go to the sea or let go to the Cornish together. There are no places outside, so let’s go and I will invite you at a restaurant.

Interviewer: why they think this way for example why they say to you let set in coffee shop instead of the Cornish?

Female 3: Because these people thing wrong.

M: the laziness, or for school life or for the carrier life. And the obstacle is you get lazy and tired. In the first day in GYM you’re excited and then with time you get bored of this.

M: The one feels lazy and can’t play

Male 6: I think it’s according to the personal goals for each individual.

M: Nobody comes and nobody of you lives near by the university comes; all of you live far away; am I right or not?! You have other places if you have an open place you’ll play in the *Hosh* (it’s like an open empty space) right or wrong?! So it’s either according to the personal goal or…..

Male 2: it’s according to the personal goal

M: If the goal if the personal has no goal to achieve he’ll not going to do exercise.

M: and maybe the tiredness.

M: Or even ... I am not familiar with…. [Male 6: interruption: if the person is sick…]. If I played once I get tired; so it’s the tiredness; and I get lazy because of the weather that’s why I don’t do exercise in the morning that’s impossible because more recently I started for a while and then I stopped.

M: There is lack of motivation to practice.

M: as well lack of motive

M: In addition, the foods have reasons; I prefer the person to not fill his stomach, instead of eating 50 % eat 30% and do not eat one meal only, it is better to distribute them between breakfast, lunch, and dinner. If he can walk about 10 meters, 20 meters, 100 meters or 1 kilometer after the dinner it would be fine. On the other hands there are some people who eat and immediately sleep and this has an effect on the body and all these things depend on person’s strength and weaknesses

Male 1: There are two types of barriers to practice physical activities; the first one is from the person himself,

1. Personal belief- elderly perception

M2: it is walking but sports [laughing with M2] 50 years it is hard now,

M: also you are not doing the outcome for sports its self , its vary according to your interest, according to outcome you want it. I’m not practicing because I don’t want to live…

M4: The same thing. the age, like my age, I have the right to practice sports but without the stress of work and the children and things at home. In my spare time, especially at my age, the age affects the person and every person will die.

M1: Diseases have nothing to do about that by the way. Diseases never go, if you became sick, you will stay on medication, how many years I have been taking medication. It never goes.

M1: hypertension, diabetes doesn’t work when you do exercise; you have to take your dose, are you going to cure it when we do exercise? It will not work, whether you do exercise or not.

Male 1: said if the person is old and has pain it become difficult for him to practice physical activity, but if the person is ready to practice he will do it even he is young or old. If the person is old he cannot. Most of us would like to walk , For example I am diabetic and if I have the ability to walk for 1-2 kilometers daily I know that my blood sugar level will drop, no doubt, but at evening I may have knee pain because I walked in the morning.

Male 1: There are two types of barriers to practice physical activities; the first one is from the person himself, weakness, aging, or the effect of his body.

Male 1: Anyway the years go over and humans also go over. Living person still alive and dead one already died. Whatever you are writing here will not return back the one who died and will not create new people. This is the same track that everyone goes through.

M: Lebanon has the best weather, In Doha and other desert places are the same because everyone has a specific time to be strong and after that he lose his strength and return back to his weakness (he means when the person was child) even if he was landing in the earth or flying into the sky, like the scale index rise until it stop in a specific point whatever are the status and the condition. If diet, weather, health, and doctors can do anything, kings and Sheikh would not die. They can pay lots of money to cure and improve their health condition, but even the money cannot cure them and as I said the person is like the scale index has limit and then it has end.

M: Again regarding the length or shortness of the person age is from the god. Thanks and I know that you people came here to teach us and educate us regarding our health. This life will end and no one will stay till the end. Before 30-40 years nothing was in the land, we have only birds and rabbits, but now cities and buildings are constructing and the people who constructed them gone already, also the people who are building now will also go no one will stay.

1. Concern about body shape

F2: its need time to see exercise result which consider frustrating factor , so I will see another thing because exercise takes long time to see the result , so I will look to alternative thing takes short time than the exercise.

F: secondly , for me and for other people, we feel disappointed when we see the fast result of plastic surgery, we don’t get the result we want fast and so we feel disappointed, this maybe one of the factors.

I1: what do you mean plastic surgery?

F5: for example, plastic surgery cover places that we cannot lose it buy sports

I: body shaping.

M: gym alone is not alone enough, those who go gym focus on body building and we want fitness to go out any play something. That is it for exercise.

M: For myself, If is essential to have the goal to have the body and fit, not only shape, there should be fitness in the end. This is for myself.

F: unlike women because we are encouraged to lose weight to make sure our clothes will fit. Men only wear Thoub(the traditional Qatari male dress) or trousers

Female 1: Because my shape as woman I should always be beautiful and nice in front of people. Why I looked fat and flabbiness why.

Interviewer: you mean you are shy to do exercise in front of them?

Female 1: yes in front of people.

Interviewer: why?

Female 1: Because my shape is horrible.

Interviewer: ok; anything else! What are the difficulties to you said it.? For example you said when you do exercise your family bring something you like for example why this happen?

Female 3: Luck.

Female 4: luck. Suddenly I do exercise and reduces sugar for suddenly people start to invite me to weddings, parties. Suddenly everything happens when I started.

1. Cultural diversity

F: for example, ln western they have coach who motivates peoples while here they will provide tools and you go and do exercise by yourself without knowing the way of using it ,

M: other western countries for example, for them exercise is a big part of their lives for different age groups but for us if we looked now you will find that most of the people who practice are for our age group not the other ages, there is no..

F: it is also the culture of the western people, they go out and any form of exercise with their kids, so they would grow having this point inside them, they would do exercise and they won’t feel embarrassed.

M: The European’s community has the culture of taking care of self but in Arab no and vice versa once they go back home from school they find a dish of rice he start to eat and sleeps no this is true no this is real. So may find most of the students are *Mash-Allah* and the weight is… so I believe if we took care of these things I think we we’ll have a healthy environment and better.

M: People do not have vision and the person is a doctor for himself; a doctor for himself. Also if the person has one meal only and realize what benefits he got from this meal, did he got from it benefits, heaviness, or harm. We have here in the gulf, thanks god; many goods and meals are available. We have a sheep for dinner, a sheep for lunch [other participants laughing and comments conformity with him]. You can find also the dining tables is full of vegetables and sweets so the person feels fullness during the 24 hours and couldn’t find a chance for two- three hours only to let the body to move or feel to feel empty as an opportunity to alternate; thanks god.

1. **Time factor**

I: ah so you mean if you were not doing sports in the university

F5: if in the house; its effort because of the effort, the factor of the effort and the continuous interruptions that always happen. So the time for sports should be one complete hour.

F3: as she said (f2) time, as university students we don’t have time for physical activities

F5: things that would make me not to practice exercise is that I don’t have time to practices because I’m busy all the time,

Male1: the time, the study, the work. These things make shortage in time. The person can’t match between his study and the sport. The person could delay in his lectures until 6.30 and when going back home it is only for having lunch, taking shower and then no time for sport. He will wake up next morning and has lectures at 8.00am. When does he have time for sport and come back home also he cannot sleep ~~(4.02).~~

Male2: dis arrangement of priorities, time management, laziness and no availability environment for doing sport

Nagla: you have said no availability of suitable environment, what do you mean by suitable environment?

Male2: for example, the sport gardens , like walk path in cornich with this temperature and humidity, so how can I doing sport in such weather, or let them , at least; providing rooms with AC or specific halls for this thing.

Male3: no time, if there is no time as my friend said, in the study, work and the family, so you will not find time for doing sport

Male4: I think the first obstacle is the will, will, form the person himself. He doesn’t like to move for example if he is lazy, like to eat, like to sleep or like to watch T.V. he doesn’t like the movement. The is the obstacle.

Male5: in our societies, the pressures on Arabic men as general are many, in addition to the study the the things he has to finish it for himself in the home and the society. He has many pressures. That consuming most of the time and the time becomes limited for resting and no time left to do any sport activity. for example if you are the eldest one, everything happen become on your responsibility

Male6: first, the pressure in the study within student community, the pressure in the study is a reason that affects the student in which there is no time available for him to do sport. Secondly the well, if you have the well to do sport, in anywhere and any weather, you will try to find the time to do sport. If you have no time, at least put 1/3 hour daily to do sport. This is the problem. This is the biggest problem of the students. The will is weak in our student society in addition to the pressure in the society. For example if you are the only one son, you will take whale the responsibility in the home. This is one of the problems.

Male7: the first thing is the time; there is no enough time to do sport. One of the important reason is the financial ability is not available for the entire student. Not all of the students can go to the gym or to buy the specific sport equipment. Thirdly the weak will of the student

M: The second thing is the availability of time especially that during university, and during the semester, at its beginning there is time to do exercise but when we go deeper, time decreases, and makes it tighter the time.

M2: Maybe work, sometimes work, when you have work pressure, you might not be able to practice exercise. When you have lots of lectures or at schools, it would be difficult to find time for that.

M3: Time tightness is the main thing.

M1: do they think it is a waste of time for example?

M3: they might think it is a waste of time…might …

M4: not important.

M3: yes. That is it.

Interviewer2: How about yourself, what prevents you from doing exercise in the same age group?

M3: time tightness is the first thing, time tightness.

M4: for myself and based on what my colleagues said, for time tightness, you can wake up early in the morning and run for example.

M5: The barrier for me is the pressure, and pressure for me means there is no time. Anything else like pressure from university, there are people who work sometimes, I might say that there is pressure,

I: the factors that prevent or frustrate you to do physical activity?

Female1: time, always time

I:How?

Female1: there is no time, traffic streets at my way back from university to home , once i reach home take rest will need to eat, study , till will reach time that i need to sleep i should sleep so there is no time. The only factor that stops from doing anything is the time.

I: and you what stops you from doing, for example you like one kind of sport and something stops you from do it?

Female2: some people have time crisis but other people have time and they are not able to do sport due to responsibilities

I: how?

Female2: for example you find the parent busy, for example, mother is busy she is studying and working, father also busy and there are young children so you have to came back from university and look after young brother eventually no time at all for myself

I: that stops you from practise sport?

Female2: yes

Female3: indeed the factor is the time, 24hrs you will be busy even to have one hour or 30min to practice exercise, also if you have to go out till you will reach your destination there is traffic so you will be lazy due to that traffic, if know that you’re going to mix place so its need extra effort, also commitment yes... that only

I: All of you mention factors that prevent you are, lacks of time, family obligation are there other factors prevent you?

I: for example you female3 spoke about mixing are there any other factor?

Female3: maybe no advertising for sport club or the importance of sport s especially for women, for me if the gym is mixed i will not think to do or practices any sport as it was during school time, and when i joined university and found gym for women, this encouraged me. Its need to support and advertise women only sports club.

f1: yes at morning sleeping is not good, keeping awake at night is not good.

F1: these days’ people start eating at night, and start to stay awake at night.

I1: ok

F2: time management, we don’t organize our time, wether person from gulf or person who live at gulf

interruption from f1: yes, we changed.

F2: they don’t have time organize for their food or sleep

I1: ok

M3: for me, we don’t have time, all our exercise is in the work. You go out in the morning and you comeback at evening. You come with no time available for exercise, our exercise is work. That is it

M3: but in Sudan in your vacation, you can go and walk, can do sports but here we don’t have the time.

M1: nothing prevents me but it sometimes being lazy

Interviewer1: being lazy,

M1: being lazy in the home or being busy, as you know the homework and children issues

Interviewer1: you mean your responsibilities with family prevent you from doing exercise?

M1: yes

M: You also consider your sons, the study, their home works and some of home responsibilities and social responsibilities

M3: the time. For me I don’t have the time. From going at 6 o’clock I come at 10-11 pm. You have no time. I sleep and wake up at 5.00 pm to do your work. The time only, but when I am in vacation, I organize my exercise.

M1: nothing, not that there is responsibilities or that, it is that we don’t do our best, honestly. It is not the country, we don’t do sport, time is as the brothers here mentioned, everyone is busy with the work, with his sons, schools and home requirements

M2: am agreeing with most of what he said, some duties prevent you from

Female1: the time, to find a time to go out and go home to do sport.

Femae2: the work time and the children *(her children)*

Female2: they sometimes don’t agree to allow me to go out. They have to be with me. Where should I put them? I can’t put them in the walking area. There are many single men there. So I have to be with them in the house.

Female3: yes it is right, the time,

F: the children,

F: For example the children, there are people have children want to go with them, others:” no I have many responsibilities either teaching, house work. All of these are obstacles, so no one go out and do sport.

F: and house work,

Interviewer1: so time?

F2: Yes, home responsibilities and kids make you feel you are enough.

Interviewer1: tired

F2: yes it will make you stop any exercise you practice.

F3: Walla hamduelah we have maids.

Interviewer1: Other than the pain, what else can be a barrier?

F4: I don’t have any other barriers, I have responsibilities, house and children but I can devote some time for that, I can devote some time to practice exercise. At night, I can practice exercise after I put her to bed, yes I can.

M: They are not thankless but they have their own life, husbands, kids, and my son with his wife and this…The whole idea is about…

Male 3: no time

I: but what are the obstacles that you might face and would prevent you from practicing exercise?

F2: maybe not having enough time,

Interviewer 1: what is the psychological status?

M2: the psychological status is that the person has his work he is busy so it is laziness, job, work, laziness I can’t!

M: But he leave it because he can’t find it, I mean there is no time, as the brother had said, the work is on two shifts; most of the people working in private sector work in two shifts; he wakeup early in the morning work, one hour break where he would have lunch and sleep wake up there is nothing he gets back home; if he has a family so he would make sports or sits with the family? He would sit with the family

M1: it’s the same thing, there is no time

M2: the only obstacle is the time

Interviewer 1: the time

M2: it is the time

M: the time

M: there are a lot of factors, the psychological factors, psychological stress at work,

M: familial factors,

M: there is not time,

M2: for the clubs, this activity is present -------- that here the clubs specify the day that you go to do the training not the day you want to go to train, so for example you have Al Khour club,

M2: you have Al Khour club, if you went today and made a membership, they will specify for you to come on this and this days you can’t come on this and this days, why? For example the gym hall, swimming, football, all in appointments

M1: Swimming pool has certain hours.

M2: how do you make appointments obligatory and then you tell me come and practice sports? I like to do sports, I like to do it every day, I have half an hour free, I will come. Okay? But you are forcing me to come at certain times, so this is not membership -----

M2: you make me in the first place if I want to do sports you make me get lazy

M2: the sports are not given enough, sports generally; there are a lot of clubs in Qatar there are a lot of sport activities inside the clubs but unfortunately it is not used because of the routine that is set you come on this and that days don’t come… why? What is the reason?

F1: sometimes social condition. I mean stresses inside the house,

F1: this is for married women. But regular woman; she can do sports,

F1: it is impossible that one would workout at home for an hour or an hour and half on a machine I mean sport machine. this is impossible

Interviewer 1: you have said; social conditions; what do you mean by that?

F1: social conditions, sometimes you get suspended, you get a very stressful period of time, students exams for example, pregnancy, giving birth, familial conditions sometimes these would prevent you maybe for months you don’t even think of sports, you would stop I mean. This all helps.

F: And another thing is time, if it is suitable and being committed to time so this makes me… the difficulties?

F2: if there was a shortage in time available

Interviewer 1: shortage of time

F2: shortage in time but if I want to force myself I would. I mean for me my children study, I have to tutor them, I have ----- (back ground noise) I mean house responsibilities ---- you need to do at least one hour of sports.

F1: the married woman is not the same as the regular woman (mostly she means single woman)

M2: the time if it was in suitable time, if time was available for me I can practice

M3: most thing that act as a barrier is time, and the family connections

I: what do you mean by family connections.

M3: because sometimes you are out on visits with the family so you are concealed.

M5: what prevents might be the time

M: family responsibilities, one could come back from work committed to running errands so this would waste more time.

M6: the most important factor is the factor of time.

M3: if he went to =Aspire=, everything is available there. The most important thing is the factor of time, how to find time to go out of the house. This is the problem.

M4: The same thing. the age, like my age, I have the right to practice sports but without the stress of work and the children and things at home. In my spare time, especially at my age, the age affects the person and every person will die.

M1: firstly getting busy with kids so many responsibilities;

M: lack of time

M4: the most imprortant thing the availabilty of time;

M: as you know working from the morning till the end of the day so you have responsibilities toward your kids and sending them to school; wife; and house. The movement you make inside the house can be consider as exercise. I think it’s quite enough just you did exercise.

M2: no unless am busy.

M: and traffic load,

M1: Traffic Transport, road, traffic especially at weekend,

F3: yes willing, and now it is not there at this time.

F4: the problem is that we want be there is lack of time. There is no enough time for person to commit to exercise; only problem in exercise that it needs commitment and time although it’s nice…

Interviewer1: here we are talking about exercise; we’ll get back to the healthy diet in a while; so the question here what prevents you from doing exercise?

F4: I told you it is about the time!

Interviewer1: time is an obstacle for you

F4: yes time getting busy with the kids, home. You try to go out, but they want to come. here I won’t be comfortable so I decide not to go, so we all sit at home.

F: sometimes the time when you are busy and have no time to go for sport

F2: the weather and the time, there is no time.

M: your wife sick,

Female 2: the time

F: The work

Female 4:My husband is little bit over weight and he thinks to practice sports. Since long time I am persuaded him to practice sports and told me it is ok, but I do not have enough time because I come back at home late after duty, I do not have time to go and practice,

Female 3: yes the problem is in the timing

Female 2: My husband is not obese but he does not like to walk and he does not have time even.

Female 2:well I am totally the opposite Siobhan- Allah I love walking. Even if he has time he ordinarysay I want to sit. May he doesn’t have time he is working all day and he doesn’t have the willing. He can wear sport cloth on Thursdaysand go for a run at Aspire.

Female 3: We have to be realistic they aregetting tried on their work. This thing depends on their job situation also the weather here. Some of them get tried from their work all day and I am standing all day on my feet. I want to rest

Female2: Thursday at the evening can wear sport cloth at least two hours in the week so that the body can move

Female 4: If you don’t have time you cannot make exercise.

Female 4: For sure if you don’t have time you can’t go out to do exercise or go and join Nutrition and fitness club. No time work or study.

Female 2: The time.

M: for me the only thing it’s the circumstances when a person is little bit busy if I don’t have anything I can practice sports, play with my friends football we play anything.

M: the laziness, or for school life or for the carrier life. And the obstacle is you get lazy and tired. In the first day in GYM you’re excited and then with time you get bored of this.

M: circumstances like having my own business and so on

Male 6: the crowd

and also the circumstances, the circumstances for example if you have exam and so on; now I’m in the finale term exam so nobody in this time will go and play football or do exercise. Well that it is.

Male 2: the studying

Interviewer: the studying! .That what prevent you from playing sport.

Male 2: yes

Male 4: the work stress and in between studying and work it effects.

Interviewer: by work stress you mean here in the university?!

Male 4: yes

M: The most cause for me and for the others who suffer from is the work stress and the study. As the student in the university if he’s a student and he’s working he works and he study he spend 10 to 11 hours per day inside the university so it’s hard to find a spare time to go and exercise.

Male 1: we used to go every day and train. I used to train since the school’s day. I was in the high school, at that time I finish my school and then I go to the club immediately after school. I finish school by 1:00 pm and my training is at 3:00- 3:30 or 4:00 pm so I stay in the school with my friends so I won’t go back home because the home was far away a little bit so I go home walking and then I go to the club for training.

M: while this is a personal mistake in in organizing his time and his stuff.

M: work load

Interviewer 2: barrier makes you to not practice?

Male 3: lack of time, work loud

M: barrier of insufficient time;

Male 2: the doctors mentioned three barriers, first if I’m working as doctor, teacher, engineer, when can I go out?

M: I will not deduct my own time to do exercise.

M: There are traffics, cars;

M: and the traffic.

M: No enough time because sometime we are busy

M: and have lots of tasks and when back home we fell exhausted even in sometimes the normal movements during the day are considering as activity.

M: For example my father lives long time in a good health, but now people reach the age of forty and have many diseases. . In addition the nature of the jobs now require prolonged sitting on the desk in front of the computer. . In addition the nature of the jobs now require prolonged sitting on the desk in front of the computer. People become dependent

1. Family obligation

I: ah so you mean if you were not doing sports in the university

F5: if in the house; its effort because of the effort, the factor of the effort and the continuous interruptions that always happen. So the time for sports should be one complete hour.

Male3: no time, if there is no time as my friend said, in the study, work and the family, so you will not find time for doing sport

Male5: in our societies, the pressures on Arabic men as general are many, in addition to the study the the things he has to finish it for himself in the home and the society. He has many pressures. That consuming most of the time and the time becomes limited for resting and no time left to do any sport activity.

Nagla: can you give me an example for these pressures

Male5: for example if you are the eldest one, everything happen become on your responsibility

M: in addition to the pressure in the society. For example if you are the only one son, you will take whale the responsibility in the home. This is one of the problems.

I: and you what stops you from doing, for example you like one kind of sport and something stops you from do it?

Female2: some people have time crisis but other people have time and they are not able to do sport due to responsibilities

I: how?

Female2: for example you find the parent busy, for example, mother is busy she is studying and working, father also busy and there are young children so you have to came back from university and look after young brother eventually no time at all for myself

I: All of you mention factors that prevent you are, lacks of time, family obligation are there other factors prevent you?

I: for example you female3 spoke about mixing are there any other factor?

Female3: maybe no advertising for sport club or the importance of sport s especially for women, for me if the gym is mixed i will not think to do or practices any sport as it was during school time, and when i joined university and found gym for women, this encouraged me. Its need to support and advertise women only sports club.

M1: nothing prevents me but it sometimes being lazy

Interviewer1: being lazy,

M1: being lazy in the home or being busy, as you know the homework and children issues

Interviewer1: you mean your responsibilities with family prevent you from doing exercise?

M1: yes

You also consider your sons, the study, their home works and some of home responsibilities and social responsibilities

M1: nothing, not that there is responsibilities or that, it is that we don’t do our best, honestly. It is not the country, we don’t do sport, time is as the brothers here mentioned, everyone is busy with the work, with his sons, schools and home requirements

M2: am agreeing with most of what he said, some duties prevent you from

Femae2: the work time and the children *(her children)*

Female2: they sometimes don’t agree to allow me to go out. They have to be with me. Where should I put them? I can’t put them in the walking area. There are many single men there. So I have to be with them in the house.

F: the children,

F: For example the children, there are people have children want to go with them, others:” no I have many responsibilities either teaching, house work. All of these are obstacles, so no one go out and do sport.

F: and house work,

Interviewer1: so time?

F2: Yes, home responsibilities and kids make you feel you are enough.

Interviewer1: tired

F2: yes it will make you stop any exercise you practice.

F3: Walla hamduelah we have maids.

Interviewer1: Other than the pain, what else can be a barrier?

F4: I don’t have any other barriers, I have responsibilities, house and children but I can devote some time for that, I can devote some time to practice exercise. At night, I can practice exercise after I put her to bed, yes I can.

M: They are not thankless but they have their own life, husbands, kids, and my son with his wife and this…The whole idea is about…

M: But he leave it because he can’t find it, I mean there is no time, as the brother had said, the work is on two shifts; most of the people working in private sector work in two shifts; he wakeup early in the morning work, one hour break where he would have lunch and sleep wake up there is nothing he gets back home; if he has a family so he would make sports or sits with the family? He would sit with the family

M: familial factors,

Interviewer 1: okay. What are factors makes it difficult for you to do sports?

F1: makes it difficult?

Interviewer 1: yes

F1: sometimes social condition

Interviewer 1: you mean by it?

F1: I mean stresses inside the house,

F1: this is for married women. But regular woman; she can do sports,

F1: it is impossible that one would workout at home for an hour or an hour and half on a machine I mean sport machine

Interviewer 1: tamam

F1: this is impossible

Interviewer 1: you have said; social conditions; what do you mean by that?

F1: social conditions, sometimes you get suspended, you get a very stressful period of time, students exams for example, pregnancy, giving birth, familial conditions sometimes these would prevent you maybe for months you don’t even think of sports, you would stop I mean. This all helps.

F2: I mean for me my children study, I have to tutor them, I have ----- (back ground noise) I mean house responsibilities ---- you need to do at least one hour of sports.

F1: the married woman is not the same as the regular woman (mostly she means single woman)

M3: most thing that act as a barrier is time, and the family connections

I: what do you mean by family connections.

M3: because sometimes you are out on visits with the family so you are concealed.

M: family responsibilities, one could come back from work committed to running errands so this would waste more time.

M1: firstly getting busy with kids so many responsibilities;

M: as you know working from the morning till the end of the day so you have responsibilities toward your kids and sending them to school; wife; and house. The movement you make inside the house can be consider as exercise. I think it’s quite enough just you did exercise.

M2: no unless am busy.

Interviewer1: here we are talking about exercise; we’ll get back to the healthy diet in a while; so the question here what prevents you from doing exercise?

F4: I told you it is about the time!

Interviewer1: time is an obstacle for you

F4: yes time getting busy with the kids, home. You try to go out, but they want to come. here I won’t be comfortable so I decide not to go, so we all sit at home.

M: your wife sick

1. Work nature

Male3: no time, if there is no time as my friend said, in the study, work and the family, so you will not find time for doing sport

M2: Physical activity it is mostly the exercise dosage that the person take on the ordinary days or weekly and that is according to his functional (work) performance. Some people have the chance daily, some people are busy with the work and they don’t have chances except intermittent periods of time. In general this is our understanding to the physical activity which is the exercising time that you allot for yourself.

Interviewer2: things encourage you

M3: for me, we don’t have time, all our exercise is in the work. You go out in the morning and you comeback at evening. You come with no time available for exercise, our exercise is work. That is it

M1: nothing, not that there is responsibilities or that, it is that we don’t do our best, honestly. It is not the country, we don’t do sport, time is as the brothers here mentioned, everyone is busy with the work, with his sons, schools and home requirements

M2: am agreeing with most of what he said, some duties prevent you from

I: what lifestyle do you mean? Can you elaborate?

F1: the work take up all the time

Interviewer 1: what is the psychological status?

M2: the psychological status is that the person has his work he is busy so it is laziness, job, work, laziness I can’t!

M:there are a lot of factors, the psychological factors, psychological stress at work,

Interviewer1: ok now what are the factors that prevent you from doing sport? What is the obstacle?

F1: for me it’s my work nature; this is what prevents me because I can’t move freely.

Interviewer1: the work!

F1: yes the work. I sit on the chair for a very long time working on my computer. That is what stopped me from moving.

Interviewer1: so what are you saying that sitting for a long time is what prevents you from doing sport!

F1: yes at work only [F2 interruption: her work’s nature].

# Interviewer1: so you mean the work’s nature itself is what prevent you from doing exercise!

# F1: but outside the work there is nothing stops me from doing sport.

Interviewer2: you mean you’re captive just during work time you can’t do exercise?! Is there are any others obstacles prevents you from doing sports; for example some people think that tiredness or some of them have a straight shift these might be obstacles for them; what about you?

F3: time. Work time.

F: The work

Female 4:My husband is little bit over weight and he thinks to practice sports. Since long time I am persuaded him to practice sports and told me it is ok, but I do not have enough time because I come back at home late after duty, I do not have time to go and practice,

Female 3: We have to be realistic they aregetting tried on their work. This thing depends on their job situation also the weather here. Some of them get tried from their work all day and I am standing all day on my feet. I want to rest

Female2: Thursday at the evening can wear sport cloth at least two hours in the week so that the body can move

M: for me the only thing it’s the circumstances when a person is little bit busy if I don’t have anything I can practice sports, play with my friends football we play anything.

M: the laziness, or for school life or for the carrier life. And the obstacle is you get lazy and tired. In the first day in GYM you’re excited and then with time you get bored of this.

M; circumstances like having my own business and so on

Male 4: the work stress and in between studying and work it effects.

Interviewer: by work stress you mean here in the university?!

Male 4: yes

The most cause for me and for the others who suffer from is the work stress and the study. As the student in the university if he’s a student and he’s working he works and he study he spend 10 to 11 hours per day inside the university so it’s hard to find a spare time to go and exercise.

M: work load

M: work loud

Male 2: the doctors mentioned three barriers, first if I’m working as doctor, teacher, engineer, when can I go out?

M: I will not deduct my own time to do exercise.

M: and have lots of tasks and when back home we fell exhausted even in sometimes the normal movements during the day are considering as activity.

M: For example my father lives long time in a good health, but now people reach the age of forty and have many diseases. . In addition the nature of the jobs now require prolonged sitting on the desk in front of the computer. . In addition the nature of the jobs now require prolonged sitting on the desk in front of the computer. People become dependent

1. Study commitments

F4: time pressure, studies, sleeping, eating so you find there is not enough time to do sports or work pressure.

F: Besides this if I am in university, I forget myself when I am in the university that’s why I wouldn’t do sports, sometimes I would miss the class

Male1: the time, the study, the work. These things make shortage in time. The person can’t match between his study and the sport. The person could delay in his lectures until 6.30 and when going back home it is only for having lunch, taking shower and then no time for sport. He will wake up next morning and has lectures at 8.00am. When does he have time for sport and come back home also he cannot sleep ~~(4.02).~~

Male2: dis arrangement of priorities, time management, laziness and no availability environment for doing sport

Nagla: you have said no availability of suitable environment, what do you mean by suitable environment?

Male2: for example, the sport gardens , like walk path in cornich with this temperature and humidity, so how can I doing sport in such weather, or let them , at least; providing rooms with AC or specific halls for this thing.

Male3: no time, if there is no time as my friend said, in the study, work and the family, so you will not find time for doing sport

Male4: I think the first obstacle is the will, will, form the person himself. He doesn’t like to move for example if he is lazy, like to eat, like to sleep or like to watch T.V. he doesn’t like the movement. The is the obstacle.

Male5: in our societies, the pressures on Arabic men as general are many, in addition to the study the the things he has to finish it for himself in the home and the society. He has many pressures. That consuming most of the time and the time becomes limited for resting and no time left to do any sport activity.

Nagla: can you give me an example for these pressures

Male5: for example if you are the eldest one, everything happen become on your responsibility

Male6: first, the pressure in the study within student community, the pressure in the study is a reason that affects the student in which there is no time available for him to do sport. Secondly the well, if you have the well to do sport, in anywhere and any weather, you will try to find the time to do sport. If you have no time, at least put 1/3 hour daily to do sport. This is the problem. This is the biggest problem of the students. The will is weak in our student society in addition to the pressure in the society. For example if you are the only one son, you will take whale the responsibility in the home. This is one of the problems.

Male7: the first thing is the time; there is no enough time to do sport. One of the important reason is the financial ability is not available for the entire student. Not all of the students can go to the gym or to buy the specific sport equipment. Thirdly the weak will of the student

I: the factors that prevent or frustrate you to do physical activity?

Female1: time, always time

I:How?

Female1: there is no time, traffic streets at my way back from university to home , once i reach home take rest will need to eat, study , till will reach time that i need to sleep i should sleep so there is no time. The only factor that stops from doing anything is the time.

Male 3: the time, the study

I: you mean because you study ?

Male 3:Yes.

I: that what prevent you?

Male 3:Yes

F2: maybe not having enough time

F1: the work take up all the time and like now we are studying there is no time, moving from one chair to the other [laughs] that’s it.

M: the laziness, or for school life or for the carrier life. And the obstacle is you get lazy and tired. In the first day in GYM you’re excited and then with time you get bored of this.

M: and also the circumstances, the circumstances for example if you have exam and so on; now I’m in the finale term exam so nobody in this time will go and play football or do exercise. Well that it is.

Male 2: the studying

Interviewer: the studying! .That what prevent you from playing sport.

Male 2: yes

1. Time management

Male2: dis arrangement of priorities, time management,

Male3: no time, if there is no time as my friend said, in the study, work and the family, so you will not find time for doing sport

M5: for me as there is only university and nothing else in my life at the moment, it is the matter of time management. If you managed your time, you will find time for everything, for exercise, not exercise, break time, you will find everything.

F2: time management, we don’t organize our time, wether person from gulf or person who live at gulf

interruption from f1: yes, we changed.

F2: they don’t have time organize for their food or sleep

M1: nothing, not that there is responsibilities or that, it is that we don’t do our best, honestly. It is not the country, we don’t do sport, time is as the brothers here mentioned, everyone is busy with the work, with his sons, schools and home requirements

M2: am agreeing with most of what he said, some duties prevent you from

I: but what are the obstacles that you might face and would prevent you from practicing exercise?

F2: maybe not having enough time,

M: while this is a personal mistake in in organizing his time and his stuff.

Interviewer 2: barrier makes you to not practice?

Male 3: lack of time,

1. **Environment**

M:but what affects more is the weather, as long as the weather is humid it affects because of the sweating and this.

M3: it is the weather

Interviewer 1: the weather?

M3: the weather in the summer its ---- generally.

Interviewer 1: And you sir?

M4: the weather also. Can you walk in this weather? You can’t honestly

M3: -------- we practice sports in the streets, half of these streets are digged in and cracked

F1: for example for me here in =Qatar= the heat for example it is impossible that you will try the corniche in the morning it is better in the evening, or if I live in a place where there is no sports hall these are the things that would prevent me from doing sports.

I: and you what prevents you?

F2: as she said, the heat this is the most important thing that makes the one oh I don’t want to do sports.

F: maybe because walking here is not helping weather

F1: because it is from home to car, from the car to home and from car to work, and work to car, and so on, there is no.

F1: yes lifestyle, no bicycles, no walking,

F:weather is not helping, we lack tools that is used to make a good body and practice exercise.

F4: lack of places that motivates you to practice sport for example, ln western they have coach who motivates peoples while here they will provide tools and you go and do exercise by yourself without knowing the way of using it ,

M:and no availability environment for doing sport

Male2: for example, the sport gardens , like walk path in cornich with this temperature and humidity, so how can I doing sport in such weather, or let them , at least; providing rooms with AC or specific halls for this thing.

Interviewer1: what are the things that make it difficult or prevent you from doing exercise?

M1: In the State of Qatar, the weather in general is a reason and I should say that.

M: The second barrier can be the weather. For sure it is better for me to go out and run in place with green areas rather than at home where you will get bored quickly, what will I do at home? Also the space area at home is not like outside for sure.

M4: umm, (yes)

M5: yes the weather sometimes put pressure on us to make something else or work on something else but in the end exercise is a main thing.

M1: I think that the weather decreases the ways to exercise only.

M5: yes but to prevent it no.

M: External factors can affect the person, or lack of spaces for doing exercise could be a negative barrier for the person who wants to practice sports.

M: or lack of arranged facilities ,

I: But there is no comment about sport?

M4: there is the, things that effect environment of this country,

How? Explain more?

M4: the environment, sports started outdoor so when you do at gym or closed hall it will control the environment so will reduce the pleasure as I guess, I don’t know

I: your personal opinion

I: All of you mention factors that prevent you are, lacks of time, family obligation are there other factors prevent you?

I: for example you female3 spoke about mixing are there any other factor?

Female3: maybe no advertising for sport club or the importance of sport s especially for women, for me if the gym is mixed i will not think to do or practices any sport as it was during school time, and when i joined university and found gym for women, this encouraged me. Its need to support and advertise women only sports club.

F: the most important is the weather, when weather is hot it difficult to practice, when temperature is high you cannot practice

M2: We have outdone the others in this concept but our application is failing… why? Because the infrastructure and the cultural background that should improve the concept of the people regarding this, unfortunately we don’t have any percentage it is zero, we don’t have it.

M: Now, this like Al-Rayan garden or Mueather that were for exercise is demolished

F: If it is in hot weather, i can’t.

F: the weather

F: the place ,

F: If all the cultural centers accompany with small gym room for free to the people or participate with few money, this will encourage them. It is not only for medical staff or the employee in the health center, no, also in youth centers. Here they care more in the centers for the men, having swimming pool etc.., but the same youth center in woman branch, the youth centers of men branch and woman branch are completely different. The woman branch is only for meeting room, lectures and etc.., but there is no gym, no swimming pool, nothing like those. On the other hand in the men branch there is football playground and tennis playground and playgrounds for many thing including swimming pool and all of these… if there is one for ladies, that will motivate them to go and it is for free, participate and can go every day. If the weather is hot and we can't go in the garden, then the youth centers for us will be ok.

Female3: of course. If I will take my daughter every day to aspire to participate in the gym or anything, No

Female1: it should be in a place ….

Nagla: available in a near place

Female3: yes. As it old you if it is in a branch near here like the men branch but for ladies, it will be very easy. Because in the men branch they brought a trainer for boy swimming, teach in gym and trainer in football. To allow them to participate is by Aspire. The only place that I have heard there are good activities

Female3: it is impossible after the work and after finishing the house work to take my children to Aspire and bring them back, but if the place is near, it will be easier.

F3: The hotness weather is not helping anyone.

F: in general is the thing that makes me sit at home and the weather.

Interviewer1: other than the weather and pain, what else.

F2: there are no other barriers. I can practice exercise but only the weather and house work,

[M1 interruption: right, the temperature of the weather.]

Interviewer1: so these are some of the obstacles

M3: yes these are the obstacles. Why? You enter a room with A/C…

Interviewer: What are the barriers to engage in physical activity and why those things make it difficult for you to engage in physical activity?

Male 1: you have answered this question now, the lack of places to play in. there are spaces, but there are no playgrounds.

M: The weather is the main reason.

Male 2: the weather

M: the places,

Male 4: it is weathers and it is about not to suffer when you walk.

There are many playgrounds but it is with registration and it will be crowded

Male 2: it should be good one, not artificial when you visit next time you find it ruined. They are many Bedouin who go, beside Bedouin…

Male1: they don’t restore it

Male 2: they don’t restore it. After 2 days it will be ruined. It became useless, no light and no water or juices

Male1: it is better than before because of the world cup, they increased the playgrounds, but still not enough. The playgrounds with reservation are very expensive 500 or 1000 QR/hour, it is not an amount that someone can… that what I think is a motivation. Make more places like *Katara* like the artificial grass mountain. To make one like for walking and exercise for site seeing

F4: It might be because of the right place, he might not find a proper place.

M1: it is the weather, but here it is difficult

M2: for the clubs, this activity is present -------- that here the clubs specify the day that you go to do the training not the day you want to go to train, so for example you have Al Khour club,

[Crosstalk]

M2: you have Al Khour club, if you went today and made a membership, they will specify for you to come on this and this days you can’t come on this and this days, why? For example the gym hall, swimming, football, all in appointments

M1: Swimming pool has certain hours.

[Crosstalk]

M2: how do you make appointments obligatory and then you tell me come and practice sports? I like to do sports, I like to do it every day, I have half an hour free, I will come. Okay? But you are forcing me to come at certain times, so this is not membership -----

[Crosstalk]

M2: you make me in the first place if I want to do sports you make me get lazy

M2: the sports are not given enough, sports generally; there are a lot of clubs in Qatar there are a lot of sport activities inside the clubs but unfortunately it is not used because of the routine that is set you come on this and that days don’t come… why? What is the reason?

F: The weather doesn’t help [all the participants talking at the same time trying to agree with her] the weather doesn’t help you.

F2: you see; when the weather is humid it won’t help you not even the hot weather [F1 interruption: yes not even the hot weather].

F3: life’s concerns.

Interviewer2: life’s concerns!

F3: yes for me for example I have problem in my knees which I can’t do anything so easily.

Interviewer2: so for you the knee’s problem is considered as obstacle for you! [Side talking and laughing by the participants]

F: sometimes the distance to health care centers,

F1: yes the distance to sports and health clubs especially that you find they far away or their timings don’t suit you,

F1: in the club

F1: in the club you would burn more calories than in the house because in the house you might play 15 minutes and then stop but there its routine (systematic); 2 hours sports means 2 hours sports

F2: right

F1: but in the house, you might so sports one day and 10 days you don’t!

F2: right

F1: but there; there is structured system

F2: the thing is that you need to try to, psychologically.

F1: I was a member in a sports club;

M: the work conditions and its nature.

M: it could be the weather, which is in the suitable weather during spring where weather would be better to practice sports,

M: lack of available places to do sport also unsuitable weather.

M: obstacles mostly from the weather,

M: and the weather,

M: climate it’s affecting also,

Female 4: first thing the hot weather.

Female 4: but there is no space at home.

Female 2: The cholesterol is high so I walk at the yard back and force. (Cross talking what makes you difficult to do physical activity?). The hot weather you can’t go out. But now all the places are air conditions.

Female 4: the hot weather because I go out to the yard and come back home. The hot.

F1: the weather. The weather doesn’t help you.

F4: true, when the weather is hot, a person can’t practice the sport even walking. I always prefer walking, but when the weather is hot; even at evening, a person can’t.

F3: the same, the weather and sometimes the time when you are busy and have no time to go for sport

F2: the weather and the time, there is no time.

Female 1: The weather.

Interviewer1: The weather; ok.

Female 2: the weather here is hot.

Female 3: The weather in the gulf area is hot most of the time than cold. The cold lasts only for short time.

F: but the thing prevents me from being exercise is that I don’t have a machine at home if I have machine that would be fine. But these three days that prevent me

F: The weather; when I go out and check the weather I go back and say no.

Female 2: The clubs at Aspire it’s difficult to put your name on it. The reservation is very booked.

Female 4:My husband is little bit over weight and he thinks to practice sports. Since long time I am persuaded him to practice sports and told me it is ok, but I do not have enough time because I come back at home late after duty, I do not have time to go and practice, but I keep pushing him and insist him to go until he get convinced with my advice. When he persuaded and has intention, he went to Aspire to register with intention and think that I should register and should follow diet program as well as practice sport and the person should and think that diet program and practicing sports together help us to reach our desire. He already went to register at Aspire, They told him to come back after three months.

Female 4: to register and we will call you later. The husband asked: “when you are going to call me back?” They replay “within two to three months more”. This is meant that the person needs five months to start practicing sports and implementing the idea that comes to your mind and convince it with difficulties. So he said I am not going to practice.

Female 2: If we do outside exercise is not like inside and if the weather is hot I feel it’s difficult to do exercise, unless it is indoors, then yes.

Female 1: The place the place for the exercise. Where should we do exercise. For example, at home sometimes no room available to do the exercise so I just leave it.

Female 3: Some time there is no machine

Female 2: There is not a lot in Qatar we can do so if we go out we don’t go to an open area, The weather does not encourage us

Female 4: We are used to that, because we are used to this and no one can change this idea. In the summer like this but in the winter may change.

F: the only thing possible that makes not to play sport is the bad weather. The one feels lazy and can’t play.

F: yes the hotness

F: the weather

F: there are football’s training courses, there is not much of these courses

F: You come with your friends to play; playgrounds as needed; 2 football playgrounds for example playgrounds for that and so on.

Male 6: the crowd

M: and the sun.

Male 1: no no! it depends on the weather. The weather honestly here in Qatar in general doesn’t help you to go out by 04:00pm and run a little bit

M: it might be the weather

M: and the temperature.

M: and he didn’t find parking or there is a rush or an opened place or see a certain game.

M: I expect you guys you are not playing in the playgrounds we have in the university the opened one in the summer. Nobody comes and nobody of you lives near by the university comes; all of you live far away; am I right or not?! You have other places if you have an open place you’ll play in the *Hosh* (it’s like an open empty space) right or wrong?! So it’s either according to the personal goal or…..

Male 6: no no; am talking here in general according to the nature of the game or the person who plays it, but the simplest thing the GYM there is no parking to park and there is the rush you need 2 hours to get in. sorry but our general facilities and our streets and the surroundings it doesn’t help.

Male 7: the obstacle is the sun

M: Or even ... I am not familiar with…. [Male 6: interruption: if the person is sick…]. If I played once I get tired; so it’s the tiredness; and I get lazy because of the weather that’s why I don’t do exercise in the morning that’s impossible because more recently I started for a while and then I stopped.

Male 4: for me, I think lack of facilities

M: its located in a difficult place, the time as well, car park is difficult.

M: no available places to do exercise

M: so if country did not facilitate the place,

M: So the first thing is that you did not provide me a place

M: The movement here in Qatar for the one who wants to practice physical activity and cannot find place or walk.

M: There are traffics, cars;

M: sometimes it is hot and dusty. The weather here in the Gulf does not encourage practicing physical activity too much comparing to other countries where the person there can walk and jog

M: Here in The gulf and especially during the summer, old people do not have the ability to go out that is why they should look for the proper time to walk like early morning and in the evening.

M; The second one is the when the person cannot walk outside in the street if he wants because if the hot weather

M; and the traffic.

M: Also when he walk he may find high pavements and do not feel safe because he afraid that someone might hit him. This issue does not affect the alert and young person as much it affects old persons.

Male 2: No designed places are available to play sports.

Male 3: These are the barriers. That parks where we used to go and walk there every day destroyed, so where can we go?

Male 3: yes now we should go to walk on beach, how can I go to the beach daily?

1. Physical environment

M3: -------- we practice sports in the streets, half of these streets are digged in and cracked

F: or if I live in a place where there is no sports hall these are the things that would prevent me from doing sports.

F1: because it is from home to car, from the car to home and from car to work, and work to car, and so on, there is no.

F1: yes lifestyle, no bicycles, no walking,

F: we lack tools that is used to make a good body and practice exercise.

F4: lack of places that motivates you to practice sport for example, ln western they have coach who motivates peoples while here they will provide tools and you go and do exercise by yourself without knowing the way of using it ,

M: and no availability environment for doing sport

Male2: for example, the sport gardens , like walk path in cornich with this temperature and humidity, so how can I doing sport in such weather, or let them , at least; providing rooms with AC or specific halls for this thing.

M: External factors can affect the person, or lack of spaces for doing exercise could be a negative barrier for the person who wants to practice sports.

M: or lack of arranged facilities ,

I: But there is no comment about sport?

M4: there is the, things that effect environment of this country,

How? Explain more?

M4: the environment, sports started outdoor so when you do at gym or closed hall it will control the environment so will reduce the pleasure as I guess, I don’t know

I: your personal opinion

I: All of you mention factors that prevent you are, lacks of time, family obligation are there other factors prevent you?

I: for example you female3 spoke about mixing are there any other factor?

Female3: maybe no advertising for sport club or the importance of sport s especially for women, for me if the gym is mixed i will not think to do or practices any sport as it was during school time, and when i joined university and found gym for women, this encouraged me. Its need to support and advertise women only sports club.

M2: We have outdone the others in this concept but our application is failing… why? Because the infrastructure and the cultural background that should improve the concept of the people regarding this, unfortunately we don’t have any percentage it is zero, we don’t have it.

M: Now, this like Al-Rayan garden or Mueather that were for exercise is demolished

F: the place ,

F: If all the cultural centers accompany with small gym room for free to the people or participate with few money, this will encourage them. It is not only for medical staff or the employee in the health center, no, also in youth centers. Here they care more in the centers for the men, having swimming pool etc.., but the same youth center in woman branch, the youth centers of men branch and woman branch are completely different. The woman branch is only for meeting room, lectures and etc.., but there is no gym, no swimming pool, nothing like those. On the other hand in the men branch there is football playground and tennis playground and playgrounds for many thing including swimming pool and all of these… if there is one for ladies, that will motivate them to go and it is for free, participate and can go every day.

Female3: of course. If I will take my daughter every day to aspire to participate in the gym or anything, No

Female1: it should be in a place ….

*Cross talk*

Nagla: available in a near place

Female3: yes. As it old you if it is in a branch near here like the men branch but for ladies, it will be very easy. Because in the men branch they brought a trainer for boy swimming, teach in gym and trainer in football. To allow them to participate is by Aspire. The only place that I have heard there are good activities

Female3: it is impossible after the work and after finishing the house work to take my children to Aspire and bring them back, but if the place is near, it will be easier.

Interviewer: What are the barriers to engage in physical activity and why those things make it difficult for you to engage in physical activity?

Male 1: you have answered this question now, the lack of places to play in. there are spaces, but there are no playgrounds.

M: the places,

M: There are many playgrounds but it is with registration and it will be crowded

Male 2: it should be good one, not artificial when you visit next time you find it ruined. They are many Bedouin who go, beside Bedouin…

Male1: they don’t restore it

Male 2: they don’t restore it. After 2 days it will be ruined. It became useless, no light and no water or juices

Male1: it is better than before because of the world cup, they increased the playgrounds, but still not enough. The playgrounds with reservation are very expensive 500 or 1000 QR/hour, it is not an amount that someone can… that what I think is a motivation. Make more places like *Katara* like the artificial grass mountain. To make one like for walking and exercise for site seeing

F4: It might be because of the right place, he might not find a proper place.

M2: for the clubs, this activity is present -------- that here the clubs specify the day that you go to do the training not the day you want to go to train, so for example you have Al Khour club,

[Crosstalk]

M2: you have Al Khour club, if you went today and made a membership, they will specify for you to come on this and this days you can’t come on this and this days, why? For example the gym hall, swimming, football, all in appointments

M1: Swimming pool has certain hours.

[Crosstalk]

M2: how do you make appointments obligatory and then you tell me come and practice sports? I like to do sports, I like to do it every day, I have half an hour free, I will come. Okay? But you are forcing me to come at certain times, so this is not membership -----

M2: you make me in the first place if I want to do sports you make me get lazy

M2: the sports are not given enough, sports generally; there are a lot of clubs in Qatar there are a lot of sport activities inside the clubs but unfortunately it is not used because of the routine that is set you come on this and that days don’t come… why? What is the reason?

F: sometimes the distance to health care centers,

F1: yes the distance to sports and health clubs especially that you find they far away or their timings don’t suit you,

F1: in the club

F1: in the club you would burn more calories than in the house because in the house you might play 15 minutes and then stop but there its routine (systematic); 2 hours sports means 2 hours sports

F2: right

F1: but in the house, you might so sports one day and 10 days you don’t!

F2: right

F1: but there; there is structured system

F2: the thing is that you need to try to, psychologically.

F1: I was a member in a sports club;

M: the work conditions and its nature.

M: lack of available places to do sport

Female 4: but there is no space at home.

F: but the thing prevents me from being exercise is that I don’t have a machine at home if I have machine that would be fine. But these three days that prevent me

Female 2: The clubs at Aspire it’s difficult to put your name on it. The reservation is very booked.

Female 4:My husband is little bit over weight and he thinks to practice sports. Since long time I am persuaded him to practice sports and told me it is ok, but I do not have enough time because I come back at home late after duty, I do not have time to go and practice, but I keep pushing him and insist him to go until he get convinced with my advice. When he persuaded and has intention, he went to Aspire to register with intention and think that I should register and should follow diet program as well as practice sport and the person should and think that diet program and practicing sports together help us to reach our desire. He already went to register at Aspire, They told him to come back after three months.

Female 4: to register and we will call you later. The husband asked: “when you are going to call me back?” They replay “within two to three months more”. This is meant that the person needs five months to start practicing sports and implementing the idea that comes to your mind and convince it with difficulties. So he said I am not going to practice.

Female 1: The place the place for the exercise. Where should we do exercise. For example, at home sometimes no room available to do the exercise so I just leave it.

Female 3: Some time there is no machine

Female 2: There is not a lot in Qatar we can do so if we go out we don’t go to an open area,

M: there are football’s training courses, there is not much of these courses

M; You come with your friends to play; playgrounds as needed; 2 football playgrounds for example playgrounds for that and so on.

Male 6: the crowd

and he didn’t find parking or there is a rush or an opened place or see a certain game.

M: I expect you guys you are not playing in the playgrounds we have in the university the opened one in the summer. Nobody comes and nobody of you lives near by the university comes; all of you live far away; am I right or not?! You have other places if you have an open place you’ll play in the *Hosh* (it’s like an open empty space) right or wrong?! So it’s either according to the personal goal or…..

Male 6: no no; am talking here in general according to the nature of the game or the person who plays it, but the simplest thing the GYM there is no parking to park and there is the rush you need 2 hours to get in. sorry but our general facilities and our streets and the surroundings it doesn’t help.

Male 4: for me, I think lack of facilities

M: its located in a difficult place, the time as well, car park is difficult.

M: no available places to do exercise

M: so if country did not facilitate the place,

M: So the first thing is that you did not provide me a place

M: The movement here in Qatar for the one who wants to practice physical activity and cannot find place or walk.

M: There are traffics, cars;

M; and the traffic.

M:Also when he walk he may find high pavements and do not feel safe because he afraid that someone might hit him. This issue does not affect the alert and young person as much it affects old persons.

Male 2: No designed places are available to play sports.

Male 3: These are the barriers. That parks where we used to go and walk there every day destroyed, so where can we go?

Male 3: yes now we should go to walk on beach, how can I go to the beach daily?

- Lack of Physical Environment - Work place

the work conditions and its nature.

M: no available places to do exercise

M: so if country did not facilitate the place,

M: So the first thing is that you did not provide me a place

1. Natural environment

M: but what affects more is the weather, as long as the weather is humid it affects because of the sweating and this.

M3: it is the weather

Interviewer 1: the weather?

M3: the weather in the summer its ---- generally.

Interviewer 1: And you sir?

M4: the weather also. Can you walk in this weather? You can’t honestly

F1: for example for me here in =Qatar= the heat for example it is impossible that you will try the corniche in the morning it is better in the evening, or if I live in a place where there is no sports hall these are the things that would prevent me from doing sports.

I: and you what prevents you?

F2: as she said, the heat this is the most important thing that makes the one oh I don’t want to do sports.

F: maybe because walking here is not helping weather

F: weather is not helping,

Male2: for example, the sport gardens , like walk path in cornich with this temperature and humidity, so how can I doing sport in such weather, or let them , at least; providing rooms with AC or specific halls for this thing.

Interviewer1: what are the things that make it difficult or prevent you from doing exercise?

M1: In the State of Qatar, the weather in general is a reason and I should say that.

M: The second barrier can be the weather. For sure it is better for me to go out and run in place with green areas rather than at home where you will get bored quickly, what will I do at home? Also the space area at home is not like outside for sure.

M4: umm, (yes)

M5: yes the weather sometimes put pressure on us to make something else or work on something else but in the end exercise is a main thing.

M1: I think that the weather decreases the ways to exercise only.

M5: yes but to prevent it no.

F: the most important is the weather, when weather is hot it difficult to practice, when temperature is high you cannot practice

F: If it is in hot weather, i can’t.

F: the weather

F: If the weather is hot and we can't go in the garden, then the youth centers for us will be ok.

F3: The hotness weather is not helping anyone.

F: in general is the thing that makes me sit at home and the weather.

Interviewer1: other than the weather and pain, what else.

F2: there are no other barriers. I can practice exercise but only the weather and house work,

[M1 interruption: right, the temperature of the weather.]

Interviewer1: so these are some of the obstacles

M3: yes these are the obstacles. Why? You enter a room with A/C…

M: The weather is the main reason.

Male 2: the weather

Male 4: it is weathers and it is about not to suffer when you walk.

M1: it is the weather, but here it is difficult

The weather doesn’t help [all the participants talking at the same time trying to agree with her] the weather doesn’t help you.

F2: you see; when the weather is humid it won’t help you not even the hot weather [F1 interruption: yes not even the hot weather].

F3: life’s concerns.

M: it could be the weather, which is in the suitable weather during spring where weather would be better to practice sports,

M: also unsuitable weather.

M: obstacles mostly from the weather,

M: and the weather,

M: climate it’s affecting also,

Female 4: first thing the hot weather.

Female 2: The cholesterol is high so I walk at the yard back and force. (Cross talking what makes you difficult to do physical activity?). The hot weather you can’t go out. But now all the places are air conditions.

Female 4: the hot weather because I go out to the yard and come back home. The hot.

F1: the weather. The weather doesn’t help you.

F4: true, when the weather is hot, a person can’t practice the sport even walking. I always prefer walking, but when the weather is hot; even at evening, a person can’t.

F3: the same, the weather and sometimes the time when you are busy and have no time to go for sport

F2: the weather and the time, there is no time.

Female 1: The weather.

Interviewer1: The weather; ok.

Female 2: the weather here is hot.

Female 3: The weather in the gulf area is hot most of the time than cold. The cold lasts only for short time.

F: The weather; when I go out and check the weather I go back and say no.

Female 2: If we do outside exercise is not like inside and if the weather is hot I feel it’s difficult to do exercise, unless it is indoors, then yes.

F: The weather does not encourage us

Female 4: We are used to that, because we are used to this and no one can change this idea. In the summer like this but in the winter may change.

M: the only thing possible that makes not to play sport is the bad weather. The one feels lazy and can’t play.

M: yes the hotness

M: the weather

M:and the sun.

Male 1: no no! it depends on the weather. The weather honestly here in Qatar in general doesn’t help you to go out by 04:00pm and run a little bit

M: it might be the weather

and the temperature.

Male 7: the obstacle is the sun

Or even ... I am not familiar with…. [Male 6: interruption: if the person is sick…]. If I played once I get tired; so it’s the tiredness; and I get lazy because of the weather that’s why I don’t do exercise in the morning that’s impossible because more recently I started for a while and then I stopped.

M: sometimes it is hot and dusty. The weather here in the Gulf does not encourage practicing physical activity too much comparing to other countries where the person there can walk and jog

M: Here in The gulf and especially during the summer, old people do not have the ability to go out that is why they should look for the proper time to walk like early morning and in the evening.

M: The second one is the when the person cannot walk outside in the street if he wants because if the hot weather

1. **Lack of support**

F5: the effort that I spend playing sports, sometimes when I do sports I have to do a lot of effort s this would discourage me so there should be someone to encourage me, if there is someone to encourage me I do more sports.

F: and community that’s , it is mainly from surrounding that affects.

F1: because it is from home to car, from the car to home and from car to work, and work to car, and so on, there is no.

F: the fees of club are too expensive it reach 3000QR per month . While the cheap club like active Aspire are so far for some people.

Male5: in our societies, the pressures on Arabic men as general are many, in addition to the study the the things he has to finish it for himself in the home and the society. He has many pressures. That consuming most of the time and the time becomes limited for resting and no time left to do any sport activity.

Nagla: can you give me an example for these pressures

Male5: for example if you are the eldest one, everything happen become on your responsibility

M:in addition to the pressure in the society. For example if you are the only one son, you will take whale the responsibility in the home. This is one of the problems.

M: One of the important reason is the financial ability is not available for the entire student. Not all of the students can go to the gym or to buy the specific sport equipment.

M: So, there is deficit and the reason for this deficit is that there is no encouragement for the young people like putting advertisement for them in this aspect.

There are some who would say that exercise is a shame and so the parents might influence them.

Interviewer1: you mean the parents think that exercise is…?

M3: some consider it a shame.

Interviewer1: Shame?

M3: Yes.

Interviewer2: how?

M3: the high standard people and like that.

M1: do they think it is a waste of time for example?

M3: they might think it is a waste of time…might …

M4: not important.

M3: yes. That is it.

Interviewer2: Would you please explain more, in your understanding what you mean when you say that they think it is a shame to do exercise?

M3: exercise in our culture is considered a shame for our women.

M4: Yes that is true.

M3: Even the men in our culture who are of high level, they would think why should I waste time in doing exercise, you are an important person, exercise is not important, you have the money and you don’t need anything.

M1: first of all the Factors frustrating are Companionship, they can be the family. They will say to me for instant, don’t go, it’s not necessary, don’t waste your time. External factors can affect the person, or lack of spaces for doing exercise could be a negative barrier for the person who wants to practice sports.

M2: There is another factor. When you don’t give a care to play, build muscles or jog or anything , and people around you they don’t give any concern, for examples I will tell someone I want to play and do so many things he will tell me no need, so why I have to do and for what? They don’t care

M4: things make you lazy to practice sports are the people, family, lack of encouragement, or lack of arranged facilities , also you are not doing the outcome for sports its self , its vary according to your interest, according to outcome you want it. I’m not practicing because I don’t want to live….

I: and you what stops you from doing, for example you like one kind of sport and something stops you from do it?

Female2: some people have time crisis but other people have time and they are not able to do sport due to responsibilities

I: how?

Female2: for example you find the parent busy, for example, mother is busy she is studying and working, father also busy and there are young children so you have to came back from university and look after young brother eventually no time at all for myself

I: All of you mention factors that prevent you are, lacks of time, family obligation are there other factors prevent you?

I: for example you female3 spoke about mixing are there any other factor?

Female3: maybe no advertising for sport club or the importance of sport s especially for women, for me if the gym is mixed i will not think to do or practices any sport as it was during school time,

I1: what is your opinion about society perception toward woman? As F2 Said.

F3: not usually society had that look, you can see some ladies practice exercise outdoor

interruption from f1: its depend on family

F3: no there is no family like that, this is sports, this is health.

***All answering at same time***

F2: yes you are right but there are some who don’t accept that.

F3: this is health.

I1: can you tell us what do mean when you said [depend on family]?

***Still all of the participants are answering***

F2: when you go outside your husband will not allow you to walk

F1: some family allow to practice sport other they are not

I1: why they don’t permit to do sport?

F2: the narrow view of the society

F1: yes

F2: the narrow view of the society

F3: some male help their wife and take them to Aspire, garden she walks and he walks with her

I1: some families…

F2: they don’t accept

I1: why they don’t accept?

F3: they are conservative

F1: yes conservative.

F2: yes conservative

I1: ok, conservative

F2: When they see the woman after 40th if she walk at streets she became hysteric, she want to become like youth, they would not say the sports for young and adult, no

I1: why they think like that?

F2: because they rose up like that, which make their perception narrow

F1: yes

M2: We have outdone the others in this concept but our application is failing… why? Because the infrastructure and the cultural background that should improve the concept of the people regarding this, unfortunately we don’t have any percentage it is zero, we don’t have it.

M:On the other side we have, I am talking about Sudanese community; we consider the sport on football. This is what we have in the neighborhood even the Sudanese woman doesn’t know the concept of the exercise and physical activity and its importance to the body. This is regarding the girls

Interviewer2: and children

M2: leave the children, the children may play in the street, for this the age stage but for the woman in general, all of these, the government has no awareness in this part. As we lived in Qatar, in comparison to Sudan, it is newly civilized, even for the new arrival person; he would know that he has to practice something new.

M2: To comment on this, this is not an issue, but our perception to it, we say Sudan as an example but that runs on all Arabic countries, for our brothers. Part of the Arabic country, the concept of physical activity is not understandable. The country doesn’t have a base for this like other cultures and nationalities have roles to clarify this issue. I am talking generally.

M:but that doesn’t mean the person should do something as a relief for example: if a person is sick, he must, must take his medication even if he is sleeping he must wake up to take his medication. As I told you the things motivate us is that there is no comparison with other nationalities. We don’t consider it as facts or duties to do it, more important than eating, more important than drinking and more important than the family and to respect the time between us. This is what I want to say.

M:For example there are people who have no financial ability and cannot go to sport places.

Female1: as she said, if there is someone to encourage you and go with you.

Female3: it makes a difference

Female1: it can be good

F: I can’t go in the morning time I can’t go alone to walk.

F: the encouragement which can motivate us.

F: If all the cultural centers accompany with small gym room for free to the people or participate with few money, this will encourage them. It is not only for medical staff or the employee in the health center, no, also in youth centers. Here they care more in the centers for the men, having swimming pool etc.., but the same youth center in woman branch, the youth centers of men branch and woman branch are completely different. The woman branch is only for meeting room, lectures and etc.., but there is no gym, no swimming pool, nothing like those. On the other hand in the men branch there is football playground and tennis playground and playgrounds for many thing including swimming pool and all of these… if there is one for ladies, that will motivate them to go and it is for free, participate and can go every day.

Female3: there is private gym, but youth centers…

Female1: it is expensive?

Female3: yes,

M1: well look. There are so many things that affect you when it comes about exercising. For example; for me they scared me, they told me I have cardiac problem. I had been through prosthetic valve replacement after that I was diagnosed by disc dislodgment; so when I walk for 200-300 meter I start to feel pain in my joints going down till my whole legs so I have to sit for 4-5 min to rest then I can continue walking.

Interviewer1: so you mean worry

M1: yes worry [interruption by M3: confusion] if the person has all of this his sons, his wife, his driver and all of it start to make problems so you would be affected too with it

Interviewer1: so according to all of this it may affect being…

M1: yes very much. If you have a troubled son; what to do? Or a girl that is disobeying

M2: interruption: this is the life’s problems.

M1: yes this is life.

Interviewer1: so all of this may prevents you…

M1: it doesn’t prevent, it restricts you...

Interviewer1: you mean make it harder

M1: exactly makes it harder.

Interviewier1: and what are the things that make it easy for you to do exercise?

M1: you know some people are careless Subhan Allah what happened to them doesn’t matter to them and this is difficult, and some of them the simplest thing may have an effect on them emotionally; physically and mentally. While someone just lives only for him-self but who lives for himself, for his sons, his wives will affect the others and get affected too. Salute you; I think I’m innocent so can I go now [said with smile]

M:Even when there is a playground they make it difficult, you have to pay money, register.

M:if there places, no one there and why? I don’t know. But no one there, so no use to go

Male 2: did you walk in the vacation?

Male 3: in vacation, it is for joyful time with the friends and it is for going out.

M:There are many playgrounds but it is with registration and it will be crowded

Male1: it is better than before because of the world cup, they increased the playgrounds, but still not enough. The playgrounds with reservation are very expensive 500 or 1000 QR/hour, it is not an amount that someone can… that what I think is a motivation. Make more places like *Katara* like the artificial grass mountain. To make one like for walking and exercise for site seeing

F5: or he wouldn’t’ find encouragement from people around him

M: friends would encourage a lot to practice sports or the opposite

F4: no one encourages you!

Interviewer1: what are the things that prevent to do sport?

F1: no encouragement,

Interviewer1: no encouragement

F1: honestly there is no encouragement in my house it’s our life style we eat drinks contains sugar and this is a problem.

F: would be nice also when there is encouragement in case your family does…

F4 interrupting: there is a place just like Aspire but the main problem is there is no space. The club supposed to be spacious place. And a private place for ladies and kids. [F1 interrupting: the fees are high and some people cannot pay] the fess is low but nobody pays and there is no vacancy [F1: interrupting: and people have no time] [F1 interrupting: what if she has no car and her husband is busy?! Aspire; all the people like to go there it’s a known place even in Thursdays…]

M1: but when your pocket is empty,

F: house, and the children make me tied

Female 5: alsoin the dresses not all things comes with it the psychological effects you will not be accepted.

Female 1: Work, ladies gathering. In the work when the ladies get gathering they eat from here and they eat Fatair.

Female 3: the problem that other clubs are expensive, unlike Aspire which is affordable cost about 300 QR per month, or 200 QR, or 600 QR. Other fitness centers are expensive comparing to Aspire.

F: I found some difficulties in the places I go; for example I don’t find encouragement or I don’t have one of my friends in the group. This destroyed me psychologically and gets me frustratedand thing makes me go there and pay money and go before the time and alone. No it’s better to be at home and gets exercise machine to practice on it. I got the machine and I don’t use it.

Female 2: My husband is not obese but he does not like to walk and he does not have time even.

F: The past months I was doing exercise and my mother felt there is something that I am doing behind her in the university. She felt that I am doing exercise and I am losing weight and she kept asking and your face is pale. She said what are doing I said nothing. But at the end she knew I am doing exercise so I had to tell her. She said why, why and she started ordering food for me and get. Everything that I want is available. This idea is wrong.

Female 6: It’s impossible for me to do exercise alone and it’s from the difficulty to find someone. At home when I asked some to do exercise they say no it’s boring we want to go out.

sometimes it could be expensive or cannot afford to join the club its difficult for the person to do exercise

Female 5: Sometimes I decide to do exercise then I get bored, no one will say something about it and to tell you to continue, you just do it by yourself.

Female 3: laziness no one encourage you at home. If you want to go out no one tell you lets go out so you get laziness and stay at home.

Female 5: I feel when I want to diet; my family brings all the food I like

Interviewer: ok; anything else! What are the difficulties to you said it.? For example you said when you do exercise your family bring something you like for example why this happen?

Female 3: Luck.

Female 4: luck. Suddenly I do exercise and reduces sugar for suddenly people start to invite me to weddings, parties. Suddenly everything happens when I started.

Interviewer: So all of these make it difficult for you to do exercise.

Female 4: If they see you. You are not eating. Then they would say “You have to eat”. Then I stop.

Interviewer: Why do they say this?

Female 5: they are worried about me. You are not eating something. They are worried about me that something could happen to me. For this reason they don’t help me. Instead they should encourage me more.

Female 2: they are a lot of campaigns to promote health, but I think the problem is in the whole world the prices of the gym and the fitness center are annual for example its $100 annually. Here you can find it with 300 or 400 in the month.

F:I feel that suddenly if someone wants to make project its food project. If someone wants to make something its suddenly cooking cupcakes I don’t know. Even when we go out its moves and at the end we have to eat dinner at restaurant. I feel that is because there is nothing to do.

F: Let’s sit. I don’t know I feel there is nothing we can do about it and then when we go out we use to this even when someone tells you lets go out with me and I will invite you. No one will tell you let go to walk or let go to the sea or let go to the Cornish together. There are no places outside, so let’s go and I will invite you at a restaurant.

M: suggestions! Approximately they are some youth their economic status doesn’t help them to join GYM

M: there are football’s training courses, there is not much of these courses

M: they but there is a little ask for money

M: So some youth they won’t have the ability to pay for it to join this course so if they would do intensive training courses, not necessary to be football where most of them are for football, they can change it into volleyball, handball.

M: And also there is some GYM asks for a lot of money for 3 months which also doesn’t help a person.

M: Regarding the clubs, for example; I used to be a member in one of the Qatari clubs I was with them for 8 years. In the club there are 30 players; only 3 of them are residents and they ask for 27 Qatari players. For sure it’s so difficult to have 27 Qatari players within the team, so you may in the team 11 players and they don’t come for the training or anything but they come on game’s times. For example daily they arrive to the Qatari’s clubs; a residents young’s or students but the Qatari’s law deterred them from being/ presence in the club and that thing let them to play in the streets or some of them may stop permanently from practicing sport. An evidence for my words; I knew some guys they were they had the ability to reach the Qatari national team because of their strong skills in the sport but because of these laws that stopped them; no *Mash-Allah* they’re over 160kg of weight. Eating and sleeping that’s. The exercising was prevented. So if there was a development in the laws and revised; may be the resident in Doha may be in the Qatari team. It may enhance the sport and would develop it for better, these are the most important. Regardless we must focus on the schools and children because we have the biggest overweight percentage ever in Qatar among children.

M: The European’s community has the culture of taking care of self but in Arab no and vice versa once they go back home from school they find a dish of rice he start to eat and sleeps no this is true no this is real. So may find most of the students are *Mash-Allah* and the weight is… so I believe if we took care of these things I think we we’ll have a healthy environment and better.

Male 6: when someone comes to me comes in the last day of the month to take his salary and buy a box of KitKat and then go home [laughing] and if you didn’t give him his salary his father will say to “where is his salary” [laughing] and [Male 5: interruption: so the student or the player doesn’t come for salary no] [Group talking] [Male 5: the mentally is materialistic; this mentality must be change].

Male 6: he doesn’t have the mentality of joining the team or if there is an Individual game for example because I want to be a champion or to raise the flag of your country; no they don’t have this mentality; it’s impossible it’s finished. Even their parents used to be like this. They don’t have a goal. They don’t have the loyalty. No, no way!

Male 5: Am talking in a base of he was a player in the swimming national team and he achieved a lot on the level of Arab he so he has the spirt of leadership and according to this he starts to talk about it.

Male 7: [Group and side talking with laughing] I think that may help people to do more exercises as you knew the depend on the weather [side talking] and the second thing is the campaigns; there are no much campaigns about sport and even if it’s found like the campaigns of Aspire “Your *Step Is Your Health*” the places are limited. They go for gatherings where these itself has no action min 17: 00. [Male 1interruption]: no but regarding that thing by the way I’ve notice something about these events of jogging and Marathon; I noticed they do more of these event and they publish it too. I’ve my little brother *Mash-Allah* he’s 15 years old. He searches the net daily for it. [Male 6: interruption with laughing]: unknown phrases 17:35

M: I stayed with them around 2 years; my problem with them was I gained medals and then I don’t know what happened. They wanted to bring some new players and those new people from the first 2 champions start to get the first places and with time they star to put us away. Even we were going to the training; the coach is focusing on them more than to focus on us.

Male 6: no there will be no restructuring. Let me tell you something. Previously about 6 or 5 years ago there was goalkeeper in the national team till this day we still remember his name , but if till this day if he became the best goalkeeper on the level of Arab world for 6,5 or 10 years the next day he retires nobody will remember his name. If we are the players don’t remember his name would we remember our (unknown word 23:21)… those are the same players who were 40 or 50 years ago they didn’t change and the new one will come… the essential thing of this is not because this is an American system, British system or Italian system or what is so ever the system is. Not because he succeeds in his country that doesn’t mean he will succeed here (unknown word 23:38)…. Find the problems and try to fix it. I really do respect what you are doing now! You are studying the community you live in and try to find solutions. It doesn’t mean let’s bring American system and see its working. No he’s not going to be a world champion no he’s not. [Male 5: interruption: yes right]

Male 6: I have to study what are my abilities. How to solve their problems! Maybe this one doesn’t need more than few things to be world champion; may be this one needs some talk or maybe motivation or maybe just... I know some people who can do anything, anything to a world champion; just give him QR500 and he get anything; he will do anything just to be world champion just give him QR500; no really I swear it became like drugs.

[Male 5: interruption: all of these are under the rules we mentioned at the beginning; is these rules have to be changed…

Interviewer 2: that is what I was talking about, the restructuring of the mangers who are setting these rules!

Male 6: no, it’s not going to be changed

Male 5: the one who sets these rules and if it has to be changed for the best it’s going to be for the benefit of everyone.

Male 6: please say something logic these rules is not going to be changed. [Group talking]

Male 5: let me tell you something; let me tell you something. I’m in the club and within the 8 years from now the rules were changed every sport’s season [Group talking]

Male 6: doesn’t change for the best or for the worst?

Male 5: well it depends on the mood… [Group talking] for example they may change it this season like register 2 players who are born in the State, next season they registered 5… it’s according to the mood. If there is must be a rule it shouldn’t be according to the opinion of administrator. Like terms 1, 2 and 3… they are some country they have the same rules for 30 or 40 years ago they go back for it, it’s like “The Constitution of the State” never change. There must be very strict rules that never change. It shouldn’t be in the hand of administrator who plays with it. So one the rules are set everyone will know what are the things that he do and doesn’t. For example someone came to register in our club we told him sorry come next season for registration check the new rules maybe you may find something for your own good; why this? , because we don’t have a discipline rules [Interruption by Male 1: yes this thing always happened]. If we have discipline rules these rules is not going to be changed. Anything will be clear in front of us. So these are one of the most suggestions we see for the development of sport.

Male1: few minutes ago he asked me why I stopped playing in the Arabic club. Honestly I couldn’t continue because they were some circumstances and so on. Secondly I shift to as I told you the basketball team, so when I found that I quit the building sport. I focused on the basketball *Allhumdellah* I trained more till I became the team captain this year *Allhumdellah* and when I won the champion I took the prize of the best player of the champion. I wasn’t expected this thing to happened because we took the second place in the champion and in the ceremony honoring they said we are going to honoring the best player in the champion; nobody was expected it everyone was expected to be one of those who took the first place in the champion. But it must be to from all the teams. When they called my name my coach didn’t believe it. I just want to tell you not because I stopped playing the building games I didn’t...

Interviewer: you just find another way

Male 1: yes I found I’ll be more comfortable in the basketball. So I joined the basketball is better, air-conditioned lounge

Male 5: good luck

Interviewer 2: it can be taken by other Mathematic or Islamic teachers [laughing]!

Male 5: If the coach is absent the sport class is taken by the other teachers so this is the problem

Male 4: another problem is the attention to football only. I love the basketball but the coach is telling me “my son the basketball has no future either you play football or nothing”. So this makes you frustrated. All the guys play football while am the only one who plays basketball; this is a problem for me. Another thing when a coach or the talent hunters came to choose students, he chooses 1, 2, and 3 and that it is. He came only once; ok; what about us?! Check our skills. Maybe if his performance improved he would be good. But to come and choose only as you like this is not going to work. You made the student to hate the sport.

Male 1: but you can’t blame them because of that. Now when you go for a school and they want to make a school team. They are going to look for the best players in the school [Group talking]

Male 5: you didn’t get his point. He’s talking about the method of selections. For example; I am a teacher. They take only 1, 2, and 3 without paying attention to the rest. Maybe you have a talent that I don’t know it, because I don’t know you personally as a coach and you as a student.

Male 1: just a second! What I am trying to say is when the coach is choosing players for the school team in general. I like to tell you…he goes for the club players because he knows them. Now a school’s coach is about to participate in a champion to present the school. Honestly who is goin26g to choose; you have the club’s players, normal players and players who just love the game that they play it but not that much. Are going to blame the coach why you didn’t choose me. No you can’t blame him [Group talking] let me just finish and then take your time …

Interruption by Male 5: now you play in the university’s basketball team right?

Male 1: yes I do.

Male 5: let us assume that I’m the coach of the basketball’s team and I have a champion I want to participate in it. I came I took A, B, C and D of students because I know them personally that they play sport. I don’t know that you have the skills. I didn’t come to you. I didn’t choose you whether I succeed or I failed. What will happen to you if you knew and I didn’t choose you? You know that you’re better than who did I choose (have personal relationship with them). Would you get sad or not?!

Male 1: yes I’ll get sad if he knew that am ok and didn’t choose me

Male 5: you see! That is what he is trying to tell you. [Group talking]

Male 4: listen the idea is the coach come and pick up only the distinct players only he asks the teacher; where are the distinct players you have? Bring them… [Group talking] The thing is he doesn’t come and talk to you and ask you what do kind of sport you like other than football? No he chooses only football players what about the basketball players. And this teacher who told me that the basketball is not a wide spread sport, because of him I stopped playing it.

Male 3: He does not have kids [Male 3 and 4 laughing].

Interviewer: So, kids are an obstacle for you to not practice?

Male 3: Most of the time family is an obstacle, when teaching the kids, buying things for kids, no time to do exercise, while this is a personal mistake in in organizing his time and his stuff. But true, the obstacles are in taking kids to school.

M: family

M: thinking about life expenses

Male 3: there is an essential factor that contributes which is education and upbringing. For example when someone came from sport culture were family support child, eventually child will be athletic, also education

Male1: also environment

Male 3: also education when student start healthy diet since childhood and do exercise, It was difficult for us to practice sport because we did not have such supportive environment , unlikely student who have the support from his parents since childhood its different, student will continue to do exercise forever.

Interviewer: So, he will get to do sport for the rest of his life.

Male 3: So the background he came from, if education did not support, if media did not do support to practice sport, people will not practice.

Interviewer 2: barrier makes you to not practice?

Male 3: lack of time, work loud, family pressure.

Participant4: family pressure, lack of education, if the person is not educated and aware, these are considered one of the barriers, if there is no sense of care from home, media or if the sport is not adopted by the country, unlike country that adopts sport you will see sport machine place automatically in everywhere, at work place, as foreign countries do, or as sport machine which are available at Al -Chornish in Qatar or at Aspire. If the country adopts sports as a project, it will direct people to exercise through media, education unlike those who did not adopt, there will be barrier in education, in family and bringing up, barrier of insufficient time; lack of knowledge if person did not practice exercise will be sick or tired. There is lack of motivation to practice.

M: lack of support from the responsible authority

M: lack of athletic thought in being raised

M: for instance how student will develop sports if the sport class was not important. At schools there are no sport classes

Male 4: sports class usually gets taken by math or science.

Male 4: Television and social media especially for youth, instead of practicing sports and participating in physical activities they keep using their mobiles on chatting. Chatting on mobiles takes long time from the person without realizing that. These social media create many problems for the people,

1. Lack of family support

F3: it might be the parents if we don’t have sports machines in the house they wouldn’t encourage to get these machines, or they are not willing to take us other places to a club or so.

I: ah so you mean if you were not doing sports in the university

F5: if in the house; its effort because of the effort, the factor of the effort and the continuous interruptions that always happen. So the time for sports should be one complete hour.

M4: things make you lazy to practice sports are the people, family, lack of encouragement

I: and you what stops you from doing, for example you like one kind of sport and something stops you from do it?

Female2: some people have time crisis but other people have time and they are not able to do sport due to responsibilities

I: how?

Female2: for example you find the parent busy, for example, mother is busy she is studying and working, father also busy and there are young children so you have to came back from university and look after young brother eventually no time at all for myself

M1: being lazy in the home or being busy, as you know the homework and children issues

Interviewer1: you mean your responsibilities with family prevent you from doing exercise?

M1: yes

M: You also consider your sons, the study, their home works and some of home responsibilities and social responsibilities

F: We sometimes tell those people who have stairs in their houses to use it if she has no bone pain, this is also a kind of exercise. If she gives a day off to the housemaid and she did the work (laugh) because hovering the house will burn many calories.

Female3: but as much as the house works burn, it will not burn enough calories as a 1 hour walk. It is muscle effort.

Female1: but there are type of house works burn these high calories

Female3: like what?

Female1: the hovering of the house, hovering burn. That is why you find people in European countries, who don’t have housemaid, are very fit, very fit, only from house work. Of course the people who have gardening, who work in gardening, that burn a lot. Just to reduce food with it (laugh).

Femae2: the work time and the children *(her children)*

Female2: they sometimes don’t agree to allow me to go out. They have to be with me. Where should I put them? I can’t put them in the walking area. There are many single men there. So I have to be with them in the house.

F: the children,

F; For example the children, there are people have children want to go with them, others:” no I have many responsibilities either teaching, house work. All of these are obstacles, so no one go out and do sport.

*Ma Sha Allah* people her have housemaid or two, you may give… ask her” you cook today and I will clean today or to do something else, as for example”.

F: and house work,

Interviewer1: so time?

F2: Yes, home responsibilities and kids make you feel you are enough.

Interviewer1: tired

F2: yes it will make you stop any exercise you practice.

F3: Walla hamduelah we have maids.

Interviewer1: Other than the pain, what else can be a barrier?

F4: I don’t have any other barriers, I have responsibilities, house and children but I can devote some time for that, I can devote some time to practice exercise. At night, I can practice exercise after I put her to bed, yes I can.

Interviewer1: so you mean worry

M1: yes worry [interruption by M3: confusion] if the person has all of this his sons, his wife, his driver and all of it start to make problems so you would be affected too with it

Interviewer1: so according to all of this it may affect being…

M1: yes very much. If you have a troubled son; what to do? Or a girl that is disobeying

M2: interruption: this is the life’s problems.

M1: yes this is life.

Interviewer1: so all of this may prevents you…

M1: it doesn’t prevent, it restricts you...

Interviewer1: you mean make it harder

M1: exactly makes it harder.

Interviewier1: and what are the things that make it easy for you to do exercise?

M1: you know some people are careless Subhan Allah what happened to them doesn’t matter to them and this is difficult, and some of them the simplest thing may have an effect on them emotionally; physically and mentally. While someone just lives only for him-self but who lives for himself, for his sons, his wives will affect the others and get affected too. Salute you; I think I’m innocent so can I go now [said with smile]

F3: it could be the family or the society around him for example, someone wants to reduce his weight, he is fat so he wants to reduce his weight, the people around him don’t convince him with this idea, they say no stop it this idea will not work you will not be able to do it, so people around him will have an effect.

M: But he leave it because he can’t find it, I mean there is no time, as the brother had said, the work is on two shifts; most of the people working in private sector work in two shifts; he wakeup early in the morning work, one hour break where he would have lunch and sleep wake up there is nothing he gets back home; if he has a family so he would make sports or sits with the family? He would sit with the family

M:familial factors,

F1: this is for married women. But regular woman; she can do sports,

F2: here we are, we are married, my husband doesn’t like me going to the sports club, and even my daughters same thing, their husbands; not all of them having the same mentality, some would allow her to go, but for my husband he doesn’t allow me. He wouldn’t accept

Interviewer 1: aha

F2: I have sport machine; use it, but there it is better, no you have the machine (constructing an imaginary conversation with her husband)

F1: it is impossible that one would workout at home for an hour or an hour and half on a machine I mean sport machine

Interviewer 1: tamam

F1: this is impossible

Interviewer 1: you have said; social conditions; what do you mean by that?

F1: social conditions, sometimes you get suspended, you get a very stressful period of time, students exams for example, pregnancy, giving birth, familial conditions sometimes these would prevent you maybe for months you don’t even think of sports, you would stop I mean. This all helps.

F2: I mean for me my children study, I have to tutor them, I have ----- (back ground noise) I mean house responsibilities ---- you need to do at least one hour of sports.

F1: the married woman is not the same as the regular woman (mostly she means single woman)

M3: most thing that act as a barrier is time, and the family connections

I: what do you mean by family connections.

M3: because sometimes you are out on visits with the family so you are concealed.

M: family responsibilities, one could come back from work committed to running errands so this would waste more time.

M1: firstly getting busy with kids so many responsibilities;

M: as you know working from the morning till the end of the day so you have responsibilities toward your kids and sending them to school; wife; and house. The movement you make inside the house can be consider as exercise. I think it’s quite enough just you did exercise.

M2: no unless am busy.

F4: no one encourages you!

Interviewer1: what are the things that prevent to do sport?

F1: no encouragement,

Interviewer1: no encouragement

F1: honestly there is no encouragement in my house it’s our life style we eat drinks contains sugar and this is a problem.

F: would be nice also when there is encouragement in case your family does…

Interviewer1: here we are talking about exercise; we’ll get back to the healthy diet in a while; so the question here what prevents you from doing exercise?

F4: I told you it is about the time!

Interviewer1: time is an obstacle for you

F4: yes time getting busy with the kids, home. You try to go out, but they want to come. here I won’t be comfortable so I decide not to go, so we all sit at home.

F4 interrupting: there is a place just like Aspire but the main problem is there is no space. The club supposed to be spacious place. And a private place for ladies and kids. [F1 interrupting: the fees are high and some people cannot pay] the fess is low but nobody pays and there is no vacancy [F1: interrupting: and people have no time] [F1 interrupting: what if she has no car and her husband is busy?! Aspire; all the people like to go there it’s a known place even in Thursdays…]

M: your wife sick,

F; house, and the children make me tied

Female 2: My husband is not obese but he does not like to walk and he does not have time even.

F: The past months I was doing exercise and my mother felt there is something that I am doing behind her in the university. She felt that I am doing exercise and I am losing weight and she kept asking and your face is pale. She said what are doing I said nothing. But at the end she knew I am doing exercise so I had to tell her. She said why, why and she started ordering food for me and get. Everything that I want is available. This idea is wrong.

Female 6: It’s impossible for me to do exercise alone and it’s from the difficulty to find someone. At home when I asked some to do exercise they say no it’s boring we want to go out.

Female 5: I feel when I want to diet; my family brings all the food I like

Interviewer: So all of these make it difficult for you to do exercise.

Female 4: If they see you. You are not eating. Then they would say “You have to eat”. Then I stop.

Interviewer: Why do they say this?

Female 5: they are worried about me. You are not eating something. They are worried about me that something could happen to me. For this reason they don’t help me. Instead they should encourage me more.

Male 3: He does not have kids [Male 3 and 4 laughing].

Interviewer: So, kids are an obstacle for you to not practice?

Male 3: Most of the time family is an obstacle, when teaching the kids, buying things for kids, no time to do exercise, while this is a personal mistake in in organizing his time and his stuff. But true, the obstacles are in taking kids to school.

M: family

Male 3: there is an essential factor that contributes which is education and upbringing. For example when someone came from sport culture were family support child, eventually child will be athletic, also education

Male1: also environment

Male 3: also education when student start healthy diet since childhood and do exercise, It was difficult for us to practice sport because we did not have such supportive environment , unlikely student who have the support from his parents since childhood its different, student will continue to do exercise forever.

Interviewer: So, he will get to do sport for the rest of his life.

Male 3: So the background he came from, if education did not support,

M: work loud, family pressure.

M:Participant4: family pressure, lack of education, if the person is not educated and aware, these are considered one of the barriers, if there is no sense of care from home, media or if the sport is not adopted by the country, unlike country that adopts sport you will see sport machine place automatically in everywhere, at work place, as foreign countries do, or as sport machine which are available at Al -Chornish in Qatar or at Aspire. If the country adopts sports as a project, it will direct people to exercise through media, education unlike those who did not adopt, there will be barrier in education, in family and bringing up, barrier of insufficient time; lack of knowledge if person did not practice exercise will be sick or tired. There is lack of motivation to practice.

M: lack of athletic thought in being raised

- Lack of Family Support - Having a housemaid

*F: Ma Sha Allah* people her have housemaid or two, you may give… ask her” you cook today and I will clean today or to do something else, as for example”.

F: We sometimes tell those people who have stairs in their houses to use it if she has no bone pain, this is also a kind of exercise. If she gives a day off to the housemaid and she did the work (laugh) because hovering the house will burn many calories.

Female3: but as much as the house works burn, it will not burn enough calories as a 1 hour walk. It is muscle effort.

Female1: but there are type of house works burn these high calories

Female3: like what?

Female1: the hovering of the house, hovering burn. That is why you find people in European countries, who don’t have housemaid, are very fit, very fit, only from house work. Of course the people who have gardening, who work in gardening, that burn a lot. Just to reduce food with it (laugh).

1. Lack of knowledge and awareness

M:for example, ln western they have coach who motivates peoples while here they will provide tools and you go and do exercise by yourself without knowing the way of using it ,

I: And what frustrates you to not practice, existing of that factor makes you to not practice sports?

M3: with availability of everything, you lack the motive, or lack of knowledge when you get older

I: ok

Female4: almost as they said, but there is time at least 30min per day at home to practice sport, teach my children to, from early stage to practice exercise 5-10min

M2: We have outdone the others in this concept but our application is failing… why? Because the infrastructure and the cultural background that should improve the concept of the people regarding this, unfortunately we don’t have any percentage it is zero, we don’t have it. On the other side we have, I am talking about Sudanese community; we consider the sport on football. This is what we have in the neighborhood even the Sudanese woman doesn’t know the concept of the exercise and physical activity and its importance to the body. This is regarding the girls

Interviewer2: and children

M2: leave the children, the children may play in the street, for this the age stage but for the woman in general, all of these, the government has no awareness in this part. As we lived in Qatar, in comparison to Sudan, it is newly civilized, even for the new arrival person; he would know that he has to practice something new.

M2: To comment on this, this is not an issue, but our perception to it, we say Sudan as an example but that runs on all Arabic countries, for our brothers. Part of the Arabic country, the concept of physical activity is not understandable. The country doesn’t have a base for this like other cultures and nationalities have roles to clarify this issue. I am talking generally.

I have notice a lot amount of people here made gastric sleeve from obesity. That Is mean I is … 18 years old! They do… for their stomach.

Female3: they are not willing to do effort.

Female1: because they don’t have foundation.

M1: Diseases have nothing to do about that by the way. Diseases never go, if you became sick, you will stay on medication, how many years I have been taking medication. It never goes.

M1: hypertension, diabetes doesn’t work when you do exercise; you have to take your dose, are you going to cure it when we do exercise? It will not work, whether you do exercise or not.

Female 5: There so may thing important in the university like exercise but we do not put priority to exercise and we do not think of the importance of it in our life in protecting us from the disease and get fat and to understand the value of exercise.

M: Regardless we must focus on the schools and children because we have the biggest overweight percentage ever in Qatar among children.

M: This is a big problem it may lead them to have diabetes, diseases and hypertension are because of no movement.

M: The European’s community has the culture of taking care of self but in Arab no and vice versa once they go back home from school they find a dish of rice he start to eat and sleeps no this is true no this is real. So may find most of the students are *Mash-Allah* and the weight is… so I believe if we took care of these things I think we we’ll have a healthy environment and better.

Male 5: no the sport class is scheduled to be once weekly between the all classes so the playground is available

Interviewer 2: So the buildings are available but the issue is the lack of attention to the sport itself

Male 5: yes exactly even the sport teacher comes holding the ball and then he thought t to them go and play and after 20 or 30 minutes he comes again asking to them go. Ok but some student doesn’t like the football, why do you force them to play this game. In the hottest weather you ask them for a walk. Many injuries happened am talking from the reality. Even the teachers have no experiences but you may find… for example if they increase the number of sport class to be 2 or 3 par of it to be theoretical and the other part is practical so the student would have the sport mentality. Teach them the games and so many other things. Honestly I finished the high school 12 years ago and through it; the coach gives them the ball and tell them to go and play [Male 1: Interruption: yes right]. Even some students keep waiting for the sport class so they can go and have fun somewhere else…

Interviewer 2: it can be taken by other Mathematic or Islamic teachers [laughing]!

Male 5: If the coach is absent the sport class is taken by the other teachers so this is the problem

Male 3: there is an essential factor that contributes which is education and upbringing. For example when someone came from sport culture were family support child, eventually child will be athletic, also education

Male1: also environment

Male 3: also education when student start healthy diet since childhood and do exercise, It was difficult for us to practice sport because we did not have such supportive environment , unlikely student who have the support from his parents since childhood its different, student will continue to do exercise forever.

Interviewer: So, he will get to do sport for the rest of his life.

Male 3: So the background he came from, if education did not support, if media did not do support to practice sport, people will not practice.

Interviewer 2: barrier makes you to not practice?

Male 3: lack of time, work loud, family pressure.

Participant4: family pressure, lack of education, if the person is not educated and aware, these are considered one of the barriers, if there is no sense of care from home, media or if the sport is not adopted by the country, unlike country that adopts sport you will see sport machine place automatically in everywhere, at work place, as foreign countries do, or as sport machine which are available at Al -Chornish in Qatar or at Aspire. If the country adopts sports as a project, it will direct people to exercise through media, education unlike those who did not adopt, there will be barrier in education, in family and bringing up, barrier of insufficient time; lack of knowledge if person did not practice exercise will be sick or tired. There is lack of motivation to practice.

Male1: one of the barrier is lack of awareness as he said

M: Secondly lack of awareness, in my whole life I eat and get obese, then become thin and then fat again, as I don’t have this awareness. The doctor pointed on the worst point, as an individual why I will concern about doing exercise? Give me one reason to practice?

Male 2: I gained and lost weight many times, so due to lack of awareness,

M: lack of athletic thought in being raised

M: for instance how student will develop sports if the sport class was not important. At schools there are no sport classes

Male 4: sports class usually gets taken by math or science.

M: if he did not practice sport or became an athlete person, you will face a lot of diseases. We don’t know about the diseases related to lack of exercise until we are adults. For example, if the diabetic patient has the knowledge of practicing sport earlier will prevent diabetes; he would not have got diabetes. Also obesity or lack of exercise and sedentary life is a risk factor in everything.

M: but the problem that many people they don’t care and are not aware of the value of physical activity especially some elderly people, they just sit in the house, watching TV and do not practice physical activity.

M: but from the people themselves. People do not care about physical activity. I retired ten years ago and the people who carry on walking and other activities remain healthy, however the people who stay only at home and do not practices sport develop many diseases.

- Lack of Knowledge and Awareness- Lack of awareness-schools

Regardless we must focus on the schools and children because we have the biggest overweight percentage ever in Qatar among children.

Male 5: no the sport class is scheduled to be once weekly between the all classes so the playground is available

Interviewer 2: So the buildings are available but the issue is the lack of attention to the sport itself

Male 5: yes exactly even the sport teacher comes holding the ball and then he thought t to them go and play and after 20 or 30 minutes he comes again asking to them go. Ok but some student doesn’t like the football, why do you force them to play this game. In the hottest weather you ask them for a walk. Many injuries happened am talking from the reality. Even the teachers have no experiences but you may find… for example if they increase the number of sport class to be 2 or 3 par of it to be theoretical and the other part is practical so the student would have the sport mentality. Teach them the games and so many other things. Honestly I finished the high school 12 years ago and through it; the coach gives them the ball and tell them to go and play [Male 1: Interruption: yes right]. Even some students keep waiting for the sport class so they can go and have fun somewhere else…

Interviewer 2: it can be taken by other Mathematic or Islamic teachers [laughing]!

Male 5: If the coach is absent the sport class is taken by the other teachers so this is the problem

Male 3: there is an essential factor that contributes which is education and upbringing. For example when someone came from sport culture were family support child, eventually child will be athletic, also education

Male1: also environment

Male 3: also education when student start healthy diet since childhood and do exercise, It was difficult for us to practice sport because we did not have such supportive environment , unlikely student who have the support from his parents since childhood its different, student will continue to do exercise forever.

Interviewer: So, he will get to do sport for the rest of his life.

Male 3: So the background he came from, if education did not support, if media did not do support to practice sport, people will not practice.

Male 3: lack of time, work loud, family pressure.

M: Participant4: family pressure, lack of education, if the person is not educated and aware, these are considered one of the barriers, if there is no sense of care from home, media or if the sport is not adopted by the country, unlike country that adopts sport you will see sport machine place automatically in everywhere, at work place, as foreign countries do, or as sport machine which are available at Al -Chornish in Qatar or at Aspire. If the country adopts sports as a project, it will direct people to exercise through media, education unlike those who did not adopt, there will be barrier in education, in family and bringing up, barrier of insufficient time; lack of knowledge if person did not practice exercise will be sick or tired. There is lack of motivation to practice.

M: lack of athletic thought in being raised

M: for instance how student will develop sports if the sport class was not important. At schools there are no sport classes

Male 4: sports class usually gets taken by math or science.

1. Lack of cultural support

Male5: in our societies, the pressures on Arabic men as general are many, in addition to the study the the things he has to finish it for himself in the home and the society. He has many pressures. That consuming most of the time and the time becomes limited for resting and no time left to do any sport activity.

M: There are some who would say that exercise is a shame and so the parents might influence them.

Interviewer1: you mean the parents think that exercise is…?

M3: some consider it a shame.

Interviewer1: Shame?

M3: Yes.

Interviewer2: how?

M3: the high standard people and like that.

M1: do they think it is a waste of time for example?

M3: they might think it is a waste of time…might …

M4: not important.

M3: yes. That is it.

Interviewer2: Would you please explain more, in your understanding what you mean when you say that they think it is a shame to do exercise?

M3: exercise in our culture is considered a shame for our women.

M4: Yes that is true.

M3: Even the men in our culture who are of high level, they would think why should I waste time in doing exercise, you are an important person, exercise is not important, you have the money and you don’t need anything.

I: All of you mention factors that prevent you are, lacks of time, family obligation are there other factors prevent you?

I: for example you female3 spoke about mixing are there any other factor?

Female3: maybe no advertising for sport club or the importance of sport s especially for women, for me if the gym is mixed i will not think to do or practices any sport as it was during school time,

I1: what is your opinion about society perception toward woman? As F2 Said.

F3: not usually society had that look, you can see some ladies practice exercise outdoor

interruption from f1: its depend on family

F3: no there is no family like that, this is sports, this is health.

***All answering at same time***

F2: yes you are right but there are some who don’t accept that.

F3: this is health.

I1: can you tell us what do mean when you said [depend on family]?

***Still all of the participants are answering***

F2: when you go outside your husband will not allow you to walk

F1: some family allow to practice sport other they are not

I1: why they don’t permit to do sport?

F2: the narrow view of the society

F1: yes

F2: the narrow view of the society

F3: some male help their wife and take them to Aspire, garden she walks and he walks with her

I1: some families…

F2: they don’t accept

I1: why they don’t accept?

F3: they are conservative

F1: yes conservative.

F2: yes conservative

I1: ok, conservative

F2: When they see the woman after 40th if she walk at streets she became hysteric, she want to become like youth, they would not say the sports for young and adult, no

I1: why they think like that?

F2: because they rose up like that, which make their perception narrow

F1: yes

M2: We have outdone the others in this concept but our application is failing… why? Because the infrastructure and the cultural background that should improve the concept of the people regarding this, unfortunately we don’t have any percentage it is zero, we don’t have it.

M: On the other side we have, I am talking about Sudanese community; we consider the sport on football. This is what we have in the neighborhood even the Sudanese woman doesn’t know the concept of the exercise and physical activity and its importance to the body. This is regarding the girls

Interviewer2: and children

M2: leave the children, the children may play in the street, for this the age stage but for the woman in general, all of these, the government has no awareness in this part. As we lived in Qatar, in comparison to Sudan, it is newly civilized, even for the new arrival person; he would know that he has to practice something new.

M2: To comment on this, this is not an issue, but our perception to it, we say Sudan as an example but that runs on all Arabic countries, for our brothers. Part of the Arabic country, the concept of physical activity is not understandable. The country doesn’t have a base for this like other cultures and nationalities have roles to clarify this issue. I am talking generally.

M: but that doesn’t mean the person should do something as a relief for example: if a person is sick, he must, must take his medication even if he is sleeping he must wake up to take his medication. As I told you the things motivate us is that there is no comparison with other nationalities. We don’t consider it as facts or duties to do it, more important than eating, more important than drinking and more important than the family and to respect the time between us. This is what I want to say.

F: If all the cultural centers accompany with small gym room for free to the people or participate with few money, this will encourage them. It is not only for medical staff or the employee in the health center, no, also in youth centers. Here they care more in the centers for the men, having swimming pool etc.., but the same youth center in woman branch, the youth centers of men branch and woman branch are completely different. The woman branch is only for meeting room, lectures and etc.., but there is no gym, no swimming pool, nothing like those. On the other hand in the men branch there is football playground and tennis playground and playgrounds for many thing including swimming pool and all of these… if there is one for ladies, that will motivate them to go and it is for free, participate and can go every day.

Male 2: did you walk in the vacation?

Male 3: in vacation, it is for joyful time with the friends and it is for going out.

Female 5: alsoin the dresses not all things comes with it the psychological effects you will not be accepted.

M: The European’s community has the culture of taking care of self but in Arab no and vice versa once they go back home from school they find a dish of rice he start to eat and sleeps no this is true no this is real. So may find most of the students are *Mash-Allah* and the weight is… so I believe if we took care of these things I think we we’ll have a healthy environment and better.

Interviewer 2: it can be taken by other Mathematic or Islamic teachers [laughing]!

Male 5: If the coach is absent the sport class is taken by the other teachers so this is the problem

M: lack of athletic thought in being raised

M: for instance how student will develop sports if the sport class was not important. At schools there are no sport classes

Male 4: sports class usually gets taken by math or science.

- Lack of Cultural Support - Lack of support to woman

M: There are some who would say that exercise is a shame and so the parents might influence them.

Interviewer1: you mean the parents think that exercise is…?

M3: some consider it a shame.

Interviewer1: Shame?

M3: Yes.

Interviewer2: how?

M3: the high standard people and like that.

Interviewer2: Would you please explain more, in your understanding what you mean when you say that they think it is a shame to do exercise?

M3: exercise in our culture is considered a shame for our women.

M4: Yes that is true.

I: All of you mention factors that prevent you are, lacks of time, family obligation are there other factors prevent you?

I: for example you female3 spoke about mixing are there any other factor?

Female3: maybe no advertising for sport club or the importance of sport s especially for women, for me if the gym is mixed i will not think to do or practices any sport as it was during school time,

I1: what is your opinion about society perception toward woman? As F2 Said.

F3: not usually society had that look, you can see some ladies practice exercise outdoor

interruption from f1: its depend on family

F3: no there is no family like that, this is sports, this is health.

***All answering at same time***

F2: yes you are right but there are some who don’t accept that.

F3: this is health.

I1: can you tell us what do mean when you said [depend on family]?

***Still all of the participants are answering***

F2: when you go outside your husband will not allow you to walk

F1: some family allow to practice sport other they are not

I1: why they don’t permit to do sport?

F2: the narrow view of the society

F1: yes

F2: the narrow view of the society

F3: some male help their wife and take them to Aspire, garden she walks and he walks with her

I1: some families…

F2: they don’t accept

I1: why they don’t accept?

F3: they are conservative

F1: yes conservative.

F2: yes conservative

I1: ok, conservative

F2: When they see the woman after 40th if she walk at streets she became hysteric, she want to become like youth, they would not say the sports for young and adult, no

I1: why they think like that?

F2: because they rose up like that, which make their perception narrow

F1: yes

M: On the other side we have, I am talking about Sudanese community; we consider the sport on football. This is what we have in the neighborhood even the Sudanese woman doesn’t know the concept of the exercise and physical activity and its importance to the body. This is regarding the girls

Interviewer2: and children

M2: leave the children, the children may play in the street, for this the age stage but for the woman in general, all of these, the government has no awareness in this part. As we lived in Qatar, in comparison to Sudan, it is newly civilized, even for the new arrival person; he would know that he has to practice something new.

F: If all the cultural centers accompany with small gym room for free to the people or participate with few money, this will encourage them. It is not only for medical staff or the employee in the health center, no, also in youth centers. Here they care more in the centers for the men, having swimming pool etc.., but the same youth center in woman branch, the youth centers of men branch and woman branch are completely different. The woman branch is only for meeting room, lectures and etc.., but there is no gym, no swimming pool, nothing like those. On the other hand in the men branch there is football playground and tennis playground and playgrounds for many thing including swimming pool and all of these… if there is one for ladies, that will motivate them to go and it is for free, participate and can go every day.

F: Here also, most of the sports and activities for boys in a young age. We must put inside the girls the sense of sport and make it a habit.

F: but the governmental centers are mainly for men. For example the *Zakirah* youth centre, you will find in the men department swimming pool, playground, There all the playgrounds in the men branch. In the woman branch, it is a villa and normal room where they do meetings and seminars memorization *(mostly Qur’an)* it is all on seats, there are no activities. It is only once they go to men branch in a month. It is rare. If the branch itself opens for the girls all the time, there will be chances to…

Female3: as I told you, for boys they have activities and they have football. You find only one place for girls and it is in Doha. What about the population of all these places, where should they go?

Female3: yes. As it old you if it is in a branch near here like the men branch but for ladies, it will be very easy. Because in the men branch they brought a trainer for boy swimming, teach in gym and trainer in football. To allow them to participate is by Aspire. The only place that I have heard there are good activities

1. Lack of group support

F5: the effort that I spend playing sports, sometimes when I do sports I have to do a lot of effort s this would discourage me so there should be someone to encourage me, if there is someone to encourage me I do more sports.

F: and community that’s , it is mainly from surrounding that affects.

M1: first of all the Factors frustrating are Companionship, they can be the family. They will say to me for instant, don’t go, it’s not necessary, don’t waste your time. External factors can affect the person, or lack of spaces for doing exercise could be a negative barrier for the person who wants to practice sports.

M2: There is another factor. When you don’t give a care to play, build muscles or jog or anything , and people around you they don’t give any concern, for examples I will tell someone I want to play and do so many things he will tell me no need, so why I have to do and for what? They don’t care

M4: things make you lazy to practice sports are the people, family, lack of encouragement, or lack of arranged facilities , also you are not doing the outcome for sports its self , its vary according to your interest, according to outcome you want it. I’m not practicing because I don’t want to live….

Female1: as she said, if there is someone to encourage you and go with you.

Female3: it makes a difference

Female1: it can be good

F: I can’t go in the morning time I can’t go alone to walk.

F: the encouragement which can motivate us.

M:if there places, no one there and why? I don’t know. But no one there, so no use to go

F5: or he wouldn’t’ find encouragement from people around him

M: friends would encourage a lot to practice sports or the opposite

F4: no one encourages you!

Interviewer1: what are the things that prevent to do sport?

F1: no encouragement,

Interviewer1: no encouragement

F1: honestly there is no encouragement in my house it’s our life style we eat drinks contains sugar and this is a problem.

F: would be nice also when there is encouragement in case your family does…

Female 1: Work, ladies gathering. In the work when the ladies get gathering they eat from here and they eat Fatair.

F: I found some difficulties in the places I go; for example I don’t find encouragement or I don’t have one of my friends in the group. This destroyed me psychologically and gets me frustratedand thing makes me go there and pay money and go before the time and alone. No it’s better to be at home and gets exercise machine to practice on it. I got the machine and I don’t use it.

Female 5: Sometimes I decide to do exercise then I get bored, no one will say something about it and to tell you to continue, you just do it by yourself.

Female 3: laziness no one encourage you at home. If you want to go out no one tell you lets go out so you get laziness and stay at home.

F: I feel that suddenly if someone wants to make project its food project. If someone wants to make something its suddenly cooking cupcakes I don’t know. Even when we go out its moves and at the end we have to eat dinner at restaurant. I feel that is because there is nothing to do.

F: Let’s sit. I don’t know I feel there is nothing we can do about it and then when we go out we use to this even when someone tells you lets go out with me and I will invite you. No one will tell you let go to walk or let go to the sea or let go to the Cornish together. There are no places outside, so let’s go and I will invite you at a restaurant.

1. Financial factor

M: the fees of club are too expensive it reach 3000QR per month . While the cheap club like active Aspire are so far for some people.

M: One of the important reason is the financial ability is not available for the entire student. Not all of the students can go to the gym or to buy the specific sport equipment.

F: For example there are people who have no financial ability and cannot go to sport places.

Female3: there is private gym, but youth centers…

Female1: it is expensive?

Female3: yes,

M: Even when there is a playground they make it difficult, you have to pay money, register.

M: There are many playgrounds but it is with registration and it will be crowded

Male1: it is better than before because of the world cup, they increased the playgrounds, but still not enough. The playgrounds with reservation are very expensive 500 or 1000 QR/hour, it is not an amount that someone can… that what I think is a motivation. Make more places like *Katara* like the artificial grass mountain. To make one like for walking and exercise for site seeing

M1: but when your pocket is empty,

Female 3: the problem that other clubs are expensive, unlike Aspire which is affordable cost about 300 QR per month, or 200 QR, or 600 QR. Other fitness centers are expensive comparing to Aspire.

F:sometimes it could be expensive or cannot afford to join the club its difficult for the person to do exercise

Female 2: they are a lot of campaigns to promote health, but I think the problem is in the whole world the prices of the gym and the fitness center are annual for example its $100 annually. Here you can find it with 300 or 400 in the month.

M: suggestions! Approximately they are some youth their economic status doesn’t help them to join GYM

M: they but there is a little ask for money

M: So some youth they won’t have the ability to pay for it to join this course so if they would do intensive training courses, not necessary to be football where most of them are for football, they can change it into volleyball, handball.

M:And also there is some GYM asks for a lot of money for 3 months which also doesn’t help a person.

M: thinking about life expenses

1. Lack of Government Support

M1: well look. There are so many things that affect you when it comes about exercising. For example; for me they scared me, they told me I have cardiac problem. I had been through prosthetic valve replacement after that I was diagnosed by disc dislodgment; so when I walk for 200-300 meter I start to feel pain in my joints going down till my whole legs so I have to sit for 4-5 min to rest then I can continue walking.

M: there are football’s training courses, there is not much of these courses

M: Regarding the clubs, for example; I used to be a member in one of the Qatari clubs I was with them for 8 years. In the club there are 30 players; only 3 of them are residents and they ask for 27 Qatari players. For sure it’s so difficult to have 27 Qatari players within the team, so you may in the team 11 players and they don’t come for the training or anything but they come on game’s times. For example daily they arrive to the Qatari’s clubs; a residents young’s or students but the Qatari’s law deterred them from being/ presence in the club and that thing let them to play in the streets or some of them may stop permanently from practicing sport. An evidence for my words; I knew some guys they were they had the ability to reach the Qatari national team because of their strong skills in the sport but because of these laws that stopped them; no *Mash-Allah* they’re over 160kg of weight. Eating and sleeping that’s. The exercising was prevented. So if there was a development in the laws and revised; may be the resident in Doha may be in the Qatari team. It may enhance the sport and would develop it for better, these are the most important.

Male 7: [Group and side talking with laughing] I think that may help people to do more exercises as you knew the depend on the weather [side talking] and the second thing is the campaigns; there are no much campaigns about sport and even if it’s found like the campaigns of Aspire “Your *Step Is Your Health*” the places are limited. They go for gatherings where these itself has no action min 17: 00. [Male 1interruption]: no but regarding that thing by the way I’ve notice something about these events of jogging and Marathon; I noticed they do more of these event and they publish it too. I’ve my little brother *Mash-Allah* he’s 15 years old. He searches the net daily for it. [Male 6: interruption with laughing]: unknown phrases 17:35

M: I stayed with them around 2 years; my problem with them was I gained medals and then I don’t know what happened. They wanted to bring some new players and those new people from the first 2 champions start to get the first places and with time they star to put us away. Even we were going to the training; the coach is focusing on them more than to focus on us.

Male 6: no there will be no restructuring. Let me tell you something. Previously about 6 or 5 years ago there was goalkeeper in the national team till this day we still remember his name , but if till this day if he became the best goalkeeper on the level of Arab world for 6,5 or 10 years the next day he retires nobody will remember his name. If we are the players don’t remember his name would we remember our (unknown word 23:21)… those are the same players who were 40 or 50 years ago they didn’t change and the new one will come… the essential thing of this is not because this is an American system, British system or Italian system or what is so ever the system is. Not because he succeeds in his country that doesn’t mean he will succeed here (unknown word 23:38)…. Find the problems and try to fix it. I really do respect what you are doing now! You are studying the community you live in and try to find solutions. It doesn’t mean let’s bring American system and see its working. No he’s not going to be a world champion no he’s not. [Male 5: interruption: yes right]

Male 6: I have to study what are my abilities. How to solve their problems! Maybe this one doesn’t need more than few things to be world champion; may be this one needs some talk or maybe motivation or maybe just... I know some people who can do anything, anything to a world champion; just give him QR500 and he get anything; he will do anything just to be world champion just give him QR500; no really I swear it became like drugs.

[Male 5: interruption: all of these are under the rules we mentioned at the beginning; is these rules have to be changed…

Interviewer 2: that is what I was talking about, the restructuring of the mangers who are setting these rules!

Male 6: no, it’s not going to be changed

Male 5: the one who sets these rules and if it has to be changed for the best it’s going to be for the benefit of everyone.

Male 6: please say something logic these rules is not going to be changed. [Group talking]

Male 5: let me tell you something; let me tell you something. I’m in the club and within the 8 years from now the rules were changed every sport’s season [Group talking]

Male 6: doesn’t change for the best or for the worst?

Male 5: well it depends on the mood… [Group talking] for example they may change it this season like register 2 players who are born in the State, next season they registered 5… it’s according to the mood. If there is must be a rule it shouldn’t be according to the opinion of administrator. Like terms 1, 2 and 3… they are some country they have the same rules for 30 or 40 years ago they go back for it, it’s like “The Constitution of the State” never change. There must be very strict rules that never change. It shouldn’t be in the hand of administrator who plays with it. So one the rules are set everyone will know what are the things that he do and doesn’t. For example someone came to register in our club we told him sorry come next season for registration check the new rules maybe you may find something for your own good; why this? , because we don’t have a discipline rules [Interruption by Male 1: yes this thing always happened]. If we have discipline rules these rules is not going to be changed. Anything will be clear in front of us. So these are one of the most suggestions we see for the development of sport.

Male1: few minutes ago he asked me why I stopped playing in the Arabic club. Honestly I couldn’t continue because they were some circumstances and so on. Secondly I shift to as I told you the basketball team, so when I found that I quit the building sport. I focused on the basketball *Allhumdellah* I trained more till I became the team captain this year *Allhumdellah* and when I won the champion I took the prize of the best player of the champion. I wasn’t expected this thing to happened because we took the second place in the champion and in the ceremony honoring they said we are going to honoring the best player in the champion; nobody was expected it everyone was expected to be one of those who took the first place in the champion. But it must be to from all the teams. When they called my name my coach didn’t believe it. I just want to tell you not because I stopped playing the building games I didn’t...

Male 5: If the coach is absent the sport class is taken by the other teachers so this is the problem

Male 4: another problem is the attention to football only. I love the basketball but the coach is telling me “my son the basketball has no future either you play football or nothing”. So this makes you frustrated. All the guys play football while am the only one who plays basketball; this is a problem for me. Another thing when a coach or the talent hunters came to choose students, he chooses 1, 2, and 3 and that it is. He came only once; ok; what about us?! Check our skills. Maybe if his performance improved he would be good. But to come and choose only as you like this is not going to work. You made the student to hate the sport.

Male 1: but you can’t blame them because of that. Now when you go for a school and they want to make a school team. They are going to look for the best players in the school [Group talking]

Male 5: you didn’t get his point. He’s talking about the method of selections. For example; I am a teacher. They take only 1, 2, and 3 without paying attention to the rest. Maybe you have a talent that I don’t know it, because I don’t know you personally as a coach and you as a student.

Male 1: just a second! What I am trying to say is when the coach is choosing players for the school team in general. I like to tell you…he goes for the club players because he knows them. Now a school’s coach is about to participate in a champion to present the school. Honestly who is goin26g to choose; you have the club’s players, normal players and players who just love the game that they play it but not that much. Are going to blame the coach why you didn’t choose me. No you can’t blame him [Group talking] let me just finish and then take your time …

Interruption by Male 5: now you play in the university’s basketball team right?

Male 1: yes I do.

Male 5: let us assume that I’m the coach of the basketball’s team and I have a champion I want to participate in it. I came I took A, B, C and D of students because I know them personally that they play sport. I don’t know that you have the skills. I didn’t come to you. I didn’t choose you whether I succeed or I failed. What will happen to you if you knew and I didn’t choose you? You know that you’re better than who did I choose (have personal relationship with them). Would you get sad or not?!

Male 1: yes I’ll get sad if he knew that am ok and didn’t choose me

Male 5: you see! That is what he is trying to tell you. [Group talking]

Male 4: listen the idea is the coach come and pick up only the distinct players only he asks the teacher; where are the distinct players you have? Bring them… [Group talking] The thing is he doesn’t come and talk to you and ask you what do kind of sport you like other than football? No he chooses only football players what about the basketball players. And this teacher who told me that the basketball is not a wide spread sport, because of him I stopped playing it.

M: lack of support from the responsible authority

1. Lack of media support

I: All of you mention factors that prevent you are, lacks of time, family obligation are there other factors prevent you?

I: for example you female3 spoke about mixing are there any other factor?

Female3: maybe no advertising for sport club or the importance of sport s especially for women,

Male 6: when someone comes to me comes in the last day of the month to take his salary and buy a box of KitKat and then go home [laughing] and if you didn’t give him his salary his father will say to “where is his salary” [laughing] and [Male 5: interruption: so the student or the player doesn’t come for salary no] [Group talking] [Male 5: the mentally is materialistic; this mentality must be change].

Male 6: he doesn’t have the mentality of joining the team or if there is an Individual game for example because I want to be a champion or to raise the flag of your country; no they don’t have this mentality; it’s impossible it’s finished. Even their parents used to be like this. They don’t have a goal. They don’t have the loyalty. No, no way!

Male 5: Am talking in a base of he was a player in the swimming national team and he achieved a lot on the level of Arab he so he has the spirt of leadership and according to this he starts to talk about it.

Male 4: another problem is the attention to football only. I love the basketball but the coach is telling me “my son the basketball has no future either you play football or nothing”. So this makes you frustrated. All the guys play football while am the only one who plays basketball; this is a problem for me. Another thing when a coach or the talent hunters came to choose students, he chooses 1, 2, and 3 and that it is. He came only once; ok; what about us?! Check our skills. Maybe if his performance improved he would be good. But to come and choose only as you like this is not going to work. You made the student to hate the sport.

M: if media did not do support to practice sport, people will not practice.

M: media or if the sport is not adopted by the country, unlike country that adopts sport you will see sport machine place automatically in everywhere, at work place, as foreign countries do, or as sport machine which are available at Al -Chornish in Qatar or at Aspire. If the country adopts sports as a project, it will direct people to exercise through media, education

Male 4: Television and social media especially for youth, instead of practicing sports and participating in physical activities they keep using their mobiles on chatting. Chatting on mobiles takes long time from the person without realizing that. These social media create many problems for the people,

1. **Health Factor**

Interruption from f1: when you want to walk my leg will not help me to walk

I1: you mean when you walk what happen you?

F1: I get tired

Interruption: mobile ringing

F1: my knee, I have too much friction

I1: your knee

F1: yes

I1: What are the factors makes difficult to practices sport, things prevent you or stop you to practices sport? You said [f1: pain]

F1: yes, sever knee pain

I1: ok, is there anything else besides knee pain prevent you to practices sport or any movement?

F1: too much effort prevent me, I’m also hypertensive

I1: what?

F1: hypertensive

I1: hypertensive, ok

I1: and you?

F2: from obesity, our legs are not able to tolerate our own body because we get obese

interruption f1: we don’t walk like before, and move, and go out as we used to do

F2: the bad habits of eating makes us obese,

F1: yes that is right.

F2: and with lack of sport body become flabby and fat and stops to move

Interruption: F3: if you have the will you will practices, nothing stops you

Interruption: F1: my daughter eats a lot of chocolate.

F3: The will is the most important thing, if you have that determination you will practice. We do exercise and we eat healthy diet.

F2: our sleeping pattern, our sleeping pattern is the important thing, in gulf our sleeping pattern is not good, we sleep at morning and we stay awake watching television at night not walking and we do nothing.

f1: yes at morning sleeping is not good, keeping awake at night is not good.

F1: these days’ people start eating at night, and start to stay awake at night.

I1: ok

I1: ok

F1: before, after dinner we sleep by 8pm, so person will be sleep comfortable, his body will be comfortable,

F2: true

F1: Now we don’t do that

I1: ok

Female1: some people tell you I can’t because they have joint pain so she can’t do sport, so they make excuses.

F: Also, there are people who cannot do sport as they have pains in their backs or joints pain. It is the opposite of what encourage you

F: Some people have chest pains and all these obstacles,

Nagla: any other obstacles

Female3: the health also as the doctor said

F1: If there is a person( may god protect you) his body is aching or tired, problems with the joints, problems in the stomach or kidneys, you have something that is heavy on you, or obesity, the most thing is the role of obesity. If the person is heavy, he won’t be able to move, she has a tough status unlike the one with a slimmer body which will be easy for him and like to practice exercise it but the obese person doesn’t do exercise.

F: Now, because of joint pain, I cannot move, I have joint pain, disc, the 4th vertebra is weak, this what makes it difficult for me.

F1: For me, my legs cannot help me. Now I cannot walk.

Interviewer1: What cannot help you?

F1: My legs.

Interviewer1: Ok, your health.

F1: yes it cannot help, for walking, No I can’t even walk.

Interviewer2: what is wrong with your legs?

F1: this gets broken and the other got broken too to help her.

[Laugh] wish you speedy recovery.

F1: it got broken from this side the joint, joint wear Hamdulleah.

Interviewer1: God gives you health. How about you, things that act as a barrier to do exercise, you want to do exercise but these things prevent you?

F2: The first thing is pain. For example, I have pain in my right leg, there was a thin bone at the sole of my foot, this was so much, I felt that this was the barrier for me to walk, to move because its pain was so strong although I took an injection in it but the pain is the thing that makes me not move.

Also back pain, pain in general is the thing that makes me sit at home

Interviewer1: And you what makes it difficult on you?

F3: at night tiredness makes me not able to sleep.

Interviewer1: what prevents you?

F3: tiredness, tiredness, at night it doesn’t make me sleep, and I stand to pray *Fajr* (dawn) then I lay down and sleep. Hamduleah, I have nothing, my husband encourages me to go and do this, do exercise but I, I don’t find myself.

Interviewer2: you don’t have the likeness to exercise?

F3: I don’t have the likeness to exercise

Interviewer1: what prevents you?

F4: The barrier is when I feel pain in my legs in the joint and ankle, when the pain increases, I stop movement, but if there is no pain, I work hard and walk.

Interviewer1: the second question; what are the factors that are easier for you to exercise or ant physical activity?

M1: well look. There are so many things that affect you when it comes about exercising. For example; for me they scared me, they told me I have cardiac problem. I had been through prosthetic valve replacement after that I was diagnosed by disc dislodgment; so when I walk for 200-300 meter I start to feel pain in my joints going down till my whole legs so I have to sit for 4-5 min to rest then I can continue walking.

Interviewer1: because of the pain

M1: yes because of the pain

Interviewer1: and who scared you?

M1: no body precisely but it’s the awareness when you are in-patients so many times you get afraid.

Interviewer1: so you mean worry

M1: yes worry [interruption by M3: confusion] if the person has all of this his sons, his wife, his driver and all of it start to make problems so you would be affected too with it

Interviewer1: so according to all of this it may affect being…

M1: yes very much. If you have a troubled son; what to do? Or a girl that is disobeying

M2: interruption: this is the life’s problems.

M1: yes this is life.

Interviewer1: so all of this may prevents you…

M1: it doesn’t prevent, it restricts you...

Interviewer1: you mean make it harder

M1: exactly makes it harder.

Interviewier1: and what are the things that make it easy for you to do exercise?

M1: you know some people are careless Subhan Allah what happened to them doesn’t matter to them and this is difficult, and some of them the simplest thing may have an effect on them emotionally; physically and mentally. While someone just lives only for him-self but who lives for himself, for his sons, his wives will affect the others and get affected too. Salute you; I think I’m innocent so can I go now [said with smile]

Interviewer1: so you think what makes it easy for you to engage in sport that it keeps your health well!?

M1: yes it keeps health well; but in case person has friction knee must walk lightly not to force him-self.

M: They are not thankless but they have their own life, husbands, kids, and my son with his wife and this…The whole idea is about…

Interviewer1: you mean these are the things that eases doing sport for you

M1: no, no he’s saying these are the obstacles. The psychological stress.

I: but what are the obstacles that you might face and would prevent you from practicing exercise?

F2: maybe not having enough time, and sometimes the fatigue and illness.

M: but it is a psychological status.

Interviewer 1: what is the psychological status?

M2: the psychological status is that the person has his work he is busy so it is laziness, job, work, laziness I can’t!

M3: the stresses around us

M2: the stresses around us

M: there are a lot of factors, the psychological factors, psychological stress at work,

M: if one has diseases that prevents him from walking some diseases one will not able to walk, so he can’t do sports so it is beyond his well, he needs to do sports but he can’t, highly obese but can’t do sports.

Interviewer2: life’s concerns!

F3: yes for me for example I have problem in my knees which I can’t do anything so easily.

Interviewer2: so for you the knee’s problem is considered as obstacle for you! [Side talking and laughing by the participants]

Interviewer 1: psychological factor. Can you explain more?

F2: for example psychologically when you feel you are psychologically stressed you don’t feel you have the desire to do sports,

F2: it’s not... I mean depression, --- you are in depression specifically, you don’t feel you have the desire to do sports or other than sports or anything. Some people get depressed they would eat, other people when they get depressed they don’t have the desire to eat,

[Interruption]

F2: you see there is opposite things, some people get depressed they would eat, some people when they get depressed they lose their appetite.

Interviewer 1: so for you physiological stress prevents you from doing sports

F2: sometimes. Sometime. And sometimes I try to overcome them.

Interviewer 2: how the psychological status ------

F2: enough you feel enough, that your bound, depressed that’s it you are bound you don’t have desire, your desire don’t ask even for sports, as I told you now you have the tendency enough you have depression you don’t have the desire to eat, some people (khalas) their appetite for everything is gone.

Interviewer 1: okay. What are factors makes it difficult for you to do sports?

F1: makes it difficult?

Interviewer 1: yes

F1: sometimes social condition

Interviewer 1: you mean by it?

F1: I mean stresses inside the house,

Interviewer 1: and you? What prevents you from doing sports? You have said some things but I want to... Because there points in the middle I want you to say them again

F2: I told you the most that would prevent me if I was psychologically stressed and this is the most that would make me not do sports… as long as I am relaxed psychologically

F4: besides obesity increased highly in children; all the time setting watching TV

F2: imagine, if you went to school how many child you will find obese

M: unless he was psychologically ill

M: maybe the thing that could prevent me it’s only sickness

M: I will say again the psychological factor

Female 1: The house is not hot, there is air condition. The obstacle from doing the exercises is the sickness.

M: we don’t do exercise now, I’m using walking stick to walk, how do think I can practice?

M: your wife sick

M:Maybe you have back pain, or have something, that’s prevents you to practice,

Female 1: when I get sick.

F: God decide when the human got sick he does not feel the value what you have or health until he found himself tired and found another human enjoying the vitality and energy and going back and force Mash-Allah

F:I remembered when I was pregnant and I can’t move the doctor told me not to move because I had bleeding, and in the same time high cholesterol and sugar. The bleeding does not need to move and the sugar and the cholesterol need movement to lower its level in blood... I used to see when I was following Mash-Allah no power than the God Mash-Allahno power than the God people you can tell that they are older in age and running. There is one lady she was pregnant may in her 8 months and running Mash-Allah. I felt that am younger than her in age and I can’t. I suppose to move so that I can reduce the sugar and reduce the cholesterol. In the same time I don’t eat much and the physiological state increased this.

Female 6: the physiological state is the difficult thing.

Female 5: I can’t move because the movement is bad to baby

M: the factor that prevents me from playing sport is the injuries factor. There are some injuries that are very bad in a way you even can’t continue

M: your psychological condition will gets tired because of…

M: Psychologically it’s a factor in human that helps the person.

M:And the exercise is something know; the effect of the right sport that the one must put a schedule to follow not according to the mood. So if we’re under pressure because of study the one is facing certain difficulties in doing sport.

M: the person will not do exercise unless he faces sickness

Male 1: said if the person is old and has pain it become difficult for him to practice physical activity, but if the person is ready to practice he will do it even he is young or old. If the person is old he cannot. Most of us would like to walk , For example I am diabetic and if I have the ability to walk for 1-2 kilometers daily I know that my blood sugar level will drop, no doubt, but at evening I may have knee pain because I walked in the morning.

Male 3: There is one main and basic factor which is diabetes. Diabetes motivates me to practice and continue practicing physical activity compulsory not optional. There was a park here in Al Rayyan, but it was removed make it difficult for us to practice physical activity and this make us not practicing at all.

M:There is Increase in the number of the people who have diabetes here in the Gulf is due to lacking of physical activity and sleep

Male 1: There are two types of barriers to practice physical activities; the first one is from the person himself, weakness, aging, or the effect of his body.

M: For example my father lives long time in a good health, but now people reach the age of forty and have many diseases. . In addition the nature of the jobs now require prolonged sitting on the desk in front of the computer. . In addition the nature of the jobs now require prolonged sitting on the desk in front of the computer. People become dependent

1. Mental health

Interviewer1: so you mean worry

M1: yes worry [interruption by M3: confusion] if the person has all of this his sons, his wife, his driver and all of it start to make problems so you would be affected too with it

Interviewer1: so according to all of this it may affect being…

M1: yes very much. If you have a troubled son; what to do? Or a girl that is disobeying

M2: interruption: this is the life’s problems.

M1: yes this is life.

Interviewer1: so all of this may prevents you…

M1: it doesn’t prevent, it restricts you...

Interviewer1: you mean make it harder

M1: exactly makes it harder.

Interviewier1: and what are the things that make it easy for you to do exercise?

M1: you know some people are careless Subhan Allah what happened to them doesn’t matter to them and this is difficult, and some of them the simplest thing may have an effect on them emotionally; physically and mentally. While someone just lives only for him-self but who lives for himself, for his sons, his wives will affect the others and get affected too. Salute you; I think I’m innocent so can I go now [said with smile]

M: They are not thankless but they have their own life, husbands, kids, and my son with his wife and this…The whole idea is about…

Interviewer1: you mean these are the things that eases doing sport for you

M1: no, no he’s saying these are the obstacles. The psychological stress.

M:but it is a psychological status.

Interviewer 1: what is the psychological status?

M2: the psychological status is that the person has his work he is busy so it is laziness, job, work, laziness I can’t!

M3: the stresses around us

M2: the stresses around us

M: there are a lot of factors, the psychological factors, psychological stress at work,

Interviewer 1: psychological factor. Can you explain more?

F2: for example psychologically when you feel you are psychologically stressed you don’t feel you have the desire to do sports,

F2: it’s not... I mean depression, --- you are in depression specifically, you don’t feel you have the desire to do sports or other than sports or anything. Some people get depressed they would eat, other people when they get depressed they don’t have the desire to eat,

[Interruption]

F2: you see there is opposite things, some people get depressed they would eat, some people when they get depressed they lose their appetite.

Interviewer 1: so for you physiological stress prevents you from doing sports

F2: sometimes. Sometime. And sometimes I try to overcome them.

Interviewer 2: how the psychological status ------

F2: enough you feel enough, that your bound, depressed that’s it you are bound you don’t have desire, your desire don’t ask even for sports, as I told you now you have the tendency enough you have depression you don’t have the desire to eat, some people (khalas) their appetite for everything is gone.

Interviewer 1: okay. What are factors makes it difficult for you to do sports?

F1: makes it difficult?

Interviewer 1: yes

F1: sometimes social condition

Interviewer 1: you mean by it?

F1: I mean stresses inside the house,

Interviewer 1: and you? What prevents you from doing sports? You have said some things but I want to... Because there points in the middle I want you to say them again

F2: I told you the most that would prevent me if I was psychologically stressed and this is the most that would make me not do sports… as long as I am relaxed psychologically

M: unless he was psychologically ill

M:I will say again the psychological factor

F: In the same time I don’t eat much and the physiological state increased this.

Female 6: the physiological state is the difficult thing.

M: your psychological condition will gets tired because of…

M: Psychologically it’s a factor in human that helps the person.

M: And the exercise is something know; the effect of the right sport that the one must put a schedule to follow not according to the mood. So if we’re under pressure because of study the one is facing certain difficulties in doing sport.

- Mental Health- Presence of depression

F2: it’s not... I mean depression, --- you are in depression specifically, you don’t feel you have the desire to do sports or other than sports or anything. Some people get depressed they would eat, other people when they get depressed they don’t have the desire to eat,

[Interruption]

F2: you see there is opposite things, some people get depressed they would eat, some people when they get depressed they lose their appetite.

Interviewer 2: how the psychological status ------

F2: enough you feel enough, that your bound, depressed that’s it you are bound you don’t have desire, your desire don’t ask even for sports, as I told you now you have the tendency enough you have depression you don’t have the desire to eat, some people (khalas) their appetite for everything is gone.

1. Presences of diseases

M1: well look. There are so many things that affect you when it comes about exercising. For example; for me they scared me, they told me I have cardiac problem. I had been through prosthetic valve replacement after that I was diagnosed by disc dislodgment; so when I walk for 200-300 meter I start to feel pain in my joints going down till my whole legs so I have to sit for 4-5 min to rest then I can continue walking.

Interviewer2: life’s concerns!

F3: yes for me for example I have problem in my knees which I can’t do anything so easily.

Interviewer2: so for you the knee’s problem is considered as obstacle for you! [Side talking and laughing by the participants]

F2: it’s not... I mean depression, --- you are in depression specifically, you don’t feel you have the desire to do sports or other than sports or anything. Some people get depressed they would eat, other people when they get depressed they don’t have the desire to eat,

[Interruption]

F2: you see there is opposite things, some people get depressed they would eat, some people when they get depressed they lose their appetite.

Female 1: when I get sick.

Male 1: said if the person is old and has pain it become difficult for him to practice physical activity, but if the person is ready to practice he will do it even he is young or old. If the person is old he cannot. Most of us would like to walk , For example I am diabetic and if I have the ability to walk for 1-2 kilometers daily I know that my blood sugar level will drop, no doubt, but at evening I may have knee pain because I walked in the morning.

Male 3: There is one main and basic factor which is diabetes. Diabetes motivates me to practice and continue practicing physical activity compulsory not optional. There was a park here in Al Rayyan, but it was removed make it difficult for us to practice physical activity and this make us not practicing at all.

M: There is Increase in the number of the people who have diabetes here in the Gulf is due to lacking of physical activity and sleep

Male 1: There are two types of barriers to practice physical activities; the first one is from the person himself, weakness, aging, or the effect of his body.

M: For example my father lives long time in a good health, but now people reach the age of forty and have many diseases. . In addition the nature of the jobs now require prolonged sitting on the desk in front of the computer. . In addition the nature of the jobs now require prolonged sitting on the desk in front of the computer. People become dependent

1. Obesity factor

I1: and you?

F2: from obesity, our legs are not able to tolerate our own body because we get obese

interruption f1: we don’t walk like before, and move, and go out as we used to do

F2: the bad habits of eating makes us obese,

F1: yes that is right.

F2: and with lack of sport body become flabby and fat and stops to move

Interruption: F3: if you have the will you will practices, nothing stops you

Interruption: F1: my daughter eats a lot of chocolate.

F3: The will is the most important thing, if you have that determination you will practice. We do exercise and we eat healthy diet.

F: Some overweight people will find it so difficult to do sport.

F1: If there is a person( may god protect you) his body is aching or tired, problems with the joints, problems in the stomach or kidneys, you have something that is heavy on you, or obesity, the most thing is the role of obesity. If the person is heavy, he won’t be able to move, she has a tough status unlike the one with a slimmer body which will be easy for him and like to practice exercise it but the obese person doesn’t do exercise.

M: if one has diseases that prevents him from walking some diseases one will not able to walk, so he can’t do sports so it is beyond his well, he needs to do sports but he can’t, highly obese but can’t do sports.

F4: besides obesity increased highly in children; all the time setting watching TV

F2: imagine, if you went to school how many child you will find obese

1. Lack of diseases

F: God decide when the human got sick he does not feel the value what you have or health until he found himself tired and found another human enjoying the vitality and energy and going back and force Mash-Allah

M?: the person will not do exercise unless he faces sickness

1. Pregnancy

Female 5: I can’t move because the movement is bad to baby

F: I remembered when I was pregnant and I can’t move the doctor told me not to move because I had bleeding, and in the same time high cholesterol and sugar. The bleeding does not need to move and the sugar and the cholesterol need movement to lower its level in blood... I used to see when I was following Mash-Allah no power than the God Mash-Allahno power than the God people you can tell that they are older in age and running. There is one lady she was pregnant may in her 8 months and running Mash-Allah. I felt that am younger than her in age and I can’t. I suppose to move so that I can reduce the sugar and reduce the cholesterol. In the same time I don’t eat much and the physiological state increased this.

- **Factors that makes it easy for you to engage in physical activity**

| **No.** | **Factors that makes it easy for you to engage in physical activity** | | **References** |
| --- | --- | --- | --- |
|  | **Health factor** | 1. Obesity factor (25) 2. Presences of diseases(17) 3. Mental health(11) 4. Lack of diseases(8) | **130** |
|  | **Personal Beliefs and Attitudes** | 1. Presence of will (43) 2. Desire to exercise (16) 3. Feeling tired (5) 4. Cultural diversity (3) 5. Elderly perception (2) 6. Exercise outcome (1) | **90** |
|  | **Having Support** | 1. Group support (45) 2. Role of Religion (24) 3. Family support (10) 4. Role of physiotherapy (3) 5. Financial Factor (1) | **71** |
|  | **Environment** | 1. Physical environment (49) 2. Natural environment (30) | **66** |
|  | **Time factor** | | **39** |
|  | **Awareness** | | **9** |

1. **Heath factor**

M3: what encourages me?

Interviewer 1: yes

M3: to activate the blood circulation and health

F1: health first, exercise for me equals health because sitting at home all day without any movement is not good for health.

F: some people would do sports to lose weight some people do it to increase weight it depends.

F2: if I was obese I would do sports to maintain my body and the weight. That’s it.

F4: I see that it is a psychological factor, when I do it I psychologically relax, and my mind becomes active and in the same time organizes my time.

F: reduces weight

F: organizes my sleeping as well.

F1: Wallahi I feel we need sports in an abnormal way seriously, it helps us specially in studying, that it benefits the mind at once. At once you would feel the difference if you did sport!

F2: that’s right I have read information about the brain that the harder the training is, the harder the training is the thinking will increase, brain cells would increase.

F2: in growth. Even if you change the hand you use the brain will grow.

F3: to be healthy,

F: mostly we take exercise to diet or reduce weight or that I can be healthy as prevention from disease.

F5: for me, when I’m nervous

F: I practice different exercise, and these days we focus on shape to be appropriate and to maintain health.

Male3: strengthen the body and the muscles

Male4: to build up a better body and maintain the body’s health,

Male7: I think the social pressure upon man in the body or in a certain shape as a certain body health that will make pressure on the man to do sport.

Samira: fitness?

Male7: yes, at least to be fit. It is not even important to have the maximum fitness, but at least to be sport and maintain his body and maintain his body organs in a healthy way.

M: gym alone is not alone enough, those who go gym focus on body building and we want fitness to go out any play something. That is it for exercise.

M: Thirdly when there is a reason that makes you like when you are obese or diseased, this can motivates you.

I: Explain what do you mean outcome?

M4: the outcome is why sport? To live good, comfortable, maybe prolong life that’s should biggest motive and the main thing.

I1: what makes for easy to practices physical activity?

F1: what?

I1: what easy to do physical activity, or any movement?

F1: walking, movement [f1 is moving her hand]

I1: moving your hand

I1: what makes on you easy to practices sport?

F1: it’s for my health and for normal activity

I1: so normal activity and health make easy for you to engage in physical activity?

F1: yes

I1: ok, and what makes easy for you to practices sport?

F2: running, [laughing], of course we don’t run, if we are running we would not be obese (F2 mean her weight)

F1: I’m with you

F2: running, jumping,

F1: also the legs are not helping.

Interruption: F1: person can’t walk, even at home, from pain due to friction in the knees I can’t walk.

I1: from the pain

F1: even when I walk at home I get tired when I walk at kitchen, because of knee problem, they told me I need to do operation, but I refuse to do it, I will look for treatment Inshallah [God willing]

F: Secondly, if you want to lose weight. A lot of people want to lose weight

Female1: factor of obesity, so they should practice exercise

F: I know about diabetic and hypertensive people when we advise them to reduce the sugar and do sport because this will reduce risk from heart diseases.

Nagla: you mean presence of a disease would encourage in doing sport?

Female1: yes, it should, it should encourage them.

Nagla: what are the factors motivate you to do sport?

Female: avoiding disease, because they advise to walk to reduce the cholesterol. They advise me a lot to walk and thanks God I started walking.

Female3: if there are sickness first,

F1: before my body got broken (ache), I used to play exercise, which was normal and what helped me is that I was lighter in my weight.

M3: well look; nothing is better than the physical activity. First it makes your body and muscles relaxed; conversely it reliefs the stress. I think walking is a good thing it’s kind of sport.

Interviewer1: so you think what makes it easy for you to engage in sport that it keeps your health well!?

M1: yes it keeps health well;

Interviewer: Now, can you please tell me about things that make it easy for you to engage in physical activity and why those things make it easy for you to engage in physical activity?

Male 1: to have good health. The health is the biggest motivation, if he likes sport it considered as a hobby. For the playing the football and running is a hobby, but walking is for human’s health.

F2: it could be the desire of a healthy lifestyle or to change the routine and there is even programs for people who have insomnia for example or unbalanced hormones they can practice exercise firstly some programs, the person with insomnia he becomes fatigue so he has to practice exercise and this would make the sleeping quality in the night better.

F4: it could be because of sickness they call it this activity would treat the disease, from this disease... The activity would treat this thing

F5: or if person finds himself very fat they try to lose weight by practicing exercise

M: it is perseverance like praying, there should be everyday half an hour that I do sports, and after he does the sports he would see the result of the sports on him; on his sleep on his movement on his shape on his health so when he sees the good results on him he would commit to it more.

M: practicing and then seeing the result of sports on him; on his health on his shape on his eating or the developments he has so he would hang to it more. That is it. It is a process of getting used to it.

Interviewer1: ok what the factors that help you to do exercise; things that encourages you?

Interviewer2: what encourages or motives you to do exercise?

F1: when I feel I am tired, or my legs are or something like that.

Interviewer2: you get motived!

F1: yes like sitting for a long time makes you feel like you have to do some exercise.

F2: yes if we felt like we are tired of siting a long time it’s better for us to go and some exercise. We don’t like to sit.

F3: for me! It’s being healthy.

F: Doing exercise makes you feel happy little bit. You get pleasant. It changes your mood.

F: The second thing is when you do exercise you feel you’re much active than sitting and your depressed feeling; these entire things go away with doing exercise.

F1: also you feel comfortable; you feel good and relaxed. If you walked you feel ok. You feel your body is relaxed. Your body circulation is getting better.

F2: the psychological factor.

Interviewer 1: psychological factor. Can you explain more?

F2: for example psychologically when you feel you are psychologically stressed you don’t feel you have the desire to do sports, also weight loss, the more weight lost this makes me more energetic, and there would be more energy, I mean -------- [laughs] of course there will be more tendency to do sports. The more the weight loss the more tendency to do sports.

Interviewer 1: What makes it easy for you? That you do sports? What encourages you?

F3: losing weight, but she said when one is stressed, for example when I am stressed I do sports to vent the charges I have, meaning with sports and if I am annoyed and like this I would burn [laughs]

Interviewer 1: so for you when you are stressed you do sports

F3: yes, I would relax.

F2: it’s not... I mean depression, --- you are in depression specifically, you don’t feel you have the desire to do sports or other than sports or anything.

Interviewer 1: so for you physiological stress prevents you from doing sports

F2: sometimes. Sometime. And sometimes I try to overcome them.

F: Sports are very important for the human. In all aspects. It is movement for blood circulation, it would be the blood circulation is okay especially in these times, people are suffering more of diabetes, of high blood pressure, and for sure sports and walking are the best cure for everything for all these diseases.

F: So it is very important for the heart for diabetes for lots of things

F2: for health generally

F4: see when you move you spend this energy and you distribute it to the whole body

F2: that is right, you are talking about movement; setting on TV or devices

M1: before I started doing sports, my weight was (Mashalla) over 120 Kgs, I reached this age, the age of 50 I found myself getting tired getting tired I started walking and doing sports the last thing I was practicing walking sports I used to walk around an hour, an hour and quarter till I reduced my weight and became active and this is what made me practice sports.

M: the person’s psychology

M1: in the first place my health is the most important. So I care about my health. I walk as much as I can and taking care of my diet also being active not lazy. I see my future in my health. If lost my health then I‘ll lose my future. That what push me to be a healthy person; *Alhamdulillah Rab El-almeen* (Thanks God) *Subhano o Taa’lah;* who created me and made me Muslim and gifted me with (asked to pray) 5 prayers daily. Through the prayer I feel am very active. With God’s bless I pray my *Al-Fajor* at time (dawn prayer) all these are activates I do. Being able to walk to the mosque; Al-Roqua’ and AL-Sojoud (Prayer movement) all these *Alhamdulillah.* Our Islam invited us for theses prayers to be good so I would help in constructing earth

M3: exercise is health. Healthy for body; active of the mind and thinking.

M3: if the person is relaxed psychologically he can practice exercise,

M: if the person is a bit obese he can practice exercise.

M3: some people are diabetic and they practices sport to maintain and decrease blood sugar

M3: to be psychologically relaxed may be the nature of work, home, to be relaxed at home,

F: a lot of factors affect the person and his psychological relaxation.

Interviewer1: ok do you have something else you want to add about sport?

F2: well if you became fat a bit you can do some exercise at home

Interviewer1: you mean there is no way to do it?

F1: you mean now… No I am pregnant we are not free.

F2: Yes if we gained weight a bit we do exercise at home on the machines.

Interviewer1: ok what the factors that encourages you to do exercise?

F1: from me the moment I feel am getting fat I start to do exercise. [Side talking]

F2: just like her; I don’t want to be fat. Fear; you get fear from people around you and from disease *I seek Allah forgiveness*

F1: yes she is right especially we have a genetic disease for example I’m married to my cousin and he was obese in the beginning. He did an operation to lose weight. Since that time everything at home start to be healthy, our children and our food so once I feel I am getting fat, he starts to ha ha [making sound as she trying to explain how she has to lose weight] and now I’m pregnant and that is a chance for me to eat what I want.

Interviewer1: and you what are the factors that encourage you to do sport?

F3: mostly when I’m upset. When I’m bored I do exercise. Really it makes me feel good. This is the only thing.

Interviewer1: what are the factors that encourage you to play sport?

F4: to be healthy and not to be fat. It’s useful for me at the end not to harm me. What doesn’t harm you don’t be useful for you [laughing]

Female 4: I walk when I am upset so I get released this is the first point

F: The second point I walk because I have high cholesterol. I walk to help my health.

F3: for example one who has problems with weight or she has health problems and has back pain or in the leg. These are the things that we advise them to walk and it is according the condition.

F: When I am over weight

F3: if I am over weight.

F4: if I have extra weight or when I feel I am little bit tired, so I try to move, I try eliminating the tiredness from my body. The movement will help me and makes me more active

F1: I feel am active but I walk only when I have thing to think about. I like to walk for a long time. To find a solution, I walk

F1: the health. When I have elevated blood sugar, I like to walk.

M2: and you have to eat food before walking if the person was diabetic to avoid hypotension, at least he have to eat a light meal before walking.

M4***: ( Gazak Allah kheera)*** God reward you, another point person should not complain too much even though he is sick or tired, he should say ***Alhamdulillah*** “thank god” I’m healthy,

M: you will be that time psychologically active

Female 1: liveliness.

Female 2: to eat healthy all time because the fat causes diseases. After growing up we figure out that the fats cause diseases and cholesterol so it’s better to eat healthy diet.

Female 1: have an ideal body and to feel the fitness. This increased the determination in the person since am the one that go and I want thins determination to increase not decreased.

Female 3: I want to reduce my weight. This is going to encourage me.

Female 2: when someone gets fat.

M: it became a lifestyle every one play sports especially guys care about muscles.; he cares about his body; his shape of the muscles.

Male 3: keeping the own health well. The sport sometimes prevents the one from un-necessary diseases.

M: And the weight it helps him to maintain his weight.

Male 5: the fear of diabetic illness because this is widespread disease and the one must try to avoid these diseases by doing sport so that is what encourage me to do sport regardless the person maintains his weight. He keeps well health

M: “a sound mind in a sound body”

Male 7: the willing for losing weight it’s the biggest motivation

M:work duty will be acceptable not exhausted

M: to have power to do extra effort

M: Only one thing is being afraid from sickness, and if the doctor tells you going to die tomorrow

Male 2: when you are at the border line to be diabetic, hypertension, going for anything, that time you will push yourself to do and after that no.

Male 2: for me yes, it’s one of the reasons. In fact Arabic person should be at the edge of danger to practice sport. When the doctor informed me that I became obese, gained weight and you might have so and so, I ran for a while but once I forget doctor words I stopped running

Male 1: The power. Everyone has power encourage him to practice physical activity. When he is fair enough and has a power; this encourages him to practice physical activity. However, if he does not have power he will not have intention to practice activities, he cannot practice even if he wants, but he cannot. When the person is in a good health or feel satiety, he cannot practice physical activity easily. In addition, when the person is aged and unable to practice physical activity; it become against his wishes.

Male 1: yes both health and power encourage practicing physical activity.

M: the first one is movement and the second one is controlling the diet. Also the person should avoid two things, sugar and fat; these things the person should fight them. Moreover there are two other things that adversely affects the body, two white things are adversity for the body, salt and sugar.

Male 2: To maintain the health and as I said before it consider as the first line of defence against diseases. You can also continue and survive as long as in a good health condition.

M: He practices sports to maintain his health and protect his body from diseases such as diabetes,

1. Obesity factor

F: some people would do sports to lose weight some people do it to increase weight it depends.

F2: if I was obese I would do sports to maintain my body and the weight. That’s it.

F:reduces weight

F: mostly we take exercise to diet or reduce weight

M: Thirdly when there is a reason that makes you like when you are obese

F2: running, [laughing], of course we don’t run, if we are running we would not be obese (F2 mean her weight)

F: Secondly, if you want to lose weight. A lot of people want to lose weight

Female1: factor of obesity, so they should practice exercise

F5: or if person finds himself very fat they try to lose weight by practicing exercise

F: also weight loss, the more weight lost this makes me more energetic, and there would be more energy, I mean -------- [laughs] of course there will be more tendency to do sports. The more the weight loss the more tendency to do sports

Interviewer 1: What makes it easy for you? That you do sports? What encourages you?

F3: losing weight,

M1: before I started doing sports, my weight was (Mashalla) over 120 Kgs, I reached this age, the age of 50 I found myself getting tired getting tired I started walking and doing sports the last thing I was practicing walking sports I used to walk around an hour, an hour and quarter till I reduced my weight and became active and this is what made me practice sports.

M: if the person is a bit obese he can practice exercise.

Interviewer1: ok do you have something else you want to add about sport?

F2: well if you became fat a bit you can do some exercise at home

F2: Yes if we gained weight a bit we do exercise at home on the machines.

Interviewer1: ok what the factors that encourages you to do exercise?

F1: from me the moment I feel am getting fat I start to do exercise. [Side talking]

F2: just like her; I don’t want to be fat. Fear; you get fear from people around you and from disease *I seek Allah forgiveness*

Interviewer1: what are the factors that encourage you to play sport?

F4: to be healthy and not to be fat

F3: for example one who has problems with weight

F: When I am over weight

F3: if I am over weight.

F4: if I have extra weight or when I feel I am little bit tired, so I try to move

Female 3: I want to reduce my weight. This is going to encourage me.

Female 2: when someone gets fat.

M: And the weight it helps him to maintain his weight.

Male 7: the willing for losing weight it’s the biggest motivation

M: When the doctor informed me that I became obese, gained weight and you might have so and so, I ran for a while but once I forget doctor words I stopped running.

1. Presences of diseases

F: or that I can be healthy as prevention from disease.

M: or diseased,

F1: also the legs are not helping.

Interruption: F1: person can’t walk, even at home, from pain due to friction in the knees I can’t walk.

I1: from the pain

F1: even when I walk at home I get tired when I walk at kitchen, because of knee problem, they told me I need to do operation, but I refuse to do it, I will look for treatment Inshallah [God willing]

F: I know about diabetic and hypertensive people when we advise them to reduce the sugar and do sport because this will reduce risk from heart diseases.

Female3: if there are sickness first,

F2: it could be the desire of a healthy lifestyle or to change the routine and there is even programs for people who have insomnia for example or unbalanced hormones they can practice exercise firstly some programs, the person with insomnia he becomes fatigue so he has to practice exercise and this would make the sleeping quality in the night better.

F4: it could be because of sickness they call it this activity would treat the disease, from this disease... The activity would treat this thing

Interviewer1: ok what the factors that help you to do exercise; things that encourages you?

Interviewer2: what encourages or motives you to do exercise?

F1: when I feel I am tired, or my legs are or something like that.

Interviewer2: you get motived!

F: times, people are suffering more of diabetes, of high blood pressure, and for sure sports and walking are the best cure for everything for all these diseases

F: So it is very important for the heart for diabetes for lots of things

M3: some people are diabetic and they practices sport to maintain and decrease blood sugar

F: The second point I walk because I have high cholesterol. I walk to help my health.

F: she has health problems and has back pain or in the leg. These are the things that we advise them to walk and it is according the condition

F1: the health. When I have elevated blood sugar, I like to walk.

Male 2: when you are at the border line to be diabetic, hypertension, going for anything, that time you will push yourself to do and after that no.

Male 2: for me yes, it’s one of the reasons. In fact Arabic person should be at the edge of danger to practice sport

1. Mental health

F: Doing exercise makes you feel happy little bit. You get pleasant. It changes your mood.

F: The second thing is when you do exercise you feel you’re much active than sitting and your depressed feeling; these entire things go away with doing exercise.

F2: the psychological factor.

Interviewer 1: psychological factor. Can you explain more?

F2: for example psychologically when you feel you are psychologically stressed you don’t feel you have the desire to do sports,

F: but she said when one is stressed, for example when I am stressed I do sports to vent the charges I have, meaning with sports and if I am annoyed and like this I would burn [laughs]

M: the person’s psychology

M: It’s a psychological relief. It’s much better than Yoga or music or the wine that others drink to find relief.

M3: if the person is relaxed psychologically he can practice exercise,

Interviewer1: and you what are the factors that encourage you to do sport?

F3: mostly when I’m upset. When I’m bored I do exercise. Really it makes me feel good. This is the only thing.

Female 4: I walk when I am upset so I get released this is the first point

M: you will be that time psychologically active

M: “a sound mind in a sound body”

1. Lack of diseases

Nagla: you mean presence of a disease would encourage in doing sport?

Female1: yes, it should, it should encourage them.

Nagla: what are the factors motivate you to do sport?

Female: avoiding disease, because they advise to walk to reduce the cholesterol. They advise me a lot to walk and thanks God I started walking

Interviewer1: How about yourself, when I ask you to do exercise, what else when things are available would make it easy for you to practice exercise?

F1: before my body got broken (ache), I used to play exercise, which was normal and what helped me is that I was lighter in my weight.

Male 3: keeping the own health well. The sport sometimes prevents the one from un-necessary diseases.

Male 5: the fear of diabetic illness because this is widespread disease and the one must try to avoid these diseases by doing sport so that is what encourage me to do sport regardless the person maintains his weight. He keeps well health

M: Only one thing is being afraid from sickness, and if the doctor tells you going to die tomorrow

Male 2: To maintain the health and as I said before it consider as the first line of defence against diseases. You can also continue and survive as long as in a good health condition.

M: He practices sports to maintain his health and protect his body from diseases such as diabetes,

1. **Personal Beliefs and Attitudes**

Interviewer 1: yes to activate the blood cycle and health?

M3: even more to entertain yourself.

F3: if I felt my body was inactive and for example I want to activate my body and my mind I do sports or I busy myself with other things like home chores.

F5: yeah. It can be for entertaining,

F: I see that sports organized

F1: Wallahi I feel we need sports in an abnormal way seriously, it helps us specially in studying, that it benefits the mind at once. At once you would feel the difference if you did sport!

F1: for me the laziness is motives me to do physical activity, because laziness is a bad feeling, but it can vary from one person to another

F2: When I see outcome of exercise on the other people, I look forward to be like them, I like the outcome on them

I1: why? The reason when you see any person outcome what do feel?

F2: the ambition to be like them

F4: sometimes the motive is being Jealous like why these people have nice body, I will want my body to be nice to be like them, or using my extra time that I have and do something that will benefit me like for example exercise.

Male1: motivate us? For example the body build. Motivate us...your look in front of the people. When the people say you practice, you train and what is your schedule. You feel your became trainer and everyone ask you. *Ma Sha Allah* you have changed.

M: Keep practicing a certain sport , not to eat much and say I will do sport because that will lead to be lazy

Male4: to build up a better body and maintain the body’s health, even for your reputation in front of the people, you will have an especial position.

Male7: I think the social pressure upon man in the body or in a certain shape as a certain body health that will make pressure on the man to do sport.

M: It is not necessary to reach the full muscle shape, no, at least to have sport shape body

M: Fourth, the exercise should be early in the morning instead of at night because in the morning the body is more active.

M1: Ok, what else from the exercise view, the person can focus on the thing he likes more either football, basketball, swimming, running and he should try to mix, not only one thing, gym alone is not alone enough, those who go gym focus on body building and we want fitness to go out any play something. That is it for exercise.

M3: the most important thing is the joy, to feel joy when you are doing the activity.

M5: in my opinion I will talk about this country or most of the countries nowadays, In Qatar, there are gyms, sports clubs, there are many things, and there are trainers and expertise from all over the world especially the main clubs like *Aspire,* those places that are well known and of a great place in the country, they bring the best people there and not anyone. So for myself, If I don’t like to go or don’t have a goal to make my body look like this or be fit enough so when I am placed in a situation I would be able to react. If I don’t have the goal that I want, I will not go to any club. For myself, If is essential to have the goal to have the body and fit, not only shape, there should be fitness in the end.

M2: i think….. you will have the motivation, if your family and friends, if all of them practice sport, i will have the motive to practice more, i will try to give more or to be at same level they are or more.

F: and environment surround me how it’s ,my life at home how its , at university and outside the university , the environments surround me should motivate me to do exercise .

F: Society's perception and the children, you are old now why do you want to exercise, the Society's perception they don’t have that open sight so if the person run or jog in the street that it’s a normal thing, people from society would say see how silly she is running at street, Society's perception.

F: Of course the western countries takes this sport dosage and everyone walks in *Cornich* and walk in the gardens. Everyone has specific hours that they should… and I read about *Condoleezza Rice* who was the foreign minister and security in charge. She has 3 hours that she spends only in exercise. This is very; very; very important

Female1: it is about what you like to do for example: it is you if you have the will to do sport so you will go and do your sport. This is the first thing.

F: The weather shouldn’t be considered as a factor, it should not stop us.

Interviewer2: So the swimming pool in the physiotherapy was one of the facilitators.

F1: Yes so much.

Interviewer2: Joint pain, and…

F1: you will not feel anything when you enter the pool as if you were lying about the sickness you had.

[Laugh]

F1: you will say you are a liar because the water is hot which will make the joints more flexible and you will not feel but when you go out in the air conditioner, you will lie if you said you are still awake.

Interviewer1: and you what are the factors that make it easy for you to practice exercise?

F2: the first thing is the weather.

Interviewer1: The weather.

F2: The weather should be nice and this is very important so I can go out and walk. I am that kind of person where weather affects me, the sun, I cannot walk under the sun, or the increased hotness. When the weather becomes nicer, I can go out in the compound and walk for example.

F3: I walk at home *walla* and do this but I have mental issue in which I feel all tired, I don’t go out, I feel…I feel I am not in the mood.

Interviewer2: this tiredness that prevents you, is there anything that helps you to move?

F3: *Hamdulleah*, when I facilitate my things and adjust it, when I am little tired, I try to facilitate things by myself.

M: I have responsibilities, house and children but I can devote some time for that, I can devote some time to practice exercise. At night, I can practice exercise after I put her

M: the awareness when you are in-patients so many times you get afraid.

Interviewer1: and you sir! What makes it easy for you to do sport or physical activity?

M3: well look; nothing is better than the physical activity. First it makes your body and muscles relaxed; conversely it reliefs the stress. I think walking is a good thing it’s kind of sport.

M2: conversely I love sport, I do love sport and I play football. What can I say to you I play even with people age 15 yrs old, I play with them really. [M3 interruption: it’s ok to do so. It’s just a sport; it is for every one whether young or old.

Interviewer1: ok and you?

M3: In fact I’m a sport person

Interviewer1: yeah what eases things for you?

M3: I stopped doing sport as practice but as walking I park my car far away and I always love to walk. This walking is a hoppy.

F: is a good way of spending time and I recommend it.

F2: it could be the desire of a healthy lifestyle or to change the routine and there is even programs for people who have insomnia for example or unbalanced hormones they can practice exercise firstly some programs, the person with insomnia he becomes fatigue so he has to practice exercise and this would make the sleeping quality in the night better.

M2: so if the time was suitable I can do my work, we now stick only to watching a football match watch a match like that… but to do physical activity I think we need time and some morals (meaning high spirits)

M: it is perseverance like praying, there should be everyday half an hour that I do sports, and after he does the sports he would see the result of the sports on him; on his sleep on his movement on his shape on his health so when he sees the good results on him he would commit to it more.

M: practicing and then seeing the result of sports on him; on his health on his shape on his eating or the developments he has so he would hang to it more. That is it. It is a process of getting used to it.

Interviewer1: ok what the factors that help you to do exercise; things that encourages you?

Interviewer2: what encourages or motives you to do exercise?

F1: when I feel I am tired, or my legs are or something like that.

Interviewer2: you get motived!

F1: yes like sitting for a long time makes you feel like you have to do some exercise.

F2: yes if we felt like we are tired of siting a long time it’s better for us to go and some exercise. We don’t like to sit.

F3: We want to move, work and to jog.

Interviewer2: and for you?

F3: what motives me to do sports?!

Interviewer1: yes.

F: Sometimes if one feels fatigue and drowsiness.

F2: but you have to try

F1: yes we try

F: But for me I can walk for half an hour. There is nothing but maybe when I am at work. But I can exchange it at any other time. But I have to walk half an hour

Interviewer 1: so work would be a difficulty but you overcome it and do sport

F3: yes. I walk at least 15 minutes or half an hour, I must walk it every day.

F4: there is nothing that would prevent me from doing sports, on the contrary I am motivated. Sports are very important for the human. In all aspects. It is movement for blood circulation, it would be the blood circulation is okay especially in these times, people are suffering more of diabetes, of high blood pressure, and for sure sports and walking are the best cure for everything for all these diseases.

M1: before I started doing sports, my weight was (Mashalla) over 120 Kgs, I reached this age, the age of 50 I found myself getting tired getting tired I started walking and doing sports the last thing I was practicing walking sports I used to walk around an hour, an hour and quarter till I reduced my weight and became active and this is what made me practice sports.

M6: the physical ability, that one doesn’t come back from work exhausted this is the first thing

M1: no no there is nothing as an obstacle that prevents one from doing sports there is no obstacle at all that the one on the contrary besides the one if he went out the first time, I told you the most important thing is that he himself wants to go out, I started doing sports when I was 50 not when I was hhh I was 50 when I started doing sports and thank god there isn’t an obstacle but the one should have the power and energy and the intention that he starts doing sports go out, not necessary to do body building or weight lifting, go out walk, ride a bike, go to corniche walk for 2 hours, himself, there is not obstacle to the human being.

M1: in the first place my health is the most important. So I care about my health. I walk as much as I can and taking care of my diet also being active not lazy. I see my future in my health. If lost my health then I‘ll lose my future. That what push me to be a healthy person; *Alhamdulillah Rab El-almeen* (Thanks God) *Subhano o Taa’lah;* who created me and made me Muslim and gifted me with (asked to pray) 5 prayers daily. Through the prayer I feel am very active. With God’s bless I pray my *Al-Fajor* at time (dawn prayer) all these are activates I do. Being able to walk to the mosque; Al-Roqua’ and AL-Sojoud (Prayer movement) all these *Alhamdulillah.* Our Islam invited us for theses prayers to be good so I would help in constructing earth

M2: well it’s just a hobby

Interviewer1: a hobby!

M2: my hobby is to do sport and walk and just keep me active.

M2: yes for sure prayer it’s very important thing for me. This is something by default is important. At last it’s like meeting between you and God. If you have spoken to a minster or a country’s state what you will do and at the end the creator and the giver is only God.

M3: prayer is like exercise it makes you move

M: but still I have different opinion that prayer is exercise; prayer is something and exercise is something else. Prayer is a worshipping but exercise

M4: but thinking while praying is different form thinking while doing exercise.

M3: but *Roquaa* or *Sojoud*(prayer movements) is not an exercise.

M4: please forgive me but this is my opinion but *Roquaa* or *Sojoud* is not exercise. *Sojoud* is worship.

M1: prayer is a spiritual exercise; a spiritual exercise and a bodily exercise.

M3: I was seeing a female doctor; an orthopedic doctor for… she said that prayers won’t cause any kind of osteoporosis in joint or rheumatoid so….

M: It’s a psychological relief. It’s much better than Yoga or music or the wine that others drink to find relief.

M2: I encourage myself.

M3: maintain my physical fitness,

M: This depends on the desire to do more exercise or not. It depends on the desire.

M4: ok, yes its deepens if I have extra physical energy to practices after working hours’ time , that will encourage me to do sport, if I have clear mind that will help,

Interviewer1: ok do you have something else you want to add about sport?

F2: well if you became fat a bit you can do some exercise at home

F1: If there is a certain occasion, an important one, all of us will do exercise for this occasion [laughing]

Interviewer1: and you what are the factors that encourage you to do sport?

F3: mostly when I’m upset. When I’m bored I do exercise. Really it makes me feel good. This is the only thing.

F2: no, for us, we got used to healthy food even if it’s presented front of us we don’t get affected. We start to bring brown bread to home and even if we have guests; my husband force them to eat it and they feel bad [laughing].

Female 2: I go to the kitchen and come back.

Female 1: Me what encourage me my health. I can’t sit. I don’t eat the market bread I have to make my own bread by myself this is exercise. I make the dough and cut I don’t havemaid. My children always bring me maid but I don’t want her. She washes my cloths no. I do my entire thing by myself no one make me comfortable even my daughters. Unless I have a feast they come to me my daughters and daughters in law.

Female 3: the activity

F1: the age

F: I try eliminating the tiredness from my body.

F: The movement will help me and makes me more active

F3: in general. Practicing sport is healthy

I3: what are the factors encourage you to practice more sports

F3: when I practice the sport, my body my health, I feel I am more active, nothing specific.

M: if these factors are not there, person should adapt himself, encourage himself and find the motive so he could practices, setting time within 24h for a certain time to practice, the perfect time to practice is after dawn prayer.

M4: Person should give himself motives, activity and always smile, never ever remove the smile from his face,

M3: for me I’m not walking but I swim, I practice swimming.

Female 1: when I feel I am tired or apathy or laziness or I ate a lot.

Female 4: For me it’s normal. I don’t like the AC. [Female 2 interruption: the situation there is different]. No; No because I have the rheumatoid arthritis so when I stay with an open AC the skin get shirked and I go under the sun I feel all the joints get stretched again with the swelling. I mean when the skin is sweating the joints get stretched because of the sun. I love to walk in the sundespite the difficulty.

F: But I try to find half an hour to do so

Female 5: unless I change the routine. It’s necessary to change the routine.

M: it became a lifestyle every one play sports especially guys care about muscles.; he cares about his body; his shape of the muscles

Male 4: the welling the person’s welling is what makes him to practice sport or not. It comes from himself.

Male 6: it depends on wither the sport as a game or in a team I’m registered in may be the goal is for getting the score or the winning, but if it was on the personal level not registered in a team it could be so to be avoid getting ill and to maintain a good health

M: by unless you have a strong motive

Male 5: for me I think the sun or the hotness is not an excuse because we used the life here so it became something normal.

Male 1: no believe me [laughing] my brother *Mash-Allah,* he participates in champions in an incredible way and he wins and so on. And he goes for theses Marathons he finds a lot of Marathons. He thinks he is going to race against them and he will win and he will gain QR100.000. I tell him go ahead, but as what he said… I don’t think that the weather is going to be a barrier as you said earlier but if the person doesn’t like to do exercise whatever the weather was. If the person has the willing for it he will do.

Male 4: time, and life style, it’s depend on how active or lazy I’m in way of life

M: the first one is movement and the second one is controlling the diet. Also the person should avoid two things, sugar and fat; these things the person should fight them. Moreover there are two other things that adversely affects the body, two white things are adversity for the body, salt and sugar.

Male 4: It comes from the person himself no one else. The person should encourage himself to practice physical activity.

1. Presences of will

F1: for me the laziness is motives me to do physical activity, because laziness is a bad feeling, but it can vary from one person to another

F2: When I see outcome of exercise on the other people, I look forward to be like them, I like the outcome on them

F4: sometimes the motive is being Jealous like why these people have nice body, I will want my body to be nice to be like them,

M: Keep practicing a certain sport , not to eat much and say I will do sport because that will lead to be lazy

M2: i think….. you will have the motivation, if your family and friends, if all of them practice sport, i will have the motive to practice more, i will try to give more or to be at same level they are or more.

F: and environment surround me how it’s ,my life at home how its , at university and outside the university , the environments surround me should motivate me to do exercise .

Female1: it is about what you like to do for example: it is you if you have the will to do sport so you will go and do your sport. This is the first thing.

F: The weather shouldn’t be considered as a factor, it should not stop us.

Interviewer1: and you what are the factors that make it easy for you to practice exercise?

F2: the first thing is the weather.

Interviewer1: The weather.

F2: The weather should be nice and this is very important so I can go out and walk. I am that kind of person where weather affects me, the sun, I cannot walk under the sun, or the increased hotness. When the weather becomes nicer, I can go out in the compound and walk for example.

F: I have responsibilities, house and children but I can devote some time for that, I can devote some time to practice exercise. At night, I can practice exercise after I put her

M2: so if the time was suitable I can do my work, we now stick only to watching a football match watch a match like that… but to do physical activity I think we need time and some morals (meaning high spirits)

M: it is perseverance like praying, there should be everyday half an hour that I do sports, and after he does the sports he would see the result of the sports on him; on his sleep on his movement on his shape on his health so when he sees the good results on him he would commit to it more.

F3: We want to move, work and to jog.

Interviewer2: and for you?

F3: what motives me to do sports?!

F2: but you have to try

F1: yes we try

But for me I can walk for half an hour. There is nothing but maybe when I am at work. But I can exchange it at any other time. But I have to walk half an hour

Interviewer 1: so work would be a difficulty but you overcome it and do sport

F3: yes. I walk at least 15 minutes or half an hour, I must walk it every day.

F4: there is nothing that would prevent me from doing sports, on the contrary I am motivated. Sports are very important for the human. In all aspects. It is movement for blood circulation, it would be the blood circulation is okay especially in these times, people are suffering more of diabetes, of high blood pressure, and for sure sports and walking are the best cure for everything for all these diseases.

M2: well it’s just a hobby

Interviewer1: a hobby!

M2: my hobby is to do sport and walk and just keep me active.

M2: I encourage myself.

Interviewer1: ok do you have something else you want to add about sport?

F2: well if you became fat a bit you can do some exercise at home

F1: If there is a certain occasion, an important one, all of us will do exercise for this occasion [laughing]

Interviewer1: and you what are the factors that encourage you to do sport?

F3: mostly when I’m upset. When I’m bored I do exercise. Really it makes me feel good. This is the only thing.

Female 1: Me what encourage me my health. I can’t sit. I don’t eat the market bread I have to make my own bread by myself this is exercise. I make the dough and cut I don’t havemaid. My children always bring me maid but I don’t want her. She washes my cloths no. I do my entire thing by myself no one make me comfortable even my daughters. Unless I have a feast they come to me my daughters and daughters in law.

F: I try eliminating the tiredness from my body.

F: The movement will help me and makes me more active

F3: in general. Practicing sport is healthy

I3: what are the factors encourage you to practice more sports

F3: when I practice the sport, my body my health, I feel I am more active, nothing specific.

M: if these factors are not there, person should adapt himself, encourage himself and find the motive so he could practices, setting time within 24h for a certain time to practice, the perfect time to practice is after dawn prayer.

M4: Person should give himself motives, activity and always smile, never ever remove the smile from his face,

M3: for me I’m not walking but I swim, I practice swimming.

Female 1: when I feel I am tired or apathy or laziness or I ate a lot.

Female 4: For me it’s normal. I don’t like the AC. [Female 2 interruption: the situation there is different]. No; No because I have the rheumatoid arthritis so when I stay with an open AC the skin get shirked and I go under the sun I feel all the joints get stretched again with the swelling. I mean when the skin is sweating the joints get stretched because of the sun. I love to walk in the sundespite the difficulty.

F: But I try to find half an hour to do so

Female 5: unless I change the routine. It’s necessary to change the routine.

it became a lifestyle every one play sports especially guys care about muscles.; he cares about his body; his shape of the muscles

Male 4: the welling the person’s welling is what makes him to practice sport or not. It comes from himself.

Male 6: it depends on wither the sport as a game or in a team I’m registered in may be the goal is for getting the score or the winning, but if it was on the personal level not registered in a team it could be so to be avoid getting ill and to maintain a good health

M: by unless you have a strong motive

Male 5: for me I think the sun or the hotness is not an excuse because we used the life here so it became something normal.

Male 1: no believe me [laughing] my brother *Mash-Allah,* he participates in champions in an incredible way and he wins and so on. And he goes for theses Marathons he finds a lot of Marathons. He thinks he is going to race against them and he will win and he will gain QR100.000. I tell him go ahead, but as what he said… I don’t think that the weather is going to be a barrier as you said earlier but if the person doesn’t like to do exercise whatever the weather was. If the person has the willing for it he will do.

Male 4: time, and life style, it’s depend on how active or lazy I’m in way of life

Male 4: It comes from the person himself no one else. The person should encourage himself to practice physical activity.

1. Desire to exercise

Interviewer 1: yes to activate the blood cycle and health?

M3: even more to entertain yourself.

F5: yeah. It can be for entertaining,

F: or using my extra time that I have and do something that will benefit me like for example exercise.

M1: Ok, what else from the exercise view, the person can focus on the thing he likes more either football, basketball, swimming, running and he should try to mix, not only one thing, gym alone is not alone enough, those who go gym focus on body building and we want fitness to go out any play something. That is it for exercise.

M3: the most important thing is the joy, to feel joy when you are doing the activity.

M5: in my opinion I will talk about this country or most of the countries nowadays, In Qatar, there are gyms, sports clubs, there are many things, and there are trainers and expertise from all over the world especially the main clubs like *Aspire,* those places that are well known and of a great place in the country, they bring the best people there and not anyone. So for myself, If I don’t like to go or don’t have a goal to make my body look like this or be fit enough so when I am placed in a situation I would be able to react. If I don’t have the goal that I want, I will not go to any club. For myself, If is essential to have the goal to have the body and fit, not only shape, there should be fitness in the end.

M2: i think….. you will have the motivation

Female1: it is about what you like to do for example: it is you if you have the will to do sport so you will go and do your sport. This is the first thing.

Interviewer1: and you sir! What makes it easy for you to do sport or physical activity?

M3: well look; nothing is better than the physical activity. First it makes your body and muscles relaxed; conversely it reliefs the stress. I think walking is a good thing it’s kind of sport.

M2: conversely I love sport, I do love sport and I play football. What can I say to you I play even with people age 15 yrs old, I play with them really. [M3 interruption: it’s ok to do so. It’s just a sport; it is for every one whether young or old.

Interviewer1: ok and you?

M3: In fact I’m a sport person

Interviewer1: yeah what eases things for you?

M3: I stopped doing sport as practice but as walking I park my car far away and I always love to walk. This walking is a hoppy.

M: it is perseverance like praying, there should be everyday half an hour that I do sports, and after he does the sports he would see the result of the sports on him; on his sleep on his movement on his shape on his health so when he sees the good results on him he would commit to it more.

M: This depends on the desire to do more exercise or not. It depends on the desire.

M4: ok, yes its deepens if I have extra physical energy to practices after working hours’ time , that will encourage me to do sport, if I have clear mind that will help,

F1: If there is a certain occasion, an important one, all of us will do exercise for this occasion [laughing]

Interviewer1: and you what are the factors that encourage you to do sport?

F3: mostly when I’m upset. When I’m bored I do exercise. Really it makes me feel good. This is the only thing.

1. Feeling tired

M6: the physical ability, that one doesn’t come back from work exhausted this is the first thing

M1: before I started doing sports, my weight was (Mashalla) over 120 Kgs, I reached this age, the age of 50 I found myself getting tired getting tired I started walking and doing sports the last thing I was practicing walking sports I used to walk around an hour, an hour and quarter till I reduced my weight and became active and this is what made me practice sports.

F: Sometimes if one feels fatigue and drowsiness.

Interviewer1: ok what the factors that help you to do exercise; things that encourages you?

Interviewer2: what encourages or motives you to do exercise?

F1: when I feel I am tired, or my legs are or something like that.

Interviewer2: you get motived!

F1: yes like sitting for a long time makes you feel like you have to do some exercise.

F2: yes if we felt like we are tired of siting a long time it’s better for us to go and some exercise. We don’t like to sit.

F3: I walk at home *walla* and do this but I have mental issue in which I feel all tired, I don’t go out, I feel…I feel I am not in the mood.

Interviewer2: this tiredness that prevents you, is there anything that helps you to move?

F3: *Hamdulleah*, when I facilitate my things and adjust it, when I am little tired, I try to facilitate things by myself.

1. Cultural Diversity

M: and environment surround me how it’s ,my life at home how its , at university and outside the university , the environments surround me should motivate me to do exercise

F: Society's perception and the children, you are old now why do you want to exercise, the Society's perception they don’t have that open sight so if the person run or jog in the street that it’s a normal thing, people from society would say see how silly she is running at street, Society's perception.

M: Of course the western countries takes this sport dosage and everyone walks in *Cornich* and walk in the gardens. Everyone has specific hours that they should… and I read about *Condoleezza Rice* who was the foreign minister and security in charge. She has 3 hours that she spends only in exercise. This is very; very; very important

1. Elderly perception

M1: no no there is nothing as an obstacle that prevents one from doing sports there is no obstacle at all that the one on the contrary besides the one if he went out the first time, I told you the most important thing is that he himself wants to go out, I started doing sports when I was 50 not when I was hhh I was 50 when I started doing sports and thank god there isn’t an obstacle but the one should have the power and energy and the intention that he starts doing sports go out, not necessary to do body building or weight lifting, go out walk, ride a bike, go to corniche walk for 2 hours, himself, there is not obstacle to the human being.

M1: before I started doing sports, my weight was (Mashalla) over 120 Kgs, I reached this age, the age of 50 I found myself getting tired getting tired I started walking and doing sports the last thing I was practicing walking sports I used to walk around an hour, an hour and quarter till I reduced my weight and became active and this is what made me practice sports.

1. Exercise outcome

M: practicing and then seeing the result of sports on him; on his health on his shape on his eating or the developments he has so he would hang to it more. That is it. It is a process of getting used to it.

1. **Having support**

M: I most of the time pray all the prayers with groups so I consider walking to the prayers is sports

M4: first thing our prayers and then what you want to do you can do.

M1: especially if there was in the place something that encourages you

M2: exactly yes.

Interviewer 1: aha

M1: I mean on your own you would get bored but when you find other people with you present there

M1: you would be encouraged to practice

M: to have a team so we can do training which can match out level.

M: and to have my friends, that what motivate me

Male5: when there is a place where he has friends and colleagues inside and they go to gym or they run together so that motivate him and he will join them in the group

M: and of courses the friends and colleagues to do the activities.

M: The second thing is the company, when you are in a company and not alone; this will makes it easy for you

M2: Encouragement, someone to encourage you like the mother or father who encourage you to go or to bring you fitness machines at home. This is better, this is a motivation. This can adjust your time.

M: The second thing is when you have encouragement for sure.

M: For friends, of course the encourage because sometimes you get bored, or feel relaxed, yes friends encourage you but it is not the main thing.

M: second role comes on the family if they encourage kids to play sports, take them to clubs or school practices sport, it will help that particular group to practice sport or any physical activity, that is.

M2: i think….. you will have the motivation, if your family and friends, if all of them practice sport, i will have the motive to practice more, i will try to give more or to be at same level they are or more.

M3: the most motive thing family and friend encouragement, to provide a lot of machines such walking machines and more advertising

I: Q2: ok, what are the factors that make you to practice sports, or to do any hobbies you like, or encourage you to?

Female1: I must be accompanied with someone so he can motivate me and I can motivate him

I: and you what are the factors encourage you?

Female2: I see the supports came from mother and father, they should teach their kids, no one is born having this knowledge, his family teach him, for example, when you want to reach goal of making your body to be appropriate to do sports your body should be fit and eat healthy diet, there are parents who give their kids fast food which is not healthy and when they reach 5 years they will be obese

Female3: usually the motivation from companionship if you chose person to go with you for a walk,

F: So the motive is the company and the presence of help from person once you need it can be a motive to person to practice sport.

I: good and you?

Female4: as they said its depend on having company or group of cousin girls how will make agreement to do physical activity together or to like certain sports will help them to love or do sport.

F3: encouragement

F2: here there is no encouragement, no encouragement from husband, her kids, You are old, that is it,

*M: Omar* PBUH (one of prophet companions) said: teach your children swimming, shooting and riding horses

F?: and presence of a partner or someone encourage you: let us go and do sport. These are the factors facilitate to do sport

Female1: as she said, if there is someone to encourage you and go with you.

Female3: it makes a difference

Female1: it can be good

Interviewer2: Ok these things that make it difficult for you the joint pain, this movement that made it difficult for you to do exercise, is there anything that makes it easy for you to do exercise? Easy things?

F1: the pool in the physiotherapy is the thing that made it easy for you.

Interviewer2: this made it easy for you to move and practice exercise.

F1: you will do exercise without caring about anything else. You will not feel pain when you are in the pool. I have been getting treatment for 3 months there with them and hamduleah I did not feel anything.

F: Also the physiotherapist, when he writes a treatment for me and I have transportation to go there, where the physiotherapy is on machines more than on the bed, I use for example the treadmill or the cycler which will help in exercise.

F: At home nothing at all [laugh] although there is a treadmill and other machines but there is no encouragement to go and walk or practice exercise.

F: And also a friend to encourage me. A friend to help.

Interviewer1: so you try to encourage yourself?

F3: I get encouraged when I am with the kids, grandchildren, I send them to the training, I play with them, I do things for them so I can forget, forget.

Interviewer2: yes yes.

Interviewer1: No, the things that makes it easy for you?

F4: the things that make it easy for me is the presence of someone who support me, presence of a person who is specialized to direct me to the moves,

F: If you found a compound that will give you space to walk as exercise which is better than…l though I prefer exercise in the natural environment, the sea or somewhere. This would be better to practice exercise.

Frankly speaking, the physiotherapy that I had at Qatar hospital made me feels better.

Interviewer1: Hamdulleah.

F2: I felt I can move faster and more.

M: Hamduleah, I have nothing, my husband encourages me to go and do this, do exercise but I, I don’t find myself.

M1 interruption: how did I overcome this thing is I walk inside my house. I have a huge hall it’s around 10*6 meter. During my walking I say some kind of *Thekkr* Allah (something close to hymns). It’s around 20-30 rounds.

M3: by the way the best thing for your soul is hymns. Goodness comes by hymns. Health comes by hymns.

M1: this *Thekkr* Allah keeps devils away from you. So when you become able to receives devils whispering. He fills your heart with fears and stress when you are not related to Allah.

M3 interruption: it keeps the devils whispering away from you. you know when you start thinking about God

Male 3: according to me, it is young men

Interviewer: young men? What do you mean?

Male 3: in case there is motivation like if the young men will go out for running and playing, then I can go.

Interviewer: young men to be with you

Male 3: yes, young men

F3: it could be the place for example, public places, a park or in a public place where I can see others around me starting a particular physical activity so this would encourage me and make me want to practice and join the activity.

F: and encouragement from a friend for example.

F4: ---- it’s the role of each mother and the role of each family in the house to encourage their children not to set in long hours on TV or computers and electronic device because these basically are harmful!

F2: That is right.

F4: highly harmful! See (الحركه بركه) (Prophet said that ) it’s good to be active,

F2: in movement god bless you as you said by machines and these stuff and eating. See how eating became…

M: when you are a team together.

M: the friends, friends would encourage a lot to practice sports

M2: yes for sure prayer it’s very important thing for me. This is something by default is important. At last it’s like meeting between you and God. If you have spoken to a minster or a country’s state what you will do and at the end the creator and the giver is only God.

M3: prayer is like exercise it makes you move

M1: If would you excuse me, Islam with all its rituals and worships calls for exercise

M1: fulfilling the rituals of Pilgrimage, *Sa’ay and Tawaf* (walking fast back and forth), walking in pilgrimage, Prayers and when you walk to pray and fasting which relates to the calories. Helping others with their needs, removing what is bad from the street, this is all movement.

M3: The movement while praying.

M1: Our Prophet, used to remove his shoes and walk on the sand, and now they found out that it absorbs the negative charges from the human body. This is surprising. In one of the Hadith “ Banu Salima( a family) had a land next to the mosque while their houses were far away, so they told the prophet that they will rent their remote houses and build new ones next to the mosque. The Prophet replied, stay at your houses, your footprints will be recorded” So the Prophet urges that the houses be far from the mosque and he said more rewards are to those who are far from the mosque and walk. So they started to walk. Secondly I want end this by saying that the Prophet’s walk was fast walking and the proof of that is the sweating. I have read in a research that fast walking is the one that burns calories and better breathing but the slow walking you do not benefit from even if you walked slowly for 10km, you will not benefit from it. The Prophet used to walk fast as if you are going down from a mountain, when you go down from a mountain you will run fast, so the prophet came for health, and if we practiced our religion in prayers and timely prayers and walking to the mosque to pray, we will not need anything else.

M4: I can’t agree more to what they said. There is hadith of Prophet Mohammed “teach your sons the swimming, archery and horse riding” which encourage them for doing sport

M1: no; no. the man is well cultured he made a point which is prayer is worship so how you would consider it exercise. Let me say something for every religion has certain times where they extract the negative energy for example in East of Asia they have what is so called the Yoga. They feel like by it they take out all the negative energy a lot of it. The Christians like to listen to music and so on; so on. We are as Muslims prayer is the alternative i.e. it’s a spiritual exercise.

M: So prayer is our relief. At the same time it’s where we seek relief with it spiritually and physically. Imagine your dawn prayer when you do it… less people to have stroke are who pray the down prayer; why? They found the continuous sleeping for 8 hrs cause stasis in the blood circulation, in medical and field research it’s found that less people to have stroke are who pray the dawn prayer together. And

F: someone to encourage you, you need someone beside you to encourages you,

M1: the activity is when your pocket is full, home is comfortable, kids are comfortable, you will be that time psychologically active

M4: that is Pessimism, your pocket is empty but fill yourself with faith, because faith is easy

M4: because when you fill yourself with reading holy Quran, and be with Allah M2: Faith, you should have hope and strong relation with God,

M4: you will be active and strong

M2: you should have hope and strong relation with God

M4: I will tell you something a few bites, small bites as lessons, about kids even though some of the messenger their kids were good other have not good kids, whatever you are able to do at home with simple few of food and smile as well having kids , With patience, and love you will reach to internal happiness

M1: Praying on time that is the most important for me, that is the exercise for me, the important thing, praying on time, dawn Prayer, afternoon prayer and evening prayer, that is full exercise, but a person who do exercise and swim then go to sleep, when Prayer calls, he is still sleeping and cannot go. If he went for prayer *Thuhr and Asr,* this is the most important thing.

M2: yes this is also a point, ok what else.

Female 5: It was walking area when you walk.Everyone is walking so you get the encouragement. In Aspire you can find some people are sitting, children are playing; you scare to hit them. There wasan open space for playing

Female 1: yes the distances and walking but the opposite the old women but we are thanks God even I would love the older so that would not effect on their aromatize. Even when I go for walking at Aspire I see even old women a very old, oldandwhen I see them I get encouraged. Some of them are grandma and they circle around wearing sport shoes they do not walk with normal shoes. At the opposite I get encouraged more and I walk more. I can say Aspire;thanks God for the walking space.

Interviewer: What are the factors that make it easy for you to engage in the physical activity? I mean what are the factors that make it easy for you to engage in the physical activity? What encourage you?

Female 1: Encouragement; in the last period we made a competition with my friends to motivate each other who lose more weight will get gift at the end. So we started the competition.

Interviewer: you mean you with your friends.

Female 1: Yes.

Female 2: There should be someone encourage you to do the exercise better than alone for example your family, sister, trainer. When you are alone there will be no wish.

F: At the same time you need someone to encourage you. Encouragement you need to have someone encourage you. If you could not find someone to encourage you will not.

Female 3: Because some people they don’t continue for example me I don’t continue to do the exercise, three four times and I stop and get bored. I need encouragement .If someone does it with me I will continue.

Female 6: necessary to have someone with me.

M: well with guys we play football, we play rugby; you have someone to encourage you to play sport so for sure every day we’ll play sport.

M: the factors are like,,, it became a lifestyle every one play sports especially guys care about muscles.; he cares about his body; his shape of the muscles. All the new tools, GYM and all your friends encourage you to the GYM; they ask you to go the GYM. So the youth and the friends are the factors so the guys and the friends.

Interviewer: You mean to be in a sport group!

Male 1: yes

Male 2: my friends. I mean I mean I love to play with my friends. I love to go out, to go out with them and do things together.

M: Add to this the one has sport activity wither in the university or with his friends outside especially if am a team leader in some sports I have to be keep on practicing sport so I would be an icon for others in these things so this encourage me to do sport.

Male 3: there is an essential factor that contributes which is education and upbringing. For example when someone came from sport culture were family support child, eventually child will be athletic, also education

Male 3: also education when student start healthy diet since childhood and do exercise, It was difficult for us to practice sport because we did not have such supportive environment , unlikely student who have the support from his parents since childhood its different, student will continue to do exercise forever.

Interviewer: So, he will get to do sport for the rest of his life.

Male 3: So the background he came from, if education did not support, if media did not do support to practice sport, people will not practice.

1. Group support

M1: especially if there was in the place something that encourages you

M2: exactly yes.

Interviewer 1: aha

M1: I mean on your own you would get bored but when you find other people with you present there

M1: you would be encouraged to practice

M: to have a team so we can do training which can match out level.

M: and to have my friends, that what motivate me

Male5: when there is a place where he has friends and colleagues inside and they go to gym or they run together so that motivate him and he will join them in the group

M: and of courses the friends and colleagues to do the activities.

M: The second thing is the company, when you are in a company and not alone; this will makes it easy for you

M2: Encouragement, someone to encourage you like the mother or father who encourage you to go or to bring you fitness machines at home. This is better, this is a motivation. This can adjust your time.

M: The second thing is when you have encouragement for sure.

M: For friends, of course the encourage because sometimes you get bored, or feel relaxed, yes friends encourage you but it is not the main thing.

M2: i think….. you will have the motivation, if your family and friends, if all of them practice sport, i will have the motive to practice more, i will try to give more or to be at same level they are or more.

M3: the most motive thing family and friend encouragement, to provide a lot of machines such walking machines and more advertising

I: Q2: ok, what are the factors that make you to practice sports, or to do any hobbies you like, or encourage you to?

Female1: I must be accompanied with someone so he can motivate me and I can motivate him

Female3: usually the motivation from companionship if you chose person to go with you for a walk,

So the motive is the company and the presence of help from person once you need it can be a motive to person to practice sport.

I: good and you?

Female4: as they said its depend on having company or group of cousin girls how will make agreement to do physical activity together or to like certain sports will help them to love or do sport.

F: and presence of a partner or someone encourage you: let us go and do sport. These are the factors facilitate to do sport

Female1: as she said, if there is someone to encourage you and go with you.

Female3: it makes a difference

Female1: it can be good

F: At home nothing at all [laugh] although there is a treadmill and other machines but there is no encouragement to go and walk or practice exercise.

F: And also a friend to encourage me. A friend to help.

Interviewer1: so you try to encourage yourself?

F3: I get encouraged when I am with the kids, grandchildren, I send them to the training, I play with them, I do things for them so I can forget, forget.

Interviewer2: yes yes.

Interviewer1: No, the things that makes it easy for you?

F4: the things that make it easy for me is the presence of someone who support me, presence of a person who is specialized to direct me to the moves,

F: If you found a compound that will give you space to walk as exercise which is better than…l though I prefer exercise in the natural environment, the sea or somewhere. This would be better to practice exercise.

Male 3: according to me, it is young men

Interviewer: young men? What do you mean?

Male 3: in case there is motivation like if the young men will go out for running and playing, then I can go.

Interviewer: young men to be with you

Male 3: yes, young men

F3: it could be the place for example, public places, a park or in a public place where I can see others around me starting a particular physical activity so this would encourage me and make me want to practice and join the activity.

F: and encouragement from a friend for example.

M: when you are a team together.

F: the friends, friends would encourage a lot to practice sports

F: someone to encourage you, you need someone beside you to encourages you,

Female 5: It was walking area when you walk.Everyone is walking so you get the encouragement. In Aspire you can find some people are sitting, children are playing; you scare to hit them. There wasan open space for playing

Female 1: yes the distances and walking but the opposite the old women but we are thanks God even I would love the older so that would not effect on their aromatize. Even when I go for walking at Aspire I see even old women a very old, oldandwhen I see them I get encouraged. Some of them are grandma and they circle around wearing sport shoes they do not walk with normal shoes. At the opposite I get encouraged more and I walk more. I can say Aspire;thanks God for the walking space.

Interviewer: What are the factors that make it easy for you to engage in the physical activity? I mean what are the factors that make it easy for you to engage in the physical activity? What encourage you?

Female 1: Encouragement; in the last period we made a competition with my friends to motivate each other who lose more weight will get gift at the end. So we started the competition.

Interviewer: you mean you with your friends.

Female 1: Yes.

Female 2: There should be someone encourage you to do the exercise better than alone for example your family, sister, trainer. When you are alone there will be no wish.

F: At the same time you need someone to encourage you. Encouragement you need to have someone encourage you. If you could not find someone to encourage you will not.

Female 3: Because some people they don’t continue for example me I don’t continue to do the exercise, three four times and I stop and get bored. I need encouragement .If someone does it with me I will continue.

Female 6: necessary to have someone with me.

M: well with guys we play football, we play rugby; you have someone to encourage you to play sport so for sure every day we’ll play sport.

M: the factors are like,,, it became a lifestyle every one play sports especially guys care about muscles.; he cares about his body; his shape of the muscles. All the new tools, GYM and all your friends encourage you to the GYM; they ask you to go the GYM. So the youth and the friends are the factors so the guys and the friends.

Interviewer: You mean to be in a sport group!

Male 1: yes

Male 2: my friends. I mean I mean I love to play with my friends. I love to go out, to go out with them and do things together.

M: Add to this the one has sport activity wither in the university or with his friends outside especially if am a team leader in some sports I have to be keep on practicing sport so I would be an icon for others in these things so this encourage me to do sport.

1. Role of Religion

M: I most of the time pray all the prayers with groups so I consider walking to the prayers is sports

M4: first thing our prayers and then what you want to do you can do.

*M: Omar* PBUH (one of prophet companions) said: teach your children swimming, shooting and riding horses

M1 interruption: how did I overcome this thing is I walk inside my house. I have a huge hall it’s around 10*6 meter. During my walking I say some kind of *Thekkr* Allah (something close to hymns). It’s around 20-30 rounds.

M3: by the way the best thing for your soul is hymns. Goodness comes by hymns. Health comes by hymns.

M1: this *Thekkr* Allah keeps devils away from you. So when you become able to receives devils whispering. He fills your heart with fears and stress when you are not related to Allah.

M3 interruption: it keeps the devils whispering away from you. you know when you start thinking about God

F4: highly harmful! See (الحركه بركه) (Prophet said that ) it’s good to be active,

F2: in movement god bless you as you said by machines and these stuff and eating. See how eating became…

M2: yes for sure prayer it’s very important thing for me. This is something by default is important. At last it’s like meeting between you and God. If you have spoken to a minster or a country’s state what you will do and at the end the creator and the giver is only God.

M3: prayer is like exercise it makes you move

M1: If would you excuse me, Islam with all its rituals and worships calls for exercise

M1: fulfilling the rituals of Pilgrimage, *Sa’ay and Tawaf* (walking fast back and forth), walking in pilgrimage, Prayers and when you walk to pray and fasting which relates to the calories. Helping others with their needs, removing what is bad from the street, this is all movement.

M3: The movement while praying.

M1: Our Prophet, used to remove his shoes and walk on the sand, and now they found out that it absorbs the negative charges from the human body. This is surprising. In one of the Hadith “ Banu Salima( a family) had a land next to the mosque while their houses were far away, so they told the prophet that they will rent their remote houses and build new ones next to the mosque. The Prophet replied, stay at your houses, your footprints will be recorded” So the Prophet urges that the houses be far from the mosque and he said more rewards are to those who are far from the mosque and walk. So they started to walk. Secondly I want end this by saying that the Prophet’s walk was fast walking and the proof of that is the sweating. I have read in a research that fast walking is the one that burns calories and better breathing but the slow walking you do not benefit from even if you walked slowly for 10km, you will not benefit from it. The Prophet used to walk fast as if you are going down from a mountain, when you go down from a mountain you will run fast, so the prophet came for health, and if we practiced our religion in prayers and timely prayers and walking to the mosque to pray, we will not need anything else.

M4: I can’t agree more to what they said. There is hadith of Prophet Mohammed “teach your sons the swimming, archery and horse riding” which encourage them for doing sport

M1: no; no. the man is well cultured he made a point which is prayer is worship so how you would consider it exercise. Let me say something for every religion has certain times where they extract the negative energy for example in East of Asia they have what is so called the Yoga. They feel like by it they take out all the negative energy a lot of it. The Christians like to listen to music and so on; so on. We are as Muslims prayer is the alternative i.e. it’s a spiritual exercise.

M: So prayer is our relief. At the same time it’s where we seek relief with it spiritually and physically. Imagine your dawn prayer when you do it… less people to have stroke are who pray the down prayer; why? They found the continuous sleeping for 8 hrs cause stasis in the blood circulation, in medical and field research it’s found that less people to have stroke are who pray the dawn prayer together. And

M4: that is Pessimism, your pocket is empty but fill yourself with faith, because faith is easy

M4: because when you fill yourself with reading holy Quran, and be with Allah M2: Faith, you should have hope and strong relation with God,

M4: you will be active and strong

M2: you should have hope and strong relation with God

M4: I will tell you something a few bites, small bites as lessons, about kids even though some of the messenger their kids were good other have not good kids, whatever you are able to do at home with simple few of food and smile as well having kids , With patience, and love you will reach to internal happiness

M1: Praying on time that is the most important for me, that is the exercise for me, the important thing, praying on time, dawn Prayer, afternoon prayer and evening prayer, that is full exercise, but a person who do exercise and swim then go to sleep, when Prayer calls, he is still sleeping and cannot go. If he went for prayer *Thuhr and Asr,* this is the most important thing.

M2: yes this is also a point, ok what else.

1. Family support

M2: Encouragement, someone to encourage you like the mother or father who encourage you to go or to bring you fitness machines at home. This is better, this is a motivation. This can adjust your time.

M: second role comes on the family if they encourage kids to play sports, take them to clubs or school practices sport, it will help that particular group to practice sport or any physical activity, that is.

M3: the most motive thing family and friend encouragement, to provide a lot of machines such walking machines and more advertising

I: and you what are the factors encourage you?

Female2: I see the supports came from mother and father, they should teach their kids, no one is born having this knowledge, his family teach him, for example, when you want to reach goal of making your body to be appropriate to do sports your body should be fit and eat healthy diet, there are parents who give their kids fast food which is not healthy and when they reach 5 years they will be obese

F3: encouragement

F2: here there is no encouragement, no encouragement from husband, her kids, You are old, that is it,

F: Hamduleah, I have nothing, my husband encourages me to go and do this, do exercise but I, I don’t find myself.

F4: ---- it’s the role of each mother and the role of each family in the house to encourage their children not to set in long hours on TV or computers and electronic device because these basically are harmful!

F2: That is right.

Female 2: There should be someone encourage you to do the exercise better than alone for example your family, sister, trainer. When you are alone there will be no wish.

Male 3: there is an essential factor that contributes which is education and upbringing. For example when someone came from sport culture were family support child, eventually child will be athletic, also education

Male 3: also education when student start healthy diet since childhood and do exercise, It was difficult for us to practice sport because we did not have such supportive environment , unlikely student who have the support from his parents since childhood its different, student will continue to do exercise forever.

Interviewer: So, he will get to do sport for the rest of his life.

Male 3: So the background he came from, if education did not support, if media did not do support to practice sport, people will not practice.

1. Role of physiotherapy

F: Frankly speaking, the physiotherapy that I had at Qatar hospital made me feels better.

Interviewer1: Hamdulleah.

F2: I felt I can move faster and more.

F: Also the physiotherapist, when he writes a treatment for me and I have transportation to go there, where the physiotherapy is on machines more than on the bed, I use for example the treadmill or the cycler which will help in exercise.

Interviewer2: Ok these things that make it difficult for you the joint pain, this movement that made it difficult for you to do exercise, is there anything that makes it easy for you to do exercise? Easy things?

F1: the pool in the physiotherapy is the thing that made it easy for you.

Interviewer2: this made it easy for you to move and practice exercise.

F1: you will do exercise without caring about anything else. You will not feel pain when you are in the pool. I have been getting treatment for 3 months there with them and hamduleah I did not feel anything.

1. Financial factor

M1: the activity is when your pocket is full, home is comfortable, kids are comfortable, you will be that time psychologically active

1. **Environment**

Interviewer 1: okay what are the factors that Makes it easy for you to engage in physical activity? Or what makes it easy for you to do sports or any activity? -----

M1: depending on what is available in the house, if halls, halls and these things anything that helps you that you practice sports. Thank god these things are available now, if not available you go to walking

M1: especially if there was in the place something that encourages you

M2: exactly yes.

Interviewer 1: aha

M1: I mean on your own you would get bored but when you find other people with you present there

Male2: first of all, what helps me to practice the sport is the weather,

Male5: when there is a place where he has friends and colleagues inside and they go to gym or they run together so that motivate him and he will join them in the group

Male6: first, availability of the suitable environment for the sport activities, tools or the suitable environment

M4: in my opinion the best ~~the~~ thing that motivates most is the availability of sports clubs. In each club there should be someone who will give you the correct awareness. When you are doing exercise alone you can do something extra or decrease an activity, on the other hand, when you are in a club and there is a doctor who knows what is excess in your body or less, he would also know how many time per day for exercise you need so you won’t feel exhausted.

M3: to build particular clubs that specialized for gym or anything else with availability of trainer to qualify all youth categories

M4: to have Sport culture person should believe on the sports outcome, that is the most important thing, other things are considered facilitating factors.

M3: the most motive thing family and friend encouragement, to provide a lot of machines such walking machines and more advertising

F: and environment surround me how it’s ,my life at home how its , at university and outside the university , the environments surround me should motivate me to do exercise .

F: or gym its motives you, also environment is a motive, environment that you will use it to practice sports.

F: For example if are wearing ***haejab( scarf that Muslim women wear it to cover their hair )***  and you went to go to sports club, if its mixed club it will cause the laziness to that person, because you will say”oh” I should wear long dress and ***haejab*** because its mixed while if you knew that the club sport for girls, you will feel comfortable, and this would be a motive, not necessary to wear long sleeves or ***haejab*** as we have at university.

F: places should be open so the person can jog their as clubs, marathon competition for women only, it’s not available her and in other Arabic country for women.

I1: or easy to practice sport?

F3: as she said [f2] weather, the most important is the weather,

F:: also open places to practices sport there are places for woman, like parks, Cornish.

M2: Physical activity it is mostly the exercise dosage that the person take on the ordinary days or weekly and that is according to his functional (work) performance. Some people have the chance daily,

M2: it is all activity, whatever the activities are, depends upon the infrastructure

Interviewer2: what do you mean?

Interviewer1: how?

M2: The infrastructure is availability of means of doing exercise.

M1: everything available, gardens, *Aspire, Cornich*. Everything provided thanks God.

Interviewer1: so availabilities of the gardens encourage you to practice sports

M1: yes for the person….

F: secondly if there are available places to do this sport. Availability for ladies places especially in this area there are not many places to go and walk or to do sport, especially here. Presence of many workers here will definitely reduce the capability to go out.

Interviewer1: What else on the level of the country as a suggestion to encourage people, you mentioned the, The psychological motives, what else could be provided to promote women to practice exercise?

F1: The country can provide a club for the elderly

Male 2: good places, green lands,

Male4: honestly there are many places to play sports

Male 1: there are *Aspire and Katara*

Male 4: you can go to cornich for walking

Male 1: make some bathrooms in these places, so you can change when you sweat, so you can go out.

F3: it could be the place for example, public places, a park or in a public place where I can see others around me starting a particular physical activity so this would encourage me and make me want to practice and join the activity.

Interviewer 1: okay. Our question; what are the factors that makes it easy for you to practice physical activity? What motivates you? ------

M1: what motivates us is that there would be qualified places that one would be able to practice his activity

Interviewer 1: like what?

M1: clubs of course

Interviewer 1: okay. What are the factors or the conditions that makes it easy for you to participate in physical activities? What makes it easy for you?

F1: participating in a health club,

M3: Thank God this country provides everything, Aspire, even the machines are available

M3: everything that we need you find it, so if one goes there he’d find everything in front of you.

M5: I mean that some people work and don’t find time to practice sports, they work early in the morning and they finish late so they don’t find time

M: the place where you can practice sports

M: the abilities like for examples the infrastructure, the place that you can go to…

M: and the availability place.

M1: the nature such as garden, calm atmosphere that’s are the most factors to me

M: the atmosphere

M4: if there is suitable atmosphere, it does not matter due to availability of air conditioner, certain sports are practiced outdoors when weather is not suitable and it would be difficult.

Interviewer1: so you mean boredom?!

F4: the place, time, someone to encourage you, you need someone beside you to encourages you, the place and time.

M4: yes, the factors if you are at home, comfortable at home and having a wife who is helping and encouraging you to go to practice, let’s go children, having a daughter or brother, these are one of the factors, if these factors are not there, person should adapt himself, encourage himself and find the motive so he could practices, setting time within 24h for a certain time to practice, the perfect time to practice is after dawn prayer.

M: home is comfortable, kids are comfortable,

Female 3: The availability of the fitness center also encourages me to do exercise; I don’t need to go to the Cornish there are places near me I can go.

Female 4: Any area has its own walking place thanks God.

Female 3: The places, for example I joined Aspire, and I have the tread-mill device, they follow me always.

Female 3:fitness centers with reasonable prices like Aspire. Also

Female 4: For example the one in Wiznan mall; my husband joined it and lost 30 kilograms.

Interviewer2: is it a dietitian center.

Female 4: yes they are a lot of centers like this.

Female 1: outside the center there is a big space you can walk on it. There is available place for walking other than the centers. Even in Aspire the gardens are everywhere and between the neighborhoods there are gardens and walking trails. I don’t see any obstacles.

Female 2: Every neighborhood has garden and available place for walking. And even without the garden some streets have walking trails that written on it so the cars don’t come on it.

Female 1: yes the distances and walking but the opposite the old women but we are thanks God even I would love the older so that would not effect on their aromatize. Even when I go for walking at Aspire I see even old women a very old, old and when I see them I get encouraged. Some of them are grandma and they circle around wearing sport shoes they do not walk with normal shoes. At the opposite I get encouraged more and I walk more. I can say Aspire; thanks God for the walking space.

M: All the new tools, GYM and all your friends encourage you to the GYM; they ask you to go the GYM. So the youth and the friends are the factors so the guys and the friends.

M: the nice weather. When the weather is nice and you gather with the guys they encourage us, and let us to go out and exercise.

M: The environment you lives in is the factor it depends in how you to play. It is the helping factor. If you’re in an environment where you find there is no sport; no one plays so your situation is no one to help me; so I won’t go to play sport.

M: And the second factor is the sport places like Aspire, if you find such places everywhere these sport places helps you to exercise. These sport places are a useful factor to play sport.

M: availability of facility

Male1: also environment

M: I have seen a TV program in a Western country that they provide gym place at work place to assist the staff,

M:The interviewer comments that the availability of parks facilitates you to practice physical activity and Participant 2 relies back yes off course it make it easy for us to practice.

M: Here in Qatar they provide many places for walking such as Aspire.

1. Physical environment

F:secondly if there are available places to do this sport. Availability for ladies places especially in this area there are not many places to go and walk or to do sport, especially here. Presence of many workers here will definitely reduce the capability to go out.

Male5: when there is a place where he has friends and colleagues inside and they go to gym or they run together so that motivate him and he will join them in the group

Male6: first, availability of the suitable environment for the sport activities, tools or the suitable environment

M4: in my opinion the best ~~the~~ thing that motivates most is the availability of sports clubs. In each club there should be someone who will give you the correct awareness. When you are doing exercise alone you can do something extra or decrease an activity, on the other hand, when you are in a club and there is a doctor who knows what is excess in your body or less, he would also know how many time per day for exercise you need so you won’t feel exhausted.

M3: to build particular clubs that specialized for gym or anything else with availability of trainer to qualify all youth categories

important thing, other things are considered facilitating factors.

M4: to have Sport culture person should believe on the sports outcome, that is the most

M3: the most motive thing family and friend encouragement, to provide a lot of machines such walking machines and more advertising

F:and environment surround me how it’s ,my life at home how its , at university and outside the university , the environments surround me should motivate me to do exercise .

F:or gym its motives you, also environment is a motive, environment that you will use it to practice sports

F:For example if are wearing ***haejab( scarf that Muslim women wear it to cover their hair )***  and you went to go to sports club, if its mixed club it will cause the laziness to that person, because you will say”oh” I should wear long dress and ***haejab*** because its mixed while if you knew that the club sport for girls, you will feel comfortable, and this would be a motive, not necessary to wear long sleeves or ***haejab*** as we have at university.

F:places should be open so the person can jog their as clubs, marathon competition for women only, it’s not available her and in other Arabic country for women.

F:also open places to practices sport there are places for woman, like parks, Cornish.

M2: Physical activity it is mostly the exercise dosage that the person take on the ordinary days or weekly and that is according to his functional (work) performance. Some people have the chance daily,

M2: it is all activity, whatever the activities are, depends upon the infrastructure

Interviewer2: what do you mean?

Interviewer1: how?

M2: The infrastructure is availability of means of doing exercise.

M2: The cultural role of giving awareness to the population about the concept of exercise itself and to maintain his body have big role to encourage the…

M1: everything available, gardens, *Aspire, Cornich*. Everything provided thanks God.

Interviewer1: so availabilities of the gardens encourage you to practice sports

M1: yes for the person

the, The psychological motives, what else could be provided to promote women to practice exercise?

F1: The country can provide a club for the elderly

Interviewer1: What else on the level of the country as a suggestion to encourage people, you mentioned

Male 2: good places, green lands

Male4: honestly there are many places to play sports

Male 1: there are *Aspire and Katara*

Male 4: you can go to cornich for walking

Male 1: make some bathrooms in these places, so you can change when you sweat, so you can go out.

F3: it could be the place for example, public places, a park or in a public place where I can see others around me starting a particular physical activity so this would encourage me and make me want to practice and join the activity.

Interviewer 1: okay. Our question; what are the factors that makes it easy for you to practice physical activity? What motivates you? ------

M1: what motivates us is that there would be qualified places that one would be able to practice his activity

Interviewer 1: like what?

M1: clubs of course

Interviewer 1: okay. What are the factors or the conditions that makes it easy for you to participate in physical activities? What makes it easy for you?

F1: participating in a health club,

M3: Thank God this country provides everything, Aspire, even the machines are available

M3: everything that we need you find it, so if one goes there he’d find everything in front of you.

M5: I mean that some people work and don’t find time to practice sports, they work early in the morning and they finish late so they don’t find time

M:the place where you can practice sports

M:the abilities like for examples the infrastructure, the place that you can go to…

M:and the availability place.

M1: the nature such as garden, calm atmosphere that’s are the most factors to me

M: the atmosphere

M4: if there is suitable atmosphere, it does not matter due to availability of air conditioner, certain sports are practiced outdoors when weather is not suitable and it would be difficult.

Interviewer1: so you mean boredom?!

F4: the place, time, someone to encourage you, you need someone beside you to encourages you, the place and time.

M4: yes, the factors if you are at home, comfortable at home and having a wife who is helping and encouraging you to go to practice, let’s go children, having a daughter or brother, these are one of the factors, if these factors are not there, person should adapt himself, encourage himself and find the motive so he could practices, setting time within 24h for a certain time to practice, the perfect time to practice is after dawn prayer.

M: home is comfortable, kids are comfortable,

Female 3: The availability of the fitness center also encourages me to do exercise; I don’t need to go to the Cornish there are places near me I can go.

Female 4: Any area has its own walking place thanks God.

Female 3: The places, for example I joined Aspire, and I have the tread-mill device, they follow me always

Female 3:fitness centers with reasonable prices like Aspire. Also

Female 4: For example the one in Wiznan mall; my husband joined it and lost 30 kilograms.

Interviewer2: is it a dietitian center.

Female 4: yes they are a lot of centers like this.

Female 1: outside the center there is a big space you can walk on it. Thereis available place for walking other than the centers. Even in Aspire the gardens are everywhere and between the neighborhoods there are gardensand walking trails. I don’t see any obstacles.

Female 2: Every neighborhood has garden and available place for walking. And even without the garden some streets have walking trails that written on it so the cars don’t come on it.

Female 1: yes the distances and walking but the opposite the old women but we are thanks God even I would love the older so that would not effect on their aromatize. Even when I go for walking at Aspire I see even old women a very old, oldandwhen I see them I get encouraged. Some of them are grandma and they circle around wearing sport shoes they do not walk with normal shoes. At the opposite I get encouraged more and I walk more. I can say Aspire;thanks God for the walking space.

M: All the new tools, GYM and all your friends encourage you to the GYM; they ask you to go the GYM. So the youth and the friends are the factors so the guys and the friends

M: The environment you lives in is the factor it depends in how you to play. It is the helping factor. If you’re in an environment where you find there is no sport; no one plays so your situation is no one to help me; so I won’t go to play sport.

M: And the second factor is the sport places like Aspire, if you find such places everywhere these sport places helps you to exercise. These sport places are a useful factor to play sport.

M: availability of facility

Male1: also environment

M: I have seen a TV program in a Western country that they provide gym place at work place to assist the staff

M: Here in Qatar they provide many places for walking such as Aspire

M: The interviewer comments that the availability of parks facilitates you to practice physical activity and Participant 2 relies back yes off course it make it easy for us to practice.

1. Natural environment

M: the nice weather. When the weather is nice and you gather with the guys they encourage us, and let us to go out and exercise.

Female 2: If we do outside exercise is not like inside and if the weather is hot I feel it’s difficult to do exercise, unless it is indoors, then yes.

Female 2: the weather sometimes.

M2: yes that is right.

M1: yes, this is the time (after dawn prayer)

M4: after dawn prayer, the weather will be good, you can practice

M2: one of the factors is when the weather is suitable, yes that is right.

M1: which factor, after 24 years!!

M2: yes one of the factors is the good weather

Interviewer1: do you have any suggestions you think that the state here can provide you with so people may be encouraged to do sports?

Interviewer2: or your suggestions for the ladies in your age group how we can encourage to do sport or any physical activity?

F1: as the winter is coming; Aspire Park is a nice place we go there to eat, people go for walking and we go for eating [laughing] when we go to Aspire Park we supposed to go for walking not to sit and eat. That is what I always try to say... When I tell them to walk they would ignore and say ok ok ok, I tell them “go and walk to lose weight!” they just like ok; ok; ok *Ensha-Allah* and then they all go. And this is something good [F4 interrupting: and the club…] but some of them don’t go to the club

F: weather around us help me to practice sport or not, some of sports are practiced indoors as heavy sports.

M2: weather

M3: weather,

M2: weather will be good

M1: weather, when weather is good you will motive to practices sport,

M2: the factors are the weather if the weather is cold or if football or jogging. Well a variety of sports

M: the weather

M2: the weather,

M1: the weather factor

Interviewer 1: can you please explain more to me?

M1: for example if you would like to go out walk around the house, you can’t guarantee, possibility the place is not air conditioned you won’t be able to move around, so the weather nature helps you move

Interviewer 1: --------

Male3: the young men take as vacation day and drive around with their cars. If you enhance running from this here to there, there will be amount of money as award

Male4: in the hot weather?

Male3: not in the hotter weather, let them make it in the winter time, you have more than one period of time, you have 4 moth winter.

Male4: to be active for 3 month and then ….

Male2: it is 3 month only

Male3: you will make a change with these 3 months.

Male2: you will enhance the sport activities

Male1: I have an idea

Male2: when I was in Malaysia

Male 4: we are not in Malaysia ( laugh)

Male 2 : when I was in Malaysia, I was walking all the time

Male4: because the weather was nice

M: fresh air, good weather that will let the person walks and to adapt to walk better.

Male 4: when the weather is good, we can go for a walk

M3: yes these are the obstacles. Why? You enter a room with A/C…

I1: ok, what make easy to you to practices sport?

F2: I think you should not ask in that way, you should ask in this way; what are factors makes you to practices sports?

I1: ok?

F2: weather to be suitable,

F: also the weather as sister said, the weather helps you.

F:When the weather is good I go and walk.

Interviewer1: and you what are the factors that make it easy for you to practice exercise?

F2: the first thing is the weather.

Interviewer1: The weather.

F2: The weather should be nice and this is very important so I can go out and walk. I am that kind of person where weather affects me, the sun, I cannot walk under the sun, or the increased hotness. When the weather becomes nicer, I can go out in the compound and walk for example.

F:The weather as she said

Female2: the weather

Nagla: how does the weather facilitate you to do sport?

Female2: if the weather is good like now, we will walk every day, thanks God. If it is in hot weather, i can’t.

I1: and you what encourage you?

F3: what motive to practices sport

I1: or easy to practice sport?

F3: as she said [f2] weather, the most important is the weather, when weather is hot it difficult to practice, when temperature is high you cannot practice,

M2: it is mam that the weather changes, the weather becomes suitable and not too hot, so you can walk

M4: air conditioning

M2: one would walk ---- at home ----- and walk

M4: yes the weather

Interviewer 1: you mean when the weather becomes better?

M4: yes the weather when it is good you can walk in it, if the weather is hot you can’t walk

M2: Especially in the winter, especially in the winter one can walk more, in the summer you feel it’s hard

---get into the car

Male2: first of all, what helps me to practice the sport is the weather,

Interviewer1: What are the things that make it easy for you to engage in physical activity or motivate you?

M1: The first thing is the weather, the first thing is weather, because exercise outside is better than at home or inside the gym. When it is outside the gym, the person will be more interested running outside than using only the treadmill inside which will make you feel bored in the end.

[M1 interruption: the weather is; hot the weather is hot].

M3: no not really now the weather is amazing, and what doesn’t make go for a walk is the humidity the temperature of the weather [M1 interruption: right, the temperature of the weather.]

Interviewer1: so these are some of the obstacles

1. **Time factor**

M: but If I was off one day I try to walk

F: or I have extra time

M: the suitable time,

M: Third thing is time, when time is available for you, that would be better.

M: So there is nothing that can prevent doing exercise, you can find time by doing it before going to bed or when you wake up, at last you will have time for it.

M: and maybe as my colleague said, to wake up early or take from his leisure time (break time).

M1: that is right

F2: and time management,

M2: Physical activity it is mostly the exercise dosage that the person take on the ordinary days or weekly and that is according to his functional (work) performance. Some people have the chance daily,

Interviewer1: What are the factors that help you practice exercise?

M1: if a person has nothing to do, he can go out daily for half an hour or one hour to go to for example garden , *Aspire* or anywhere, if he has a time to go and practicing exercise like walking

Interviewer1: what does encourage you to practice this sport?

Interviewer2: something if available it will encourage you to practice sport

M1: I don’t know but I say if the person has nothing to do.

Interviewer1: the time?

Interviewer2: the time factor?

M1: yes

Interviewer1: so the work’s nature!

M3: but in Sudan in your vacation, you can go and walk, can do sports

M: The time only, but when I am in vacation, I organize my exercise.

F: After that if you have the will, you will find the time, and you will find the effort and everything will be facilitated. He will find the time.

Female2: the time is the most important thing.

M: I have responsibilities, house and children but I can devote some time for that, I can devote some time to practice exercise. At night, I can practice exercise after I put her

M: I love to do that so I walk. I have around 4 cars but I don’t use them. I don’t take taxi or ride a car to reach mosque to pray as most of my friends do, they drive to mosque and I’m like “please walk instead of driving your car”; they reply me “no we don’t have enough time to catch the prayer”

F: is a good way of spending time and I recommend it.

F1: things that motivate me if I had time, more time I can do.

M2: so if the time was suitable I can do my work, we now stick only to watching a football match watch a match like that… but to do physical activity I think we need time and some morals (meaning high spirits)

M3: as the brother had said, it’s the time that is the most important. The time. It is the most important thing that the one finds time in the day that he can use to do sports wither it was in the morning or the night, the important thing is that he finds time when he can do physical activity,

M: So the first thing is finding the time

F3: time. If I have enough time; I’ll do it.

M5: the time,

M5: I mean that some people work and don’t find time to practice sports, they work early in the morning and they finish late so they don’t find time

M5: even the physical effort they provide in their work makes them unable to find time to practice sports.

M: then the availability of time,

M3: first of all the availability of time to do sport

M: relaxed and person has a vacation

M: to have free time,

M2: yes, because the person has work and duty and so he cannot do exercise. That is it.

M4: Adding what he mentioned, working time and the effort we do at work.

Interviewer1: so you mean boredom?!

F4: the place, time, someone to encourage you, you need someone beside you to encourages you, the place and time.

I1: What are the factors that help or encourage you to practices sports?

M3: the person should allocate certain time, part of 24hrs for exercise; he should put it in his mind, have time for it…

Female 4: The time.

Female 4: If you don’t have time you cannot make exercise.

M: to have time,

Interview: So mostly is the time

1. **Awareness**

M4: to have Sport culture person should believe on the sports outcome, that is the most important thing, other things are considered facilitating factors.

M2: The cultural role of giving awareness to the population about the concept of exercise itself and to maintain his body have big role to encourage the…

Interviewer1: It will make the person motivated.

F: I know about diabetic and hypertensive people when we advise them to reduce the sugar and do sport because this will reduce risk from heart diseases.

Nagla: what are the factors motivate you to do sport?

Female: avoiding disease, because they advise to walk to reduce the cholesterol. They advise me a lot to walk and thanks God I started walking.

M: the awareness when you are in-patients so many times you get afraid.

F: we are in health care field we advise people that they learn and that they walk, to cure themselves... one would be his own doctor. The man is his own doctor at once. Especially in Doha mashalla awareness on national level and even international beside; don’t forget we have national sports day that HRH Emir Tamim encourages, mashalla this thing is going good, even the non-literate man knows what it means to walk that it make the blood move in his body. So it is very important for the heart for diabetes for lots of things

Interviewer 1: What encourages you to do sports?

F4: what encourages me that I always read and I know

F4: yes eating ---- we are supposed to give attention to our children

F: We must put inside the girls the sense of sport and make it a habit. As we go shopping, it Is ok… the woman take her children and we will go for practicing sport etc.., Since childhood, and when she grows up, when she reach 24 years old, she will not be shy and she , no she will be happy and she is familiar with it.

| **No.** | **Ideas and suggestions to promote physical activity** | | **References** |
| --- | --- | --- | --- |
| **1.** | **Adequate Support** | 1. Governmental support (172)    - Physical environment (105)      1. Increase number of places (42)      2. Coach available to exercise (9)      3. Subsidized registration fees (9)    - Educational institutions and organizations(29)    - Role of HCC providers (4) 2. Group Support (35) 3. Role of Media (22) 4. Cultural support for women (15) 5. Family Support(4) 6. Family support (4) | **171** |
| **2.** | **Promote Physical Activity** | 1. Encourage PA since foundation age (25) 2. Increase the will toward PA (23) 3. Tournaments (9) 4. Balancing between PA and health diet (5) | **88** |
| **3.** | **Increase Awareness** | 1. Awareness in all organization and institutions (17) 2. Awareness at school (11) | **76** |
| **4.** | **Role of individual** | | **19** |

- **Ideas and suggestions to promote physical activity**

1. **Adequate Support**

M1: the most important thing is providing the necessities of this subject such as playgrounds (sport courts and fields) and others; sports halls, and these things to be present. That’s it

M1: yes there isn’t something else.

M2: same thing, the covered halls that will... that help even if the weather is summer; one could walk because it’s hard when you will be totally wet, so if there are more halls and are distributed

M2: yes that would help better

M3: same thing; air conditioned sports halls especially in summer, in winter there is no problem

M4: same thing

M2: for example mam there are schools… I mean part from things that could include old schools the government had leased them to new schools and all of these schools are air conditioned, so if these schools were changed into sports halls that would be a good thing, it will also provide a place….

M1: the most important thing is…there are schools, clubs..

M2: in these schools, there are playgrounds and this can be the area to use

M1: especially if there was in the place something that encourages you

M2: exactly yes.

M1: I mean on your own you would get bored but when you find other people with you present there

M2: or use the schools during summer

M1: you would be encouraged to practice

M2: in the summer there is no studying and schools are closed, so they could be opened for people and make in them... let walking and running be practiced there specially in the area of ----

M2: or all the schools

M2: its only the schools suggestions

M1: the most important thing is to make swimming pools

M1: because this process is present and the sea is a bit shallow ---- so things to encourage people

F: so exercise is important so that one stays healthy even if her reached older age.

F1: I suggest for example each one has a sport hall in his house, or in compounds there be sports hall or in neighborhoods, each neighborhood there would be places they call them green areas for people to walk or play.

F4: make groups for example that would encourage each other; volunteering groups, the girls gather and form sportive clubs for them, volunteering groups encourage people, pass by people and encourage them, they would have a logo or something that would encourage girls and don’t get discouraged. They would be the main point.

F: for example. Or they would increase the number of contest like the biggest winner; add more contests; another contest for losing weight, another contest for fitness, add more sports; football, things like this, there should be something that would encourage us to do sports. Not sitting and using the bus to go to another building and coming back. There is not any movement at all.

F1:….. [Laugh] I don’t know, if each person starts from himself, she said she doesn’t have time to practice, so they can allocate time to practice at least 30min sure they have 30 min free, for me I have so many free hours, I do have extra time and never get use it, if I walk daily 30 min around house it could increase gradually until became a lifestyle, habit to walk every morning when I wake up. Gradually person we will be able to practice.

F3: you can take it as entertaining such as to go with friends and use bicycle, so it can be entertaining and exercise at the same time so the body can get benefit from it. You can walk from building to other even though weather is not helping but at winter you can do at the university. That’s all.

F4: I’m with providing center that is specialized in promoting exercise, a club for young females only for example, controlled by a special department and professional coach not any person to be there,

F: and to be around with people who like movement and practice exercise, this is for me.

M: or let them , at least; providing rooms with AC or specific halls for this thing.

Male1: for example the advertisement “how did you look and how you look now”. This is the first thing they do in the gym. From day 1 in the gym, you take a photo of yourself, ok? And after 2 month take a photo again and look the difference, after 3 months look the difference, after 1 year or 2 years, look to the first photo and the last photo and look to the difference. When the people see what you made to yourself in your life, this will encourage them to do what you did.

M: secondly to provide them the suitable environment and suitable time.

M: Thirdly there should be trainers in good standard with the students or young people to prepare them for doing sport.

M: Secondly I suggest putting a day for sport. There is sport day here in Qatar put other Arabic countries don’t have sport day or… if you look to the gulf countries specifically they care about the sports but they didn’t reach the desirable goal of the young people.

M:So, there is deficit and the reason for this deficit is that there is no encouragement for the young people like putting advertisement for them in this aspect.

Male4: motivate the young people more for doing sport.

Male4: because a country like Qatar, it has a lot of sport activities but there is shortage in motivation and make the sport likable. The sport awareness in general

Male5: through competition like in the *Aldori and Alkas* (T.V channels).

Male6: young people are always together, if you target some friends, they will come all. For example every sport complex target group of young people and talk to them in this subject, if they are all together they will all go together. So that may help the young people.

Male6: yes. Targeting the group who stay together, they are usually friends

Male1: there are universities here in Qatar set up a day for a championship for all the sports. For example there is volleyball and there are 20 teams in the universities and there is playoff between them. I heard about the strong man. They started with light weight with 15 participates and they start to elevate it. Then they increase the weights and elevate until they reach the last 2 or 3 participates and they playoff until they reach the maximum. They give awards and academic recommendations and you will have it together with the graduation certificate.

Male2: most of the young people don’t accept that the sport is … it is rarely when you find it very few. If you see the volunteering work in Qatar who did it start? It was rarely when you see the Qatari young people go for volunteering like volunteering in Qatar volunteering company or Qatar University they all go why? Because they know their own benefits from that and they know there are experiences and relations. He will improve himself. It should be the same for the sport, motivations and benefits at least to be financially or something let the young people care about it. After this stage, the young man spontaneously will say no need to take awards every time. When he adapts this, he will do sport spontaneously.

M4: in my opinion the best ~~the~~ thing that motivates most is the availability of sports clubs. In each club there should be someone who will give you the correct awareness. When you are doing exercise alone you can do something extra or decrease an activity, on the other hand, when you are in a club and there is a doctor who knows what is excess in your body or less, he would also know how many time per day for exercise you need so you won’t feel exhausted.

M: Regarding the shame point for women or anything, there are women sports club that are only for women that are not mixed and they can practice exercise freely there.

M: To increase it at schools, make PE classes more, increase the importance of doing exercise at school. For example, for me, at my school, if there was a program after school, an exercise program, that would be the beginning, from here. Let say leave the school hours, the school schedule can finish at 1:30-2:00pm, and for a certain days in the week, the student can stay from 2:00 to 4:00pm even till *Maghrib* (afternoon 5:00pm), there is no problem, there is transportation where he can go back home and at the same time he is under school care, he can be in the exercise programs, games. This is because the tournaments at schools in different sports is there so if we put something like that at schools, there will be challenge and this will grow and the student will have the talent and in the end this will appear as all the people would be doing exercise and the awareness will increase. In addition, the country will find talents in different sports; this is because it started from school. Physical activity is 2 to 4 hours per week. You can bring up a talented footballer who would be the world champion in football, for example.

M2: We can encourage them through games, when there are more games, when there are more games there would be more motivation. Like in *Katara*, there was a game that was done in *Katara* and there were lots of people there.

M: Besides, providing large playgrounds on a large scale in this country and not like any other country because *Masha Allah* this country has this, also at the same time, you might not find a place to exercise, as a game not like walking, you can find a place to walk. Providing the place is important.

M4: When the country comes and motivates, this mean that it can, when a country comes and encourage it means that it can give awareness. Awareness is an encouragement to exercise and it can motivate; and motivation can be by founding playgrounds; bring trainers and things like that. But awareness (alone) is not often practical; it is theoretical in portraying pictures and things like that. If the country’s budget that it will spend, if it is going to spend it in one area, my opinion is to, motivation is more important than awareness because some people know they want to be so, like students at college, each one drew his future, I, for example, will graduate and will take this, this and that. He has the awareness but where is the motivation.

M4: where is the motivation, the movement? You know where you want to reach and know that, for example, I want to be a world champion; I want to be, to take medals in this and this. Ok, you know, but where is the motive? Motivation could be through clubs,

M: Through *Instagram*, the social media, we started to see pictures of this, we would say wow this person is strong, this exercise is what? This is this type of exercise, ok, how do I reach this level? I go to Gym, ok how can I do that? Vision, I saw those who surround me are like that so I started. So these things open a big door.

M5: for me regarding the suggestions, for example, the time that we are now in, most of them go to hospitals and health centers, and they treat with medicine and others, meanwhile the disease like hypertension and diabetes and most of the diseases that can be treated mostly by exercise. Hypertension can be treated by decreasing weight, which will decrease blood pressure and pressure in the arteries, for example if this patient practiced exercise, he will decrease in his weight and so will increase in the percentage that he will get rid of hypertension or the percentage of not getting the disease. The health centers and the hospitals should add the exercise part in them not only the medicines, because most medicines have side effects, it is used but has side effects, so we as health care facilities, should add this part as an exercise program for the hypertensive and the diabetic patient.

M1: first of all “Healthy mind is in a healthy body”, second of all provide places for sports only across the country, and to tell people to practice sports for your own health.

M2: I think once parents have children or the kids reach 10 years parents should implant love to be something, if kid says I want to play father should say yes I will take you and make his kid feel he is doing something right, so kid will have the impact of love to play, to do any correct thing.

M3: I think to increases trainers at gym, they should be available for the encouragement from the trainer to the youth not for financial issues.

Female1: it’s not from government it personal motive, exactly if person would like to do sport from time to time he will do it. if he leave it for a while and return back to practice f he will do it .You don’t need someone to tell you do sports or motive you, it’s from yourself if i had time i will practice, i I’m able also i will practice also , so if person did not want to do it he will not do it.

F: marketing for other clubs, they should give people other option so if that club not suitable you will go for another one,

Female4: organize your time, prioritize first of all, Allocate Time for mental rest, because sport play major role in reducing stress ,anger, Psychological pressure, the sport relief all of that then you see what you like ,

F: there is not advertisement, so I need to search and ask for the place suitable for women. Sometimes it is not suitable in time or the coach is male and not female, this would be difficult. These are things that tie you not to practice.

Female2: i greed with her in the lack of couch. They provide gym but will find trainer by appointment , for example first time for me at gym i don’t know how to use machines and what to do exactly , and trainer will come and teach on such machines but you still don’t know what kind of machine you are working on. They should provide for each 3 individual a special trainer

Female4: to make awareness and event at universities and schools or even in ***Montadayat*** (social media) , posters on physical activity and what is the benefit at the mall about or events on physical activity that talks about the benefits of sports, to make people aware. Physical activity helps to focus, and how person can continue his life along with physical activity.

Female1: they can provide for us places at university, in truth we have gym but we don’t have a female trainer and she will not be available all the time for us , no time, also lack of spaces at gym which cannot fit all university students

Female1: yes the spaces is small and not enough for all student to motive them. The machine also limited, to limited. I will not find all kind of machine i need

F3: they can make for us clubs, [interruption from f2: yes particular clubs for women)

F3: gym, as there is gym for men’s they can help women and do gym for them, gym should be on the expense of country, so instead person will go and pay money on gym country should provide that centers ,clubs for women[ interruption F2: yes for Women]

F: to open clubs, besides that they should provide garden for women only so they can run with their kids safely

F3: if you see open doors play grounds most of them are men especially youth, so woman get (Harassment of young people), to encourage people to do sports, open clubs for women’s,

F2: to do competition, marathons for women only, particular places that women can use it to jog

F3: to help and motivate

M2: I told you about which is the infrastructure when it is available near to people houses .this will play an important role.

M3: of course, it will encourage

M2: This plays a very big role in that, when the club provide places for example, *Qatar Sports Club* may cover the in *Dafna* area. If there are trainers who would have programs that help in giving education, it is not for young… and age group issues [*Interruption by patient asking for the doctor min 10:33]*

M2: I say this as an example.it plays an important role even if it is late, he can find an hour or half an hour, and things like that.

F: I have noticed that when I was on vacation on another Gulf country, I have seen the ladies wearing trainers and they were at mall. They turn around the mall with AC and cool. It is cheap and cost nothing and you can leave your children in a place to play in or to leave them with the housemaid and you turn around the mall. If you turn around the mall 10 times, this will be …..

Female1: I have noticed that they opened centers, big centers prepared with everything, if they make a small room for gym for the staff. This may help people and it will not cost a lot. A simple thing like treadmills, steps. Simple things and with the staff… after the work and before she start the work half an hour before it, she can do it with her friends, some exercises and start her shift this may help people to activate them at the work place and will not go to a distance places. These are simple things and I think it could be done.

F: The mall also, because the mall avoids the weather problem.

Female2: provide gardens, with walking tracks for only ladies. We can put our children to play and we can walk at the same time with the family.

Female3: in Qatar there are many centers for youth and cultural centers for ladies. All the cultural centers here depend on the seminars and meetings, etc...there is no part for gym; as the doctor said, for … as an extension to it. If all the cultural centers accompany with small gym room for free to the people or participate with few money, this will encourage them. It is not only for medical staff or the employee in the health center, no, also in youth centers. Here they care more in the centers for the men, having swimming pool etc.., but the same youth center in woman branch, the youth centers of men branch and woman branch are completely different. The woman branch is only for meeting room, lectures and etc.., but there is no gym, no swimming pool, nothing like those. On the other hand in the men branch there is football playground and tennis playground and playgrounds for many thing including swimming pool and all of these… if there is one for ladies, that will motivate them to go and it is for free, participate and can go every day.

F: If the weather is hot and we can't go in the garden, then the youth centers for us will be ok.

F: We must put inside the girls the sense of sport and make it a habit. As we go shopping, it Is ok… the woman take her children and we will go for practicing sport etc.., Since childhood, and when she grows up, when she reach 24 years old, she will not be shy and she , no she will be happy and she is familiar with it.

F: So they need to provide these things and what they provide from activities to the boys, they should make the same for the girls, so they will be involved in that. They put that inside them and this will make big difference since childhood.

Female3: to make our duty lesser (laugh)

Female3: if there is extension room for gym, as the doctor said, in our health center. Instead of eating, drinking etc. in our break time and that is it we can do sport and that will encourage us too. that will motivate all the staff.

F: but the governmental centers are mainly for men. For example the *Zakirah* youth centre, you will find in the men department swimming pool, playground, There all the playgrounds in the men branch. In the woman branch, it is a villa and normal room where they do meetings and seminars memorization *(mostly Qur’an)* it is all on seats, there are no activities. It is only once they go to men branch in a month. It is rare. If the branch itself opens for the girls all the time, there will be chances to…

Female1: there is a very nice sport, swimming, if they bring a lady to train the young girl for swimming, together with the mother, this will be make the girls busy with it, because the sport will make the young people busy from internet, mobiles, movies and games etc. the sport is very very important.

Female3: as I told you, for boys they have activities and they have football. You find only one place for girls and it is in Doha. What about the population of all these places, where should they go?

Female1: The mother can take her daughters and go!

F: I try to walk, because if the person falls especially the elderly, it is over, if he stayed in his place, he will be sluggish and the whole world’s diseases will affect him. He has to walk, if he cannot do exercise, he should walk, walking is enough, walking is enough. He will burn some of what is in his body, this is for the elderly. There are some people who would say we passed this stage and this is for the young. This is not for young people; this is for every person who has a live body, his back is alive, his knees are alive, it is not that they are old in age, then that is it, I keep aside all the organs, no. I should give them a space for that; if I cannot exercise then I should walk.

F1: The country can provide a club for the elderly

F2:that is right, they can provide a hall in the districts

F1: provide a club for the elderly so they get encouraged.

F2:They can provide a hall for women for example

F1: women and men.

F2:They can do that for the age 45 and above with trainers and with small registration fees, because not all people can pay expensive. They can do that for a month of 45 days for a group who will encourage each other, so I and the sister can go and know each other and this will encourage us that we are going on this day to the hall to practice exercise. We have this hour for exercise, you will feel nice when you are with people and also you will forget about your pain as there is a specialized hall for women of this age. Of

F: I previously have seen a video of elderly women of our age and heavy, they were walking on the treadmill with music and you see the trainer is active who is giving you the energy and the push for you to move. Where are we? They are women of our age or even older like the sisters here, you feel they have energy and they are active, where are we? How are we living and how they are living? Look they live longer not like us [laugh]. When we reach 60; women will not be able to move, sitting on her chair, and she became obese…

F3: Also on the State level

Interviewer1: Like what?

F3: they can provide us with clubs and things like that; we go there and come back, chat and drink coffee and do this..[Laugh] without coffee and tea it will not work.

F2: Subhan Allah, you feel company when you are with people, and take experience from older people.

F4: I have the same idea, to provide places for exercise not necessarily clubs, there are open areas and public gardens but they should provide us with specialists, when you all gather, there will be someone to encourage you. He would give you the movements to do according to your age, and your health status and so give you the correct movement to your age, the body movement that would not make you tired, according to your age and they diseases you have, so they have to be specialists available to assist and support you. You can go back to them and ask them for the movement that suits you.

Male 1: on my point of view and for the football playing if they make more playground for public

M: There are many playgrounds but it is with registration and it will be crowded

Male4: honestly there are many places to play sports

Male 1: there are *Aspire and Katara*

Male 4: you can go to cornich for walking

Male 2: if Qatar has a lot of money, why do they take us a broad for sport and losing weight, to other countries? Take number of people, number of people every time, 30 persons, 40 persons. Take Qatari only. This is my suggestion

Male1: it is nothing really, This sports day became nothing really.

Male3: yes it is but

Male1: people don’t go anywhere.

Male2: it became like the mother day. To bring a gift for your mother and you take the money

Male 1: That is what they think

Male1: this day doesn’t increase the sport in the country, but decrease it

Male3: the young men take as vacation day and drive around with their cars. If you enhance running from this here to there, there will be amount of money as award

F1: to provide the appropriate atmosphere; like proper places for the gyms, here I think we have Aspire for females but we don’t have many places for females I think here in Doha

F3: or they could have activities that includes all the members of the family. If the mother likes to take her children and her husband would be there with them, of course they will be convinced and would like to join if there are such activates.

F1: they should make us like to exercise; I think, for example some people don’t like movement because there are some difficult ones, they can make it easier for us… they can bring ~~by they want~~ trainers that would show us exercises that are beneficial for us that we can do at home, because there are lots of people who don’t know that.

M1: I prefer sports; gym and such, closed halls, with AC, helps one to be able to move around that’s it.

M2: for me I say this will not be from the people, it should be from the Country, the country provides a suitable day for a sports day, but not one day in the year, it should be more than one day as a vacation. I have another suggestion

M2: I want here I want here in the country for Friday and Saturday to be days off

M3: yes right

M2: Friday day off and Saturday.

M2: you would provide suitable time for all people to do sports, what shall I do on Saturday? Stay at home? For sure I will go out to walk, I might not necessary go out do sports; I will go walk to my friends this is sports.

M3: the country should use its power; first thing to explain the aim of doing sports, force the private sector companies since I am working with a private sector I used to work even on Friday; force private sectors not to force workers work on Fridays, they might not refuse it but they leave the choice for them; and not say: if he didn’t work on Friday I will….

M3: yes, force private sectors to have periodical checkups for the employees; blood test, I can do it on my own I move around normally I don’t complain of anything, but when I find high blood cholesterol, the start of a problem, the start of fat on liver so I would fear for myself so I would go to sports, I know that doing sports would be the only way and to organize eating, so I put point which are regulatory for private sector that at least make Friday a day off. Obligatory, and after that it should be implemented you would go to the employee ask him he will say he is working Friday because he wants but it is not true, he is forced, not directly but he is indirectly forced to work on Fridays

M2: ---- and the time,

M3: yes, yes

M2: providing the time

M3: and at the same time we can apply, the work law can be applied in a way like; what is the official working hours? 7 hours? Make half an hour walking as now we have a break from 11:00 to 11:30

M: but the most important thing is providing the time.

M3: providing the time that’s it, whether by a weekly day off or taking a period of time from the official working hours for example half an hour out of the official 7 hours or 25 minutes or 15 minutes to practice sports even if in the same place it is not necessary

M2: move in the place

M3: yes yes for sports. One day a year is not enough

M2: to provide day off.

M3: it is off day, they have to give a day off for you to do sports, besides, when they give him off day he is not the only one who will do sports; it him and his family, for example my wife is staying home she wouldn’t go out except when I am there so now I will make her do sports and my kids do sports the whole family I mean, so the day off will not be for me alone; it will be for me and those who are around me. So this is important because there is no time so he wouldn’t do sports.

M1: I will say 2 suggestions first one; one day off a week not necessary to specify to make sports no, specify one day off, naturally this day off I will not spend it at home, I will have to go see my friend, I will walk a bit, So I did exercise without feeling that I did, this is one thing

M: the second thing; the clubs, if I am talking about exercise so the clubs, this is a suggestion to organize parties, sports games. That is it for my suggestions.

M2: for the clubs, this activity is present -------- that here the clubs specify the day that you go to do the training not the day you want to go to train, so for example you have Al Khour club,

M2: you have Al Khour club, if you went today and made a membership, they will specify for you to come on this and this days you can’t come on this and this days, why? For example the gym hall, swimming, football, all in appointments

M1: Swimming pool has certain hours.

M2: how do you make appointments obligatory and then you tell me come and practice sports? I like to do sports, I like to do it every day, I have half an hour free, I will come. Okay? But you are forcing me to come at certain times, so this is not membership

M2: you make me in the first place if I want to do sports you make me get lazy

M2: the sports are not given enough, sports generally; there are a lot of clubs in Qatar there are a lot of sport activities inside the clubs but unfortunately it is not used because of the routine that is set you come on this and that days don’t come… why? What is the reason?

M2: I am one of these people I went to apply in the club, I found him handing me a table saying this is the timing table

Interviewer1: timing table to work with,

M2: I told him no of course I am not free on this day, he said: it is not my problem you come to me later

F2: sometimes you help by advising them to move and walk instead of staying at home. Try to move and walk; try to do anything.

Interviewer2: so you think movement inside house can be considered as an exercise?

F2: yes it could be considered as exercise. [F3 interruption: for me I don’t consider movement at home as an exercise because you will not burns enough calories; you got my point]

F2: but movement at home is nice.

F3: well still it’s better than sitting all the time at home but still it’s not an actual kind of exercise. It is supposed to be another kind of sport rather than house work. Something with more movement; like swimming for example; you know. Swimming is one of the enjoyable activities.

Interviewer2: ok according to your age group what do you suggest for them?

F3: at least go for walking.

Interviewer2: walking! [Other participants also agree with F3]

F3: walking is the easiest thing people can do. If the weather is not nice and can’t walk he can buy the walking machine and use it at home. He can even put it in front of the T.V so to get motived to use it.

Interviewer2: and if we asked you regarding your job how you…any ideas?!

F1: even if it’s inside home. And if they have a big house they can walk around inside. So what is wrong to walk inside as they have a big house!?

Interviewer2: ok on the base level of the work. What are the things that if they provided to you at work that encourages you to exercise?

F3: Wow! What a beautiful thing if they do that… [Group talking as they all agrees] if they can provide a break time so we can go for the GYM and you can move a little bit.

Interviewer2: so you suggest providing you with GYM hall full equipment.

F3: exactly; so the break would be for exercise. You got my point! You have a certain time just to move your body.

Interviewer2: so it’s not necessary to use GYM outside the work place?!

F3: yes exactly. At least 15 min her and 15 min at home for example to do something. It really became something nice, it will really help.

Interviewer1: do you have anything to add regarding the sport; any ideas? For example your colleague suggests providing GYM hall room in the work place and to specify a certain time during the work to exercise that would be better?

F1: I believe when we get to know people, they would go together and practice the same activity or something like that.

Interviewer2: you mean to be grouped.

F1: yes.

F3: yes, because the company sometimes is the reason to get fat or being slim. Have group encourages all of us to lose weight; did you get my point.

F1: even her at work [F2 interruption: right even at work] they can ask us for a walk or go to this room for activity or so that would let to move.

F3: or this watch for example. It counts the steps for you.

F3: so it motives you see how many steps you walk and encourages you, if you did not walk that much steps and so on. it helps.

F2: it’s right what you say (F3) it is a good choice

F3: you will be alert to move especially in the weekend as you don’t have work and you will not say that you have work or something. You can’t make the work as excuse that you don’t have time. So you supposed to have time to move. You know what I mean.

F1: the suggestions are to increase the number of health and sports clubs, and sometimes the fees for example to be reduced, sometimes could be encouraging fees not commercial, now most of the clubs; women wouldn’t go because the fees are commercial. Groups; for example each group in the neighborhood for example each group in a certain area there should be a sports club a special club for example for ladies, you will find all encouraged, otherwise all clubs you find them far away something in Doha, further. But in each area, if there is a sport club it will motivates that all people in this area to practice sports.

F1: and low fees.

F: Another thing as she had said increasing the number of sports centers in many places for example we are living in Al Wukair, inside is the center so in all al Wukair there is not a single sports club.

F1: there is

F2: where?

F1: the closest is in Barwa

F2: there is one in Ezdan I don’t know god knows if the word is correct or not. So imagine we have to go from al Wukair to Barwa

F2: if the place was close or would need 5 minutes to reach it this would help us

F3: the workplace

Interviewer 1: inside the workplace?

F3: yes, to do it during the break time,

F4: of course, you are talking about governmental encouragement? The country encourages sports and the centers are available everywhere thank god.

Interviewer 2: she means ---- are 2 types; on personal level and fits the same age group, and nation level for example there are female workers there are ideas and thoughts about them on the national level would be general

F4: yeah,

Interviewer 2: so what ideas (thoughts) do you have?

F4: national level of course to join the sports clubs by hours, take one or two hours of our time to practice sports; swimming or walking or using sports machines and weight lifting the ones that are light in order to strengthen your bones, on work level I suggest the same as this sister said,

F4: that what? The sister here said she wishes in the work place to be there a special place for sports so that one wouldn’t get bored, in the time of the break he could go move in that time, first thing he will refresh his activeness and Sobhan Allah sports give you new thoughts and new productivity and makes you fresh so this helps better in regards to productivity at work and not being lazy

M: So I say if the person keeps praying regularly at time with other Muslims this is sport it burns so much calories and feel a psychological relief.

M:The government provides like treadmill machines and you can do that once or twice per week. I used to be committed to it but then the time issue came up where I couldn’t use it any more. It was really useful for the belly part. Even for breathing, there were times I couldn’t even try to (participant was pointing to his socks)

M2: I strongly advise them to do sport

M2: to make timetable so to follow and be committed to it

M1: my suggestion in general to make activities constantly, to ride bicycle, to jog, marathon

M: provide suitable facilities and places to jog, preparing the suitable ground that all.

M2: first of all the Supreme Council of health they don’t have activity I mean places for exercise,

M1: to have extra time, I work at company, they have branch in Dubai, they have a branch where when staff have free time they practice exercise. So they do that in during their duty

M3: for long shift duty I prefer if they will provide places to practice during resting hours

F1: as the winter is coming; Aspire Park is a nice place we go there to eat, people go for walking and we go for eating [laughing] when we go to Aspire Park we supposed to go for walking not to sit and eat. That is what I always try to say... When I tell them to walk they would ignore and say ok ok ok, I tell them “go and walk to lose weight!” they just like ok; ok; ok *Ensha-Allah* and then they all go. And this is something good

F4 interrupting: there is a place just like Aspire but the main problem is there is no space. The club supposed to be spacious place. And a private place for ladies and kids. [F1 interrupting: the fees are high and some people cannot pay] the fess is low but nobody pays and there is no vacancy [F1: interrupting: and people have no time] [F1 interrupting: what if she has no car and her husband is busy?! Aspire; all the people like to go there it’s a known place even in Thursdays…]

F4: because you are alone you don’t know and you want someone to get motivated; Lets go and lets do [F1 interrupting: why don’t you gather there; Aspire is a known place and everyone goes there you can gather there. Everyone goes there to Aspire these days. It’s good to go for a walk even these days the weather is beautiful. We always do that].

F3: for me because I have kids, and I am very bonded to them, frankly speaking [F1 interrupting & laughing: we always like to go to Aspire]. It’s a responsibility, you get me. How come I leave them alone and go for walking. I can take the older kids but what about the little one, poor him?! [F1: interrupting: yes right] you feel he needs one more year... You get me... This is the first time for me with this kid, in every pregnancy I do diet but I didn’t do this time as I feel like I have no will to do. I get lazy [laughing & door opened] I swear every time I do and like to.

F1: Aspire Park honestly is the best solution. Everybody satisfy with it. [Side talking and door opened] we don’t want to go to malls; you know what I mean, its better if one takes the advantage of this weather!

F3: Yes the weather.

F2: area for sports, beside the clubs. True there are centers for exercising but these are not provided by the government. It is not the same as the club where you can go at any time. Soany place to practice sport for example clubs for ladies same as clubs for men.

F4: Same thing in addition to bing close to those areas.

F3: the same, provide the alternatives, for example: people not doing sport because of long distance and time factor. Provide clubs in each area. Provide everything and should be for ladies. I think this will be encouraging.

F 4: the same, clubs.

F1: the places are far away.

F: it should be mandatory for the student. Some employee in their beak time. Encouraging procedures for the employee and to be mandatory for the students.

F3: for the employee, for example in certain time according to their work times, as I mentioned, near places and provide the suitable environment. For example if the weather is hot, it should be inside, this would be encouraging

F3: yes. The same thing This is the encouraging.

F2: when the place is near you can reach very fast but when it is far, no.

M4: I have suggestion, if any one from the doctors here is obese to quit from his work,

**[All laughing]**

M4: yes, the doctors should be the first one, I see one who is obese and like this, he is a doctor.

M4: My first suggestion is they should make for us logo and start on it, so they will be the role model*, a quote in Arab society says ‘the door of carpenter is broken’*, this cannot be.

M2: that is good idea

M3: how will motive you as foundation, hospital?

M4: they can make advertisement.

M2: They themselves

M3: and as advertising they can add health education, so if someone forgets other one will remind him

M4: there should be a club in each in each village, the brother said there is a lot club, there are not so many clubs in the area, and each village must have a club to practices for free so the people of this village would go for free.

M3: there are gardens to walk.

Female 5: For us in the work they should put the one hour break to the exercise since most of them they don’t eat or they use only ten minutes from it because most of them on diet. I which If they can do like the other countries.

Female 1: outside the center there is a big space you can walk on it. Thereis available place for walking other than the centers. Even in Aspire the gardens are everywhere and between the neighborhoods there are gardensand walking trails. I don’t see any obstacles.

Female 2: Every neighborhood has garden and available place for walking. And even without the garden some streets have walking trails that written on it so the cars don’t come on it.

Interviewer1: For you what is the suggestion?

Interviewer1: has to encourage himself. How we can encourage our self?

Female 4: to provide the exercise machine with reasonable prices.

Female 5: privacy for women .Also the public machine should have some privacy for the women. When you go to Aspire and its empty you can play there but in the presence ofsomeone it’s difficult. There is walking place in Al-Rayan but it has been closed; it was wonderful. Al-Rayan walking placewas more comfortable than Aspire.

Female 6: Al Gharafa is better.

Female 5: It was walking area when you walk.Everyone is walking so you get the encouragement. In Aspire you can find some people are sitting, children are playing; you scare to hit them. There wasan open space for playing.I used to prefer if there is close one for women; we had one but they closed it now. We want special place for women wearing*Hajab*whichit is difficult to take it off in front of the presence of male. I like quietness…The sports machine sometimes helps me.

Interviewer1: Is there is any suggestion?

Female 6: availability of the sports machine with reasonable prices. Make more places especially for women.

Interviewer1: and you what is the suggestion that we can offer or Qatar states regarding the sports?

Female 1: Regarding the sports here*Mash-Allah*they did their best. They did it well. They create the “Sport Day”.In the school the sport class became very important. The boys are very keen about it and the teacher as well...

Female 2: The obese kids like my daughter and my daughter-in- low*Mash-Allah*they register them in Aspire. They sent us the paper telling us they want them to join. They started to follow and check-up for the diabetes. Honestly we thank them the schools for that*Mash-Allah*.

Female 3: Even the teacher told me that your son is fatand he has to join...but she did not register him. She told me he has to eat healthy food and since that timewe stopped eating eggs, nuggets or burger and we start to eat healthy food. She told me when he grow up he will become tired. He is in the first grade now when she saw him he used to be fat although the food at the school is healthy.

Female 4: I will complete what she said even in the school they don’t sell candies, or chips or Pepsi for children. And then they sell things with good advantage like cheese and also the fruits.My daughter told me that they sell fruits and vegetables in school.

Female 3: apples, cucumber, carrots all available in the school.

Female 4: all of these things are good they encourage the children themselvesfor healthy food may; God guide them.

Female 1: Suggestion is like; the clubs should be special for women and the working hours should be suitable for the employee.

Female 1: through clubs.

Female 1: Clubs or educate them through sending SMS via the phone and participatethrough places like this conferences and lectures which is encourage little bit. Especially the old women who sit at homes, I have my mother in lowwith me I always ask her to go out and we registered her at the diet center and then she used to go and thengot laziness. You know the elderly women they got laziness when we go back and force.

Female 1: yes the distances and walking but the opposite the old women but we are thanks God even I would love the older so that would not effect on their aromatize. Even when I go for walking at Aspire I see even old women a very old, oldandwhen I see them I get encouraged. Some of them are grandma and they circle around wearing sport shoes they do not walk with normal shoes. At the opposite I get encouraged more and I walk more. I can say Aspire;thanks God for the walking space

Female 3: If they increased the clubs only one club.

F: It is better to increase the numbers of clubs or designated place for sport. For example, put small club in each park to encourage people to practice especially here in Qatar I cannot see.

Female 6: yes if they are in group from they will encourage each other especially males,

F: It is better to encourage this physical activity in the health center. It is also good if we as group together participate in activity and make it as competition between us as a female until we reach ideal body shape. To have fitness; this will increase my strength to continue practicing activity. I need somebody to encourage me, if I do not have any one of my friend with me in the club; this will discourage me to practice physical activity. I will think why I am going to participate alone and to go to the club an hour before the lesson start. I will say no, I will stay at home and bring machine at home to practice the activity. Then when I bought the machine, I did not use it.

F: The company they work at it they should support it. Well when you see employee in a good health and enjoying the lightness and move that gives them more energy. If I am the owner of a company and I found an obese employee but his working is good and his brain is very high this is jewel I cannot lose him. He will not give the production but his brain is good. I want to utilize his brain in the job. What should I do? I will support him to exercise. Guys we are enrolled the company in a center with reasonable price so that the employee will pay it. This will encourage the whole employee to be in group and they will noble competition between them to make encouragement to make them to give energy in the morning and at the end of the day. They feel it’s a recreation not like a type of routine. No this journey I don’t want to go. If my friends are going with me and this one his weight is that and the other is thiswhy I am like this I have to be diligent to reach.

Female 1: I hope in any public place at least they should have park, gym, exercise machine.

F: To encourage someone who wants to do exercise any he doesn’t have machines at home or they can’t buy it. So it should be free or could have free membership to do exercise.

F: I feel the prices are different instead it should promote health and should be reasonable for people.

Female 3: The same thing, there should be gyms that everyone can join and should not be expensive so cannot join.

Female 4: In the university should have sport hall for the student to join it.

Female 5: It should be near the homes.

Female 6: increase the numbers and near homes and available in any area. There should be section for female different males. Not only fitness but also the numbers of park places, you find Aspire or Cornish is for away from your residence.

M: Wallahi to increase the number of playgrounds, makes it bigger because it is small

M: And try more to encourage the youth for sport like the National Day of Sport

M: well it the same idea but we can make some advertisements; awareness groups for the importance of sport for human.

M: Also they can make bigger athletic supplements

M: And also it should be a nice place to attract youth for GYM, you know there is GYM that is well designed

M: Also if they provide more athletic supplements also it will attract people to them.

M: an awareness campaign for example on the T.V or advertisements about the same idea that show the difference between the guys who always play sports and the guy who is always lazy, you know an encouragement, encouragement.

M: They can make a special building or a special club, a very big building in Qatar just for sports games, there is no GYM just only games.

M: so by increasing these public facilities like a football playground either made of grass or a halls, the same thing some people love the big playground and there is the normal one so also increasing the number of the sport public facilities and the advertisement.

M: we can make champions with prizes, advertisement. And that it is

Male 1: *Wallahi*! What can I say; first of all here in this country let them try to change their rules in those clubs because it hinders us honestly… well these are. If you were not born in Qatar it’s so hard to play with them …. They prevents... I mean the majority of the people in this country are… [Male 6: interruption: I think in each team they are 1 or 2 or 3 …] [Male 5: interruption: no they are 3…]

Male 1: yes 3

Male 6: no but there is like in the relay races and in the individual’s games... It’s allowed to have 2 players in each category age groups in each team according to the sports club’s laws.

Male 1: so this is a problem. Almost all the Qatar’s populations are foreigners

Male 6: no no; I am a Vice Chairman of the sport club and am an employee here in Qatar University – specialty of sports; so I know all these rules.

Male 2: really we suffer because of these rules. We don’t know…even if I would like to go the club to play and to be encouraged; you will play with them 2 or 3 days then they say to you by the way we’re not going to registered you with us. It’s like he broke the motivation that you have he just broke it so you go home.

Male 6: and the people who set the rules regarding the age group is the same thing… the people who set the rules regarding the age group the put it as an obstacle, where I don’t see there is no certain age to play sport.

Male 1: even it’s not necessary for these clubs to made rules of being a Qatari or not and so on... I mean if they want so they can make a foreign team just only for foreigners here in Qatar and let them play against each other and to go out of the Qatar it’s just. They are foreigner they shouldn’t go out from Qatar but at least they can bring the best players put them in one team so they can make champion [Interruption and group talking]. Honestly why they prevent it because they want to increase the number of Qatari’s players and then they can reach to the national team and can bet abroad but we’re the foreigners here, they made playgrounds for us but sometimes we need little motivation, some champions here and there so we can…; so if they can reset these rules even if… so they can make more champions and increase the number of the teams; let them bring some people to play so.

Male 6: I would say not to bring foreigners for example we have certain sport category where it can all be foreigners so this thing would lead not to love the sport… there will be one strong team and all the others teams are weak so the competition is know from the beginning not to bring foreigners and relaying on the local players.

Male 5: For me there are suggestions because we have major and essential problems with secondary problems. First of all we must be instilling the concept of practicing sport in schools. We have schools; if you can see it; where the sport’s class is once weekly and even maybe the coach may not send the students to go out and play; although these students have energy. The student needs to disperse this inner energy in a form of sport because if the student didn’t disperse the energy he has may lead to dispersed in others things. Regarding the clubs, for example; I used to be a member in one of the Qatari clubs I was with them for 8 years. In the club there are 30 players; only 3 of them are residents and they ask for 27 Qatari players. For sure it’s so difficult to have 27 Qatari players within the team, so you may in the team 11 players and they don’t come for the training or anything but they come on game’s times. For example daily they arrive to the Qatari’s clubs; a residents young’s or students but the Qatari’s law deterred them from being/ presence in the club and that thing let them to play in the streets or some of them may stop permanently from practicing sport. An evidence for my words; I knew some guys they were they had the ability to reach the Qatari national team because of their strong skills in the sport but because of these laws that stopped them; no *Mash-Allah* they’re over 160kg of weight. Eating and sleeping that’s. The exercising was prevented. So if there was a development in the laws and revised; may be the resident in Doha may be in the Qatari team. It may enhance the sport and would develop it for better, these are the most important. Regardless we must focus on the schools and children because we have the biggest overweight percentage ever in Qatar among children. This is a big problem it may lead them to have diabetes, diseases and hypertension are because of no movement. We must encourage them because it so hard to... The European’s community has the culture of taking care of self but in Arab no and vice versa once they go back home from school they find a dish of rice he start to eat and sleeps no this is true no this is real. So may find most of the students are *Mash-Allah* and the weight is… so I believe if we took care of these things I think we we’ll have a healthy environment and better.

M: The proof of that is the state is working on that. It organized champions on the highest level almost all the events.

M: Ok God bless them but still we need to focus on children because these children are going to be leaders. If those children haven’t the mentality and thoughtful power in the future they can’t lead or became leaders in the future. The state will relay on them in everything; in Qatar they depends on 20 -30 % in sports so if there was no development in the children who are going to be a leader. It’s a disaster

Interviewer 2: sorry but earlier you mentioned that in the school they have one sport class per week. Do you think this things is because unavailability of the buildings or if they make sport to be as a subject of success or drop-outs. I mean you won’t be able to upgrade you to higher level unless you get a certain score?

Male 5: well this might be a good suggestion for the sport class.

Male 5: no the sport class is scheduled to be once weekly between the all classes so the playground is available

Interviewer 2: So the buildings are available but the issue is the lack of attention to the sport itself

Male 5: yes exactly even the sport teacher comes holding the ball and then he thought t to them go and play and after 20 or 30 minutes he comes again asking to them go. Ok but some student doesn’t like the football, why do you force them to play this game. In the hottest weather you ask them for a walk. Many injuries happened am talking from the reality. Even the teachers have no experiences but you may find… for example if they increase the number of sport class to be 2 or 3 par of it to be theoretical and the other part is practical so the student would have the sport mentality. Teach them the games and so many other things. Honestly I finished the high school 12 years ago and through it; the coach gives them the ball and tell them to go and play [Male 1: Interruption: yes right]. Even some students keep waiting for the sport class so they can go and have fun somewhere else…

M: If you provide more facility especially in this weather

M: variety of facilities will help people to practice sport.

M: such as awareness programs for group age (1-20yrs) that is vertically, whereas horizontally in providing training for awareness,

M: We need also to provide a motivation for doing exercise for the young and not the adult. Instead of putting 50,000 prices, I would put 10,000 or 5,000 and in that case the young boy would want to score these points in all sports, such a simple price to stimulate youth, I knew one program in foreign country “Coach Carter”, he linked sport with marks the more students practice the more marks they get. So this is with the young students. For those who are adults: Doctors who are working and you are loading them with work, so provide them with a gym. Not only for doctors, go for the education and I am not saying that they put expensive devices, but at least you need to support your employees.

M: On society level, the awareness for those who don’t know and to provide facility to motivate society such as the sport day and to make it at regular base as well encourage active live. One sports day per year is not enough; it has to be always there and to encourage active life style in general.

M: media and in education

Male 4:On long run investment, it will impact on society for example, the treatment cost of diabetic or any other diseases, and so it is profitable by all measures. Do not belittle a club that will be suitable with the weather. If you did that, you will pay money on those who are diabetic, stroke or other chronic diseases.

M: It is also good if these designed places are close to your house make it easy for you to reach them.

M: Also should provide specific places for sports and are close to living areas. Like a park.

Male 3: Provide designed place to practice physical activity. For example when *Al-Rayyan* Park was destroyed we walk on the street and the street is not safe due to many cars, hot and sunny weather. Before I went there day and night, but now I walk on the street for half hour.

a- **Government support**

M1: the most important thing is providing the necessities of this subject such as playgrounds (sport courts and fields) and others; sports halls, and these things to be present. That’s it

Interviewer 1: so to provide halls

M1: yes there isn’t something else.

M2: same thing, the covered halls that will... that help even if the weather is summer; one could walk because it’s hard when you will be totally wet, so if there are more halls and are distributed

M2: yes that would help better

M3: same thing; air conditioned sports halls especially in summer, in winter there is no problem

M4: same thing

Interviewer 1: same thing

M2: for example mam there are schools… I mean part from things that could include old schools the government had leased them to new schools and all of these schools are air conditioned, so if these schools were changed into sports halls that would be a good thing, it will also provide a place….

M1: the most important thing is…there are schools, clubs..

M2: in these schools, there are playgrounds and this can be the area to use

M1: especially if there was in the place something that encourages you

M2: exactly yes.

M2: or use the schools during summer

M2: in the summer there is no studying and schools are closed, so they could be opened for people and make in them... let walking and running be practiced there specially in the area of ----

M2: or all the schools

M2: its only the schools suggestions

Interviewer 1: the schools? Okay

M1: the most important thing is to make swimming pools

Interviewer 1: swimming centers

M1: yes

[Crosstalk]

M1: because this process is present and the sea is a bit shallow ---- so things to encourage people

F1: I suggest for example each one has a sport hall in his house, or in compounds there be sports hall or in neighborhoods, each neighborhood there would be places they call them green areas for people to walk or play.

F4: I’m with providing center that is specialized in promoting exercise, a club for young females only for example, controlled by a special department and professional coach not any person to be there,

F: or let them , at least; providing rooms with AC or specific halls for this thing.

F: secondly to provide them the suitable environment and suitable time.

F: Thirdly there should be trainers in good standard with the students or young people to prepare them for doing sport.

F: Secondly I suggest putting a day for sport. There is sport day here in Qatar put other Arabic countries don’t have sport day or… if you look to the gulf countries specifically they care about the sports but they didn’t reach the desirable goal of the young people.

Male4: because a country like Qatar, it has a lot of sport activities but there is shortage in motivation and make the sport likable. The sport awareness in general

Male5: through competition like in the *Aldori and Alkas* (T.V channels).

Male1: there are universities here in Qatar set up a day for a championship for all the sports. For example there is volleyball and there are 20 teams in the universities and there is playoff between them. I heard about the strong man. They started with light weight with 15 participates and they start to elevate it. Then they increase the weights and elevate until they reach the last 2 or 3 participates and they playoff until they reach the maximum. They give awards and academic recommendations and you will have it together with the graduation certificate.

Male2: most of the young people don’t accept that the sport is … it is rarely when you find it very few. If you see the volunteering work in Qatar who did it start? It was rarely when you see the Qatari young people go for volunteering like volunteering in Qatar volunteering company or Qatar University they all go why? Because they know their own benefits from that and they know there are experiences and relations. He will improve himself. It should be the same for the sport, motivations and benefits at least to be financially or something let the young people care about it. After this stage, the young man spontaneously will say no need to take awards every time. When he adapts this, he will do sport spontaneously.

M4: in my opinion the best ~~the~~ thing that motivates most is the availability of sports clubs. In each club there should be someone who will give you the correct awareness. When you are doing exercise alone you can do something extra or decrease an activity, on the other hand, when you are in a club and there is a doctor who knows what is excess in your body or less, he would also know how many time per day for exercise you need so you won’t feel exhausted.

M: To increase it at schools, make PE classes more, increase the importance of doing exercise at school. For example, for me, at my school, if there was a program after school, an exercise program, that would be the beginning, from here. Let say leave the school hours, the school schedule can finish at 1:30-2:00pm, and for a certain days in the week, the student can stay from 2:00 to 4:00pm even till *Maghrib* (afternoon 5:00pm), there is no problem, there is transportation where he can go back home and at the same time he is under school care, he can be in the exercise programs, games. This is because the tournaments at schools in different sports is there so if we put something like that at schools, there will be challenge and this will grow and the student will have the talent and in the end this will appear as all the people would be doing exercise and the awareness will increase. In addition, the country will find talents in different sports; this is because it started from school. Physical activity is 2 to 4 hours per week. You can bring up a talented footballer who would be the world champion in football, for example.

M2: We can encourage them through games, when there are more games, when there are more games there would be more motivation. Like in *Katara*, there was a game that was done in *Katara* and there were lots of people there.

M: Besides, providing large playgrounds on a large scale in this country and not like any other country because *Masha Allah* this country has this, also at the same time, you might not find a place to exercise, as a game not like walking, you can find a place to walk. Providing the place is important.

M4: When the country comes and motivates, this mean that it can, when a country comes and encourage it means that it can give awareness. Awareness is an encouragement to exercise and it can motivate; and motivation can be by founding playgrounds; bring trainers and things like that. But awareness (alone) is not often practical; it is theoretical in portraying pictures and things like that. If the country’s budget that it will spend, if it is going to spend it in one area, my opinion is to, motivation is more important than awareness because some people know they want to be so, like students at college, each one drew his future, I, for example, will graduate and will take this, this and that. He has the awareness but where is the motivation.

M4: where is the motivation, the movement? You know where you want to reach and know that, for example, I want to be a world champion; I want to be, to take medals in this and this. Ok, you know, but where is the motive? Motivation could be through clubs,

M1: first of all “Healthy mind is in a healthy body”, second of all provide places for sports only across the country, and to tell people to practice sports for your own health.

M3: I think to increases trainers at gym, they should be available for the encouragement from the trainer to the youth not for financial issues.

F: there is not advertisement, so I need to search and ask for the place suitable for women. Sometimes it is not suitable in time or the coach is male and not female, this would be difficult. These are things that tie you not to practice.

Female2: i greed with her in the lack of couch. They provide gym but will find trainer by appointment , for example first time for me at gym i don’t know how to use machines and what to do exactly , and trainer will come and teach on such machines but you still don’t know what kind of machine you are working on. They should provide for each 3 individual a special trainer

I: How we could benefit from your suggestion as student studying in university level?

Female1: they can provide for us places at university, in truth we have gym but we don’t have a female trainer and she will not be available all the time for us , no time, also lack of spaces at gym which cannot fit all university students

I: The spaces of gym dose not accommodate students?

Female1: yes the spaces is small and not enough for all student to motive them. The machine also limited, to limited. I will not find all kind of machine i need

F3: they can make for us clubs, [interruption from f2: yes particular clubs for women)

F3: gym, as there is gym for men’s they can help women and do gym for them, gym should be on the expense of country, so instead person will go and pay money on gym country should provide that centers ,clubs for women[ interruption F2: yes for Women]

F: to open clubs, besides that they should provide garden for women only so they can run with their kids safely

F3: if you see open doors play grounds most of them are men especially youth, so woman get (Harassment of young people)

F3: to encourage people to do sports, open clubs for women’s,

F2: to do competition, marathons for women only,

F2: particular places that women can use it to jog

F3: to help and motivate

M2: I told you about which is the infrastructure when it is available near to people houses .this will play an important role.

M3: of course, it will encourage

M2: This plays a very big role in that, when the club provide places for example, *Qatar Sports Club* may cover the in *Dafna* area. If there are trainers who would have programs that help in giving education, it is not for young… and age group issues [*Interruption by patient asking for the doctor min 10:33]*

M2: I say this as an example.it plays an important role even if it is late, he can find an hour or half an hour, and things like that.

F: I have noticed that when I was on vacation on another Gulf country, I have seen the ladies wearing trainers and they were at mall. They turn around the mall with AC and cool. It is cheap and cost nothing and you can leave your children in a place to play in or to leave them with the housemaid and you turn around the mall. If you turn around the mall 10 times, this will be …..

Female1: I have noticed that they opened centers, big centers prepared with everything, if they make a small room for gym for the staff. This may help people and it will not cost a lot. A simple thing like treadmills, steps. Simple things and with the staff… after the work and before she start the work half an hour before it, she can do it with her friends, some exercises and start her shift this may help people to activate them at the work place and will not go to a distance places. These are simple things and I think it could be done.

F: The mall also, because the mall avoids the weather problem.

Female2: provide gardens, with walking tracks for only ladies. We can put our children to play and we can walk at the same time with the family.

Female3: in Qatar there are many centers for youth and cultural centers for ladies. All the cultural centers here depend on the seminars and meetings, etc...there is no part for gym; as the doctor said, for … as an extension to it. If all the cultural centers accompany with small gym room for free to the people or participate with few money, this will encourage them. It is not only for medical staff or the employee in the health center, no, also in youth centers. Here they care more in the centers for the men, having swimming pool etc.., but the same youth center in woman branch, the youth centers of men branch and woman branch are completely different. The woman branch is only for meeting room, lectures and etc.., but there is no gym, no swimming pool, nothing like those. On the other hand in the men branch there is football playground and tennis playground and playgrounds for many thing including swimming pool and all of these… if there is one for ladies, that will motivate them to go and it is for free, participate and can go every day.

F:If the weather is hot and we can't go in the garden, then the youth centers for us will be ok.

F: So they need to provide these things and what they provide from activities to the boys, they should make the same for the girls, so they will be involved in that. They put that inside them and this will make big difference since childhood.

Female3: to make our duty lesser (laugh)

Female3: if there is extension room for gym, as the doctor said, in our health center. Instead of eating, drinking etc. in our break time and that is it we can do sport and that will encourage us too. that will motivate all the staff.

F: but the governmental centers are mainly for men. For example the *Zakirah* youth centre, you will find in the men department swimming pool, playground, There all the playgrounds in the men branch. In the woman branch, it is a villa and normal room where they do meetings and seminars memorization *(mostly Qur’an)* it is all on seats, there are no activities. It is only once they go to men branch in a month. It is rare. If the branch itself opens for the girls all the time, there will be chances to…

Female1: there is a very nice sport, swimming, if they bring a lady to train the young girl for swimming, together with the mother, this will be make the girls busy with it, because the sport will make the young people busy from internet, mobiles, movies and games etc. the sport is very very important.

Female3: as I told you, for boys they have activities and they have football. You find only one place for girls and it is in Doha. What about the population of all these places, where should they go?

F1: The country can provide a club for the elderly

F2:that is right, they can provide a hall in the districts

F1: provide a club for the elderly so they get encouraged.

F2:They can provide a hall for women for example

F1: women and men.

F2:They can do that for the age 45 and above with trainers and with small registration fees, because not all people can pay expensive.

Interviewer1: On the country level?

F3: Also on the State level

Interviewer1: Like what?

F3: they can provide us with clubs and things like that; we go there and come back, chat and drink coffee and do this..[Laugh] without coffee and tea it will not work.

F4: I have the same idea, to provide places for exercise not necessarily clubs, there are open areas and public gardens but they should provide us with specialists, when you all gather, there will be someone to encourage you. He would give you the movements to do according to your age, and your health status and so give you the correct movement to your age, the body movement that would not make you tired, according to your age and they diseases you have, so they have to be specialists available to assist and support you. You can go back to them and ask them for the movement that suits you.

Male 1: on my point of view and for the football playing if they make more playground for public

M: There are many playgrounds but it is with registration and it will be crowded

Male4: honestly there are many places to play sports

Male 1: there are *Aspire and Katara*

Male 4: you can go to cornich for walking

Male 2: if Qatar has a lot of money, why do they take us a broad for sport and losing weight, to other countries? Take number of people, number of people every time, 30 persons, 40 persons. Take Qatari only. This is my suggestion

F1: to provide the appropriate atmosphere; like proper places for the gyms, here I think we have Aspire for females but we don’t have many places for females I think here in Doha

F3: or they could have activities that includes all the members of the family. If the mother likes to take her children and her husband would be there with them, of course they will be convinced and would like to join if there are such activates.

M1: I prefer sports; gym and such, closed halls, with AC, helps one to be able to move around that’s it.

M2: for me I say this will not be from the people, it should be from the Country, the country provides a suitable day for a sports day, but not one day in the year, it should be more than one day as a vacation. I have another suggestion

M2: I want here I want here in the country for Friday and Saturday to be days off

M3: yes right

M2: Friday day off and Saturday.

M2: you would provide suitable time for all people to do sports, what shall I do on Saturday? Stay at home? For sure I will go out to walk, I might not necessary go out do sports; I will go walk to my friends this is sports.

M3: the country should use its power; first thing to explain the aim of doing sports, force the private sector companies since I am working with a private sector I used to work even on Friday; force private sectors not to force workers work on Fridays, they might not refuse it but they leave the choice for them; and not say: if he didn’t work on Friday I will….

M3: yes, force private sectors to have periodical checkups for the employees; blood test, I can do it on my own I move around normally I don’t complain of anything, but when I find high blood cholesterol, the start of a problem, the start of fat on liver so I would fear for myself so I would go to sports, I know that doing sports would be the only way and to organize eating, so I put point which are regulatory for private sector that at least make Friday a day off. Obligatory, and after that it should be implemented you would go to the employee ask him he will say he is working Friday because he wants but it is not true, he is forced, not directly but he is indirectly forced to work on Fridays

M2: ---- and the time,

M3: yes, yes

M2: providing the time

M3: and at the same time we can apply, the work law can be applied in a way like; what is the official working hours? 7 hours? Make half an hour walking as now we have a break from 11:00 to 11:30

M: but the most important thing is providing the time.

M3: providing the time that’s it, whether by a weekly day off or taking a period of time from the official working hours for example half an hour out of the official 7 hours or 25 minutes or 15 minutes to practice sports even if in the same place it is not necessary

M2: move in the place

M3: yes yes for sports. One day a year is not enough

M2: to provide day off.

M3: it is off day, they have to give a day off for you to do sports, besides, when they give him off day he is not the only one who will do sports; it him and his family, for example my wife is staying home she wouldn’t go out except when I am there so now I will make her do sports and my kids do sports the whole family I mean, so the day off will not be for me alone; it will be for me and those who are around me. So this is important because there is no time so he wouldn’t do sports.

M1: I will say 2 suggestions first one; one day off a week not necessary to specify to make sports no, specify one day off, naturally this day off I will not spend it at home, I will have to go see my friend, I will walk a bit, So I did exercise without feeling that I did, this is one thing

M: the second thing; the clubs, if I am talking about exercise so the clubs, this is a suggestion to organize parties, sports games. That is it for my suggestions.

M2: for the clubs, this activity is present -------- that here the clubs specify the day that you go to do the training not the day you want to go to train, so for example you have Al Khour club,

M2: you have Al Khour club, if you went today and made a membership, they will specify for you to come on this and this days you can’t come on this and this days, why? For example the gym hall, swimming, football, all in appointments

M1: Swimming pool has certain hours.

M2: how do you make appointments obligatory and then you tell me come and practice sports? I like to do sports, I like to do it every day, I have half an hour free, I will come. Okay? But you are forcing me to come at certain times, so this is not membership

M2: you make me in the first place if I want to do sports you make me get lazy

M2: the sports are not given enough, sports generally; there are a lot of clubs in Qatar there are a lot of sport activities inside the clubs but unfortunately it is not used because of the routine that is set you come on this and that days don’t come… why? What is the reason?

M2: I am one of these people I went to apply in the club, I found him handing me a table saying this is the timing table

Interviewer1: timing table to work with,

M2: I told him no of course I am not free on this day, he said: it is not my problem you come to me later

F3: Wow! What a beautiful thing if they do that… [Group talking as they all agrees] if they can provide a break time so we can go for the GYM and you can move a little bit.

Interviewer2: so you suggest providing you with GYM hall full equipment.

F3: exactly; so the break would be for exercise. You got my point! You have a certain time just to move your body.

Interviewer2: so it’s not necessary to use GYM outside the work place?!

F3: yes exactly. At least 15 min her and 15 min at home for example to do something. It really became something nice, it will really help.

F1: the suggestions are to increase the number of health and sports clubs, and sometimes the fees for example to be reduced, sometimes could be encouraging fees not commercial, now most of the clubs; women wouldn’t go because the fees are commercial. Groups; for example each group in the neighborhood for example each group in a certain area there should be a sports club a special club for example for ladies, you will find all encouraged, otherwise all clubs you find them far away something in Doha, further. But in each area, if there is a sport club it will motivates that all people in this area to practice sports.

F1: and low fees.

F: Another thing as she had said increasing the number of sports centers in many places for example we are living in Al Wukair, inside is the center so in all al Wukair there is not a single sports club.

F1: there is

F2: where?

F1: the closest is in Barwa

F2: there is one in Ezdan I don’t know god knows if the word is correct or not. So imagine we have to go from al Wukair to Barwa

F2: if the place was close or would need 5 minutes to reach it this would help us

F3: the workplace

Interviewer 1: inside the workplace?

F3: yes, to do it during the break time,

F4: of course, you are talking about governmental encouragement? The country encourages sports and the centers are available everywhere thank god.

Interviewer 2: she means ---- are 2 types; on personal level and fits the same age group, and nation level for example there are female workers there are ideas and thoughts about them on the national level would be general

F4: yeah,

F4: national level of course to join the sports clubs by hours, take one or two hours of our time to practice sports; swimming or walking or using sports machines and weight lifting the ones that are light in order to strengthen your bones, on work level I suggest the same as this sister said,

F4: that what? The sister here said she wishes in the work place to be there a special place for sports so that one wouldn’t get bored, in the time of the break he could go move in that time, first thing he will refresh his activeness and Sobhan Allah sports give you new thoughts and new productivity and makes you fresh so this helps better in regards to productivity at work and not being lazy

M:The government provides like treadmill machines and you can do that once or twice per week. I used to be committed to it but then the time issue came up where I couldn’t use it any more. It was really useful for the belly part. Even for breathing, there were times I couldn’t even try to (participant was pointing to his socks)

M: provide suitable facilities and places to jog, preparing the suitable ground that all.

M2: first of all the Supreme Council of health they don’t have activity I mean places for exercise,

M1: to have extra time, I work at company, they have branch in Dubai, they have a branch where when staff have free time they practice exercise. So they do that in during their duty

M3: for long shift duty I prefer if they will provide places to practice during resting hours

F4 interrupting: there is a place just like Aspire but the main problem is there is no space. The club supposed to be spacious place. And a private place for ladies and kids. [F1 interrupting: the fees are high and some people cannot pay] the fess is low but nobody pays and there is no vacancy [F1: interrupting: and people have no time] [F1 interrupting: what if she has no car and her husband is busy?! Aspire; all the people like to go there it’s a known place even in Thursdays…]

F1: Aspire Park honestly is the best solution. Everybody satisfy with it. [Side talking and door opened] we don’t want to go to malls; you know what I mean, its better if one takes the advantage of this weather!

F3: Yes the weather.

F2: area for sports, beside the clubs. True there are centers for exercising but these are not provided by the government. It is not the same as the club where you can go at any time. Soany place to practice sport for example clubs for ladies same as clubs for men.

F4: Same thing in addition to bing close to those areas.

F3: the same, provide the alternatives, for example: people not doing sport because of long distance and time factor. Provide clubs in each area. Provide everything and should be for ladies. I think this will be encouraging.

F 4: the same, clubs.

F1: the places are far away.

F: It should be mandatory for the student. Some employee in their beak time. Encouraging procedures for the employee and to be mandatory for the students.

F3: for the employee, for example in certain time according to their work times, as I mentioned, near places and provide the suitable environment. For example if the weather is hot, it should be inside, this would be encouraging

F3: yes. The same thing This is the encouraging.

F2: when the place is near you can reach very fast but when it is far, no.

M4: I have suggestion, if any one from the doctors here is obese to quit from his work,

M4: yes, the doctors should be the first one, I see one who is obese and like this, he is a doctor.

M4: My first suggestion is they should make for us logo and start on it, so they will be the role model*, a quote in Arab society says ‘the door of carpenter is broken’*, this cannot be.

M2: that is good idea

M4: there should be a club in each in each village, the brother said there is a lot club, there are not so many clubs in the area, and each village must have a club to practices for free so the people of this village would go for free.

M3: there are gardens to walk.

Female 5: For us in the work they should put the one hour break to the exercise since most of them they don’t eat or they use only ten minutes from it because most of them on diet. I which If they can do like the other countries.

Female 1: outside the center there is a big space you can walk on it. Thereis available place for walking other than the centers. Even in Aspire the gardens are everywhere and between the neighborhoods there are gardensand walking trails. I don’t see any obstacles.

Female 2: Every neighborhood has garden and available place for walking. And even without the garden some streets have walking trails that written on it so the cars don’t come on it.

Female 4: to provide the exercise machine with reasonable prices.

Female 5: privacy for women .Also the public machine should have some privacy for the women. When you go to Aspire and its empty you can play there but in the presence ofsomeone it’s difficult. There is walking place in Al-Rayan but it has been closed; it was wonderful. Al-Rayan walking placewas more comfortable than Aspire.

Female 6: Al Gharafa is better.

Female 5: It was walking area when you walk.Everyone is walking so you get the encouragement. In Aspire you can find some people are sitting, children are playing; you scare to hit them. There wasan open space for playing.I used to prefer if there is close one for women; we had one but they closed it now. We want special place for women wearingHajabwhichit is difficult to take it off in front of the presence of male. I like quietness…The sports machine sometimes helps me.

Female 6: availability of the sports machine with reasonable prices. Make more places especially for women.

Female 1: Regarding the sports hereMash-Allahthey did their best. They did it well. They create the “Sport Day”.In the school the sport class became very important. The boys are very keen about it and the teacher as well...

Female 2: The obese kids like my daughter and my daughter-in- lowMash-Allahthey register them in Aspire. They sent us the paper telling us they want them to join. They started to follow and check-up for the diabetes. Honestly we thank them the schools for thatMash-Allah.

Female 3: Even the teacher told me that your son is fatand he has to join...but she did not register him. She told me he has to eat healthy food and since that timewe stopped eating eggs, nuggets or burger and we start to eat healthy food. She told me when he grow up he will become tired. He is in the first grade now when she saw him he used to be fat although the food at the school is healthy.

Female 4: I will complete what she said even in the school they don’t sell candies, or chips or Pepsi for children. And then they sell things with good advantage like cheese and also the fruits.My daughter told me that they sell fruits and vegetables in school.

Female 3: apples, cucumber, carrots all available in the school.

Female 4: all of these things are good they encourage the children themselvesfor healthy food may; God guide them.

Female 1: Suggestion is like; the clubs should be special for women and the working hours should be suitable for the employee.

Female 1: through clubs.

Female 1: Clubs or educate them through sending SMS via the phone and participatethrough places like this conferences and lectures which is encourage little bit.

Female 1: yes the distances and walking but the opposite the old women but we are thanks God even I would love the older so that would not effect on their aromatize. Even when I go for walking at Aspire I see even old women a very old, oldandwhen I see them I get encouraged. Some of them are grandma and they circle around wearing sport shoes they do not walk with normal shoes. At the opposite I get encouraged more and I walk more. I can say Aspire;thanks God for the walking space.

Female 3: If they increased the clubs only one club.

F: It is better to increase the numbers of clubs or designated place for sport. For example, put small club in each park to encourage people to practice especially here in Qatar I cannot see.

F: It is better to encourage this physical activity in the health center.

F: The company they work at it they should support it. Well when you see employee in a good health and enjoying the lightness and move that gives them more energy. If I am the owner of a company and I found an obese employee but his working is good and his brain is very high this is jewel I cannot lose him. He will not give the production but his brain is good. I want to utilize his brain in the job. What should I do? I will support him to exercise. Guys we are enrolled the company in a center with reasonable price so that the employee will pay it. This will encourage the whole employee to be in group and they will noble competition between them to make encouragement to make them to give energy in the morning and at the end of the day. They feel it’s a recreation not like a type of routine. No this journey I don’t want to go. If my friends are going with me and this one his weight is that and the other is thiswhy I am like this I have to be diligent to reach.

Female 1: I hope in any public place at least they should have park, gym, exercise machine.

F: To encourage someone who wants to do exercise any he doesn’t have machines at home or they can’t buy it. So it should be free or could have free membership to do exercise.

F: I feel the prices are different instead it should promote health and should be reasonable for people.

Female 3: The same thing, there should be gyms that everyone can join and should not be expensive so cannot join.

Female 4: In the university should have sport hall for the student to join it.

Female 5: It should be near the homes.

Female 6: increase the numbers and near homes and available in any area. There should be section for female different males. Not only fitness but also the numbers of park places, you find Aspire or Cornish is for away from your residence.

M: Wallahi to increase the number of playgrounds, makes it bigger because it is small

M: And try more to encourage the youth for sport like the National Day of Sport

M: Also they can make bigger athletic supplements

M: And also it should be a nice place to attract youth for GYM, you know there is GYM that is well designed

M: Also if they provide more athletic supplements also it will attract people to them.

M: They can make a special building or a special club, a very big building in Qatar just for sports games, there is no GYM just only games.

M: so by increasing these public facilities like a football playground either made of grass or a halls, the same thing some people love the big playground and there is the normal one so also increasing the number of the sport public facilities and the advertisement.

Male 1: *Wallahi*! What can I say; first of all here in this country let them try to change their rules in those clubs because it hinders us honestly…

Male 1: well these are. If you were not born in Qatar it’s so hard to play with them …. They prevents... I mean the majority of the people in this country are… [Male 6: interruption: I think in each team they are 1 or 2 or 3 …] [Male 5: interruption: no they are 3…]

Male 1: yes 3

Male 6: no but there is like in the relay races and in the individual’s games... It’s allowed to have 2 players in each category age groups in each team according to the sports club’s laws.

Male 1: so this is a problem. Almost all the Qatar’s populations are foreigners

Male 6: no no; I am a Vice Chairman of the sport club and am an employee here in Qatar University – specialty of sports; so I know all these rules.

Male 2: really we suffer because of these rules. We don’t know…even if I would like to go the club to play and to be encouraged; you will play with them 2 or 3 days then they say to you by the way we’re not going to registered you with us. It’s like he broke the motivation that you have he just broke it so you go home.

Male 6: and the people who set the rules regarding the age group is the same thing… the people who set the rules regarding the age group the put it as an obstacle, where I don’t see there is no certain age to play sport.

Male 6: I would say not to bring foreigners for example we have certain sport category where it can all be foreigners so this thing would lead not to love the sport… there will be one strong team and all the others teams are weak so the competition is know from the beginning not to bring foreigners and relaying on the local players.

Male 5: For me there are suggestions because we have major and essential problems with secondary problems. First of all we must be instilling the concept of practicing sport in schools. We have schools; if you can see it; where the sport’s class is once weekly and even maybe the coach may not send the students to go out and play; although these students have energy. The student needs to disperse this inner energy in a form of sport because if the student didn’t disperse the energy he has may lead to dispersed in others things. Regarding the clubs, for example; I used to be a member in one of the Qatari clubs I was with them for 8 years. In the club there are 30 players; only 3 of them are residents and they ask for 27 Qatari players. For sure it’s so difficult to have 27 Qatari players within the team, so you may in the team 11 players and they don’t come for the training or anything but they come on game’s times. For example daily they arrive to the Qatari’s clubs; a residents young’s or students but the Qatari’s law deterred them from being/ presence in the club and that thing let them to play in the streets or some of them may stop permanently from practicing sport. An evidence for my words; I knew some guys they were they had the ability to reach the Qatari national team because of their strong skills in the sport but because of these laws that stopped them; no *Mash-Allah* they’re over 160kg of weight. Eating and sleeping that’s. The exercising was prevented. So if there was a development in the laws and revised; may be the resident in Doha may be in the Qatari team. It may enhance the sport and would develop it for better, these are the most important. Regardless we must focus on the schools and children because we have the biggest overweight percentage ever in Qatar among children. This is a big problem it may lead them to have diabetes, diseases and hypertension are because of no movement. We must encourage them because it so hard to... The European’s community has the culture of taking care of self but in Arab no and vice versa once they go back home from school they find a dish of rice he start to eat and sleeps no this is true no this is real. So may find most of the students are *Mash-Allah* and the weight is… so I believe if we took care of these things I think we we’ll have a healthy environment and better.

M: The proof of that is the state is working on that. It organized champions on the highest level almost all the events.

Interviewer 2: sorry but earlier you mentioned that in the school they have one sport class per week. Do you think this things is because unavailability of the buildings or if they make sport to be as a subject of success or drop-outs. I mean you won’t be able to upgrade you to higher level unless you get a certain score?

Male 5: well this might be a good suggestion for the sport class.

Male 5: no the sport class is scheduled to be once weekly between the all classes so the playground is available

Interviewer 2: So the buildings are available but the issue is the lack of attention to the sport itself

Male 5: yes exactly even the sport teacher comes holding the ball and then he thought t to them go and play and after 20 or 30 minutes he comes again asking to them go. Ok but some student doesn’t like the football, why do you force them to play this game. In the hottest weather you ask them for a walk. Many injuries happened am talking from the reality. Even the teachers have no experiences but you may find… for example if they increase the number of sport class to be 2 or 3 par of it to be theoretical and the other part is practical so the student would have the sport mentality. Teach them the games and so many other things. Honestly I finished the high school 12 years ago and through it; the coach gives them the ball and tell them to go and play [Male 1: Interruption: yes right]. Even some students keep waiting for the sport class so they can go and have fun somewhere else…

M: If you provide more facility especially in this weather

M: variety of facilities will help people to practice sport.

M: We need also to provide a motivation for doing exercise for the young and not the adult. Instead of putting 50,000 prices, I would put 10,000 or 5,000 and in that case the young boy would want to score these points in all sports, such a simple price to stimulate youth, I knew one program in foreign country “Coach Carter”, he linked sport with marks the more students practice the more marks they get. So this is with the young students. For those who are adults: Doctors who are working and you are loading them with work, so provide them with a gym. Not only for doctors, go for the education and I am not saying that they put expensive devices, but at least you need to support your employees.

Male 4:On long run investment, it will impact on society for example, the treatment cost of diabetic or any other diseases, and so it is profitable by all measures. Do not belittle a club that will be suitable with the weather. If you did that, you will pay money on those who are diabetic, stroke or other chronic diseases.

M: It is also good if these designed places are close to your house make it easy for you to reach them.

M: Also should provide specific places for sports and are close to living areas. Like a park.

Male 3: Provide designed place to practice physical activity. For example when *Al-Rayyan* Park was destroyed we walk on the street and the street is not safe due to many cars, hot and sunny weather. Before I went there day and night, but now I walk on the street for half hour.

- **Physical Environment**

M1: the most important thing is providing the necessities of this subject such as playgrounds (sport courts and fields) and others; sports halls, and these things to be present. That’s it

M1: yes there isn’t something else.

M2: same thing, the covered halls that will... that help even if the weather is summer; one could walk because it’s hard when you will be totally wet, so if there are more halls and are distributed

M2: yes that would help better

M3: same thing; air conditioned sports halls especially in summer, in winter there is no problem

M4: same thing

M2: for example mam there are schools… I mean part from things that could include old schools the government had leased them to new schools and all of these schools are air conditioned, so if these schools were changed into sports halls that would be a good thing, it will also provide a place….

M1: the most important thing is…there are schools, clubs..

M2: in these schools, there are playgrounds and this can be the area to use

M1: especially if there was in the place something that encourages you

M2: exactly yes.

M2: or use the schools during summer

M2: in the summer there is no studying and schools are closed, so they could be opened for people and make in them... let walking and running be practiced there specially in the area of ----

M2: or all the schools

M2: its only the schools suggestions

M1: the most important thing is to make swimming pools because this process is present and the sea is a bit shallow ---- so things to encourage people

F1: I suggest for example each one has a sport hall in his house, or in compounds there be sports hall or in neighborhoods, each neighborhood there would be places they call them green areas for people to walk or play.

F4: I’m with providing center that is specialized in promoting exercise, a club for young females only for example, controlled by a special department and professional coach not any person to be there,

M: or let them , at least; providing rooms with AC or specific halls for this thing.

M: secondly to provide them the suitable environment and suitable time.

M: Thirdly there should be trainers in good standard with the students or young people to prepare them for doing sport.

M:Secondly I suggest putting a day for sport. There is sport day here in Qatar put other Arabic countries don’t have sport day or… if you look to the gulf countries specifically they care about the sports but they didn’t reach the desirable goal of the young people.

Male5: through competition like in the *Aldori and Alkas* (T.V channels).

Male1: there are universities here in Qatar set up a day for a championship for all the sports. For example there is volleyball and there are 20 teams in the universities and there is playoff between them. I heard about the strong man. They started with light weight with 15 participates and they start to elevate it. Then they increase the weights and elevate until they reach the last 2 or 3 participates and they playoff until they reach the maximum. They give awards and academic recommendations and you will have it together with the graduation certificate.

Male2: most of the young people don’t accept that the sport is … it is rarely when you find it very few. If you see the volunteering work in Qatar who did it start? It was rarely when you see the Qatari young people go for volunteering like volunteering in Qatar volunteering company or Qatar University they all go why? Because they know their own benefits from that and they know there are experiences and relations. He will improve himself. It should be the same for the sport, motivations and benefits at least to be financially or something let the young people care about it. After this stage, the young man spontaneously will say no need to take awards every time. When he adapts this, he will do sport spontaneously.

M4: in my opinion the best ~~the~~ thing that motivates most is the availability of sports clubs. In each club there should be someone who will give you the correct awareness. When you are doing exercise alone you can do something extra or decrease an activity, on the other hand, when you are in a club and there is a doctor who knows what is excess in your body or less, he would also know how many time per day for exercise you need so you won’t feel exhausted.

M:To increase it at schools, make PE classes more, increase the importance of doing exercise at school. For example, for me, at my school, if there was a program after school, an exercise program, that would be the beginning, from here. Let say leave the school hours, the school schedule can finish at 1:30-2:00pm, and for a certain days in the week, the student can stay from 2:00 to 4:00pm even till *Maghrib* (afternoon 5:00pm), there is no problem, there is transportation where he can go back home and at the same time he is under school care, he can be in the exercise programs, games. This is because the tournaments at schools in different sports is there so if we put something like that at schools, there will be challenge and this will grow and the student will have the talent and in the end this will appear as all the people would be doing exercise and the awareness will increase. In addition, the country will find talents in different sports; this is because it started from school. Physical activity is 2 to 4 hours per week. You can bring up a talented footballer who would be the world champion in football, for example.

M2: We can encourage them through games, when there are more games, when there are more games there would be more motivation. Like in *Katara*, there was a game that was done in *Katara* and there were lots of people there.

M: Besides, providing large playgrounds on a large scale in this country and not like any other country because *Masha Allah* this country has this, also at the same time, you might not find a place to exercise, as a game not like walking, you can find a place to walk. Providing the place is important.

M4: When the country comes and motivates, this mean that it can, when a country comes and encourage it means that it can give awareness. Awareness is an encouragement to exercise and it can motivate; and motivation can be by founding playgrounds; bring trainers and things like that. But awareness (alone) is not often practical; it is theoretical in portraying pictures and things like that. If the country’s budget that it will spend, if it is going to spend it in one area, my opinion is to, motivation is more important than awareness because some people know they want to be so, like students at college, each one drew his future, I, for example, will graduate and will take this, this and that. He has the awareness but where is the motivation.

M4: where is the motivation, the movement? You know where you want to reach and know that, for example, I want to be a world champion; I want to be, to take medals in this and this. Ok, you know, but where is the motive? Motivation could be through clubs,

M1: first of all “Healthy mind is in a healthy body”, second of all provide places for sports only across the country, and to tell people to practice sports for your own health.

M3: I think to increases trainers at gym, they should be available for the encouragement from the trainer to the youth not for financial issues.

M: there is not advertisement, so I need to search and ask for the place suitable for women. Sometimes it is not suitable in time or the coach is male and not female, this would be difficult. These are things that tie you not to practice.

Female2: i greed with her in the lack of couch. They provide gym but will find trainer by appointment , for example first time for me at gym i don’t know how to use machines and what to do exactly , and trainer will come and teach on such machines but you still don’t know what kind of machine you are working on. They should provide for each 3 individual a special trainer

Female1: they can provide for us places at university, in truth we have gym but we don’t have a female trainer and she will not be available all the time for us , no time, also lack of spaces at gym which cannot fit all university students

I: The spaces of gym dose not accommodate students?

Female1: yes the spaces is small and not enough for all student to motive them. The machine also limited, to limited. I will not find all kind of machine i need

F3: they can make for us clubs, [interruption from f2: yes particular clubs for women)

F3: gym, as there is gym for men’s they can help women and do gym for them, gym should be on the expense of country, so instead person will go and pay money on gym country should provide that centers ,clubs for women[ interruption F2: yes for Women]

to open clubs, besides that they should provide garden for women only so they can run with their kids safely

F3: if you see open doors play grounds most of them are men especially youth, so woman get (Harassment of young people)

F3: to encourage people to do sports, open clubs for women’s,

F2: to do competition, marathons for women only,

F2: particular places that women can use it to jog

F3: to help and motivate

M2: I told you about which is the infrastructure when it is available near to people houses .this will play an important role.

M3: of course, it will encourage

M2: This plays a very big role in that, when the club provide places for example, *Qatar Sports Club* may cover the in *Dafna* area. If there are trainers who would have programs that help in giving education, it is not for young… and age group issues [*Interruption by patient asking for the doctor min 10:33]*

M2: I say this as an example.it plays an important role even if it is late, he can find an hour or half an hour, and things like that.

F: I have noticed that when I was on vacation on another Gulf country, I have seen the ladies wearing trainers and they were at mall. They turn around the mall with AC and cool. It is cheap and cost nothing and you can leave your children in a place to play in or to leave them with the housemaid and you turn around the mall. If you turn around the mall 10 times, this will be …..

F:The mall also, because the mall avoids the weather problem.

Female2: provide gardens, with walking tracks for only ladies. We can put our children to play and we can walk at the same time with the family.

Female3: in Qatar there are many centers for youth and cultural centers for ladies. All the cultural centers here depend on the seminars and meetings, etc...there is no part for gym; as the doctor said, for … as an extension to it. If all the cultural centers accompany with small gym room for free to the people or participate with few money, this will encourage them. It is not only for medical staff or the employee in the health center, no, also in youth centers. Here they care more in the centers for the men, having swimming pool etc.., but the same youth center in woman branch, the youth centers of men branch and woman branch are completely different. The woman branch is only for meeting room, lectures and etc.., but there is no gym, no swimming pool, nothing like those. On the other hand in the men branch there is football playground and tennis playground and playgrounds for many thing including swimming pool and all of these… if there is one for ladies, that will motivate them to go and it is for free, participate and can go every day.

F: If the weather is hot and we can't go in the garden, then the youth centers for us will be ok.

F: So they need to provide these things and what they provide from activities to the boys, they should make the same for the girls, so they will be involved in that. They put that inside them and this will make big difference since childhood.

F:but the governmental centers are mainly for men. For example the *Zakirah* youth centre, you will find in the men department swimming pool, playground, There all the playgrounds in the men branch. In the woman branch, it is a villa and normal room where they do meetings and seminars memorization *(mostly Qur’an)* it is all on seats, there are no activities. It is only once they go to men branch in a month. It is rare. If the branch itself opens for the girls all the time, there will be chances to…

Female1: there is a very nice sport, swimming, if they bring a lady to train the young girl for swimming, together with the mother, this will be make the girls busy with it, because the sport will make the young people busy from internet, mobiles, movies and games etc. the sport is very very important.

Female3: as I told you, for boys they have activities and they have football. You find only one place for girls and it is in Doha. What about the population of all these places, where should they go?

F1: The country can provide a club for the elderly

F2:that is right, they can provide a hall in the districts

F1: provide a club for the elderly so they get encouraged.

F2:They can provide a hall for women for example

F1: women and men.

F2:They can do that for the age 45 and above with trainers and with small registration fees, because not all people can pay expensive.

Interviewer1: On the country level?

F3: Also on the State level

Interviewer1: Like what?

F3: they can provide us with clubs and things like that; we go there and come back, chat and drink coffee and do this..[Laugh] without coffee and tea it will not work.

F4: I have the same idea, to provide places for exercise not necessarily clubs, there are open areas and public gardens but they should provide us with specialists, when you all gather, there will be someone to encourage you. He would give you the movements to do according to your age, and your health status and so give you the correct movement to your age, the body movement that would not make you tired, according to your age and they diseases you have, so they have to be specialists available to assist and support you. You can go back to them and ask them for the movement that suits you.

Male 1: on my point of view and for the football playing if they make more playground for public

M: There are many playgrounds but it is with registration and it will be crowded

Male4: honestly there are many places to play sports

Male 1: there are *Aspire and Katara*

Male 4: you can go to cornich for walking

F1: to provide the appropriate atmosphere; like proper places for the gyms, here I think we have Aspire for females but we don’t have many places for females I think here in Doha

M1: I prefer sports; gym and such, closed halls, with AC, helps one to be able to move around that’s it.

M: the second thing; the clubs, if I am talking about exercise so the clubs, this is a suggestion to organize parties, sports games. That is it for my suggestions.

M2: for the clubs, this activity is present -------- that here the clubs specify the day that you go to do the training not the day you want to go to train, so for example you have Al Khour club,

M2: you have Al Khour club, if you went today and made a membership, they will specify for you to come on this and this days you can’t come on this and this days, why? For example the gym hall, swimming, football, all in appointments

M1: Swimming pool has certain hours.

M2: how do you make appointments obligatory and then you tell me come and practice sports? I like to do sports, I like to do it every day, I have half an hour free, I will come. Okay? But you are forcing me to come at certain times, so this is not membership

M2: you make me in the first place if I want to do sports you make me get lazy

M2: the sports are not given enough, sports generally; there are a lot of clubs in Qatar there are a lot of sport activities inside the clubs but unfortunately it is not used because of the routine that is set you come on this and that days don’t come… why? What is the reason?

M2: I am one of these people I went to apply in the club, I found him handing me a table saying this is the timing table. I told him no of course I am not free on this day, he said: it is not my problem you come to me later

F1: the suggestions are to increase the number of health and sports clubs, and sometimes the fees for example to be reduced, sometimes could be encouraging fees not commercial, now most of the clubs; women wouldn’t go because the fees are commercial. Groups; for example each group in the neighborhood for example each group in a certain area there should be a sports club a special club for example for ladies, you will find all encouraged, otherwise all clubs you find them far away something in Doha, further. But in each area, if there is a sport club it will motivates that all people in this area to practice sports.

F1: and low fees.

F:Another thing as she had said increasing the number of sports centers in many places for example we are living in Al Wukair, inside is the center so in all al Wukair there is not a single sports club.

F1: there is

F2: where?

F1: the closest is in Barwa

F2: there is one in Ezdan I don’t know god knows if the word is correct or not. So imagine we have to go from al Wukair to Barwa

F2: if the place was close or would need 5 minutes to reach it this would help us

M:The government provides like treadmill machines and you can do that once or twice per week. I used to be committed to it but then the time issue came up where I couldn’t use it any more. It was really useful for the belly part. Even for breathing, there were times I couldn’t even try to (participant was pointing to his socks)

M:provide suitable facilities and places to jog, preparing the suitable ground that all.

M2: first of all the Supreme Council of health they don’t have activity I mean places for exercise,

F4: there is a place just like Aspire but the main problem is there is no space. The club supposed to be spacious place. And a private place for ladies and kids. [F1 interrupting: the fees are high and some people cannot pay] the fess is low but nobody pays and there is no vacancy [F1: interrupting: and people have no time] [F1 interrupting: what if she has no car and her husband is busy?! Aspire; all the people like to go there it’s a known place even in Thursdays…]

F1: Aspire Park honestly is the best solution. Everybody satisfy with it. [Side talking and door opened] we don’t want to go to malls; you know what I mean, its better if one takes the advantage of this weather!

F3: Yes the weather.

F2: area for sports, beside the clubs. True there are centers for exercising but these are not provided by the government. It is not the same as the club where you can go at any time. Soany place to practice sport for example clubs for ladies same as clubs for men.

F4: Same thing in addition to bing close to those areas.

F3: the same, provide the alternatives, for example: people not doing sport because of long distance and time factor. Provide clubs in each area. Provide everything and should be for ladies. I think this will be encouraging.

F 4: the same, clubs.

F1: the places are far away.

It should be mandatory for the student. Some employee in their beak time. Encouraging procedures for the employee and to be mandatory for the students.

F3: for the employee, for example in certain time according to their work times, as I mentioned, near places and provide the suitable environment. For example if the weather is hot, it should be inside, this would be encouraging

F3: yes. The same thing This is the encouraging.

F2: when the place is near you can reach very fast but when it is far, no.

M4: there should be a club in each in each village, the brother said there is a lot club, there are not so many clubs in the area, and each village must have a club to practices for free so the people of this village would go for free.

M3: there are gardens to walk.

Female 1: outside the center there is a big space you can walk on it. Thereis available place for walking other than the centers. Even in Aspire the gardens are everywhere and between the neighborhoods there are gardensand walking trails. I don’t see any obstacles.

Female 2: Every neighborhood has garden and available place for walking. And even without the garden some streets have walking trails that written on it so the cars don’t come on it.

Female 4: to provide the exercise machine with reasonable prices.

Female 5: privacy for women .Also the public machine should have some privacy for the women. When you go to Aspire and its empty you can play there but in the presence ofsomeone it’s difficult. There is walking place in Al-Rayan but it has been closed; it was wonderful. Al-Rayan walking placewas more comfortable than Aspire.

Female 6: Al Gharafa is better.

Female 5: It was walking area when you walk.Everyone is walking so you get the encouragement. In Aspire you can find some people are sitting, children are playing; you scare to hit them. There wasan open space for playing.I used to prefer if there is close one for women; we had one but they closed it now. We want special place for women wearingHajabwhichit is difficult to take it off in front of the presence of male. I like quietness…The sports machine sometimes helps me.

Female 6: availability of the sports machine with reasonable prices. Make more places especially for women.

Female 1: Suggestion is like; the clubs should be special for women and the working hours should be suitable for the employee.

Female 1: through clubs.

Female 1: Clubs or educate them through sending SMS via the phone and participatethrough places like this conferences and lectures which is encourage little bit.

Female 1: yes the distances and walking but the opposite the old women but we are thanks God even I would love the older so that would not effect on their aromatize. Even when I go for walking at Aspire I see even old women a very old, oldandwhen I see them I get encouraged. Some of them are grandma and they circle around wearing sport shoes they do not walk with normal shoes. At the opposite I get encouraged more and I walk more. I can say Aspire;thanks God for the walking space.

Female 3: If they increased the clubs only one club.

F: It is better to increase the numbers of clubs or designated place for sport. For example, put small club in each park to encourage people to practice especially here in Qatar I cannot see.

Female 1: I hope in any public place at least they should have park, gym, exercise machine.

F: To encourage someone who wants to do exercise any he doesn’t have machines at home or they can’t buy it. So it should be free or could have free membership to do exercise.

F: I feel the prices are different instead it should promote health and should be reasonable for people.

Female 3: The same thing, there should be gyms that everyone can join and should not be expensive so cannot join.

Female 4: In the university should have sport hall for the student to join it.

Female 5: It should be near the homes.

Female 6: increase the numbers and near homes and available in any area. There should be section for female different males. Not only fitness but also the numbers of park places, you find Aspire or Cornish is for away from your residence.

M: Wallahi to increase the number of playgrounds, makes it bigger because it is small

M; Also they can make bigger athletic supplements

M: And also it should be a nice place to attract youth for GYM, you know there is GYM that is well designed

M: Also if they provide more athletic supplements also it will attract people to them.

M: They can make a special building or a special club, a very big building in Qatar just for sports games, there is no GYM just only games.

M: so by increasing these public facilities like a football playground either made of grass or a halls, the same thing some people love the big playground and there is the normal one so also increasing the number of the sport public facilities and the advertisement.

Male 1: *Wallahi*! What can I say; first of all here in this country let them try to change their rules in those clubs because it hinders us honestly…

M: If you provide more facility especially in this weather

M: variety of facilities will help people to practice sport.

Male 4:On long run investment, it will impact on society for example, the treatment cost of diabetic or any other diseases, and so it is profitable by all measures. Do not belittle a club that will be suitable with the weather. If you did that, you will pay money on those who are diabetic, stroke or other chronic diseases.

M: It is also good if these designed places are close to your house make it easy for you to reach them.

M: Also should provide specific places for sports and are close to living areas. Like a park.

Male 3: Provide designed place to practice physical activity. For example when *Al-Rayyan* Park was destroyed we walk on the street and the street is not safe due to many cars, hot and sunny weather. Before I went there day and night, but now I walk on the street for half hour.

- **Physical Environment- Increase number of places**

M1: the most important thing is providing the necessities of this subject such as playgrounds (sport courts and fields) and others; sports halls, and these things to be present. That’s it

M: secondly to provide them the suitable environment and suitable time.

M4: in my opinion the best ~~the~~ thing that motivates most is the availability of sports clubs. In each club there should be someone who will give you the correct awareness. When you are doing exercise alone you can do something extra or decrease an activity, on the other hand, when you are in a club and there is a doctor who knows what is excess in your body or less, he would also know how many time per day for exercise you need so you won’t feel exhausted.

M: Besides, providing large playgrounds on a large scale in this country and not like any other country because *Masha Allah* this country has this, also at the same time, you might not find a place to exercise, as a game not like walking, you can find a place to walk. Providing the place is important.

M4: When the country comes and motivates, this mean that it can, when a country comes and encourage it means that it can give awareness. Awareness is an encouragement to exercise and it can motivate; and motivation can be by founding playgrounds; bring trainers and things like that. But awareness (alone) is not often practical; it is theoretical in portraying pictures and things like that. If the country’s budget that it will spend, if it is going to spend it in one area, my opinion is to, motivation is more important than awareness because some people know they want to be so, like students at college, each one drew his future, I, for example, will graduate and will take this, this and that. He has the awareness but where is the motivation.

M4: where is the motivation, the movement? You know where you want to reach and know that, for example, I want to be a world champion; I want to be, to take medals in this and this. Ok, you know, but where is the motive? Motivation could be through clubs,

F3: they can make for us clubs, [interruption from f2: yes particular clubs for women)

F3: gym, as there is gym for men’s they can help women and do gym for them, gym should be on the expense of country, so instead person will go and pay money on gym country should provide that centers ,clubs for women[ interruption F2: yes for Women]

F2: to do competition, marathons for women only,

M2: I told you about which is the infrastructure when it is available near to people houses .this will play an important role.

M3: of course, it will encourage

M2: This plays a very big role in that, when the club provide places for example, *Qatar Sports Club* may cover the in *Dafna* area. If there are trainers who would have programs that help in giving education, it is not for young… and age group issues [*Interruption by patient asking for the doctor min 10:33]*

M2: I say this as an example.it plays an important role even if it is late, he can find an hour or half an hour, and things like that.

F1: The country can provide a club for the elderly

F2:that is right, they can provide a hall in the districts

F1: provide a club for the elderly so they get encouraged.

Interviewer1: On the country level?

F3: Also on the State level

Interviewer1: Like what?

F3: they can provide us with clubs and things like that; we go there and come back, chat and drink coffee and do this..[Laugh] without coffee and tea it will not work.

Male 1: on my point of view and for the football playing if they make more playground for public

M: There are many playgrounds but it is with registration and it will be crowded

M2: for the clubs, this activity is present -------- that here the clubs specify the day that you go to do the training not the day you want to go to train, so for example you have Al Khour club,

[Crosstalk]

M2: you have Al Khour club, if you went today and made a membership, they will specify for you to come on this and this days you can’t come on this and this days, why? For example the gym hall, swimming, football, all in appointments

M1: Swimming pool has certain hours.

M2: how do you make appointments obligatory and then you tell me come and practice sports? I like to do sports, I like to do it every day, I have half an hour free, I will come. Okay? But you are forcing me to come at certain times, so this is not membership

M2: the sports are not given enough, sports generally; there are a lot of clubs in Qatar there are a lot of sport activities inside the clubs but unfortunately it is not used because of the routine that is set you come on this and that days don’t come… why? What is the reason?

M2: I am one of these people I went to apply in the club, I found him handing me a table saying this is the timing table. I told him no of course I am not free on this day, he said: it is not my problem you come to me later

F1: the suggestions are to increase the number of health and sports clubs, and sometimes the fees for example to be reduced, sometimes could be encouraging fees not commercial, now most of the clubs; women wouldn’t go because the fees are commercial. Groups; for example each group in the neighborhood for example each group in a certain area there should be a sports club a special club for example for ladies, you will find all encouraged, otherwise all clubs you find them far away something in Doha, further. But in each area, if there is a sport club it will motivates that all people in this area to practice sports.

Another thing as she had said increasing the number of sports centers in many places for example we are living in Al Wukair, inside is the center so in all al Wukair there is not a single sports club.

F1: there is

F2: where?

F1: the closest is in Barwa

F2: there is one in Ezdan I don’t know god knows if the word is correct or not. So imagine we have to go from al Wukair to Barwa

F2: if the place was close or would need 5 minutes to reach it this would help us

M:The government provides like treadmill machines and you can do that once or twice per week. I used to be committed to it but then the time issue came up where I couldn’t use it any more. It was really useful for the belly part. Even for breathing, there were times I couldn’t even try to (participant was pointing to his socks)

F4 interrupting: there is a place just like Aspire but the main problem is there is no space. The club supposed to be spacious place. And a private place for ladies and kids. [F1 interrupting: the fees are high and some people cannot pay] the fess is low but nobody pays and there is no vacancy [F1: interrupting: and people have no time] [F1 interrupting: what if she has no car and her husband is busy?! Aspire; all the people like to go there it’s a known place even in Thursdays…]

M4: there should be a club in each in each village, the brother said there is a lot club, there are not so many clubs in the area, and each village must have a club to practices for free so the people of this village would go for free.

M3: there are gardens to walk.

Female 1: outside the center there is a big space you can walk on it. Thereis available place for walking other than the centers. Even in Aspire the gardens are everywhere and between the neighborhoods there are gardensand walking trails. I don’t see any obstacles.

Female 2: Every neighborhood has garden and available place for walking. And even without the garden some streets have walking trails that written on it so the cars don’t come on it.

Female 4: to provide the exercise machine with reasonable prices.

Female 1: through clubs.

Female 1: Clubs or educate them through sending SMS via the phone and participatethrough places like this conferences and lectures which is encourage little bit.

Female 1: yes the distances and walking but the opposite the old women but we are thanks God even I would love the older so that would not effect on their aromatize. Even when I go for walking at Aspire I see even old women a very old, oldandwhen I see them I get encouraged. Some of them are grandma and they circle around wearing sport shoes they do not walk with normal shoes. At the opposite I get encouraged more and I walk more. I can say Aspire;thanks God for the walking space.

Female 3: If they increased the clubs only one club.

F: It is better to increase the numbers of clubs or designated place for sport. For example, put small club in each park to encourage people to practice especially here in Qatar I cannot see.

Female 1: I hope in any public place at least they should have park, gym, exercise machine.

Female 3: The same thing, there should be gyms that everyone can join and should not be expensive so cannot join.

Female 4: In the university should have sport hall for the student to join it.

Female 5: It should be near the homes.

Female 6: increase the numbers and near homes and available in any area. There should be section for female different males. Not only fitness but also the numbers of park places, you find Aspire or Cornish is for away from your residence.

M: Wallahi to increase the number of playgrounds, makes it bigger because it is small

M: Also they can make bigger athletic supplements

M: so by increasing these public facilities like a football playground either made of grass or a halls, the same thing some people love the big playground and there is the normal one so also increasing the number of the sport public facilities and the advertisement.

Male 1: *Wallahi*! What can I say; first of all here in this country let them try to change their rules in those clubs because it hinders us honestly…

M: If you provide more facility especially in this weather

M: variety of facilities will help people to practice sport.

Male 4:On long run investment, it will impact on society for example, the treatment cost of diabetic or any other diseases, and so it is profitable by all measures. Do not belittle a club that will be suitable with the weather. If you did that, you will pay money on those who are diabetic, stroke or other chronic diseases.

M: It is also good if these designed places are close to your house make it easy for you to reach them.

M: Also should provide specific places for sports and are close to living areas. Like a park.

Male 3: Provide designed place to practice physical activity. For example when *Al-Rayyan* Park was destroyed we walk on the street and the street is not safe due to many cars, hot and sunny weather. Before I went there day and night, but now I walk on the street for half hour.

- **Physical Environment- Coach available to exercise**

M3: I think to increases trainers at gym, they should be available for the encouragement from the trainer to the youth not for financial issues.

F: there is not advertisement, so I need to search and ask for the place suitable for women. Sometimes it is not suitable in time or the coach is male and not female, this would be difficult. These are things that tie you not to practice.

Female2: i greed with her in the lack of couch. They provide gym but will find trainer by appointment , for example first time for me at gym i don’t know how to use machines and what to do exactly , and trainer will come and teach on such machines but you still don’t know what kind of machine you are working on. They should provide for each 3 individual a special trainer

Female1: they can provide for us places at university, in truth we have gym but we don’t have a female trainer and she will not be available all the time for us , no time, also lack of spaces at gym which cannot fit all university students

M2: I told you about which is the infrastructure when it is available near to people houses .this will play an important role.

M3: of course, it will encourage

M2: This plays a very big role in that, when the club provide places for example, *Qatar Sports Club* may cover the in *Dafna* area. If there are trainers who would have programs that help in giving education, it is not for young… and age group issues [*Interruption by patient asking for the doctor min 10:33]*

M2: I say this as an example.it plays an important role even if it is late, he can find an hour or half an hour, and things like that.

Female1: there is a very nice sport, swimming, if they bring a lady to train the young girl for swimming, together with the mother, this will be make the girls busy with it, because the sport will make the young people busy from internet, mobiles, movies and games etc. the sport is very very important.

F2:They can provide a hall for women for example

F1: women and men.

F2:They can do that for the age 45 and above with trainers and with small registration fees, because not all people can pay expensive.

F4: I have the same idea, to provide places for exercise not necessarily clubs, there are open areas and public gardens but they should provide us with specialists,

F: He would give you the movements to do according to your age, and your health status and so give you the correct movement to your age, the body movement that would not make you tired, according to your age and they diseases you have, so they have to be specialists available to assist and support you. You can go back to them and ask them for the movement that suits you.

- **Physical Environment- Subsidized registration fees**

F: as an extension to it. If all the cultural centers accompany with small gym room for free to the people or participate with few money, this will encourage them

F2:They can provide a hall for women for example

F1: women and men.

F2:They can do that for the age 45 and above with trainers and with small registration fees, because not all people can pay expensive.

F1: the suggestions are to increase the number of health and sports clubs, and sometimes the fees for example to be reduced, sometimes could be encouraging fees not commercial, now most of the clubs; women wouldn’t go because the fees are commercial. Groups; for example each group in the neighborhood for example each group in a certain area there should be a sports club a special club for example for ladies, you will find all encouraged, otherwise all clubs you find them far away something in Doha, further. But in each area, if there is a sport club it will motivates that all people in this area to practice sports.

F1: and low fees.

Female 4: to provide the exercise machine with reasonable prices.

Female 6: availability of the sports machine with reasonable prices

M: I feel the prices are different instead it should promote health and should be reasonable for people.

M: and should not be expensive so cannot join.

- **Educational Institutions and organizations**

M2: for example mam there are schools… I mean part from things that could include old schools the government had leased them to new schools and all of these schools are air conditioned, so if these schools were changed into sports halls that would be a good thing, it will also provide a place….

M1: the most important thing is…there are schools, clubs..

M2: in these schools, there are playgrounds and this can be the area to use

M1: especially if there was in the place something that encourages you

M2: exactly yes.

M2: or use the schools during summer.

M2: in the summer there is no studying and schools are closed, so they could be opened for people and make in them... let walking and running be practiced there specially in the area of ----

M2: or all the schools

M2: its only the schools suggestions

M1: the most important thing is to make swimming pools

M1: because this process is present and the sea is a bit shallow ---- so things to encourage people

Female1: they can provide for us places at university, in truth we have gym but we don’t have a female trainer and she will not be available all the time for us , no time, also lack of spaces at gym which cannot fit all university students

Female1: I have noticed that they opened centers, big centers prepared with everything, if they make a small room for gym for the staff. This may help people and it will not cost a lot. A simple thing like treadmills, steps. Simple things and with the staff… after the work and before she start the work half an hour before it, she can do it with her friends, some exercises and start her shift this may help people to activate them at the work place and will not go to a distance places. These are simple things and I think it could be done.

F: as an extension to it. If all the cultural centers accompany with small gym room for free to the people or participate with few money, this will encourage them

Female3: to make our duty lesser (laugh)

Nagla: you mean the work load?

Female3: sure.

Female3: if there is extension room for gym, as the doctor said, in our health center. Instead of eating, drinking etc. in our break time and that is it we can do sport and that will encourage us too. that will motivate all the staff.

M2: I want here I want here in the country for Friday and Saturday to be days off

M3: yes right

M2: Friday day off and Saturday.

M2: you would provide suitable time for all people to do sports, what shall I do on Saturday? Stay at home? For sure I will go out to walk, I might not necessary go out do sports; I will go walk to my friends this is sports.

M3: the country should use its power; first thing to explain the aim of doing sports, force the private sector companies since I am working with a private sector I used to work even on Friday; force private sectors not to force workers work on Fridays, they might not refuse it but they leave the choice for them; and not say: if he didn’t work on Friday I will….

M3: yes, force private sectors to have periodical checkups for the employees; blood test, I can do it on my own I move around normally I don’t complain of anything, but when I find high blood cholesterol, the start of a problem, the start of fat on liver so I would fear for myself so I would go to sports, I know that doing sports would be the only way and to organize eating, so I put point which are regulatory for private sector that at least make Friday a day off. Obligatory, and after that it should be implemented you would go to the employee ask him he will say he is working Friday because he wants but it is not true, he is forced, not directly but he is indirectly forced to work on Fridays

M2: ---- and the time,

M3: yes, yes

M2: providing the time

M3: and at the same time we can apply, the work law can be applied in a way like; what is the official working hours? 7 hours? Make half an hour walking as now we have a break from 11:00 to 11:30

M: but the most important thing is providing the time.

M3: providing the time that’s it, whether by a weekly day off or taking a period of time from the official working hours for example half an hour out of the official 7 hours or 25 minutes or 15 minutes to practice sports even if in the same place it is not necessary

F3: Wow! What a beautiful thing if they do that… [Group talking as they all agrees] if they can provide a break time so we can go for the GYM and you can move a little bit.

Interviewer2: so you suggest providing you with GYM hall full equipment.

F3: exactly; so the break would be for exercise. You got my point! You have a certain time just to move your body.

Interviewer2: so it’s not necessary to use GYM outside the work place?!

F3: yes exactly. At least 15 min her and 15 min at home for example to do something. It really became something nice, it will really help.

F3: the workplace

Interviewer 1: inside the workplace?

F3: yes, to do it during the break time,

F: on work level I suggest the same as this sister said,

F4: that what? The sister here said she wishes in the work place to be there a special place for sports so that one wouldn’t get bored, in the time of the break he could go move in that time, first thing he will refresh his activeness and Sobhan Allah sports give you new thoughts and new productivity and makes you fresh so this helps better in regards to productivity at work and not being lazy

M1: to have extra time, I work at company, they have branch in Dubai, they have a branch where when staff have free time they practice exercise. So they do that in during their duty

M3: for long shift duty I prefer if they will provide places to practice during resting hours

F: It should be mandatory for the student. Some employee in their beak time. Encouraging procedures for the employee and to be mandatory for the students.

F3: for the employee, for example in certain time according to their work times, as I mentioned, near places and provide the suitable environment. For example if the weather is hot, it should be inside, this would be encouraging

F3: yes. The same thing This is the encouraging.

F2: when the place is near you can reach very fast but when it is far, no.

Female 5: For us in the work they should put the one hour break to the exercise since most of them they don’t eat or they use only ten minutes from it because most of them on diet. I which If they can do like the other countries.

Female 1: Regarding the sports hereMash-Allahthey did their best. They did it well. They create the “Sport Day”.In the school the sport class became very important. The boys are very keen about it and the teacher as well...

Female 2: The obese kids like my daughter and my daughter-in- lowMash-Allahthey register them in Aspire. They sent us the paper telling us they want them to join. They started to follow and check-up for the diabetes. Honestly we thank them the schools for thatMash-Allah.

Female 3: Even the teacher told me that your son is fatand he has to join...but she did not register him. She told me he has to eat healthy food and since that timewe stopped eating eggs, nuggets or burger and we start to eat healthy food. She told me when he grow up he will become tired. He is in the first grade now when she saw him he used to be fat although the food at the school is healthy.

Female 4: I will complete what she said even in the school they don’t sell candies, or chips or Pepsi for children. And then they sell things with good advantage like cheese and also the fruits.My daughter told me that they sell fruits and vegetables in school.

Female 3: apples, cucumber, carrots all available in the school.

Female 4: all of these things are good they encourage the children themselves for healthy food may; God guide them.

Female 1: Suggestion is like; the clubs should be special for women and the working hours should be suitable for the employee.

Female 4: In the university should have sport hall for the student to join it.

M: Regardless we must focus on the schools and children because we have the biggest overweight percentage ever in Qatar among children.

Interviewer 2: sorry but earlier you mentioned that in the school they have one sport class per week. Do you think this things is because unavailability of the buildings or if they make sport to be as a subject of success or drop-outs. I mean you won’t be able to upgrade you to higher level unless you get a certain score?

Male 5: well this might be a good suggestion for the sport class.

Male 5: no the sport class is scheduled to be once weekly between the all classes so the playground is available

Interviewer 2: So the buildings are available but the issue is the lack of attention to the sport itself

Male 5: yes exactly even the sport teacher comes holding the ball and then he thought t to them go and play and after 20 or 30 minutes he comes again asking to them go. Ok but some student doesn’t like the football, why do you force them to play this game. In the hottest weather you ask them for a walk. Many injuries happened am talking from the reality. Even the teachers have no experiences but you may find… for example if they increase the number of sport class to be 2 or 3 par of it to be theoretical and the other part is practical so the student would have the sport mentality. Teach them the games and so many other things. Honestly I finished the high school 12 years ago and through it; the coach gives them the ball and tell them to go and play [Male 1: Interruption: yes right]. Even some students keep waiting for the sport class so they can go and have fun somewhere else…

M: We need also to provide a motivation for doing exercise for the young and not the adult. Instead of putting 50,000 prices, I would put 10,000 or 5,000 and in that case the young boy would want to score these points in all sports, such a simple price to stimulate youth, I knew one program in foreign country “Coach Carter”, he linked sport with marks the more students practice the more marks they get. So this is with the young students. For those who are adults: Doctors who are working and you are loading them with work, so provide them with a gym. Not only for doctors, go for the education and I am not saying that they put expensive devices, but at least you need to support your employees.

- **Role of HCPs**

M4: I have suggestion, if any one from the doctors here is obese to quit from his work,

M4: yes, the doctors should be the first one, I see one who is obese and like this, he is a doctor.

M4: My first suggestion is they should make for us logo and start on it, so they will be the role model*, a quote in Arab society says ‘the door of carpenter is broken’*, this cannot be.

M2: that is good idea

F: It is better to encourage this physical activity in the health center.

Male 4:On long run investment, it will impact on society for example, the treatment cost of diabetic or any other diseases, and so it is profitable by all measures. Do not belittle a club that will be suitable with the weather. If you did that, you will pay money on those who are diabetic, stroke or other chronic diseases.

b- **Group Support**

M1: especially if there was in the place something that encourages you

M2: exactly yes.

M1: I mean on your own you would get bored but when you find other people with you present there

M1: you would be encouraged to practice

F: so exercise is important so that one stays healthy even if her reached older age.

F1: I suggest for example each one has a sport hall in his house, or in compounds there be sports hall or in neighborhoods, each neighborhood there would be places they call them green areas for people to walk or play.

F4: make groups for example that would encourage each other; volunteering groups, the girls gather and form sportive clubs for them, volunteering groups encourage people, pass by people and encourage them, they would have a logo or something that would encourage girls and don’t get discouraged. They would be the main point.

F3: you can take it as entertaining such as to go with friends and use bicycle, so it can be entertaining and exercise at the same time so the body can get benefit from it. You can walk from building to other even though weather is not helping but at winter you can do at the university. That’s all.

F: and to be around with people who like movement and practice exercise, this is for me.

M: So, there is deficit and the reason for this deficit is that there is no encouragement for the young people like putting advertisement for them in this aspect.

Male6: young people are always together, if you target some friends, they will come all. For example every sport complex target group of young people and talk to them in this subject, if they are all together they will all go together. So that may help the young people.

Male6: yes. Targeting the group who stay together, they are usually friends

M1: first of all “Healthy mind is in a healthy body”, second of all provide places for sports only across the country, and to tell people to practice sports for your own health.

M3: I think to increases trainers at gym, they should be available for the encouragement from the trainer to the youth not for financial issues.

Female1: it’s not from government it personal motive, exactly if person would like to do sport from time to time he will do it. if he leave it for a while and return back to practice f he will do it .You don’t need someone to tell you do sports or motive you, it’s from yourself if i had time i will practice, i I’m able also i will practice also , so if person did not want to do it he will not do it.

F: They can do that for a month of 45 days for a group who will encourage each other, so I and the sister can go and know each other and this will encourage us that we are going on this day to the hall to practice exercise. We have this hour for exercise, you will feel nice when you are with people and also you will forget about your pain as there is a specialized hall for women of this age. Of

F2: Subhan Allah, you feel company when you are with people, and take experience from older people.

Male1: it is nothing really, This sports day became nothing really.

Male3: yes it is but

Male1: people don’t go anywhere.

Male2: it became like the mother day. To bring a gift for your mother and you take the money

Male 1: That is what they think

Male1: this day doesn’t increase the sport in the country, but decrease it

Male3: the young men take as vacation day and drive around with their cars. If you enhance running from this here to there, there will be amount of money as award

F3: or they could have activities that includes all the members of the family. If the mother likes to take her children and her husband would be there with them, of course they will be convinced and would like to join if there are such activates.

F1: they should make us like to exercise; I think,

F1: for example some people don’t like movement because there are some difficult ones, they can make it easier for us… they can bring ~~by they want~~ trainers that would show us exercises that are beneficial for us that we can do at home, because there are lots of people who don’t know that.

F2: sometimes you help by advising them to move and walk instead of staying at home. Try to move and walk; try to do anything.

Interviewer2: so you think movement inside house can be considered as an exercise?

F2: yes it could be considered as exercise. [F3 interruption: for me I don’t consider movement at home as an exercise because you will not burns enough calories; you got my point]

F2: but movement at home is nice.

F3: well still it’s better than sitting all the time at home but still it’s not an actual kind of exercise. It is supposed to be another kind of sport rather than house work. Something with more movement; like swimming for example; you know. Swimming is one of the enjoyable activities.

Interviewer2: ok according to your age group what do you suggest for them?

F3: at least go for walking.

Interviewer2: walking! [Other participants also agree with F3]

F3: walking is the easiest thing people can do. If the weather is not nice and can’t walk he can buy the walking machine and use it at home. He can even put it in front of the T.V so to get motived to use it.

F1: even if it’s inside home. And if they have a big house they can walk around inside. So what is wrong to walk inside as they have a big house!?

Interviewer1: do you have anything to add regarding the sport; any ideas? For example your colleague suggests providing GYM hall room in the work place and to specify a certain time during the work to exercise that would be better?

F1: I believe when we get to know people, they would go together and practice the same activity or something like that.

Interviewer2: you mean to be grouped.

F1: yes.

F3: yes, because the company sometimes is the reason to get fat or being slim. Have group encourages all of us to lose weight; did you get my point.

F1: even her at work [F2 interruption: right even at work] they can ask us for a walk or go to this room for activity or so that would let to move.

F3: or this watch for example. It counts the steps for you.

F3: so it motives you see how many steps you walk and encourages you, if you did not walk that much steps and so on. it helps.

F2: it’s right what you say (F3) it is a good choice

F: Groups; for example each group in the neighborhood for example each group in a certain area there should be a sports club a special club for example for ladies, you will find all encouraged, otherwise all clubs you find them far away something in Doha, further. But in each area, if there is a sport club it will motivates that all people in this area to practice sports.

M:So I say if the person keeps praying regularly at time with other Muslims this is sport it burns so much calories and feel a psychological relief.

M2: I strongly advise them to do sport

M1: my suggestion in general to make activities constantly, to ride bicycle, to jog, marathon

F1: as the winter is coming; Aspire Park is a nice place we go there to eat, people go for walking and we go for eating [laughing] when we go to Aspire Park we supposed to go for walking not to sit and eat. That is what I always try to say... When I tell them to walk they would ignore and say ok ok ok, I tell them “go and walk to lose weight!” they just like ok; ok; ok *Ensha-Allah* and then they all go. And this is something good

F4: because you are alone you don’t know and you want someone to get motivated; Lets go and lets do [F1 interrupting: why don’t you gather there; Aspire is a known place and everyone goes there you can gather there. Everyone goes there to Aspire these days. It’s good to go for a walk even these days the weather is beautiful. We always do that].

F1: Aspire Park honestly is the best solution. Everybody satisfy with it. [Side talking and door opened] we don’t want to go to malls; you know what I mean, its better if one takes the advantage of this weather!

F3: Yes the weather.

M3: and as advertising they can add health education, so if someone forgets other one will remind him

Female 6: yes if they are in group from they will encourage each other especially males,

F: It is also good if we as group together participate in activity and make it as competition between us as a female until we reach ideal body shape. To have fitness; this will increase my strength to continue practicing activity. I need somebody to encourage me, if I do not have any one of my friend with me in the club; this will discourage me to practice physical activity. I will think why I am going to participate alone and to go to the club an hour before the lesson start. I will say no, I will stay at home and bring machine at home to practice the activity. Then when I bought the machine, I did not use it.

F: The company they work at it they should support it. Well when you see employee in a good health and enjoying the lightness and move that gives them more energy. If I am the owner of a company and I found an obese employee but his working is good and his brain is very high this is jewel I cannot lose him. He will not give the production but his brain is good. I want to utilize his brain in the job. What should I do? I will support him to exercise. Guys we are enrolled the company in a center with reasonable price so that the employee will pay it. This will encourage the whole employee to be in group and they will noble competition between them to make encouragement to make them to give energy in the morning and at the end of the day. They feel it’s a recreation not like a type of routine. No this journey I don’t want to go. If my friends are going with me and this one his weight is that and the other is thiswhy I am like this I have to be diligent to reach.

c- **Role of Media**

F: pass by people and encourage them, they would have a logo or something that would encourage girls and don’t get discouraged. They would be the main point.

Male1: for example the advertisement “how did you look and how you look now”. This is the first thing they do in the gym. From day 1 in the gym, you take a photo of yourself, ok? And after 2 month take a photo again and look the difference, after 3 months look the difference, after 1 year or 2 years, look to the first photo and the last photo and look to the difference. When the people see what you made to yourself in your life, this will encourage them to do what you did.

M: The sport awareness in general

Male5: through competition like in the *Aldori and Alkas* (T.V channels).

M:Through *Instagram*, the social media, we started to see pictures of this, we would say wow this person is strong, this exercise is what? This is this type of exercise, ok, how do I reach this level? I go to Gym, ok how can I do that? Vision, I saw those who surround me are like that so I started. So these things open a big door.

M5: for me regarding the suggestions, for example, the time that we are now in, most of them go to hospitals and health centers, and they treat with medicine and others, meanwhile the disease like hypertension and diabetes and most of the diseases that can be treated mostly by exercise. Hypertension can be treated by decreasing weight, which will decrease blood pressure and pressure in the arteries, for example if this patient practiced exercise, he will decrease in his weight and so will increase in the percentage that he will get rid of hypertension or the percentage of not getting the disease. The health centers and the hospitals should add the exercise part in them not only the medicines, because most medicines have side effects, it is used but has side effects, so we as health care facilities, should add this part as an exercise program for the hypertensive and the diabetic patient.

F: marketing for other clubs, they should give people other option so if that club not suitable you will go for another one,

Female4: organize your time, prioritize first of all, Allocate Time for mental rest, because sport play major role in reducing stress ,anger, Psychological pressure, the sport relief all of that then you see what you like ,

Female4: to make awareness and event at universities and schools or even in ***Montadayat*** (social media) , posters on physical activity and what is the benefit at the mall about or events on physical activity that talks about the benefits of sports, to make people aware. Physical activity helps to focus, and how person can continue his life along with physical activity.

F: We must put inside the girls the sense of sport and make it a habit. As we go shopping, it Is ok… the woman take her children and we will go for practicing sport etc.., Since childhood, and when she grows up, when she reach 24 years old, she will not be shy and she , no she will be happy and she is familiar with it.

F:So they need to provide these things and what they provide from activities to the boys, they should make the same for the girls, so they will be involved in that. They put that inside them and this will make big difference since childhood.

F: I try to walk, because if the person falls especially the elderly, it is over, if he stayed in his place, he will be sluggish and the whole world’s diseases will affect him. He has to walk, if he cannot do exercise, he should walk, walking is enough, walking is enough. He will burn some of what is in his body, this is for the elderly. There are some people who would say we passed this stage and this is for the young. This is not for young people; this is for every person who has a live body, his back is alive, his knees are alive, it is not that they are old in age, then that is it, I keep aside all the organs, no. I should give them a space for that; if I cannot exercise then I should walk.

F: I previously have seen a video of elderly women of our age and heavy, they were walking on the treadmill with music and you see the trainer is active who is giving you the energy and the push for you to move. Where are we? They are women of our age or even older like the sisters here, you feel they have energy and they are active, where are we? How are we living and how they are living? Look they live longer not like us [laugh]. When we reach 60; women will not be able to move, sitting on her chair, and she became obese…

M3: how will motive you as foundation, hospital?

M4: they can make advertisement.

M2: They themselves

M3: and as advertising they can add health education, so if someone forgets other one will remind him

M: well it the same idea but we can make some advertisements; awareness groups for the importance of sport for human.

M: an awareness campaign for example on the T.V or advertisements about the same idea that show the difference between the guys who always play sports and the guy who is always lazy, you know an encouragement, encouragement.

M: the advertisement.

M: we can make champions with prizes, advertisement. And that it is

M: Regardless we must focus on the schools and children because we have the biggest overweight percentage ever in Qatar among children.

M: such as awareness programs for group age (1-20yrs) that is vertically, whereas horizontally in providing training for awareness,

M:media and in education

d- **Cultural Support for Women**

F4: I’m with providing center that is specialized in promoting exercise, a club for young females only for example, controlled by a special department and professional coach not any person to be there,

M: Regarding the shame point for women or anything, there are women sports club that are only for women that are not mixed and they can practice exercise freely there.

F3: they can make for us clubs, [interruption from f2: yes particular clubs for women)

F3: gym, as there is gym for men’s they can help women and do gym for them, gym should be on the expense of country, so instead person will go and pay money on gym country should provide that centers ,clubs for women[ interruption F2: yes for Women]

F2: to do competition, marathons for women only,

F2:They can provide a hall for women for example

F1: women and men.

F2:They can do that for the age 45 and above with trainers and with small registration fees, because not all people can pay expensive.

F1: the suggestions are to increase the number of health and sports clubs, and sometimes the fees for example to be reduced, sometimes could be encouraging fees not commercial, now most of the clubs; women wouldn’t go because the fees are commercial. Groups; for example each group in the neighborhood for example each group in a certain area there should be a sports club a special club for example for ladies, you will find all encouraged, otherwise all clubs you find them far away something in Doha, further. But in each area, if there is a sport club it will motivates that all people in this area to practice sports.

F1: and low fees.

Female 4: to provide the exercise machine with reasonable prices.

Female 5: privacy for women .Also the public machine should have some privacy for the women. When you go to Aspire and its empty you can play there but in the presence ofsomeone it’s difficult. There is walking place in Al-Rayan but it has been closed; it was wonderful. Al-Rayan walking placewas more comfortable than Aspire.

F: used to prefer if there is close one for women; we had one but they closed it now. We want special place for women wearing Hajab which it is difficult to take it off in front of the presence of male.

F: Make more places especially for women.

Female 1: Suggestion is like; the clubs should be special for women and the working hours should be suitable for the employee.

F: Especially the old women who sit at homes, I have my mother in low with me I always ask her to go out and we registered her at the diet center and then she used to go and then got laziness. You know the elderly women they got laziness when we go back and force.

F: There should be section for female different males. Not only fitness but also the numbers of park places, you find Aspire or Cornish is for away from your residence.

M: And also it should be a nice place to attract youth for GYM, you know there is GYM that is well designed

M: They can make a special building or a special club, a very big building in Qatar just for sports games, there is no GYM just only games.

e- **Family Support**

M2: I think once parents have children or the kids reach 10 years parents should implant love to be something, if kid says I want to play father should say yes I will take you and make his kid feel he is doing something right, so kid will have the impact of love to play, to do any correct thing.

Female1: The mother can take her daughters and go!

F3: or they could have activities that includes all the members of the family. If the mother likes to take her children and her husband would be there with them, of course they will be convinced and would like to join if there are such activates.

2- **Promote Physical Activity**

F1: exercise is important that we do it every day some people, you find one person in his 60s still doing it since he was little other one who didn’t do sports, would reach his 40s and you find him as if he 100 years, so exercise is important so that one stays healthy even if her reached older age.

F1: I suggest for example each one has a sport hall in his house, or in compounds there be sports hall or in neighborhoods, each neighborhood there would be places they call them green areas for people to walk or play.

F3: from the country for example, from little age, I mean mandatory according to the group age, not give them much pressure but make them do sports. But how I don’t know! I will think!

F: for example. Or they would increase the number of contest like the biggest winner; add more contests; another contest for losing weight, another contest for fitness, add more sports; football, things like this, there should be something that would encourage us to do sports. Not sitting and using the bus to go to another building and coming back. There is not any movement at all.

F1:….. [Laugh] I don’t know, if each person starts from himself, she said she doesn’t have time to practice, so they can allocate time to practice at least 30min sure they have 30 min free, for me I have so many free hours, I do have extra time and never get use it, if I walk daily 30 min around house it could increase gradually until became a lifestyle, habit to walk every morning when I wake up. Gradually person we will be able to practice.

F2; for example we can start at university, no need to use bus from college to other, instead we can walk as type of sports

F2: yes weather, but they make everything easy, yes weather is hot but movement is good, they can encourage to use bicycle more and reduce using of cars.

F: also to add extra things such as practicing outdoors and see the nature so the girls would be motivated

Male4: motivate the young people more for doing sport.

Male4: because a country like Qatar, it has a lot of sport activities but there is shortage in motivation and make the sport likable. The sport awareness in general

M: there are ways to motivate the young people in indirect way. For example walking in the university, between the colleges, there can be a project for providing the bicycles using it for passing. It is the same but in the indirect way. It is doing sport and at the same time it is a mean of passing

Male1: there are universities here in Qatar set up a day for a championship for all the sports. For example there is volleyball and there are 20 teams in the universities and there is playoff between them. I heard about the strong man. They started with light weight with 15 participates and they start to elevate it. Then they increase the weights and elevate until they reach the last 2 or 3 participates and they playoff until they reach the maximum. They give awards and academic recommendations and you will have it together with the graduation certificate.

Male2: most of the young people don’t accept that the sport is … it is rarely when you find it very few. If you see the volunteering work in Qatar who did it start? It was rarely when you see the Qatari young people go for volunteering like volunteering in Qatar volunteering company or Qatar University they all go why? Because they know their own benefits from that and they know there are experiences and relations. He will improve himself. It should be the same for the sport, motivations and benefits at least to be financially or something let the young people care about it. After this stage, the young man spontaneously will say no need to take awards every time. When he adapts this, he will do sport spontaneously.

M1: Other than awareness. To increase it at schools, make PE classes more, increase the importance of doing exercise at school. For example, for me, at my school, if there was a program after school, an exercise program, that would be the beginning, from here. Let say leave the school hours, the school schedule can finish at 1:30-2:00pm, and for a certain days in the week, the student can stay from 2:00 to 4:00pm even till *Maghrib* (afternoon 5:00pm), there is no problem, there is transportation where he can go back home and at the same time he is under school care, he can be in the exercise programs, games. This is because the tournaments at schools in different sports is there so if we put something like that at schools, there will be challenge and this will grow and the student will have the talent and in the end this will appear as all the people would be doing exercise and the awareness will increase. In addition, the country will find talents in different sports; this is because it started from school. Physical activity is 2 to 4 hours per week. You can bring up a talented footballer who would be the world champion in football, for example.

M2: We can encourage them through games, when there are more games, when there are more games there would be more motivation. Like in *Katara*, there was a game that was done in *Katara* and there were lots of people there.

M3: when there is prizes, like when someone comes and play with us we would give him some money. Something like that might encourage.

M2: I think once parents have children or the kids reach 10 years parents should implant love to be something, if kid says I want to play father should say yes I will take you and make his kid feel he is doing something right, so kid will have the impact of love to play, to do any correct thing.

Female4: organize your time, prioritize first of all, Allocate Time for mental rest, because sport play major role in reducing stress ,anger, Psychological pressure, the sport relief all of that then you see what you like ,

Female4: to make awareness and event at universities and schools

Feamle1: they should impose the sports. Not only at the school. For example at primary or elementary and high school it’s ok if i did not play sport. This is completely normal. Yes. In our time were sport class was mandatory, not optional but now the student does not want to play sport, and make any excuses to not play sports.

F: balance healthy diet,

Female1: if there is no place to do sport. There are things to do in the house and it is very simple. We sometimes tell those people who have stairs in their houses to use it if she has no bone pain, this is also a kind of exercise. If she gives a day off to the housemaid and she did the work (laugh) because hovering the house will burn many calories.

Female3: but as much as the house works burn, it will not burn enough calories as a 1 hour walk. It is muscle effort.

Female1: but there are type of house works burn these high calories

Female3: like what?

Female1: the hovering of the house, hovering burn. That is why you find people in European countries, who don’t have housemaid, are very fit, very fit, only from house work. Of course the people who have gardening, who work in gardening, that burn a lot. Just to reduce food with it (laugh).

F: I just advise people*. Ma Sha Allah* people her have housemaid or two, you may give… ask her” you cook today and I will clean today or to do something else, as for example”. For those having houses, she may go around the house; they definitely have yards in their house, to wear her shoes and to turn around. I have noticed that when I was on vacation on another Gulf country, I have seen the ladies wearing trainers and they were at mall. They turn around the mall with AC and cool. It is cheap and cost nothing and you can leave your children in a place to play in or to leave them with the housemaid and you turn around the mall. If you turn around the mall 10 times, this will be …..

Female1: I think to educate the people is very important since they are young. If we say if you allow the children to put in sport field after that they will care about the sport and they will keep about their bodies, because the fitness starts from the childhood. It is difficult to go to a young man 21 or 25 years old to tell now you go and do sport. It is difficult. As much as earlier it will be better.

F: The foundation should… it should start from the school. There should be a time for children’s sport. It has to be mandatory. There should be exercise and fitness classes in the school.

F1: there are many people, especially the elderly, they don’t like exercise, and they say I am old, it is over, I am passed this stage, they would say these words, we are passed this stage, and exercise is for the young. But we have to say this is not only for the young, those who are awake with a good body and strength and can exercise and walk, he should do it and walk. It is exactly the same as the person who gives himself and stays on the bed. If he stays on the bed, he will deteriorate, it is over. Yes right now, I cannot walk but I don’t stay in my place. I try to walk, because if the person falls especially the elderly, it is over, if he stayed in his place, he will be sluggish and the whole world’s diseases will affect him. He has to walk, if he cannot do exercise, he should walk, walking is enough, walking is enough. He will burn some of what is in his body, this is for the elderly. There are some people who would say we passed this stage and this is for the young. This is not for young people; this is for every person who has a live body, his back is alive, his knees are alive, it is not that they are old in age, then that is it, I keep aside all the organs, no. I should give them a space for that; if I cannot exercise then I should walk.

F3: when the lady moves in her home instead of the maid but she goes to the kitchen, do this, carry this, stand and do that, this is exercise for her, this is at home, if you have a maid, ok leave her at home to work, but you will be at the kitchen, with your children, with these, she should not sleep before them (family), she should check, prepare for them the food and pet them. This is exercise, and this is what people do, Hamdulleah.

F3: they should do exercise, exercise, they should do exercise at home, the most thing is at home, your home with your kids.

F3: Also on the State level

F3: Our family in Emirates, they have maids but they are managers and they don’t know the meaning of sitting (they don’t sit), They don’t read, they did not leant and he doesn’t sit, when the kids go to school, they would go to work, sewing, do this, this is exercise (*Sedu*) (is a type of fabric Bedouin traditional tent )and you do this, do this for the country and encourage them. Here, they don’t do that for us, we all are sitting at home, we only if so go to do exercise and come back. They should do that for us. Hamdulleah life is long and there is no deficiency here and there is good stuff here, but they should do that here for us.

F1: they should make us like to exercise; I think, for example some people don’t like movement because there are some difficult ones, they can make it easier for us… they can bring ~~by they want~~ trainers that would show us exercises that are beneficial for us that we can do at home, because there are lots of people who don’t know that.

F3: to provide more information about exercise and its benefits

M: for example I can’t stay setting all day at work, setting on the chair, I can’t stay setting, I should every half an hour, hour to go out move about an hour and come back. This is called -------. One should create activity in his work, park your car far from the parking, instead of the elevator use the stairs this is called physical activity.

M2: it is not necessary, you can in the house there is AC bring a machine you can walk on anything to walk,

M3: yes, force private sectors to have periodical checkups for the employees; blood test, I can do it on my own I move around normally I don’t complain of anything, but when I find high blood cholesterol, the start of a problem, the start of fat on liver so I would fear for myself so I would go to sports, I know that doing sports would be the only way and to organize eating, so I put point which are regulatory for private sector that at least make Friday a day off. Obligatory, and after that it should be implemented you would go to the employee ask him he will say he is working Friday because he wants but it is not true, he is forced, not directly but he is indirectly forced to work on Fridays

M2: a lot a lot for example do every month one sports day give away prizes to motivate people this is very nice but the most important thing is providing the time.

F4: that what? The sister here said she wishes in the work place to be there a special place for sports so that one wouldn’t get bored, in the time of the break he could go move in that time, first thing he will refresh his activeness and Sobhan Allah sports give you new thoughts and new productivity and makes you fresh so this helps better in regards to productivity at work and not being lazy

M1: well I advise my brothers that it is better to do something little and keep on doing it than to do soM4: the most important thing the availability of tim much and not to keep doing it , be committed for short time even if t’s half an hour… for example I have part of Holy Quran that I read while am walking to the mosque.

M: So I say if the person keeps praying regularly at time with other Muslims this is sport it burns so much calories and feel a psychological relief.

M: the most important thing is “*stomach is the house of disease*”. Eating well is linked with exercise. Secondly exercise is divided according to the ages. A male with 16 yrs old will not do exercise as a 40 yrs old man. It’s an age thing. The exercise that is suitable for all ages is walking. I park my car in my work place and then I walk till I reach the Sheraton with God’s help and come back again.

M2: yes; when I kept on walking program with moderating my food. There is a hadith saying” eat your whole breakfast, part of it for your lunch and leave your dinner for your enemy”

M2: walking. Everyone and must walk according to his age.

M: have balance diet,

M: to walk or go to beach or club to practices sport like that at weekend

M1: Yes, to teach it for all age categories, those who are younger and older than me

M4: as Arabs we do have secondary things to the extent of long term, which includes exercise, exercise is one of them. Regarding our age group, we are in extreme threat, which is we were not raised to love exercise or even the healthy behavior. We have to learn from our mistakes so we lead and direct our kids at early stage, from school age to motivate them to exercise, and as my brother said this is not the government’s responsibility , from my point of view, the government’s responsibility is secondary, responsibility fall first on the individual , also it depends on the environment that he was raised in, so his family should teach their kids the love of exercise and the healthy behaviors in general, and the government to encourage , so responsibility falls on us.

M4: So we should learn from our mistakes and build in our kids when they are young, at school. Usually at school the physical activity in Arabic country is secondary and the physical activity teacher do the activity in the morning for the queue that is only it. There is no…, sometimes the physical activity class is only once per week, or two per week. yes, from younger ages so what we fell into will not repeated in other generation

F1: as the winter is coming; Aspire Park is a nice place we go there to eat, people go for walking and we go for eating [laughing] when we go to Aspire Park we supposed to go for walking not to sit and eat. That is what I always try to say... When I tell them to walk they would ignore and say ok ok ok, I tell them “go and walk to lose weight!” they just like ok; ok; ok *Ensha-Allah* and then they all go. And this is something good

Female 3: No suggestion everything is fine. If someone wants to do exercise has to encourage oneself and go ahead.

Female 1: Clubs or educate them through sending SMS via the phone and participatethrough places like this conferences and lectures which is encourage little bit. Especially the old women who sit at homes, I have my mother in lowwith me I always ask her to go out and we registered her at the diet center and then she used to go and thengot laziness. You know the elderly women they got laziness when we go back and force.

Female 1: yes the distances and walking but the opposite the old women but we are thanks God even I would love the older so that would not effect on their aromatize. Even when I go for walking at Aspire I see even old women a very old, oldandwhen I see them I get encouraged. Some of them are grandma and they circle around wearing sport shoes they do not walk with normal shoes. At the opposite I get encouraged more and I walk more. I can say Aspire;thanks God for the walking space

Female 4: I see that the clubs encourage people to practice even for the men to commit because he pays money. He commits that at a specific time he is going to change clothes and go to the club. This encourages him like going to the duty, during this time I am at the club till it become a habit.

Female6: my husband participated in many costly clubs for one to two months and it showed good results along with diet program at home. He loses weight, but after sometime he feels boring even he paid money. Don’t think of the money you paid? Let me talk honesty, when the person pay money, he should tolerate al the difficulties until he reach his desire with his money. You force yourself and cut from the household expense your money to reach your desire so why did you stop? The husband said I do not have intention.

Female 2:really it is the intention

Female 3: he needs someone to encourage him and be with him

Female 4: That is why he decided to go to aspire where there are many peoples and might bring my friends and make groups to encourage each other but it is difficult to contact them and until he reaches his turn.

F: For example if there is smart one at the school but he is too shy to tell the teacher the information so whyI do not involve him with it with the group but if he founds the group in the class there is competition and everyone wants to show he’s better than the other. Each one will compete to tell the right information. Each one will say because he wants to prove to his group he is the better.

M: And try more to encourage the youth for sport like the National Day of Sport

M: we can make champions with prizes, advertisement. And that it is

M: yes with prizes the most important thing is to motivation

Male 6: you can’t say you’re above 8 years old so stay at home; you’re not allowed to play go and check different game. As I know the child till the age 15 must play all kind of sport after a certain age he can choose the game he is good at and the game he likes it. This’s the father’s role. I don’t force him to play a certain games. He is automatically after 18 or 20 years old will choose the game that it has the benefit for him. If he didn’t get the advantage of it he will chose the game he loves. He is not obliged to join a club he can make his own team.

M: I mean if they want so they can make a foreign team just only for foreigners here in Qatar and let them play against each other and to go out of the Qatar it’s just. They are foreigner they shouldn’t go out from Qatar but at least they can bring the best players put them in one team so they can make champion [Interruption and group talking]. Honestly why they prevent it because they want to increase the number of Qatari’s players and then they can reach to the national team and can bet abroad but we’re the foreigners here, they made playgrounds for us but sometimes we need little motivation, some champions here and there so we can…; so if they can reset these rules even if… so they can make more champions and increase the number of the teams; let them bring some people to play so.

Male 5: For me there are suggestions because we have major and essential problems with secondary problems. First of all we must be instilling the concept of practicing sport in schools. We have schools; if you can see it; where the sport’s class is once weekly and even maybe the coach may not send the students to go out and play; although these students have energy. The student needs to disperse this inner energy in a form of sport because if the student didn’t disperse the energy he has may lead to dispersed in others things

M: It may enhance the sport and would develop it for better, these are the most important. Regardless we must focus on the schools and children because we have the biggest overweight percentage ever in Qatar among children. This is a big problem it may lead them to have diabetes, diseases and hypertension are because of no movement. We must encourage them because it so hard to... The European’s community has the culture of taking care of self but in Arab no and vice versa once they go back home from school they find a dish of rice he start to eat and sleeps no this is true no this is real. So may find most of the students are *Mash-Allah* and the weight is… so I believe if we took care of these things I think we we’ll have a healthy environment and better.

M: The proof of that is the state is working on that. It organized champions on the highest level almost all the events.

M: Ok God bless them but still we need to focus on children because these children are going to be leaders. If those children haven’t the mentality and thoughtful power in the future they can’t lead or became leaders in the future. The state will relay on them in everything; in Qatar they depends on 20 -30 % in sports so if there was no development in the children who are going to be a leader. It’s a disaster

Interviewer 2: sorry but earlier you mentioned that in the school they have one sport class per week. Do you think this things is because unavailability of the buildings or if they make sport to be as a subject of success or drop-outs. I mean you won’t be able to upgrade you to higher level unless you get a certain score?

Male 5: well this might be a good suggestion for the sport class.

Male 5: no the sport class is scheduled to be once weekly between the all classes so the playground is available

Interviewer 2: So the buildings are available but the issue is the lack of attention to the sport itself

Male 5: yes exactly even the sport teacher comes holding the ball and then he thought t to them go and play and after 20 or 30 minutes he comes again asking to them go. Ok but some student doesn’t like the football, why do you force them to play this game. In the hottest weather you ask them for a walk. Many injuries happened am talking from the reality. Even the teachers have no experiences but you may find… for example if they increase the number of sport class to be 2 or 3 par of it to be theoretical and the other part is practical so the student would have the sport mentality. Teach them the games and so many other things. Honestly I finished the high school 12 years ago and through it; the coach gives them the ball and tell them to go and play [Male 1: Interruption: yes right]. Even some students keep waiting for the sport class so they can go and have fun somewhere else…

M: people should be aware about the importance of sport in preventing a lot of things, if we have that kind of awareness we will have the motive to practice or have an active style .

M: You need to motivate youth to practice sport and then provide a place. We need also to provide a motivation for doing exercise for the young and not the adult. Instead of putting 50,000 prices, I would put 10,000 or 5,000 and in that case the young boy would want to score these points in all sports, such a simple price to stimulate youth, I knew one program in foreign country “Coach Carter”, he linked sport with marks the more students practice the more marks they get. So this is with the young students

M: At an individual level we need to merge between sport and health awareness from child hood for example, if he did not practice sport or became an athlete person, you will face a lot of diseases. We don’t know about the diseases related to lack of exercise until we are adults. For example, if the diabetic patient has the knowledge of practicing sport earlier will prevent diabetes; he would not have got diabetes. Also obesity or lack of exercise and sedentary life is a risk factor in everything. Thus Awareness at early age is a primary factor exactly the same as eating well so you have a healthy life, this is from an individual level.

M: It is better to have a program to walk for an hour in the morning and evening according to your capabilities.

M: I recommended balancing between diet and physical activity. As we say in proverb: A sound mind in a sound body. My mother is around 80 years old and she keeps walking and does not feel tired. She eats small amount to control her weight. My mother is not educated but she learns from her experience.

Male 1: I agree with playing sports and I do not have any ideas or request from the in charge people, government or from you, but I can see that it is not the proper time to do sports and if we wants to practice sport or not. Sports have specific people to carry it out, certain age, nature, weather, and particular foods. For example, the food I eat is different from what this person eats [pointed on other Participant]. This person from other country has different character than us here in the Gulf. We here in the Gulf have different nature, we should eat meat, we should add oil and fat. We should gather with friends and everyone insisted to invite others and prepares the foods for them (hospitality). Our nature here in the Gulf is different than other people in other countries. No doubt that all of us are Arab and we have many commonalities. Long ago there people were in different places, from Africa, Gulf, Yemen, but now they all share the same life together. Also no doubt that the previous yeas are unlike this moment, if you enter my kitchen in the house you could fine distinct food than the kitchen of this person [pointed on other Participant]. Here in the Gulf we brag on what we have. For your information, we in the Gulf have different nature, characteristics, foods, and souls unlike other countries. At present the gulf is rich country, has many goods and should continue the same way they were before. In the past the man borrow money from others to buy a sheep cost around 500- 1000QR to slaughter it and invite his friend for lunch, but now the people have many goods

Male 2: The person should manage his time.

Male 4: third for food, third for water and third for breathing.

Male 4: As I told you, the person should encourage himself by himself. The prophet said third for food, third for water and third for air,

M: so the person, either Muslim or non-Muslim, exercise is very important, without it the person will get diseased. Sports are important and as I said previously that long time ago we walked around looking for the foods by fishing; this makes the people healthy.

a- **Encourage PA since foundation age**

F3: from the country for example, from little age, I mean mandatory according to the group age, not give them much pressure but make them do sports. But how I don’t know! I will think!

M1: Other than awareness. To increase it at schools, make PE classes more, increase the importance of doing exercise at school. For example, for me, at my school, if there was a program after school, an exercise program, that would be the beginning, from here. Let say leave the school hours, the school schedule can finish at 1:30-2:00pm, and for a certain days in the week, the student can stay from 2:00 to 4:00pm even till *Maghrib* (afternoon 5:00pm), there is no problem, there is transportation where he can go back home and at the same time he is under school care, he can be in the exercise programs, games. This is because the tournaments at schools in different sports is there so if we put something like that at schools, there will be challenge and this will grow and the student will have the talent and in the end this will appear as all the people would be doing exercise and the awareness will increase. In addition, the country will find talents in different sports; this is because it started from school. Physical activity is 2 to 4 hours per week. You can bring up a talented footballer who would be the world champion in football, for example.

M2: I think once parents have children or the kids reach 10 years parents should implant love to be something, if kid says I want to play father should say yes I will take you and make his kid feel he is doing something right, so kid will have the impact of love to play, to do any correct thing.

Female4: to make awareness and event at universities and schools

Feamle1: they should impose the sports. Not only at the school. For example at primary or elementary and high school it’s ok if i did not play sport. This is completely normal. In our time were sport class was mandatory, not optional but now the student does not want to play sport, and make any excuses to not play sports.

Female1: I think to educate the people is very important since they are young. If we say if you allow the children to put in sport field after that they will care about the sport and they will keep about their bodies, because the fitness starts from the childhood. It is difficult to go to a young man 21 or 25 years old to tell now you go and do sport. It is difficult. As much as earlier it will be better.

F: The foundation should… it should start from the school. There should be a time for children’s sport. It has to be mandatory. There should be exercise and fitness classes in the school.

M4: as Arabs we do have secondary things to the extent of long term, which includes exercise, exercise is one of them. Regarding our age group, we are in extreme threat, which is we were not raised to love exercise or even the healthy behavior. We have to learn from our mistakes so we lead and direct our kids at early stage, from school age to motivate them to exercise, and as my brother said this is not the government’s responsibility , from my point of view, the government’s responsibility is secondary, responsibility fall first on the individual , also it depends on the environment that he was raised in, so his family should teach their kids the love of exercise and the healthy behaviors in general, and the government to encourage , so responsibility falls on us.

M4: So we should learn from our mistakes and build in our kids when they are young, at school. Usually at school the physical activity in Arabic country is secondary and the physical activity teacher do the activity in the morning for the queue that is only it. There is no…, sometimes the physical activity class is only once per week, or two per week. yes, from younger ages so what we fell into will not repeated in other generation

F1: as the winter is coming; Aspire Park is a nice place we go there to eat, people go for walking and we go for eating [laughing] when we go to Aspire Park we supposed to go for walking not to sit and eat. That is what I always try to say... When I tell them to walk they would ignore and say ok ok ok, I tell them “go and walk to lose weight!” they just like ok; ok; ok *Ensha-Allah* and then they all go. And this is something good

Male 6: you can’t say you’re above 8 years old so stay at home; you’re not allowed to play go and check different game. As I know the child till the age 15 must play all kind of sport after a certain age he can choose the game he is good at and the game he likes it. This’s the father’s role. I don’t force him to play a certain games. He is automatically after 18 or 20 years old will choose the game that it has the benefit for him. If he didn’t get the advantage of it he will chose the game he loves. He is not obliged to join a club he can make his own team.

Male 5: For me there are suggestions because we have major and essential problems with secondary problems. First of all we must be instilling the concept of practicing sport in schools. We have schools; if you can see it; where the sport’s class is once weekly and even maybe the coach may not send the students to go out and play; although these students have energy. The student needs to disperse this inner energy in a form of sport because if the student didn’t disperse the energy he has may lead to dispersed in others things

M: It may enhance the sport and would develop it for better, these are the most important. Regardless we must focus on the schools and children because we have the biggest overweight percentage ever in Qatar among children. This is a big problem it may lead them to have diabetes, diseases and hypertension are because of no movement. We must encourage them because it so hard to... The European’s community has the culture of taking care of self but in Arab no and vice versa once they go back home from school they find a dish of rice he start to eat and sleeps no this is true no this is real. So may find most of the students are *Mash-Allah* and the weight is… so I believe if we took care of these things I think we we’ll have a healthy environment and better.

M: Ok God bless them but still we need to focus on children because these children are going to be leaders. If those children haven’t the mentality and thoughtful power in the future they can’t lead or became leaders in the future. The state will relay on them in everything; in Qatar they depends on 20 -30 % in sports so if there was no development in the children who are going to be a leader. It’s a disaster

Interviewer 2: sorry but earlier you mentioned that in the school they have one sport class per week. Do you think this things is because unavailability of the buildings or if they make sport to be as a subject of success or drop-outs. I mean you won’t be able to upgrade you to higher level unless you get a certain score?

Male 5: well this might be a good suggestion for the sport class.

Male 5: no the sport class is scheduled to be once weekly between the all classes so the playground is available

Interviewer 2: So the buildings are available but the issue is the lack of attention to the sport itself

Male 5: yes exactly even the sport teacher comes holding the ball and then he thought t to them go and play and after 20 or 30 minutes he comes again asking to them go. Ok but some student doesn’t like the football, why do you force them to play this game. In the hottest weather you ask them for a walk. Many injuries happened am talking from the reality. Even the teachers have no experiences but you may find… for example if they increase the number of sport class to be 2 or 3 par of it to be theoretical and the other part is practical so the student would have the sport mentality. Teach them the games and so many other things. Honestly I finished the high school 12 years ago and through it; the coach gives them the ball and tell them to go and play [Male 1: Interruption: yes right]. Even some students keep waiting for the sport class so they can go and have fun somewhere else…

M: We need also to provide a motivation for doing exercise for the young and not the adult. Instead of putting 50,000 prices, I would put 10,000 or 5,000 and in that case the young boy would want to score these points in all sports, such a simple price to stimulate youth, I knew one program in foreign country “Coach Carter”, he linked sport with marks the more students practice the more marks they get. So this is with the young students

b- **Increase the will towards PA**

F1:….. [Laugh] I don’t know, if each person starts from himself, she said she doesn’t have time to practice, so they can allocate time to practice at least 30min sure they have 30 min free, for me I have so many free hours, I do have extra time and never get use it, if I walk daily 30 min around house it could increase gradually until became a lifestyle, habit to walk every morning when I wake up. Gradually person we will be able to practice.

Male4: motivate the young people more for doing sport.

Male4: because a country like Qatar, it has a lot of sport activities but there is shortage in motivation and make the sport likable. The sport awareness in general

F: balance healthy diet,

M3: yes, force private sectors to have periodical checkups for the employees; blood test, I can do it on my own I move around normally I don’t complain of anything, but when I find high blood cholesterol, the start of a problem, the start of fat on liver so I would fear for myself so I would go to sports, I know that doing sports would be the only way and to organize eating, so I put point which are regulatory for private sector that at least make Friday a day off. Obligatory, and after that it should be implemented you would go to the employee ask him he will say he is working Friday because he wants but it is not true, he is forced, not directly but he is indirectly forced to work on Fridays

M: the most important thing is “*stomach is the house of disease*”. Eating well is linked with exercise.

M2: yes; when I kept on walking program with moderating my food. There is a hadith saying” eat your whole breakfast, part of it for your lunch and leave your dinner for your enemy”

M: have balance diet,

Female 3: No suggestion everything is fine. If someone wants to do exercise has to encourage oneself and go ahead.

Female 1: Clubs or educate them through sending SMS via the phone and participatethrough places like this conferences and lectures which is encourage little bit. Especially the old women who sit at homes, I have my mother in lowwith me I always ask her to go out and we registered her at the diet center and then she used to go and thengot laziness. You know the elderly women they got laziness when we go back and force.

Female 1: yes the distances and walking but the opposite the old women but we are thanks God even I would love the older so that would not effect on their aromatize. Even when I go for walking at Aspire I see even old women a very old, oldandwhen I see them I get encouraged. Some of them are grandma and they circle around wearing sport shoes they do not walk with normal shoes. At the opposite I get encouraged more and I walk more. I can say Aspire;thanks God for the walking space.

Female 4: I see that the clubs encourage people to practice even for the men to commit because he pays money. He commits that at a specific time he is going to change clothes and go to the club. This encourages him like going to the duty, during this time I am at the club till it become a habit.

Female6: my husband participated in many costly clubs for one to two months and it showed good results along with diet program at home. He loses weight, but after sometime he feels boring even he paid money. Don’t think of the money you paid? Let me talk honesty, when the person pay money, he should tolerate al the difficulties until he reach his desire with his money. You force yourself and cut from the household expense your money to reach your desire so why did you stop? The husband said I do not have intention.

Female 2:really it is the intention

Female 3: he needs someone to encourage him and be with him

Female 4: That is why he decided to go to aspire where there are many peoples and might bring my friends and make groups to encourage each other but it is difficult to contact them and until he reaches his turn.

For example if there is smart one at the school but he is too shy to tell the teacher the information so whyI do not involve him with it with the group but if he founds the group in the class there is competition and everyone wants to show he’s better than the other. Each one will compete to tell the right information. Each one will say because he wants to prove to his group he is the better.

M: we can make champions with prizes, advertisement. And that it is

M: yes with prizes the most important thing is to motivation

M: people should be aware about the importance of sport in preventing a lot of things, if we have that kind of awareness we will have the motive to practice or have an active style .

M: You need to motivate youth to practice sport and then provide a place. We need also to provide a motivation for doing exercise for the young and not the adult

M: At an individual level we need to merge between sport and health awareness from child hood for example, if he did not practice sport or became an athlete person, you will face a lot of diseases. We don’t know about the diseases related to lack of exercise until we are adults. For example, if the diabetic patient has the knowledge of practicing sport earlier will prevent diabetes; he would not have got diabetes. Also obesity or lack of exercise and sedentary life is a risk factor in everything. Thus Awareness at early age is a primary factor exactly the same as eating well so you have a healthy life, this is from an individual level.

Male 2: The person should manage his time.

M:so the person, either Muslim or non-Muslim, exercise is very important, without it the person will get diseased. Sports are important and as I said previously that long time ago we walked around looking for the foods by fishing; this makes the people healthy.

c- **Tournaments**

F: for example. Or they would increase the number of contest like the biggest winner; add more contests; another contest for losing weight, another contest for fitness, add more sports; football, things like this, there should be something that would encourage us to do sports. Not sitting and using the bus to go to another building and coming back. There is not any movement at all.

Male1: there are universities here in Qatar set up a day for a championship for all the sports. For example there is volleyball and there are 20 teams in the universities and there is playoff between them. I heard about the strong man. They started with light weight with 15 participates and they start to elevate it. Then they increase the weights and elevate until they reach the last 2 or 3 participates and they playoff until they reach the maximum. They give awards and academic recommendations and you will have it together with the graduation certificate.

Male2: most of the young people don’t accept that the sport is … it is rarely when you find it very few. If you see the volunteering work in Qatar who did it start? It was rarely when you see the Qatari young people go for volunteering like volunteering in Qatar volunteering company or Qatar University they all go why? Because they know their own benefits from that and they know there are experiences and relations. He will improve himself. It should be the same for the sport, motivations and benefits at least to be financially or something let the young people care about it. After this stage, the young man spontaneously will say no need to take awards every time. When he adapts this, he will do sport spontaneously.

M2: We can encourage them through games, when there are more games, when there are more games there would be more motivation. Like in *Katara*, there was a game that was done in *Katara* and there were lots of people there.

M3: when there is prizes, like when someone comes and play with us we would give him some money. Something like that might encourage.

M2: a lot a lot for example do every month one sports day give away prizes to motivate people this is very nice but the most important thing is providing the time.

M: I mean if they want so they can make a foreign team just only for foreigners here in Qatar and let them play against each other and to go out of the Qatar it’s just. They are foreigner they shouldn’t go out from Qatar but at least they can bring the best players put them in one team so they can make champion [Interruption and group talking]. Honestly why they prevent it because they want to increase the number of Qatari’s players and then they can reach to the national team and can bet abroad but we’re the foreigners here, they made playgrounds for us but sometimes we need little motivation, some champions here and there so we can…; so if they can reset these rules even if… so they can make more champions and increase the number of the teams; let them bring some people to play so.

M:The proof of that is the state is working on that. It organized champions on the highest level almost all the events.

M: It is better to have a program to walk for an hour in the morning and evening according to your capabilities.

d- **Balancing Between PA and Healthy Diet**

M: So I say if the person keeps praying regularly at time with other Muslims this is sport it burns so much calories and feel a psychological relief.

M: I recommended balancing between diet and physical activity. As we say in proverb: A sound mind in a sound body. My mother is around 80 years old and she keeps walking and does not feel tired. She eats small amount to control her weight. My mother is not educated but she learns from her experience.

Male 1: I agree with playing sports and I do not have any ideas or request from the in charge people, government or from you, but I can see that it is not the proper time to do sports and if we wants to practice sport or not. Sports have specific people to carry it out, certain age, nature, weather, and particular foods. For example, the food I eat is different from what this person eats [pointed on other Participant]. This person from other country has different character than us here in the Gulf. We here in the Gulf have different nature, we should eat meat, we should add oil and fat. We should gather with friends and everyone insisted to invite others and prepares the foods for them (hospitality). Our nature here in the Gulf is different than other people in other countries. No doubt that all of us are Arab and we have many commonalities. Long ago there people were in different places, from Africa, Gulf, Yemen, but now they all share the same life together. Also no doubt that the previous yeas are unlike this moment, if you enter my kitchen in the house you could fine distinct food than the kitchen of this person [pointed on other Participant]. Here in the Gulf we brag on what we have. For your information, we in the Gulf have different nature, characteristics, foods, and souls unlike other countries. At present the gulf is rich country, has many goods and should continue the same way they were before. In the past the man borrow money from others to buy a sheep cost around 500- 1000QR to slaughter it and invite his friend for lunch, but now the people have many goods

Male 4: third for food, third for water and third for breathing.

Male 4: As I told you, the person should encourage himself by himself. The prophet said third for food, third for water and third for air,

3- **Increase Awareness**

F1: I suggest for example each one has a sport hall in his house, or in compounds there be sports hall or in neighborhoods, each neighborhood there would be places they call them green areas for people to walk or play.

F5: now I see that obesity is increased; children, teenagers, young people, so there should be, for example in the university, I give examples all in the university

F5: if they close between the buildings this would encourage the girls to walk not use the bus because of the heat, for example. Or they would increase the number of contest like the biggest winner; add more contests; another contest for losing weight, another contest for fitness, add more sports; football, things like this, there should be something that would encourage us to do sports. Not sitting and using the bus to go to another building and coming back. There is not any movement at all.

F: and to be around with people who like movement and practice exercise, this is for me.

F4: the idea of girl practicing exercise as a view from the society, Some family are concerned that when their girls practice sport, they might be selected eventually at the Olympic games, so awareness should be given for families as it’s not necessary if the girl practices sports she will be selected at Olympics, it can only be as a hobby to empty her energy in this field. For example, competition can be conducted among girls without taking photos if girls like to share without mixing in order to preserve the community.

F4: yes but there are some communities that are conservative , they will say I don’t want my girls to join Olympics or to appear at TV and so , they can join Olympic games for woman as alternative solution were photos and camera is not allowed.

F5: to educate parent exercise is important and is useful not harmful, because when I was young I have been selected for Qatar football national team but my family refused and they said this is a shame and we cannot do that, and this is against habits and traditions

F4; I love to dance ballet but I’m afraid

F4: Gymnastics and everything.

Male1: for example the advertisement “how did you look and how you look now”. This is the first thing they do in the gym. From day 1 in the gym, you take a photo of yourself, ok? And after 2 month take a photo again and look the difference, after 3 months look the difference, after 1 year or 2 years, look to the first photo and the last photo and look to the difference. When the people see what you made to yourself in your life, this will encourage them to do what you did.

Male2: my opinion is what encourage the young people is the advertisement,

M: So, there is deficit and the reason for this deficit is that there is no encouragement for the young people like putting advertisement for them in this aspect.

Male3: set up seminars and awareness and lectures for the young people and teenagers.

Male4: because a country like Qatar, it has a lot of sport activities but there is shortage in motivation and make the sport likable. The sport awareness in general

Male5: through competition like in the *Aldori and Alkas* (T.V channels).

Male7: the awareness tools which motivate the young peoplein general. It is not necessary to reach the full muscle shape, no, at least to have sport shape body

Male7: yes, at least to be fit. It is not even important to have the maximum fitness, but at least to be sport and maintain his body and maintain his body organs in a healthy way.

Male1: there are universities here in Qatar set up a day for a championship for all the sports. For example there is volleyball and there are 20 teams in the universities and there is playoff between them. I heard about the strong man. They started with light weight with 15 participates and they start to elevate it. Then they increase the weights and elevate until they reach the last 2 or 3 participates and they playoff until they reach the maximum. They give awards and academic recommendations and you will have it together with the graduation certificate.

M4: in my opinion the best ~~the~~ thing that motivates most is the availability of sports clubs. In each club there should be someone who will give you the correct awareness. When you are doing exercise alone you can do something extra or decrease an activity, on the other hand, when you are in a club and there is a doctor who knows what is excess in your body or less, he would also know how many time per day for exercise you need so you won’t feel exhausted.

M1: Awareness is the first thing. For example, if there are sports festivals for running for exercise that include groups that address the community’s needs, any age group, that promotes exercise, that would be better because for Qatar there is nothing that would prevent it from being like any other country in exercise like other western countries for example, for them exercise is a big part of their lives for different age groups but for us if we looked now you will find that most of the people who practice are for our age group not the other ages, there is no..

M1: Other than awareness. To increase it at schools, make PE classes more, increase the importance of doing exercise at school. For example, for me, at my school, if there was a program after school, an exercise program, that would be the beginning, from here. Let say leave the school hours, the school schedule can finish at 1:30-2:00pm, and for a certain days in the week, the student can stay from 2:00 to 4:00pm even till *Maghrib* (afternoon 5:00pm), there is no problem, there is transportation where he can go back home and at the same time he is under school care, he can be in the exercise programs, games. This is because the tournaments at schools in different sports is there so if we put something like that at schools, there will be challenge and this will grow and the student will have the talent and in the end this will appear as all the people would be doing exercise and the awareness will increase. In addition, the country will find talents in different sports; this is because it started from school. Physical activity is 2 to 4 hours per week. You can bring up a talented footballer who would be the world champion in football, for example.

M4: When the country comes and motivates, this mean that it can, when a country comes and encourage it means that it can give awareness. Awareness is an encouragement to exercise and it can motivate; and motivation can be by founding playgrounds; bring trainers and things like that. But awareness (alone) is not often practical; it is theoretical in portraying pictures and things like that. If the country’s budget that it will spend, if it is going to spend it in one area, my opinion is to, motivation is more important than awareness because some people know they want to be so, like students at college, each one drew his future, I, for example, will graduate and will take this, this and that. He has the awareness but where is the motivation.

M4: where is the motivation, the movement? You know where you want to reach and know that, for example, I want to be a world champion; I want to be, to take medals in this and this. Ok, you know, but where is the motive? Motivation could be through clubs, through trainers, through picture presentation, when I see the result of what I am going to be in the end, paving my way will be stronger and faster because I know that my picture in the end will be this and it is clear. I know what I am going to reach in the end, so I will go faster and faster, more motivation and faster I will be and I will even motivate myself by myself. But, when we, we paid on the motivation, how about the awareness, awareness will come when I see my friend is this, and the society is this, I will by myself start. Through *Instagram*, the social media, we started to see pictures of this, we would say wow this person is strong, this exercise is what? This is this type of exercise, ok, how do I reach this level? I go to Gym, ok how can I do that? Vision, I saw those who surround me are like that so I started. So these things open a big door.

M5: for me regarding the suggestions, for example, the time that we are now in, most of them go to hospitals and health centers, and they treat with medicine and others, meanwhile the disease like hypertension and diabetes and most of the diseases that can be treated mostly by exercise. Hypertension can be treated by decreasing weight, which will decrease blood pressure and pressure in the arteries, for example if this patient practiced exercise, he will decrease in his weight and so will increase in the percentage that he will get rid of hypertension or the percentage of not getting the disease. The health centers and the hospitals should add the exercise part in them not only the medicines, because most medicines have side effects, it is used but has side effects, so we as health care facilities, should add this part as an exercise program for the hypertensive and the diabetic patient.

M4: things make you lazy to practice sports are the people, family, lack of encouragement, or lack of arranged facilities , also you are not doing the outcome for sports its self , its vary according to your interest, according to outcome you want it. I’m not practicing because I don’t want to live….

M1: first of all “Healthy mind is in a healthy body”, second of all provide places for sports only across the country, and to tell people to practice sports for your own health.

M4: Awareness is not about if you did not practice sport what going happen that make people to turns away. The awareness should be about what you will gain by doing exercise. That’s it.

Female4: organize your time, prioritize first of all, Allocate Time for mental rest, because sport play major role in reducing stress ,anger, Psychological pressure, the sport relief all of that then you see what you like ,

F: there is not advertisement, so I need to search and ask for the place suitable for women.

F1: health, activity.

F: We must put inside the girls the sense of sport and make it a habit. As we go shopping, it Is ok… the woman take her children and we will go for practicing sport etc.., Since childhood, and when she grows up, when she reach 24 years old, she will not be shy and she , no she will be happy and she is familiar with it.

F: So they need to provide these things and what they provide from activities to the boys, they should make the same for the girls, so they will be involved in that. They put that inside them and this will make big difference since childhood.

Female1: I think to educate the people is very important since they are young. If we say if you allow the children to put in sport field after that they will care about the sport and they will keep about their bodies, because the fitness starts from the childhood. It is difficult to go to a young man 21 or 25 years old to tell now you go and do sport. It is difficult. As much as earlier it will be better.

F: I try to walk, because if the person falls especially the elderly, it is over, if he stayed in his place, he will be sluggish and the whole world’s diseases will affect him. He has to walk, if he cannot do exercise, he should walk, walking is enough, walking is enough. He will burn some of what is in his body, this is for the elderly. There are some people who would say we passed this stage and this is for the young. This is not for young people; this is for every person who has a live body, his back is alive, his knees are alive, it is not that they are old in age, then that is it, I keep aside all the organs, no. I should give them a space for that; if I cannot exercise then I should walk.

F: Of course the kids, we have now obese kids. There is increase in obesity among kids, they will also need this in addition to the presence of a trainer who will encourage you to move this muscle.

F: I previously have seen a video of elderly women of our age and heavy, they were walking on the treadmill with music and you see the trainer is active who is giving you the energy and the push for you to move. Where are we? They are women of our age or even older like the sisters here, you feel they have energy and they are active, where are we? How are we living and how they are living? Look they live longer not like us [laugh]. When we reach 60; women will not be able to move, sitting on her chair, and she became obese…

F2: there could be awareness or brochures with tables for examples that contains each exercise and its benefits

F4: or would provide awareness in schools and universities.

F1: they should make us like to exercise; I think, for example some people don’t like movement because there are some difficult ones, they can make it easier for us… they can bring ~~by they want~~ trainers that would show us exercises that are beneficial for us that we can do at home, because there are lots of people who don’t know that.

F3: to provide more information about exercise and its benefits

M3: the country should use its power; first thing to explain the aim of doing sports,

F2: sometimes you help by advising them to move and walk instead of staying at home. Try to move and walk; try to do anything.

F2: almost same answer as hers, spreading awareness about the dangers that affect the woman because of lack of doing sports, spread awareness in between people, like for example this thing is good like this is good ----- great.

F4: that what? The sister here said she wishes in the work place to be there a special place for sports so that one wouldn’t get bored, in the time of the break he could go move in that time, first thing he will refresh his activeness and Sobhan Allah sports give you new thoughts and new productivity and makes you fresh so this helps better in regards to productivity at work and not being lazy

*F: Masha-Allah* the state is developing the sport. There is awareness about sport for the same thing diabetes and hypertension. Things that if you have it you may get better and get cured. We hear a lot and watch the TV.

F4: suggestions! The awareness is now *Mash-Allah* nobody isn’t doing exercise or not eating healthy or not even paying attention to these things or do investigations. We all need to do investigations. One must keep these things in his life as programme in his life. Sometimes I advise people even in the sport day there is awareness. They do blood sugar level test for you. I get motivated honestly I go. I’d like to check my blood sugar level, my blood pressure. I l do dental checkup. I like to do to check up Thanks God. You must not to get scared anything.

Female 1: Regarding the sports hereMash-Allahthey did their best. They did it well. They create the “Sport Day”.In the school the sport class became very important. The boys are very keen about it and the teacher as well...

Female 2: The obese kids like my daughter and my daughter-in- lowMash-Allahthey register them in Aspire. They sent us the paper telling us they want them to join. They started to follow and check-up for the diabetes. Honestly we thank them the schools for thatMash-Allah.

Female 3: Even the teacher told me that your son is fatand he has to join...but she did not register him. She told me he has to eat healthy food and since that timewe stopped eating eggs, nuggets or burger and we start to eat healthy food. She told me when he grow up he will become tired. He is in the first grade now when she saw him he used to be fat although the food at the school is healthy.

Female 4: I will complete what she said even in the school they don’t sell candies, or chips or Pepsi for children. And then they sell things with good advantage like cheese and also the fruits.My daughter told me that they sell fruits and vegetables in school.

Female 3: apples, cucumber, carrots all available in the school.

Female 4: all of these things are good they encourage the children themselvesfor healthy food may; God guide them.

Female 1: yes the distances and walking but the opposite the old women but we are thanks God even I would love the older so that would not effect on their aromatize. Even when I go for walking at Aspire I see even old women a very old, oldandwhen I see them I get encouraged. Some of them are grandma and they circle around wearing sport shoes they do not walk with normal shoes. At the opposite I get encouraged more and I walk more. I can say Aspire;thanks God for the walking space.

Female5: HMC made a program about the 500. I already register and this already consider as encouragements for the employees. It was nice action from them to encourage the employees. I think if every occupation department, not only HMC, but also the schools, private organization should encourage their employees. If the organization cut 200- 300 QR from each employee’s salary to register him in fitness club to encourage them.

M: well it the same idea but we can make some advertisements; awareness groups for the importance of sport for human.

M: an awareness campaign for example on the T.V or advertisements about the same idea that show the difference between the guys who always play sports and the guy who is always lazy, you know an encouragement, encouragement.

M: the advertisement.

M: we can make champions with prizes, advertisement. And that it is

Male 4: They can aware people, the youth of diseases that can be avoided if they were playing sport and also taking care of kind of sport.

M: I feel like all the care is about the football. For sure they are people who love volleyball; they are people who love basketball. The care should be equal to all kind of sport not only for football

Male 5: For me there are suggestions because we have major and essential problems with secondary problems. First of all we must be instilling the concept of practicing sport in schools. We have schools; if you can see it; where the sport’s class is once weekly and even maybe the coach may not send the students to go out and play; although these students have energy. The student needs to disperse this inner energy in a form of sport because if the student didn’t disperse the energy he has may lead to dispersed in others things. Regarding the clubs, for example; I used to be a member in one of the Qatari clubs I was with them for 8 years. In the club there are 30 players; only 3 of them are residents and they ask for 27 Qatari players. For sure it’s so difficult to have 27 Qatari players within the team, so you may in the team 11 players and they don’t come for the training or anything but they come on game’s times. For example daily they arrive to the Qatari’s clubs; a residents young’s or students but the Qatari’s law deterred them from being/ presence in the club and that thing let them to play in the streets or some of them may stop permanently from practicing sport. An evidence for my words; I knew some guys they were they had the ability to reach the Qatari national team because of their strong skills in the sport but because of these laws that stopped them; no *Mash-Allah* they’re over 160kg of weight. Eating and sleeping that’s. The exercising was prevented. So if there was a development in the laws and revised; may be the resident in Doha may be in the Qatari team. It may enhance the sport and would develop it for better, these are the most important. Regardless we must focus on the schools and children because we have the biggest overweight percentage ever in Qatar among children. This is a big problem it may lead them to have diabetes, diseases and hypertension are because of no movement. We must encourage them because it so hard to... The European’s community has the culture of taking care of self but in Arab no and vice versa once they go back home from school they find a dish of rice he start to eat and sleeps no this is true no this is real. So may find most of the students are *Mash-Allah* and the weight is… so I believe if we took care of these things I think we we’ll have a healthy environment and better.

Male 5: there is small point; now in the universities in the State of Qatar they do walking races and these things. Also now the star to do the school lounges of football the state is working on that but the parents didn’t develop things in the children’s mind whatever you do 100 competitions there will not be a difference. The proof of what I say. They lease the school lounges for high school you’ll find 300 or 400 students and the participants barely are 10. Why is this; because there are laziness and idle. They don’t want to participate, they don’t want to play. The state has no fees for it, no nothing. Everyone can participate in the school lounges. Any player anyone can reach to the top. But we especially we in school are the important. In the future this child will grow up into a man no one will come and talk to him; maybe he is going to be responsible about his family, about himself. So since he was fat not taking care of himself; his health; his sport so when get old it won’t help him [Male 1Interruption: he spend more time with his parents] so if there are specially lectures in the school or if there are programs in T.V

M: The problem these programs in the T.V they talk about the diabetes 1 or 2 hours and that is it, but the problem the children don’t understand anything, but if you aware them in different ways; aware them by the role of sport.

M: Ok God bless them but still we need to focus on children because these children are going to be leaders. If those children haven’t the mentality and thoughtful power in the future they can’t lead or became leaders in the future. The state will relay on them in everything; in Qatar they depends on 20 -30 % in sports so if there was no development in the children who are going to be a leader. It’s a disaster

Interviewer 2: sorry but earlier you mentioned that in the school they have one sport class per week. Do you think this things is because unavailability of the buildings or if they make sport to be as a subject of success or drop-outs. I mean you won’t be able to upgrade you to higher level unless you get a certain score?

Male 5: well this might be a good suggestion for the sport class.

Male 5: no the sport class is scheduled to be once weekly between the all classes so the playground is available

Interviewer 2: So the buildings are available but the issue is the lack of attention to the sport itself

Male 5: yes exactly even the sport teacher comes holding the ball and then he thought t to them go and play and after 20 or 30 minutes he comes again asking to them go. Ok but some student doesn’t like the football, why do you force them to play this game. In the hottest weather you ask them for a walk. Many injuries happened am talking from the reality. Even the teachers have no experiences but you may find… for example if they increase the number of sport class to be 2 or 3 par of it to be theoretical and the other part is practical so the student would have the sport mentality. Teach them the games and so many other things. Honestly I finished the high school 12 years ago and through it; the coach gives them the ball and tell them to go and play [Male 1: Interruption: yes right]. Even some students keep waiting for the sport class so they can go and have fun somewhere else…

M: such as awareness programs for group age (1-20yrs) that is vertically, whereas horizontally in providing training for awareness,

M: So you need to build in him the exercise culture and give him sport classes

M: At an individual level we need to merge between sport and health awareness from child hood for example, if he did not practice sport or became an athlete person

M: Thus Awareness at early age is a primary factor exactly the same as eating well so you have a healthy life, this is from an individual level.

M: On society level, the awareness for those who don’t know and to provide facility to motivate society such as the sport day and to make it at regular base as well encourage active live. One sports day per year is not enough; it has to be always there and to encourage active life style in general.

Male1: my suggestions to divide them to sections. First government role in media and in education, secondly society role, which doesn’t mean the individuals, it means the organization. There are certain organizations that do educational awareness for example Aspitar hospitalas organization to educate society about sport and health. They have to provide lectures and workshops to themselves and the society. So if we took the role of the government and the public organizations as well as the individuals to the young people, and not like me because I am old now, so I take care of my kids and teach them to prevent the mistake that happened to me and correct it in them. These are the suggestions and if we did that, exercise will be a primary part in our life. so each person, organizations and government will deliver this knowledge to his family and within ten years the society will move from using cars to healthy society who prefers walking . Any society need to raise awareness on sport culture so sport will become part of his life and this is for any culture, it’s like religion, if he was not raised by it, it won’t be part of him.[Interruption: doors open, moving the chair]

Male 4:On long run investment, it will impact on society for example, the treatment cost of diabetic or any other diseases, and so it is profitable by all measures. Do not belittle a club that will be suitable with the weather. If you did that, you will pay money on those who are diabetic, stroke or other chronic diseases.

M: but education pamphlet and booklets can help in improving people health and well as their awareness.

a- **Awareness in all organizations and institutions**

F5: now I see that obesity is increased; children, teenagers, young people, so there should be, for example in the university, I give examples all in the university

F5: if they close between the buildings this would encourage the girls to walk not use the bus because of the heat, for example. Or they would increase the number of contest like the biggest winner; add more contests; another contest for losing weight, another contest for fitness, add more sports; football, things like this, there should be something that would encourage us to do sports. Not sitting and using the bus to go to another building and coming back. There is not any movement at all.

Male1: there are universities here in Qatar set up a day for a championship for all the sports. For example there is volleyball and there are 20 teams in the universities and there is playoff between them. I heard about the strong man. They started with light weight with 15 participates and they start to elevate it. Then they increase the weights and elevate until they reach the last 2 or 3 participates and they playoff until they reach the maximum. They give awards and academic recommendations and you will have it together with the graduation certificate.

M4: in my opinion the best ~~the~~ thing that motivates most is the availability of sports clubs. In each club there should be someone who will give you the correct awareness. When you are doing exercise alone you can do something extra or decrease an activity, on the other hand, when you are in a club and there is a doctor who knows what is excess in your body or less, he would also know how many time per day for exercise you need so you won’t feel exhausted.

M1: Other than awareness. To increase it at schools, make PE classes more, increase the importance of doing exercise at school. For example, for me, at my school, if there was a program after school, an exercise program, that would be the beginning, from here. Let say leave the school hours, the school schedule can finish at 1:30-2:00pm, and for a certain days in the week, the student can stay from 2:00 to 4:00pm even till *Maghrib* (afternoon 5:00pm), there is no problem, there is transportation where he can go back home and at the same time he is under school care, he can be in the exercise programs, games. This is because the tournaments at schools in different sports is there so if we put something like that at schools, there will be challenge and this will grow and the student will have the talent and in the end this will appear as all the people would be doing exercise and the awareness will increase. In addition, the country will find talents in different sports; this is because it started from school. Physical activity is 2 to 4 hours per week. You can bring up a talented footballer who would be the world champion in football, for example.

M5: for me regarding the suggestions, for example, the time that we are now in, most of them go to hospitals and health centers, and they treat with medicine and others, meanwhile the disease like hypertension and diabetes and most of the diseases that can be treated mostly by exercise. Hypertension can be treated by decreasing weight, which will decrease blood pressure and pressure in the arteries, for example if this patient practiced exercise, he will decrease in his weight and so will increase in the percentage that he will get rid of hypertension or the percentage of not getting the disease. The health centers and the hospitals should add the exercise part in them not only the medicines, because most medicines have side effects, it is used but has side effects, so we as health care facilities, should add this part as an exercise program for the hypertensive and the diabetic patient.

M4: things make you lazy to practice sports are the people, family, lack of encouragement, or lack of arranged facilities , also you are not doing the outcome for sports its self , its vary according to your interest, according to outcome you want it. I’m not practicing because I don’t want to live….

M1: first of all “Healthy mind is in a healthy body”, second of all provide places for sports only across the country, and to tell people to practice sports for your own health.

F: Of course the kids, we have now obese kids. There is increase in obesity among kids, they will also need this in addition to the presence of a trainer who will encourage you to move this muscle.

F4: or would provide awareness in schools and universities.

M3: the country should use its power; first thing to explain the aim of doing sports,

F4: that what? The sister here said she wishes in the work place to be there a special place for sports so that one wouldn’t get bored, in the time of the break he could go move in that time, first thing he will refresh his activeness and Sobhan Allah sports give you new thoughts and new productivity and makes you fresh so this helps better in regards to productivity at work and not being lazy

Interviewer1: and you what is the suggestion that we can offer or Qatar states regarding the sports?

Female 1: Regarding the sports hereMash-Allahthey did their best. They did it well. They create the “Sport Day”.In the school the sport class became very important. The boys are very keen about it and the teacher as well...

Female 2: The obese kids like my daughter and my daughter-in- lowMash-Allahthey register them in Aspire. They sent us the paper telling us they want them to join. They started to follow and check-up for the diabetes. Honestly we thank them the schools for thatMash-Allah.

Female 3: Even the teacher told me that your son is fatand he has to join...but she did not register him. She told me he has to eat healthy food and since that timewe stopped eating eggs, nuggets or burger and we start to eat healthy food. She told me when he grow up he will become tired. He is in the first grade now when she saw him he used to be fat although the food at the school is healthy.

Female 4: I will complete what she said even in the school they don’t sell candies, or chips or Pepsi for children. And then they sell things with good advantage like cheese and also the fruits.My daughter told me that they sell fruits and vegetables in school.

Female 3: apples, cucumber, carrots all available in the school.

Female 4: all of these things are good they encourage the children themselvesfor healthy food may; God guide them.

Female5: HMC made a program about the 500. I already register and this already consider as encouragements for the employees. It was nice action from them to encourage the employees. I think if every occupation department, not only HMC, but also the schools, private organization should encourage their employees. If the organization cut 200- 300 QR from each employee’s salary to register him in fitness club to encourage them.

M: At an individual level we need to merge between sport and health awareness from child hood for example, if he did not practice sport or became an athlete person

M: Thus Awareness at early age is a primary factor exactly the same as eating well so you have a healthy life, this is from an individual level.

Male1: my suggestions to divide them to sections. First government role in media and in education, secondly society role, which doesn’t mean the individuals, it means the organization. There are certain organizations that do educational awareness for example Aspitar hospitalas organization to educate society about sport and health. They have to provide lectures and workshops to themselves and the society. So if we took the role of the government and the public organizations as well as the individuals to the young people, and not like me because I am old now, so I take care of my kids and teach them to prevent the mistake that happened to me and correct it in them. These are the suggestions and if we did that, exercise will be a primary part in our life. so each person, organizations and government will deliver this knowledge to his family and within ten years the society will move from using cars to healthy society who prefers walking . Any society need to raise awareness on sport culture so sport will become part of his life and this is for any culture, it’s like religion, if he was not raised by it, it won’t be part of him.[Interruption: doors open, moving the chair]

Male 4:On long run investment, it will impact on society for example, the treatment cost of diabetic or any other diseases, and so it is profitable by all measures. Do not belittle a club that will be suitable with the weather. If you did that, you will pay money on those who are diabetic, stroke or other chronic diseases.

b- **Awareness at school**

Female 1: Regarding the sports hereMash-Allahthey did their best. They did it well. They create the “Sport Day”.In the school the sport class became very important. The boys are very keen about it and the teacher as well...

Female 2: The obese kids like my daughter and my daughter-in- lowMash-Allahthey register them in Aspire. They sent us the paper telling us they want them to join. They started to follow and check-up for the diabetes. Honestly we thank them the schools for thatMash-Allah.

Female 3: Even the teacher told me that your son is fatand he has to join...but she did not register him. She told me he has to eat healthy food and since that timewe stopped eating eggs, nuggets or burger and we start to eat healthy food. She told me when he grow up he will become tired. He is in the first grade now when she saw him he used to be fat although the food at the school is healthy.

Female 4: I will complete what she said even in the school they don’t sell candies, or chips or Pepsi for children. And then they sell things with good advantage like cheese and also the fruits.My daughter told me that they sell fruits and vegetables in school.

Female 3: apples, cucumber, carrots all available in the school.

Female 4: all of these things are good they encourage the children themselvesfor healthy food may; God guide them.

Male 4: They can aware people, the youth of diseases that can be avoided if they were playing sport and also taking care of kind of sport.

M: I feel like all the care is about the football. For sure they are people who love volleyball; they are people who love basketball. The care should be equal to all kind of sport not only for football

Male 5: For me there are suggestions because we have major and essential problems with secondary problems. First of all we must be instilling the concept of practicing sport in schools. We have schools; if you can see it; where the sport’s class is once weekly and even maybe the coach may not send the students to go out and play; although these students have energy. The student needs to disperse this inner energy in a form of sport because if the student didn’t disperse the energy he has may lead to dispersed in others things. Regarding the clubs, for example; I used to be a member in one of the Qatari clubs I was with them for 8 years. In the club there are 30 players; only 3 of them are residents and they ask for 27 Qatari players. For sure it’s so difficult to have 27 Qatari players within the team, so you may in the team 11 players and they don’t come for the training or anything but they come on game’s times. For example daily they arrive to the Qatari’s clubs; a residents young’s or students but the Qatari’s law deterred them from being/ presence in the club and that thing let them to play in the streets or some of them may stop permanently from practicing sport. An evidence for my words; I knew some guys they were they had the ability to reach the Qatari national team because of their strong skills in the sport but because of these laws that stopped them; no *Mash-Allah* they’re over 160kg of weight. Eating and sleeping that’s. The exercising was prevented. So if there was a development in the laws and revised; may be the resident in Doha may be in the Qatari team. It may enhance the sport and would develop it for better, these are the most important. Regardless we must focus on the schools and children because we have the biggest overweight percentage ever in Qatar among children. This is a big problem it may lead them to have diabetes, diseases and hypertension are because of no movement. We must encourage them because it so hard to... The European’s community has the culture of taking care of self but in Arab no and vice versa once they go back home from school they find a dish of rice he start to eat and sleeps no this is true no this is real. So may find most of the students are *Mash-Allah* and the weight is… so I believe if we took care of these things I think we we’ll have a healthy environment and better.

Male 5: there is small point; now in the universities in the State of Qatar they do walking races and these things. Also now the star to do the school lounges of football the state is working on that but the parents didn’t develop things in the children’s mind whatever you do 100 competitions there will not be a difference. The proof of what I say. They lease the school lounges for high school you’ll find 300 or 400 students and the participants barely are 10. Why is this; because there are laziness and idle. They don’t want to participate, they don’t want to play. The state has no fees for it, no nothing. Everyone can participate in the school lounges. Any player anyone can reach to the top. But we especially we in school are the important. In the future this child will grow up into a man no one will come and talk to him; maybe he is going to be responsible about his family, about himself. So since he was fat not taking care of himself; his health; his sport so when get old it won’t help him [Male 1Interruption: he spend more time with his parents] so if there are specially lectures in the school or if there are programs in T.V

M: The problem these programs in the T.V they talk about the diabetes 1 or 2 hours and that is it, but the problem the children don’t understand anything, but if you aware them in different ways; aware them by the role of sport.

M: Ok God bless them but still we need to focus on children because these children are going to be leaders. If those children haven’t the mentality and thoughtful power in the future they can’t lead or became leaders in the future. The state will relay on them in everything; in Qatar they depends on 20 -30 % in sports so if there was no development in the children who are going to be a leader. It’s a disaster

Interviewer 2: sorry but earlier you mentioned that in the school they have one sport class per week. Do you think this things is because unavailability of the buildings or if they make sport to be as a subject of success or drop-outs. I mean you won’t be able to upgrade you to higher level unless you get a certain score?

Male 5: well this might be a good suggestion for the sport class.

Male 5: no the sport class is scheduled to be once weekly between the all classes so the playground is available

Interviewer 2: So the buildings are available but the issue is the lack of attention to the sport itself

Male 5: yes exactly even the sport teacher comes holding the ball and then he thought t to them go and play and after 20 or 30 minutes he comes again asking to them go. Ok but some student doesn’t like the football, why do you force them to play this game. In the hottest weather you ask them for a walk. Many injuries happened am talking from the reality. Even the teachers have no experiences but you may find… for example if they increase the number of sport class to be 2 or 3 par of it to be theoretical and the other part is practical so the student would have the sport mentality. Teach them the games and so many other things. Honestly I finished the high school 12 years ago and through it; the coach gives them the ball and tell them to go and play [Male 1: Interruption: yes right]. Even some students keep waiting for the sport class so they can go and have fun somewhere else…

M: So if we took the role of the government and the public organizations as well as the individuals to the young people, and not like me because I am old now, so I take care of my kids and teach them to prevent the mistake that happened to me and correct it in them. These are the suggestions and if we did that, exercise will be a primary part in our life. so each person, organizations and government will deliver this knowledge to his family and within ten years the society will move from using cars to healthy society who prefers walking

M:but education pamphlet and booklets can help in improving people health and well as their awareness.

4- **Role of individual**

F1: I suggest for example each one has a sport hall in his house,

F2: I suggest that they take the hobby of doing sports as an ID, it is fun and entertaining not take it as effort and boredom, no. that it is.

F5: For me it has to be a lifestyle , to make exercise lifestyle for my health, shape not to change for worse, to know for what I’m eating, not be affected by people who are around me who eats fast food, and to be around with people who like movement and practice exercise, this is for me.

F: You don’t need someone to tell you do sports or motive you, it’s from yourself if i had time i will practice, i I’m able also i will practice also , so if person did not want to do it he will not do it.

F: the personal motive is know the benefit of sports and the physical activity and not to limited on losing weight it’s can improve mental health for human. Know the importance of sport will help you.

Female4: organize your time, prioritize first of all, Allocate Time for mental rest, because sport play major role in reducing stress ,anger, Psychological pressure, the sport relief all of that then you see what you like ,

F1: awareness to avoid staying awake whole night

F: because women movement at gulf are few , thus is necessary to reduce the carbohydrates and sugars, in cold countries have a lot of sugar and starches because they walk and do a lot of effort , so they burn energy, while we don’t burn energy because all the time we sit, thus we should reduce the amount of sugars and starches which are the most deadly poison, its deadly poison.

F3: yes, a lot of fat.

I1: ok

Female1: if there is no place to do sport. There are things to do in the house and it is very simple. We sometimes tell those people who have stairs in their houses to use it if she has no bone pain

F: I just advise people*. Ma Sha Allah* people her have housemaid or two, you may give… ask her” you cook today and I will clean today or to do something else, as for example”. For those having houses, she may go around the house; they definitely have yards in their house, to wear her shoes and to turn around. I have noticed that when I was on vacation on another Gulf country, I have seen the ladies wearing trainers and they were at mall. They turn around the mall with AC and cool. It is cheap and cost nothing and you can leave your children in a place to play in or to leave them with the housemaid and you turn around the mall. If you turn around the mall 10 times, this will be …..

Female1: exactly. She even has the headphone and she turns around the mall in fast steps, cool place, people around, no boredom, and it is exercise as well.

F:The mall also, because the mall avoids the weather problem.

M2: it is not necessary, you can in the house there is AC bring a machine you can walk on anything to walk,

F3: you will be alert to move especially in the weekend as you don’t have work and you will not say that you have work or something. You can’t make the work as excuse that you don’t have time. So you supposed to have time to move. You know what I mean.

Interviewer 2: so what ideas (thoughts) do you have?

F4: national level of course to join the sports clubs by hours, take one or two hours of our time to practice sports; swimming or walking or using sports machines and weight lifting the ones that are light in order to strengthen your bones,

M2: to make timetable so to follow and be committed to it

M3: the responsibility of physical activity doesn’t fall on the government or a particular institution, its a personal thing.

M: And the exercise is something know; the effect of the right sport that the one must put a schedule to follow not according to the mood. So if we’re under pressure because of study the one is facing certain difficulties in doing sport.
